# High contiguity de novo genome assembly and DNA modification analyses for the fungus fly, *Sciara coprophila*, using single-molecule sequencing

**DOI:** 10.1186/s12864-021-07926-2

**Published:** 2021-09-06

**Authors:** John M. Urban, Michael S. Foulk, Jacob E. Bliss, C. Michelle Coleman, Nanyan Lu, Reza Mazloom, Susan J. Brown, Allan C. Spradling, Susan A. Gerbi

**Affiliations:** 1 Department of Molecular Biology, Cell Biology and Biochemistry, Brown University Division of Biology and Medicine, Sidney Frank Hall for Life Sciences, 185 Meeting Street, Providence, RI 02912 USA; 2 Department of Embryology, Carnegie Institution for Science, Howard Hughes Medical Institute Research Laboratories, 3520 San Martin Drive, Baltimore, MD 21218 USA; 3 Present Address: Department of Biology, Mercyhurst University, Erie, PA 16546 USA; 4 KSU Bioinformatics Center, Kansas State University Division of Biology, Ackert Hall, Manhattan, Kansas 66502 USA

**Keywords:** Genome assembly, Single molecule sequencing, Long reads, Optical maps, DNA modifications, Emerging model organism, Insect genomes, Fungus fly *Sciara* (*Bradysia*) *coprophila*

## Abstract

**Background:**

The lower Dipteran fungus fly, *Sciara coprophila*, has many unique biological features that challenge the rule of genome DNA constancy. For example, *Sciara* undergoes paternal chromosome elimination and maternal X chromosome nondisjunction during spermatogenesis, paternal X elimination during embryogenesis, intrachromosomal DNA amplification of DNA puff loci during larval development, and germline-limited chromosome elimination from all somatic cells. Paternal chromosome elimination in *Sciara* was the first observation of imprinting, though the mechanism remains a mystery. Here, we present the first draft genome sequence for *Sciara coprophila* to take a large step forward in addressing these features.

**Results:**

We assembled the *Sciara* genome using PacBio, Nanopore, and Illumina sequencing. To find an optimal assembly using these datasets, we generated 44 short-read and 50 long-read assemblies. We ranked assemblies using 27 metrics assessing contiguity, gene content, and dataset concordance. The highest-ranking assemblies were scaffolded using BioNano optical maps. RNA-seq datasets from multiple life stages and both sexes facilitated genome annotation. A set of 66 metrics was used to select the first draft assembly for *Sciara*. Nearly half of the *Sciara* genome sequence was anchored into chromosomes, and all scaffolds were classified as X-linked or autosomal by coverage.

**Conclusions:**

We determined that X-linked genes in *Sciara* males undergo dosage compensation. An entire bacterial genome from the *Rickettsia* genus, a group known to be endosymbionts in insects, was co-assembled with the *Sciara* genome, opening the possibility that *Rickettsia* may function in sex determination in *Sciara*. Finally, the signal level of the PacBio and Nanopore data support the presence of cytosine and adenine modifications in the *Sciara* genome, consistent with a possible role in imprinting.

**Supplementary Information:**

The online version contains supplementary material available at 10.1186/s12864-021-07926-2.

## Background

**Fig. 1:**
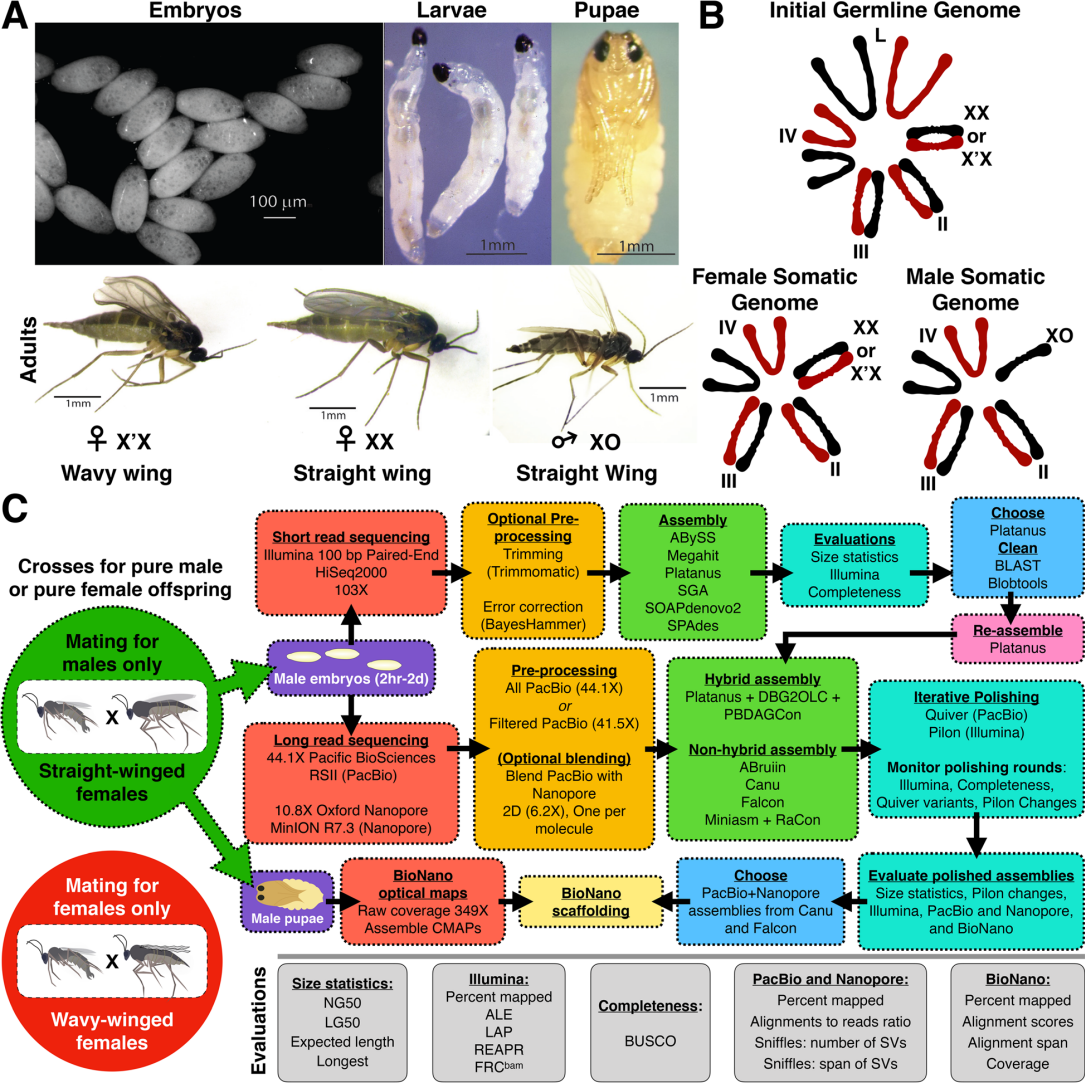
Genome sequencing and assembly strategy for *Sciara coprophila*. **a** Images of *Sciara coprophila* embryos, larvae, pupae, and adults. **b** Examples of different chromosome compositions in *Sciara* cells. Red chromosomes are paternal, black are maternal. **c** Genome assembly workflow. The green circle indicates the cross used for male-only progeny used for genome sequencing. The colored boxes show analogous steps in the different pipelines. The grey boxes name specific metrics and tools used for the category named in the turquoise boxes in the pipeline

The black fungus gnat, *Sciara coprophila* (also known as *Bradysia coprophila*), is a Dipteran fly that is both an old and emerging model organism for studying fundamental chromosome biology. Its dynamic genome gives rise to numerous research opportunities not found in the standard Dipteran model organism, *Drosophila*. The *Sciara* genome has three autosomes (chromosomes II, III and IV), an X but no Y chromosome, and germline limited L chromosomes (Fig. 1) [1]. It is ~ 280 Mb in somatic cells, ~ 363 Mb in germ cells [2] (Supplemental Table S1A-D), and is ~ 38% GC [3]. Sex is determined by whether or not the mother carries a variant of the X, called X’, that has a long paracentric inversion. Females that are XX have only sons, whereas X’X females have only daughters. The XX or X’X genotype of adult females is identified by phenotypic wing markers (Fig. 1). In contrast to the rule that the amount of nuclear DNA is constant in all cells of an organism [4], nuclear DNA in *Sciara* cells exhibits copy number regulation at the levels of loci, chromosomes, and the genome. Genomic copy number varies across cell types, from canonical haploid and diploid cells to cells with 8192 synapsed chromatids [5] that form giant polytene chromosomes where locus-specific intrachromosomal DNA amplification occurs in “DNA puffs” driven by DNA re-replication [6, 7].

Throughout *Sciara* development, specific chromosomes are targeted for copy number changes in somatic and germline cells [1]. Whereas oogenesis is canonical, spermatogenesis generates sperm that are haploid for autosomes, diploid for the X, and variable for the L. X diploidy in the sperm arises from developmentally programmed X chromosome nondisjunction in male meiosis [1]. Fertilization produces zygotes and early embryos that are temporarily triploid for the X, and variable for the L. In germline nuclei, X and L diploidy is restored through chromosome elimination events in early larval development [1]. In somatic nuclei, during early embryogenesis, all L chromosomes are eliminated, but the number of X chromosomes eliminated varies as part of sex determination. Specifically, X diploidy is restored in female somatic nuclei by the elimination of one X, but the elimination of two X chromosomes in male somatic nuclei leads to X haploidy [1]. The eliminated X chromosomes are paternally derived. Paternal chromosome elimination also occurs in the first meiotic division of spermatogenesis in the only known case of a naturally occurring monopolar spindle [1] where all paternal chromosomes, except L, are eliminated. Discrimination between maternal and paternal chromosomes in *Sciara* was the first description of “imprinting”, or an epigenetic parent-of-origin “memory” associated with a DNA sequence, in any system [8]. Two events show that L chromosomes escape imprinting: (i) all L chromosomes are eliminated from nuclei destined to become somatic cells, and (ii) none of the L chromosomes are eliminated with the paternal chromosomes during male meiosis I [9]. Although a detailed mechanism for imprinting in *Sciara* remains unknown, differences in histone modifications have been correlated [10]. It is of interest to learn if DNA modifications occur in the *Sciara* genome, since imprinting in mammals utilizes DNA methylation [11].

*Sciara coprophila* is part of an interesting and large yet little-studied suborder in the order of Dipteran flies: the Nematocera (“lower Diptera”) that contains agricultural pests and disease vectors, such as mosquitoes [12]. The other major Dipteran suborder is the Brachycera (“higher Diptera”) that includes the fruit fly *Drosophila melanogaster*. Nematocera and Brachycera diverged ~ 200 million years ago [13]. Within the Nematocera, *Sciara (Bradysia) coprophila* is classified as part of the infraorder Bibionomorpha in the Sciaroidea super family (Sciarid flies) that also contains the Cecidomyiidae (gall midges), a family that includes the Hessian fly wheat pest [14], and the Mycetophilidae, a fungus gnat family that can withstand freezing and thawing [15]. Despite flies making up at least 10% of all metazoan diversity, as of June 2021, there are only 262 Dipteran reference genomes, just 69 of which are annotated and have chromosome information [16]. Although genome assembly quality and contiguity have increased recently due to technological advances [17–19], most Dipteran genome assemblies are highly fragmented, and most are from the higher Diptera [20]. Thus, there is a real need for high quality genomes across the Dipteran tree, and particularly for the lower Diptera that includes *Sciara* [20].

We report here the first draft genome assembly for *Sciara coprophila* with gene and repeat annotations (Bcop_v1). Using tests measuring completeness, gene content, contiguity, consensus accuracy, mis-assemblies, and concordance with datasets from multiple technologies (short-read, long-read, optical maps), Bcop_v1 was selected as the best hypothesis of the underlying genome sequence out of 94 assemblies produced with different combinations of datasets, pre-processing, assembly algorithms, and parameters. Optical maps were used to scaffold a subset of the highest-ranked among the 94 assemblies using 27 such tests. A final set of scaffolds was selected using an expanded set of 66 tests that included RNA-seq-, transcriptome-, and annotation-based evaluations. In the final selected assembly (Bcop_v1), more than half the somatic genome (autosomes and X) is contained on contigs > 1.9 Mb and scaffolds > 6.8 Mb. This exceeds the contiguity of most current Dipteran genome assemblies [16]. On the release date of the *Sciara* genome (09/2020), there were just 4 Nematoceran reference genomes with annotations and chromosome information, all mosquitoes [16]. Thus, Bcop_v1 is one of only a few annotated Nematoceran assemblies anchored into chromosome maps, and the first such representation from Sciarids. Up to 49% of the genome sequence is anchored into specific loci on chromosomes X, II, III, and IV, and 100% is classified as X or autosomal. The latter allowed an analysis of dosage compensation of the single male X utilizing the first draft gene set for *Sciara*, which contains > 97% of expected gene content. The signal data from both PacBio and Nanopore suggest the presence of DNA modifications in the *Sciara* genome. Finally, a *Rickettsia* genome was co-assembled with the *Sciara* genome, suggesting it may be an endosymbiont. Overall, this work serves as the foundation for future studies on the many unique features of *Sciara coprophila*, and provides a valuable resource for future comparative genomics analyses. The *Sciara* genome is one of the highest-quality Nematoceran genome sequences available, is the only genome sequence from the Sciaridae family, and represents a phylogenetic position at the gateway between lower and higher Dipterans.

## Results

### Data collection

The somatic genome in males was targeted for the current assembly to (i) optimize the assembly of the autosomes and X chromosome by reducing the complexity introduced by the X’ and L chromosomes, and (ii) to use X haploidy in male somatic cells to partition the assembly into autosomal and X-linked sequences by coverage. Thus, the coverage, contiguity, and completeness estimates reported below are with respect to the male somatic genome (autosomes and X). To minimize complexity further, genomic DNA from washed male embryos was preferred to avoid possible complications from later life stages due to polytenization and gut microbiome contamination. To acquire male-only datasets, straight-winged *Sciara* adult females (XX) were crossed with males (XO) to produce male embryos (Fig. 1). For short-reads, 103X coverage of 100 bp paired-end Illumina data was collected. For long reads, 50-55X coverage of Pacific Biosciences (PacBio) RSII Single-Molecule Real-Time (SMRT) sequencing data and 10-11X coverage of Oxford Nanopore Technologies (ONT) MinION nanopore sequencing data was collected, referred to as PacBio and Nanopore throughout, respectively. Nearly 350X of the BioNano Genomics Irys optical map [21] coverage was collected from male pupal DNA (Table 1). Sex- and stage-specific 100 bp paired-end RNA-seq datasets were acquired from whole embryos, larvae, pupae, and adults (Supplemental Table S2).

**Table 1:** Genome sequencing datasets for *Sciara coprophila*

	Illumina HiSeq 2000	PacBio RSII	Oxford Nanopore MinION MkI	BioNano Genomics Irys
**Source**	Male Embryos	Male Embryos	Male Embryos^a^	Male pupae
**Library**	Paired-End^a^	SMRTBell	MAP002-006 (2D)	IrysPrep
**Details**	–	P5-C3	Pores R7.3-R7.3 70 bps 6mer	BssSI
**Read Length N50 (kb)**	0.1	9.681	9.934	132.613
**Mean Read Length (kb)**	0.1	6.607	5.883	62.531
**Count**	301,513,554	1,949,427	532,714	1,628,681
**Span (Gb)**	30.15	12.88	3.15	101.84
**Coverage > 0 kb**	103.26	44.11	10.77	348.78
**> 20 kb**	0	1.28	2.91	330.22
**> 30 kb**	0	0.01	1.72	323.31
**> 50 kb**	0	0	0.71	303.02
**> 100 kb**	0	0	0.28	226.1
**> 150 kb**	0	0	0.2	148.5

^a^A minority of the Nanopore data came from male adults (see Methods)

### Short-read assembly selection for the hybrid assembly approach

**Fig. 2:**
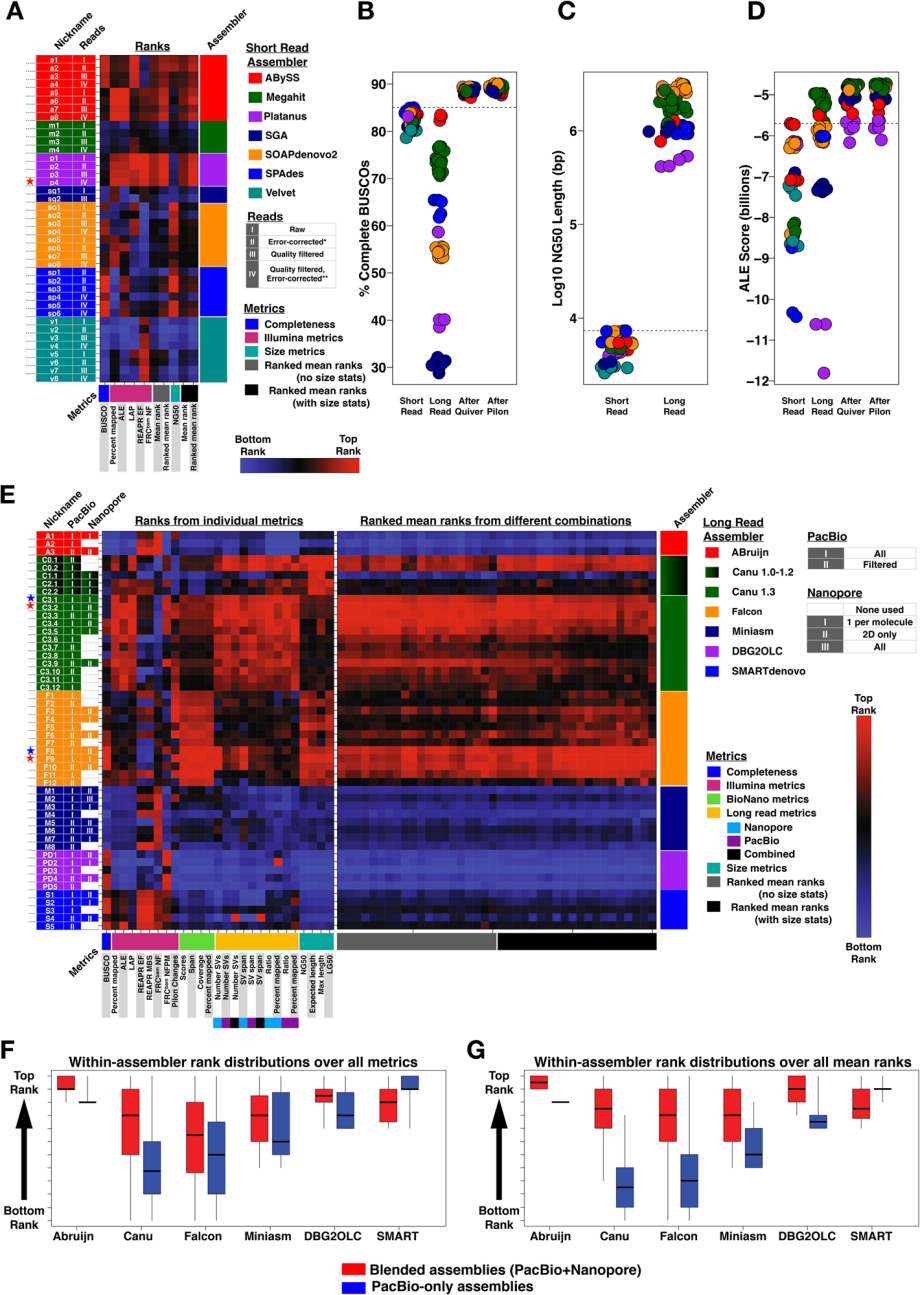
Assembly evaluations. **a** Rank matrix for 40 Illumina assemblies. Columns correspond to metrics and are organized by metric classes. Rows correspond to assemblies and are sorted by assembler. Multiple assemblies were generated for each assembler differing by the input reads, parameters used, or both. Assembly nicknames correspond to Supplemental Tables S3 and S4. Assembly ranks span from worst (blue) to best (red). The red star marks the Platanus assembly used for hybrid assemblies. **b-d** Use the short-read assembly color scheme from (**a**) and the long-read color scheme from (**e**) to visualize (**b**) percent of complete BUSCOs found, **c** Log10 NG50, and **d** ALE scores. **b** and **d** show long-read scores before and after polishing steps. Dotted lines in (**b-d**) represent the best short-read assembly. **e** Rank matrices for 50 long-read assemblies organized as described in (**a**). Columns in the left matrix correspond to individual metrics whereas columns in the right matrix correspond to mean ranks of 40 different combinations of the 27 metrics. Red and blue stars mark assemblies used for BioNano scaffolding. Red stars represent the scaffolded assemblies that were selected for gene and repeat annotation. **f-g** Box and whisker plots of within-assembler rank distributions comparing blended (red) to PacBio-only (blue) inputs. Boxplots are not comparable between assemblers. Boxes show the 25th–75th percentile, the black line is the median, and the whiskers span the range. Assemblies from a given assembler were ranked either using (**f**) all individual metrics or (**g**) all ranked mean ranks from different combinations of metrics (see left and right panels of **e**). Ranks were then partitioned into those from blended versus PacBio-only assemblies. In both cases (**f-g**), blended assemblies from all assemblers except SMARTdenovo had significantly higher ranks by Wilcoxon Rank Sum Test than PacBio-only assemblies from the same assembler

To test multiple assembly hypotheses given the Illumina data, we generated 44 assemblies using 7 short-read genome assemblers (named in Figs. 1c and 2a; details in Methods and Supplemental Materials Section 4.1.1–4.1.2). Assembly sizes ranged from 226 to 348 Mb in size (Supplemental Table S3), with a mean of ~ 280 Mb, exactly the expected somatic genome size of *Sciara*. Assemblies were ranked by gene content, contig lengths, and tools measuring the consistency with the Illumina data (metrics named in Figs. 1c, 2a-d, Supplemental Fig. S1; also see Methods and Supplemental Materials Section 4.1.3). Rankings across metrics generally correlated with each other (Fig. 2a, Supplemental Fig. S2A). Platanus and ABySS assemblies most consistently returned the best rankings with Platanus assemblies having higher mean ranks overall (Fig. 2a and Supplemental Fig. S1). Gene content was fair in most assemblies containing between 80% and 85% of the expected Arthropod BUSCOs (Fig. 2b). Nonetheless, all were highly fragmented, containing up to hundreds of thousands of short contigs (NG50 = 2.5–7.3 kb; Fig. 2c, Supplemental Table S3). The longest scaffolds of insect origin were 50–60 kb whereas bacterial scaffold lengths reached megabases, and re-assembling after removing bacterial contamination did not change this result (Supplemental Fig. S3, Supplemental Table S3, Supplemental Materials Section 4.1.4). Most short-read scaffolds were shorter than most PacBio and Nanopore reads used for long read assemblies described below (Supplemental Fig. S4). Thus, only the highest quality short-read assembly (Platanus) was chosen for hybrid assemblies with long reads to compare to long-read only approaches.

### Long-read assemblies

To test multiple assembly hypotheses given our long-read datasets, 50 long-read assemblies were generated using 6 long-read assemblers (named in Fig. 1c and 2e-g), including 5 hybrid assemblies that incorporated the chosen short read assembly above and 45 non-hybrid long-read-only assemblies (Fig. 2e, details in Methods and Supplemental Materials Section 4.2.1–4.2.3). Most long-read coverage (50-55X total) was from PacBio (44.1X; Table 1; Supplemental Fig. S4) and alone produced 21 high quality assemblies. Although there was four-fold less Nanopore coverage (10.77X), it had over two-fold and over 100-fold more coverage from reads > 20 kb and > 30 kb, respectively (Table 1). Nanopore reads were validated on PacBio assemblies (Supplemental Fig. S5). Hundreds of 1D and 2D Nanopore reads exceeding 50 kb, some > 100 kb, aligned across their full lengths to PacBio assemblies with up to 94.6% identity. A notable 131 kb 2D read aligned with 91.1% accuracy. Therefore, we also generated assemblies with blends of both long-read technologies, referred to as “blended assemblies” to distinguish them from “hybrid assemblies” that combine short-read and long-read technologies (Fig. 1c). The initial assemblies were evaluated with the same metrics used above (Fig. 2b-d, Supplemental Fig. S1). ABruijn and Canu assemblies ranked highest in most metrics (Fig. 2b-d, Supplemental Fig. S1). Nonetheless, most long-read assemblies outperformed short-read assemblies for percent error-free bases (REAPR) and had comparable or better scores in other metrics (e.g. LAP, ALE, FRC). In contrast, fewer expected Arthropod genes (< 80%) were detected in most long-read assemblies than short-read assemblies at this stage (Fig. 2b-d, Supplemental Fig. S1). The assemblies were further polished to improve upon this result.

### Long-read assembly polishing and monitoring

All assemblies were polished with several rounds of Quiver and Pilon (Fig. 1c; see Supplemental Materials Section 4.2.4). Iterative Quiver-polishing using PacBio reads progressively improved evaluations and reduced the number of variants in each assembly from millions to thousands, with the biggest impacts occurring in the first round (Supplemental Fig. S6). After the final Quiver rounds, Canu assemblies continued to rank highest, whereas ABruijn assemblies lost their lead (Fig. 2b-d, Supplemental Fig. S1). Moreover, the differences between the highest and lowest scores across assemblies narrowed in each metric. For example, 30–83% of BUSCOs were detected in the assemblies before Quiver polishing, but ~ 90% were detected in all assemblies after (Fig. 2b). Except for some hybrid assemblies, Quiver-polished assemblies outperformed the highest scoring short-read assemblies in all metrics (Fig. 2d). Non-hybrid (long-read-only) assemblies additionally outperformed hybrid assemblies that incorporated both long and short reads, even in metrics based on the Illumina dataset used in short-read and hybrid assemblies. This speaks to the high quality of contigs assembled and polished with long reads alone (Fig. 2d, Supplemental Fig. S1; “After Quiver”). Nevertheless, Pilon-polishing using Illumina reads further fixed 19.2–25.8 thousand single-nucleotide and small indel errors (~ 60–90 errors/Mb) in the first round, another 0.9–2.4 thousand (~ 3–8 errors/Mb) in the second round, and further improved evaluations (Fig. 2b, d, Supplemental Fig. S1; “After Pilon”). For example, up to an additional 1.05% of BUSCOs were detected. Overall, after polishing, metrics that reflect consensus sequence quality converged to similar scores across assemblies.

### Contig lengths and long-range integrity of long-read assemblies

The polished long-read assemblies ranged from 281.5–306.6 Mb (Supplemental Table S4), close to the expected *Sciara* male somatic genome size of 280 Mb (Supplemental Table S1) [2]. All had NG50s that were 2–3 orders of magnitude higher than that of short-read assemblies (Fig. 2c, Supplemental Fig. S1F, Supplemental Table S4). For all contig length metrics, Canu and Falcon assemblies were consistently in the top ranks. They had the highest NG50s (exceeding 3 Mb), the lowest LG50s (containing 50% of the expected genome size on just 21–23 contigs), the highest normalized expected contig sizes (exceeding 5 Mb), and the longest contigs (exceeding 20 Mb) (Fig. 2c, e, Supplemental Fig. S1F, Supplemental Table S4). An expanded set of 27 metrics that incorporated long reads and optical maps was used to determine if the longer contigs in Canu and Falcon assemblies were simply a consequence of more aggressively joining reads at the cost of more errors (metrics summarized in Figs. 1c and 2e; detailed in Supplemental Section 4.2.5). However, the opposite was true. Canu and Falcon assemblies were consistent rank leaders in the evaluations (Fig. 2e), including metrics that evaluate long-range integrity. They had the fewest putative mis-assemblies as proxied by long-read detection of structural variants (Supplemental Fig. S7J) and by BioNano map alignments, which spanned a range of 237–252 Mb in Falcon and Canu assemblies, but only 181–230 Mb in others (Supplemental Fig. S7H, S7J, S7L). These results were supported by evaluations using all four orthogonal technologies (Illumina, PacBio, Nanopore, optical maps), which produced correlated rankings (Supplemental Fig. S2B-C). Although differences were negligible, Canu assemblies led most Illumina-, PacBio-, and Nanopore-based metrics whereas Falcon assemblies led BioNano and gene content metrics (Fig. 2b, e; Supplemental Fig. S1).

### Scaffolding with optical maps

To select a final subset of assemblies for BioNano scaffolding, we sorted the assemblies by taking mean ranks across 40 combinations of the 27 metrics (Fig. 2e, Supplemental Fig. S2C). Blended assemblies that incorporated both PacBio and Nanopore reads tended to rank higher than their PacBio-only counterparts, but the largest variation amongst scores reflected the assembler used (Fig. 2f-g, Supplemental Fig. S7). Blended assemblies from Canu and Falcon were the clear rank leaders (Fig. 2e-g), and two assemblies from each were chosen for BioNano scaffolding (Fig. 2e stars).

**Fig. 3:**
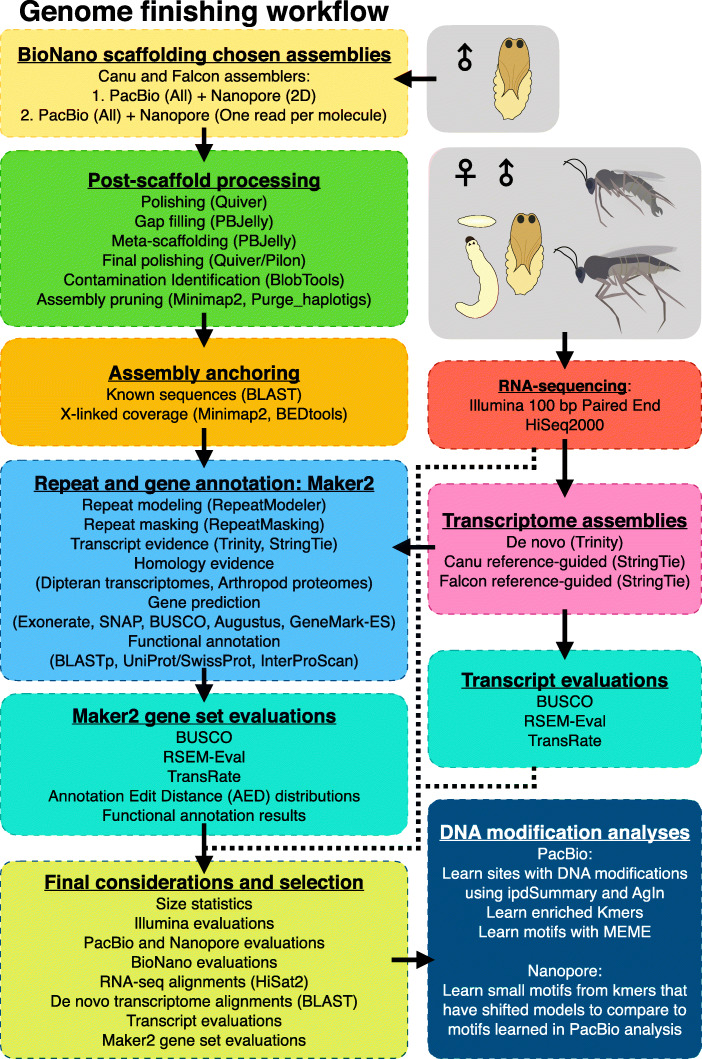
Post-assembly work flow. Chosen assemblies were scaffolded, polished, gap-filled, filtered, anchored into chromosomes where possible, and classified as X or autosomal by coverage. Repeats were identified. RNA-seq was used for transcriptome assembly and gene annotation. Single-molecule datasets were used to investigate DNA modifications

BioNano Irys optical map data from male pupae (Fig. 3, Table 1, Supplemental Materials Sections 3.6 and 4.2.6) produced a raw molecule N50 of 214.1 kb for molecules > 150 kb. The resulting genomic consensus maps (CMAPs) had a map N50 of 712 kb, a cumulative length of 325.5 Mb, which is between the expected sizes of the somatic and germline genomes [2] (Supplemental Table S1A-E), and spanned 266–278 Mb of the sequence contigs. The CMAPs and sequence contigs were used to produce “hybrid scaffold maps” (HSMs). Both CMAPs and sequence contigs spanned approximately 275–280 Mb of the HSMs. The scaffolds derived from the two Canu assemblies were nearly identical as determined by evaluations and whole genome alignments (Supplemental Figs. S8-S9, Supplemental Table S5, Supplemental Materials Section 4.2.6.4), and the same was true for HSMs derived from both Falcon assemblies. Therefore, we moved forward with only one set of scaffolds corresponding to each assembler, hereafter referred to as “Canu” and “Falcon”. Throughout the following text, Canu assembly statistics will be described with corresponding Falcon statistics in parentheses.

**Fig. 4:**
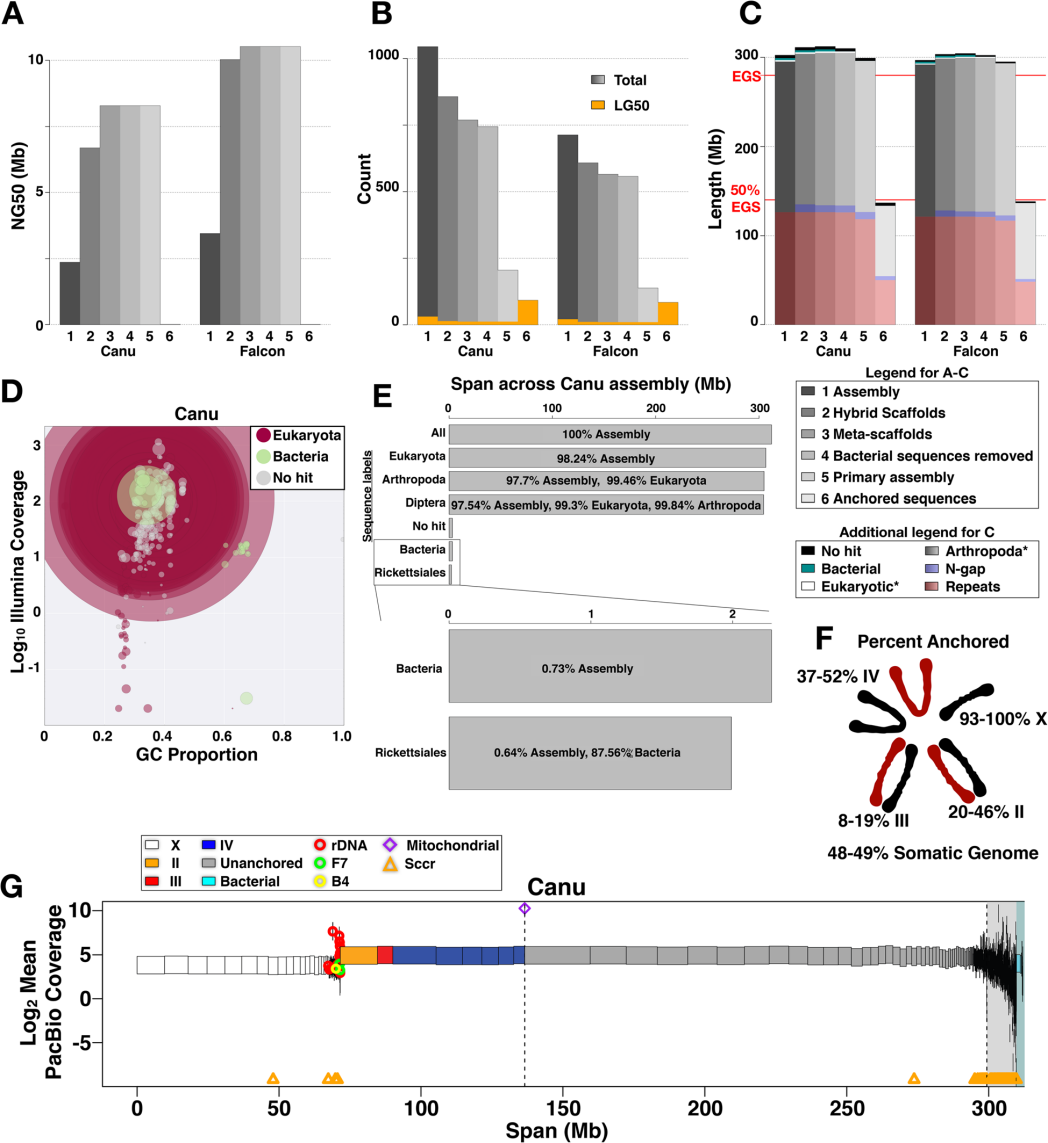
Assembly scaffolding and anchoring. **a** NG50 of the assembly at stages 1–6 as defined in “Legend for A-C” within the figure. **b** Number of sequences in the assembly at stages 1–6 as in (**a**). Orange bars = LG50. **c** Total length of the assembly at different stages 1–6 as in (**a**). The “Additional legend for C” defines colored portions of the bars. *The length of the Eukaryotic and Arthropod labeled sequences includes everything up through that color. **d** Log10 Illumina coverage versus GC content over the Canu assembly (similar results for Falcon), colored by taxonomy information, and with circle sizes proportional to the contig sizes they represent. **e** Proportion of the assembly labeled as Eukaryotic, Arthropoda, Diptera, Bacteria, and Rickettsiales. **f** Anchored percentage of the expected genome size and chromosome sizes. Ranges in Canu and Falcon assemblies indicated. **g** Canu assembly with scaffolds drawn as rectangles corresponding to their lengths, colored according to the chromosome they were anchored to (or unanchored), and on Y-axis according to mean coverage from PacBio reads. The white background highlights sequences in the primary assembly whereas the grey and cyan backgrounds are set behind associated and bacterial sequences, respectively. All sequences to the left of the first vertical dashed line are anchored. See the plot legend for symbols

BioNano scaffolding approximately tripled the contiguity of the assemblies (Fig. 4a, Supplementary Tables S6, S7). The total numbers of sequences in the Canu (Falcon) assembly decreased from 1044 to 857 (713 to 608) while increasing the NG50 of 2.3 Mb to 6.7 Mb (3.5 Mb to 10 Mb). The assembly size increased from 302 Mb to 311 Mb (296 Mb to 303 Mb) (Fig. 4a-c). The scaffolds had 187 (105) gaps summing to 8.7 Mb (6.7 Mb) with a maximum gap size of 677 kb (965 kb) and median of 20.8 kb (30.5 kb) (Supplemental Table S8).

Gaps were iteratively filled and polished using PBJelly and Quiver (see Supplemental Materials Section 4.2.7.1). In the Canu (Falcon) scaffolds, 31 (14) gaps were completely closed and over 972 kb (1.06 Mb) of gap sequence was filled in (Fig. 4c, Supplemental Table S8). In the final round, “meta-scaffolds” were constructed using connections from long-read alignments. This decreased the total number of sequences from 857 to 769 (608 to 565) and increased the NG50 of 6.7 Mb to 8.3 Mb (10.0 Mb to 10.5 Mb) and the assembly size from 311 Mb to 312 Mb (303 Mb to 304 Mb) (Fig. 4a, Supplemental Table S6, S7). We used Quiver and Pilon to correct errors in the gap-filled meta-scaffolds. In the final round, Pilon made only 18 (27) changes to the consensus sequence, translating to 1 change per 16.9 Mb (11 Mb) of non-gap sequence.

### Assembly cleaning

BlobTools was used to identify contaminating contigs in the final scaffolds by separating sequences by coverage and GC content (Fig. 4c-e, Supplemental Fig. S10, S11, Supplemental Materials Section 4.2.7.2). *Sciara* male embryo coverage from Illumina, PacBio, and the Nanopore reads all gave similar results (Supplemental Fig. S10). The vast majority of the final Canu and Falcon scaffolds (> 97.7% of the total sequence length) was identified as Arthopoda, > 99% of which was Dipteran (Fig. 4c, e, Supplemental Fig. S11). Canu and Falcon had 25 and 8 bacterial contigs respectively, with total lengths of 2.0–2.3 Mb (< 1% of the total sequence length) and N50s of 1.0–1.3 Mb (Fig. 4c, d, e, g; Supplemental Fig. S11, Supplemental Table S9). There were no BioNano optical map alignments over the bacterial contigs, and accordingly no bacterial contigs attached to or found in any of the final Arthropod-associated scaffolds. Removing bacterial contigs only marginally affected contig size statistics of the *Sciara* assemblies (Fig. 4g; Supplemental Tables S6, S7).

The majority of bacterial sequence (87–96%) was labeled as Rickettsiales (Fig. 4d-e, Supplemental Fig. S11), nearly all of which was *Rickettsia prowazekii* (88.5–90.1%) and *Rickettsia peacockii* (9.9–10.8%). The N50 of these contigs was equivalent to *Rickettsia* genome sizes. Interestingly, in the Illumina, PacBio, and Nanopore datasets, the *Rickettsia* genome has nearly the same coverage as the *Sciara* genome (Fig. 4d, g, Supplemental Fig. S10), indicating ~ 1 *Rickettsia* genome per haploid *Sciara* genome in male embryos. No *Rickettsia* optical maps from male pupae were observed.

After removing bacterial sequences, each assembly was partitioned into “primary” and “associated” sequences (scaffolds and contigs; see Supplemental Materials Section 4.2.7.3). Primary sequences represent one haplotype of the genome whereas associated sequences are short redundant contigs (haplotigs) that represent other haplotypes of heterozygous loci (Fig. 4g). Canu (Falcon) contained 744 (557) sequences, 205 (138) primary and 539 (419) associated, giving a primary assembly size of ~ 299 Mb (~ 295 Mb) with ~ 13 Mb (9.4 Mb) of associated sequences (Fig. 4a-c, Supplemental Tables S6, S7). The associated sequences are numerous and generally short (mean = ~ 23 kb). In contrast, compared to all sequences, the mean length of sequences in the Canu (Falcon) primary assembly increased from ~ 416 kb to 1.5 Mb (542 kb to 2.1 Mb), although NG50 stays the same (Supplemental Tables S6, S7). The difference of ~ 4 Mb between the Canu and Falcon primary assembly sizes is in part owed to Canu having ~ 2.2 Mb more gap length than Falcon.

### Assembly anchoring

Previous in situ hybridization results (Table 2) were used to anchor 7–8 primary autosome-linked sequences from each assembly that sum to 64.9–75.6 Mb (Fig. 4g; Supplemental Materials Section 4.2.7.4). Based on polytene banding patterns [22], chromosomes II, III, and IV are approximately 62–66 Mb, 66–71 Mb, and 88–94 Mb, respectively (Supplementary Table S1E). Thus, 20–46% of II, 8–19% of III, and 37–52% of IV, 28–33% of all autosomes, and 23–27% of the expected somatic genome size have been anchored with unique sequences (Table 2). Between 1 and 2 Mb of X-linked contigs was anchored using repetitive sequences specific to the X (Table 2, e.g. rDNA, Fig. 4g). In addition, the “Sccr” (*Sciara* centromere consensus sequence) repeat that hybridized to the centromeres of all *Sciara* chromosomes [23] mapped to 48–105 contigs (Table 2, Fig. 4g).

**Table 2:** Anchoring into chromosomes using previously known sequences

Sequence	Location	Canu contig size	Falcon contig size	Reference
DNA puff II/9A	Chr II locus 9A	13.1 Mb	28.5 Mb	[124–127]
RNA Puff III/9B	Chr III locus 9B	5.4 Mb	12.5 Mb	[126, 128]
Ecdysone receptor	Chr IV locus 12A	3.8 Mb	9.6 Mb^a^	[129]
Ultraspiracle	Chr IV locus 10A	9.3 Mb	5.5 Mb	[129]
Hsp70	Chr IV locus 4A or 12C	5.4 Mb	13 Mb	[130]
Hsp70	Chr IV locus 4A or 12C	6.8 Mb	2.6 Mb	[130]
ScoHet1	Chr IV locus 5A	15.2 Mb	(9.6 Mb)^a^	[57]
ScoHet2	Chr IV locus 12C-13A	5.9 Mb	4 Mb	[57]
rDNA	Chr X locus 1A	5 primary contigs and 11 associated contigs (Σ 1.3 Mb)	2 primary contigs and 41 associated contigs (Σ 1.7 Mb)	[3, 131–133]
Microclone B4	Chr X locus 1A	69.8 kb	59 kb	[23]
Microclone F7	Near centromere of Chr X^b^, non-centromeric Chr IV, L chromosomes	3 associated contigs (Σ 66.8 kb)	1 primary and 1 associated contig (Σ 161.6 kb)	[23]
Microclone G2 (Sccr)	Centromeres of all chromosomes	20 primary and 85 associated contigs (Σ 1.3 Mb)	6 primary and 42 associated contigs (Σ 604 kb)	[23]

^**a**^Ecdysone receptor (EcR) and ScoHet1 identified the same 9.6 Mb contig in Falcon. The locus inconsistency may represent a misassembly in Falcon or misannotation from Greciano et al. [57]. Nevertheless, both EcR and ScoHet1 results agree it is from chromosome IV

^b^ Coverage analyses confirm contigs with F7 as chromosome X sequence

Since male *Sciara* embryos are X haploid and autosomal diploid, X-linked contigs were defined as primary contigs with > 80% haploid coverage (Fig. 4g; Supplemental Materials Section 4.2.7.5). The Canu (Falcon) assembly contained 69 (36) X-linked (haploid) contigs that summed to 71 Mb (62 Mb) with an N50 of 5.95 Mb (7.3 Mb). The longest X-linked contig was 9.68 Mb (12 Mb). The set of haploid X-linked contigs contained those identified as X-linked using X-specific sequences above as well as contigs containing the F7 repeat sequence known to be on X, IV, and L [23] (Table 2, Fig. 4g, Supplemental Fig. S11C). The X chromosome is estimated to be ~ 50 Mb based on DNA-Feulgen cytophotometry or ~ 62 Mb based on the number of polytene bands [2, 22] (Supplementary Table S1 A-E). Therefore, most or all of the X chromosome was anchored. In total, at least 136.6–138.0 Mb of *Sciara* sequence, or ~ 49% of the expected somatic genome size, was anchored into specific chromosomes with 100% of the assembly characterized as either X or autosomal.

### Repeats in the *Sciara* genome

**Fig. 5:**
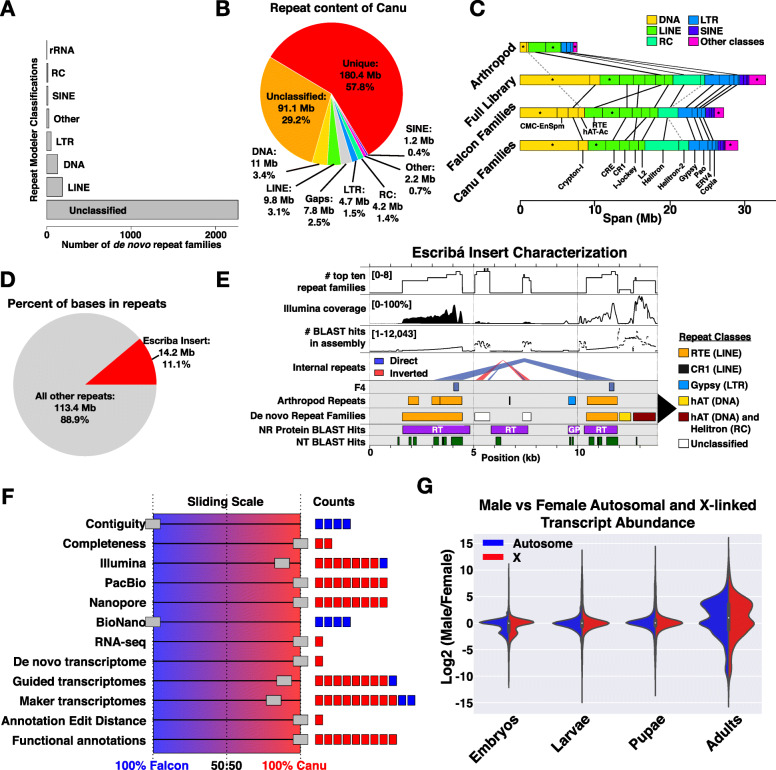
Repeats and genes in the chosen assembly. **a** Classification and counts of de novo repeat families trained on Canu. **b** Partition of Canu into the major repeat categories (DNA = DNA transposons). **c** Major sub-classes of repeats in each repeat class in Canu when masking with different repeat libraries. Boxes with asterisks are all other sub-classes. **d** Number of bases masked by the Escribá insert [23] compared to all masked bases. **e** Characterization of the Escribá insert, highlighting major repeats in the *Sciara* genome. Black arrowhead on right pointing to repeat classes legend corresponds only to the two repeat family rows. F4 = ScRTE probe in Escribá et al. [23]. RT = reverse transcriptase. GP = gag-pol. **f** Ranking results of the final two assemblies showing the number of metrics in each category each assembler scored better on. **g** Distributions of log2 male/female transcript abundance ratios for X (red) and autosomes (blue) across multiple life stages

RepeatModeler identified 2695 (2661) repeat families in Canu (Falcon), of which 15 (19) were classified as SINEs, 186 (160) as LINEs, 53 (48) as LTR, 131 (130) as DNA elements, and 43 (50) as other repeat classes (Fig. 5a, Supplemental Fig. S11D, Supplemental Tables S10, S11; Supplemental Materials Section 4.3.3), leaving most repeats unclassified. These were combined with previously known repeats from *Sciara* and other arthropods to make a comprehensive repeat library (CRL) for RepeatMasker [24], which classified ~ 121–126 MB (39–41%) of the assemblies as repeats (Fig. 5b, Supplemental Fig. S11E, Supplemental Tables S12, S13). Most repeats (93.3–96.7 Mb; 76.6–76.9%) were unclassified (Fig. 5b). SINE, LINE, LTR, RC, and DNA elements each constitute 0.4–3.4% of the assemblies (Fig. 5b). DNA elements had the largest total span with Crypton-I the largest sub-class therein (Fig. 5c), but RC Helitron elements was the largest sub-class overall (Fig. 5c). Simple repeats made up ~ 1% of the assemblies (Supplemental Table S12). Components of the CRL gave similar results (Fig. 5c), but arthropod repeats proportionally identified LINE RTE elements to be most abundant (Fig. 5c). Assuming scaffold gaps are repetitive, 180 Mb (58%) of the *Sciara* genome (Canu) is unique (Fig. 5b).

Escribá et al. [23] published a 13.8 kb lambda phage insert sequence containing two copies of an RTE-related transposon (ScRTE) that they localized to pericentromeric regions of all *Sciara* chromosomes (see Supplemental Materials Section 4.3.7). There was only one full-length copy of the lambda insert in each assembly (Supplemental Fig. S12), but pieces of it are scattered across the assembly totaling nearly 60,000 alignments that span 14.2 Mb, or ~ 11% of bases labeled as repeats (Fig. 5d-e). Of the top ten most abundant de novo repeat families, eight map to the Escribá insert across most of their length and correspond to the direct repeats of the ScRTE element near the 5′ and 3′ ends, the central unclassified inverted and direct repeats, and the hAT and Helitron elements at the 3′ end (Fig. 5e). It is possible that contigs with high densities of the Escribá ScRTE probe (F4) are pericentromeric. These contigs are typically highly repetitive and full of degenerating transposons, including but not limited to ScRTE. Other repeats on the insert are not restricted to pericentromeric regions. For example, the super abundant central inverted repeats are found in known euchromatic regions, including DNA puff II/9A.

### Gene annotation and final assembly selection

Protein-coding genes in the Canu and Falcon genome assemblies were annotated with Maker2 guided by transcriptome assemblies from poly-A enriched RNA-seq datasets from male and female embryos, larvae, pupae, and adults (Fig. 3; Supplemental Materials Section 4.3). A final round of evaluations was performed, using 66 metrics partitioned into 12 categories, to choose a single assembly to release as Bcop_v1 (Fig. 3 and Fig. 5f; detailed in Supplemental Materials Section 4.3.5). Falcon had a slight lead in contig size statistics and optical map alignments (Fig. 5f, Supplemental Fig. S13). Canu led in metrics for completeness, RNA-seq and de novo transcriptome alignments, and from Illumina, PacBio, and Nanopore datasets (Fig. 5f, Supplemental Fig. S13). Moreover, both the Canu-guided transcriptome assembly and the transcripts in the final Canu annotation had better evaluations (Fig. 5f, Supplemental Tables S14, S15), and the latter had lower annotation edit distances, more genes with GO terms, Pfam domains, and/or BLAST hits in the UniProt-SwissProt database, more BUSCOs, and more hits from *Drosophila melanogaster* and *A. gambiae* proteomes (Fig. 5f, Supplemental Fig. S14, Supplemental Table S16). The Canu assembly won 54 of the 66 metrics, and 10 of the 12 categories (Fig. 5f), indicating that it had higher consistency with the genome sequencing datasets and yielded the superior gene set, and was therefore chosen as the first draft genome for *Sciara* (*Bradysia*) *coprophila*, and named Bcop_v1*.*

The final annotation of the Canu assembly (Bcop_v1) had 23,117 protein-coding gene models with 28,870 associated transcripts (Supplemental Table S15A). *Sciara* has more genes than the 17–18,000 genes of the Brachyceran, *Drosophila melanogaster* (http://ftp.flybase.net/genomes/Drosophila_melanogaster/dmel_r6.40_FB2021_03), but a similar amount as the 23,884 found in the house fly, *Musca domestica* [20]. Moreover, within the more closely related Nematocera, the Hessian fly, *Mayetiola destructor*, contains slightly over 20 thousand genes (https://i5k.nal.usda.gov/data/Arthropoda/maydes-%28Mayetiola_destructor%29/GCA_000149185.1) [25] and the mosquito *A. aegypti* contains 19.2 thousand [12]. Still, the high number of genes found for *Sciara* may also be a result of gene splitting in the annotation. To increase the quality of the *Sciara* gene set, the annotation was deposited at the i5k-workspace for community-enabled manual curation [26]. Nevertheless, the annotation contains nearly all expected Dipteran genes: 94.2% complete Dipteran BUSCOs, 97% when including fragmented BUSCOs (Supplemental Fig. S14E, Supplemental Table S15A). Most genes in the annotation (87.5%) had only a single transcript isoform (Supplemental Fig. S14B). The median gene and transcript lengths are ~ 2.6 kb and ~ 1.3 kb, respectively (Supplemental Table S15A), with a median of 4 exons, ranging from just one (10.8% of genes) to over 100 exons. Both 5′ and 3′ UTRs were annotated for 10,801 genes, and one or the other for 13,335. Exons, introns, 5′ and 3′ UTRs had median lengths of 182 bp, 80 bp, 165 bp and 184 bp, respectively. Functional information was identified for ~ 65% of the genes: 8671 (37.5%) have Ontology Terms; 13,745 (59.5%) have UniProt/SwissProt hits; 13,789 (59.6%) have Pfam descriptions [27]; 8252 (35.7%) have all three; and 14,961 (64.7%) have one or more (Supplemental Fig. S14F, Supplemental Table S16). Genes spanned over 54% of the Canu assembly (Bcop_v1), mostly attributable to introns, and ~ 20% was both unique and intergenic (Supplemental Fig. S14H).

NCBI also ran the NCBI Eukaryotic Genome Annotation Pipeline on Bcop_v1 (Canu) to create a set of annotations, named “NCBI *Bradysia coprophila* Annotation Release 100”, for the RefSeq database [28]. NCBI found 20,106 genes and pseudogenes of which 16,546 are protein-coding, and found similar length statistics for features such as genes, transcripts, exons, and introns, as well as similar count statistics for features such as transcripts per gene, exons per gene, alignments to *Drosophila* genes, etc. The NCBI annotations will also be used to inform manual curation on the i5k workspace [26].

### Querying male X dosage compensation using the gene annotation

In the standard Dipteran model, *Drosophila melanogaster*, where males are XY and females are XX, male flies exhibit dosage compensation of transcripts from X-linked genes. We used the *Sciara* gene annotations and anchoring information to explore dosage compensation in *Sciara* where males are XO and females are XX. Genes were defined as X-linked if they were on contigs anchored into the X chromosome as described above. If dosage compensation does not exist, then most X-linked genes would be expected to have 2-fold lower transcript abundances in male samples. Across each stage of development sequenced, the distributions of log2 fold changes between male and female transcript abundance were the same for autosomal and X-linked genes (Fig. 5g, Supplemental Fig. S15; Supplemental Materials Section 4.3.6). There were many examples of both autosomal and X-linked genes that were differentially expressed between males and females, but there was no difference between males and females for most genes in both classes. Therefore, the existence of dosage compensation of most X-linked genes in *S. coprophila* is strongly supported in agreement with previous autoradiographic data in a related species, *Sciara ocellaris* [29].

### DNA modification signatures in single-molecule data

**Fig. 6:**
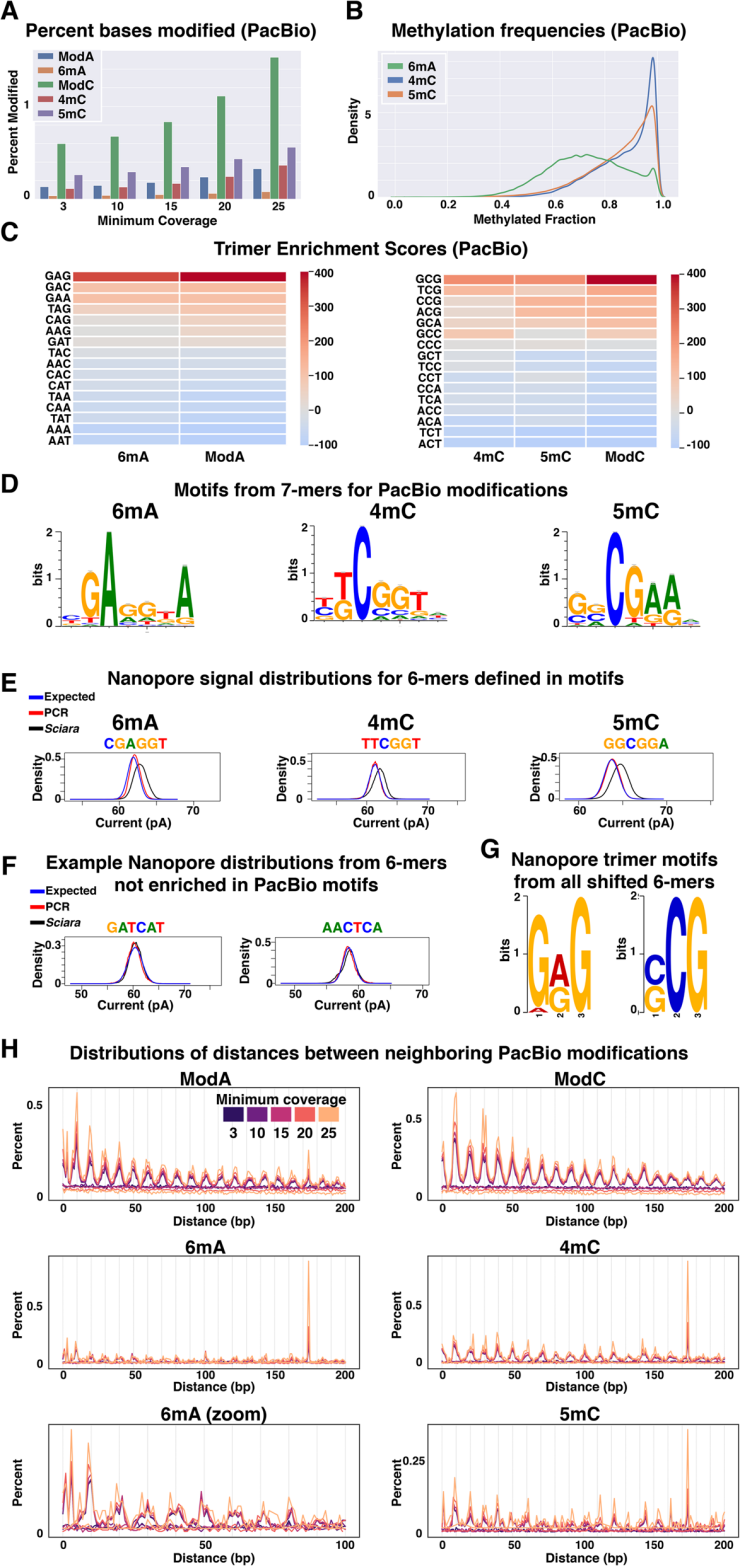
DNA modifications in male embryo genomic DNA of *Sciara coprophila*. **a** Percent of adenines or cytosines assigned to a modification class given a minimum coverage level in the PacBio analysis. ModA and ModC are the sets of all adenines or cytosines, respectively, flagged as modified whereas 6mA, 4mC, and 5mC are the subsets therein with those specific classifications. **b** Methylation frequencies at modification sites in PacBio analysis. **c** Chi-square standardized residuals (enrichment scores) indicating how many standard deviations away each observation is from expectation for trimers with middle adenines or middle cytosines from PacBio analysis. **d** Position weighted motifs from sets of 7-mers (where the modified base occurs at position 3) enriched for 6mA, 4mC, or 5mC. **e** Distributions of ionic current means from Nanopore template reads for 6-mers defined by PacBio motifs in (**d**). Blue line shows expected distribution given the Nanopore model for each kmer. Red line shows distribution learned from whole *E. coli* genome PCR data [34] using only canonical nucleotides. Black line shows distribution learned from native *Sciara* genomic DNA. **f** As in (**e**), but showing examples of 6-mers not defined by motifs learned in the PacBio analysis. **g** Two of the top three trimer motifs learned from the set of all 6-mers with shifted Nanopore signal distributions compared to expected models. **h** Distributions of distances between neighboring DNA modifications on the same strand

Since imprinting in mammals utilizes DNA methylation [11] and the *Sciara* transcriptome contains proteins involved in cytosine and adenine methylation pathways found in other Dipterans (reviewed in [30–32]) (Supplemental Table S17A-C), we determined if DNA modifications are present in the *Sciara* genome of male embryos using the single-molecule datasets (see Supplemental Materials Section 4.4). PacBio SMRT kinetics analysis revealed that 0.6–1.1% of cytosine sites were modified with 0.11–0.24% and 0.26–0.43% showing 4-methylcytosine (4mC) and 5-methylcytosine (5mC) signatures, respectively, and flagged ~ 0.13–0.24% of adenine sites as modified with ~ 0.04–0.06% of adenine sites exhibiting the 6-methyl-Adenine (6mA) signature (Fig. 6a, Supplemental Table S18A and S18C). Modified cytosines and adenines were found throughout the *Sciara* genome (both autosomal and X-linked), including in genes and repeats (Supplemental Fig. S16A-C). Most 6mA sites and many 4mC and 5mC sites had methylation frequencies > 50 and > 80%, respectively (Fig. 6b).

Adenine and cytosine modifications were found in many contexts, but AG and CG dimers as well as GAG and GCG trimers were most enriched (Fig. 6c-d, Supplemental Fig. S16D, Supplemental Tables S19, S20). GAG sites were modified 7–8 times more frequently than the rate for A alone (Supplemental Table S18B). Enriched 7mers showed a prominent 4 bp GAGG motif (Fig. 6d), which did not differ between X and autosomal sequences (Supplemental Fig. S17). Other 6mA-associated motifs included CAG within them (Supplemental Fig. S18). The PacBio kinetics analysis flagged ~ 1.3–2.5% of CpG dinucleotides as modified with 0.26–0.57% and 0.55–0.96% classified as 4mCpG and 5mCpG, respectively (Supplemental Table S18D). A more sensitive algorithm [33] flagged up to 6.4% of CpG dinucleotide sites as methylation targets (Supplemental Table S18E). The rate at which GCG sites were flagged as modified (2.5–4.9% total; 0.5–1.2% 4mC; 0.9–1.5% 5mC) was 4–5 times more frequently than the rate for C alone and 2 times more than CG (Supplemental Table S18F). Interestingly, GCG is depleted and GTG is enriched in both the genome and transcriptome, consistent with 5mC deamination to thymine in the germline over evolutionary time (Supplemental Fig. S20; Supplemental Section 4.4.3).

The Nanopore dataset was used to test modification-associated 6-mers in the PacBio results by checking whether their ionic current distributions from *Sciara* genomic DNA conformed to their expected modeled distributions or not, the latter of which is suggestive of DNA modifications [34]. The 6mers defined in the PacBio kinetics analyses that were associated with 6mA, 4mC, and 5mC signatures had shifted Nanopore signal distributions whereas control kmers conformed to their expected models (Fig. 6e-f, Supplemental Fig. S19). Moreover, sub-motifs found in the set of all 6-mers with shifted signal distributions were similar to motifs found in the analyses of 6mA, 4mC, and 5mC sites identified in the PacBio analysis (e.g. GAG and GCG; Fig. 6g, Supplemental Fig. S18).

The distribution of distances between adjacent DNA modifications, for both C and A, was enriched for short distances with a periodicity of 10 bp (Fig. 6h, Supplemental Figs. S21–22), which is suggestive of turns of the DNA helix. Periodic spacing of 10 bp between methylation sites and target motifs has been observed enriched over nucleosome positions in *Arabidopsis* and mammals [35–37]. Moreover, 6mA was shown to be phased between nucleosomes in *Chlamydomonas* and *Tetrahymena* (reviewed in [38, 39]). In *Sciara*, ~ 175 bp is one of the most enriched distances separating two modifications (Fig. 6h, Supplemental Figs. S21–22), reminiscent of nucleosomal spacing in general and the exact length of nucleosome intervals in *Drosophila* [40].

DNA modifications (6mA, 4mC, 5mC) were random or slightly depleted in annotated protein-coding genes, exhibiting slight depletions in exons and promoters and slight enrichments in introns (Supplemental Fig. S16B, Supplemental Table S21A-B). These trends were the same when using gene locations defined by the StringTie transcriptome assembly (Supplemental Table S21C) and were generally true even when the genes were split into categories of unexpressed, lowly expressed, and highly expressed using male embryo RNA-seq data (Supplemental Table S21D). Repeat regions in the genome had more modifications than expected and conversely unique regions had fewer (Supplemental Fig. S16B, Supplemental Table S21E-F). In the de novo repeat library, there were repeat families with 2–100 fold more modifications than expected and many families with no modifications, indicating that specific classes of repeats are targeted for DNA modifications.

## Discussion

### The first *Sciara* genome sequence and its impact

We report here the first genome sequence of the lower Dipteran fly, *Sciara (Bradysia) coprophila*, as well as its gene and repeat annotation (Bcop_v1). As determined by multiple rounds of evaluations using up to 66 metrics, Bcop_v1 was consistently the best hypothesis and representation of the underlying genome sequence out of 44 short-read and 50 long-read assembly hypotheses generated from different technologies, algorithms, pre-processing, and parameters. More broadly, Canu and Falcon assemblies generated using a blend of PacBio and Nanopore data ranked highest and were selected for scaffolding with BioNano Irys optical maps. Scaffolding long-read assemblies with optical maps has also been noted by others to give excellent contiguity [19]. We found as a rule that long-read-only assemblies outperformed hybrid assemblies that included short-read contigs, although others have found that the hybrid approach was desirable with lower amounts of long-read coverage [17], or with tools that merge hybrid and non-hybrid assemblies together [17, 18].

Bcop_v1 specifically represents the *Sciara* somatic genome found in male (XO) and male-producing female (XX) somatic cells (chromosomes X, II, III, IV). The female-limited version of the X chromosome (X’) found only in female-producing females (X’X), and the germline-limited L chromosome will be the subject of future updates to this assembly. Bcop_v1 contains 299 Mb of sequence on 205 primary contigs, 10 Mb of associated contigs, 50% of the expected genome size on only 12 scaffolds ranging from 8 to 23 Mb, and a gene set with 97% of Dipteran BUSCOs detected suggesting completeness. Previous in situ hybridization data was used to anchor 20–46% of chromosome II, 8–19% of chromosome III and 37–52% of chromosome IV. Haploid coverage levels classified all sequences as X or autosomal. In total, ~ 137–138 Mb of sequence, or ~ 49% of the expected somatic genome size, was anchored into specific chromosomes. These data provide the foundation for future research with targeted approaches to study the L chromosome, the paracentric inversion on the X’ chromosome, DNA puff amplification, chromosome identity and elimination, and many other unique features of *Sciara*.

The *Sciara* genome assembly (Bcop_v1) is more contiguous than 83% of all Arthropod genomes currently described [16] and exceeds 82% of currently available lower Dipteran genome assemblies, over 60% of which have sub-100 kb N50s. The low contiguity of most Dipteran assemblies and the lack of chromosome anchoring limits their utility. The *Sciara* genome assembly may be useful for scaffolding fragmented Nematoceran genomes by synteny. The long contigs in Bcop_v1 reflect the successful use of long reads and optical maps, both of which can span repeats, and will be useful for analyzing regions of repetitive DNA, like rDNA, centromeres, telomeres, and transposable elements.

The phylogenetic position of *Sciara (Bradysia) coprophila* in the Dipteran tree makes it valuable for future comparative genomics studies concerned with evolutionary rates and patterns of genes, genomes, pathways, populations, and species [20]. Some unresolved questions remain in the field of Dipteran phylogenetics [41, 42]. Morphological criteria suggested that the Brachycera (containing *Drosophila*) and the Nematocera (containing *Sciara*) diverged from a common ancestor. However, more recent molecular data supports a model where the Nematoceran infraorder Bibionomorpha ultimately gave rise to the Brachycera ~ 200 MYA [13]. The *Sciara* genome and transcriptome will be valuable resources to further describe Dipteran phylogenetic relationships, and elucidate the evolution and molecular structure of genes and pathways in Dipterans. Furthermore, *Sciara* males are haploid only for the X but diploid for autosomes, unlike haplodiploid males in other insects that are haploid for their entire genome. This is accomplished by X chromosome elimination in the early *Sciara* embryo, noted by White [43] to occur in the Nematoceran families of Sciaridae and Cecidomyidae (including the Hessian fly *Mayetiola destructor*). Comparison of the genomes/transcriptomes of *Sciara* and *M. destructor* may elucidate the regulation of X chromosome elimination.

### Could an endosymbiont influence sex determination in *Sciara?*

The most common form of sex determination is male heterogamety (XY males, XX females), but there is also female heterogamety (ZZ males, ZW females) that is exhibited by *Sciara*: males are XO whereas females are either XX or X’X [1]. Presumably, the ooplasm is differentially conditioned by X’X and XX *Sciara* mothers, which determines whether 1 or 2 paternal X chromosomes will be eliminated, leading to only female or only male offspring, respectively. Indeed, in *Sciara ocellaris,* a temperature-sensitive maternal effect controls X chromosome elimination and determines sex [44].

Cytoplasmic sex determination can be controlled by endosymbionts. *Wolbachia* and *Rickettsia* are related groups of intracellular alpha proteobacteria that can distort the sex ratio of their arthropod hosts [45, 46]. They are transmitted through the egg cytoplasm and alter reproduction in various ways, including cytoplasmic incompatibility, feminization of genetic males, and male killing [46, 47]. Both can induce parthenogenesis, which is of interest since (i) parthenogenetic *Sciara* embryos have been observed [48], and (ii) an entire *Rickettsia* genome was co-assembled with the *Sciara* genome with coverage suggesting an average of two *Rickettsia* genomes per diploid *Sciara* cell in 1–2 day old male embryos. Similarly, symbiont bacteriods have been observed in the cytoplasm of embryos, eggs, and germ cells of a related species, *Sciara ocellaris* (syn. *Bradysia tritici*), with electron and light microscopy [49, 50]. Our data strongly suggest the symbiont bacteriods observed during both oogenesis and embryogenesis in those studies were in the genus *Rickettsia*. Nonetheless, further evidence is needed to ascertain if *Rickettsia* plays a role in *Sciara* sex determination.

### Could DNA modifications play a role in paternal chromosome imprinting in *Sciara*?

Chromosome imprinting for maternal or paternal origin occurs in *Sciara* male meiosis I and in X chromosome elimination in *Sciara* embryos [8], but the mechanism remains elusive. Imprints in mammalian genomes occur in eggs and sperm through a DNA methylation mechanism, leading to differential gene expression at imprinted loci in the offspring [11]. Methylation in mammals typically occurs at CpG sites where it is established de novo by DNA methyltransferase 3 (DNMT3) and maintained by DNMT1 (reviewed in [31]), neither of which are found in Diptera that only have DNMT2 [30, 31, 51]. Our gene annotation set suggests *Sciara* contains DNMT2, but lacks DNMT1 and DNMT3 like other Dipterans. Studies on cytosine methylation in flies have had mixed results. Some found CpG methylation in all insect Orders except flies [51]. Others assert that *Drosophila melanogaster* has DNA methyltransferase activity and CpC methylation [52], low levels of 5-methylcytosine (5mC) [53–55], and more cytosine methylation in stage 5 embryos than oocytes [55]. Immunofluorescence studies identified 5mC in *Sciara* chromosomes [56, 57]. In this study, single-molecule analyses provide additional evidence for the presence of cytosine modifications in the *Sciara* genome, albeit rare. Overall, data in *Sciara* support the existence of low levels of cytosine modifications in flies.

Adenine methylation, particularly 6-methyladenine (6mA), has been reported in the genomic DNA of *Drosophila* and other eukaryotes (reviewed in [30, 38, 39]). DAMT-1 appears to be the methyltransferase for 6mA in insects and DMAD has 6mA demethylating activity in *Drosophila* [58]. Our *Sciara* gene annotation contains both DAMT-1 and DMAD (Supplemental Table 17A). Typically, the level of 6mA in eukaryotes is quite low, such as 0.001–0.07% in early *Drosophila* embryos [58]. Moreover, ApG motifs across diverse eukaryotes have been associated with 6mA, including GAG, CAG, or GAGG. Our single-molecule analyses found 6mA in the *Sciara* genome at similar rates and with similar motifs to other eukaryotes. Moreover, we found that both cytosine and adenine modifications in *Sciara* genomic DNA are phased with 10 bp and 175 bp periodicities, suggesting physical interactions between the 10 bp turns of the DNA helix and methylation machinery as well as a relationship with nucleosome spacing as observed previously [35–39].

Overall, single-molecule sequencing supports the presence of low levels of modified cytosines and adenines in all somatic chromosomes in the male embryo genome of *Sciara*, setting the stage for future studies to elucidate modification differences in females and other developmental stages and tissues, and to determine their biological significance. Base modifications may be a promising avenue for the study of imprinting in *Sciara*.

## Conclusions

We assembled the *Sciara* genome using PacBio, Nanopore, and Illumina sequencing. As no single assembly is likely to be the best assembly, we generated 44 short-read and 50 long-read assemblies. These assemblies were ranked across several dimensions (completeness, gene content, consistency with data) using numerous approaches to find a comparatively-best assembly. BioNano Genomics optical maps were used to scaffold the highest-ranking assemblies. Overall, the *Sciara* genome assembly has excellent contiguity. We annotated this genome, facilitated by RNA-seq datasets from both sexes and multiple life stages. Nearly half of the *Sciara* genome sequence was anchored into chromosomes, and all sequences were classified as X or autosomal. We determined that X-linked genes in *Sciara* males undergo dosage compensation. An entire *Rickettsia* genome was co-assembled with the *Sciara* genome, raising the possibility that it may function in *Sciara’s* unique sex determination mechanism. Finally, the signal level of the PacBio and Oxford Nanopore data revealed the presence of cytosine and adenine modifications in the *Sciara* genome, making feasible their possible role in chromosome imprinting. The assembled, annotated and anchored *Sciara* genome serves as the foundation for future research of the unique features of this emerging model organism. Moreover, these data for *Sciara* greatly expand the genomic information for lower Dipteran flies and will be a valuable resource for phylogenetic studies.

## Methods

### Tissue collection, DNA extraction, DNA sequencing and mapping

Sciara flies (HoLo2) were from the International *Sciara* Stock Center at Brown University (https://www.brown.edu/research/facilities/sciara-stock/). Crosses between straight-winged (XX) females and males (XO) were used to obtain strictly male progeny. Where relevant, embryos were aged 2 h – 2 days. Genomic DNA (gDNA) was isolated using DNAzol (ThermoFisher), cleaned with AMPure beads (Beckman Coulter), and analyzed for purity and concentration with NanoDrop and Qubit (ThermoFisher). For 100 bp paired-end reads from Illumina HiSeq 2000, male embryo gDNA was sonicated to 100–600 bp, prepared using the NEBNext kit (New England Biolabs; NEB), run on a 2% NuSieve agarose (Lonza) gel, size-selected for 500 bp, gel purified (Qiagen), and sequenced. Pacific Biosciences RSII Single Molecule Real Time sequencing datasets (P5-C3 chemistry; 2 libraries; 24 SMRT cells) were obtained by the Technology Development Group (Institute of Genomics and Multiscale Biology, Mount Sinai Icahn School of Medicine). Nanopore data was collected using various kits (SQK-MAP002, MAP004, MAP005, MAP006), pores (R7.3 and R7.3 70 bps 6mer), and MinION devices (original, MkI) across 15 libraries from male *Sciara* embryo gDNA and 2 from male adult gDNA, prepared with modifications to the manufacturer’s instructions to increase read lengths [59] (Suppl. Methods), base-called with Oxford Nanopore Technologies’ Metrichor 2d basecaller (versions 1.10.2, 10.13.1, 1.14.4, 1.19.0, and 1.20.0), and analyzed using our own custom set of tools: Fast5Tools [60]. For BioNano Genomics (BNG) Irys optical maps, flash frozen male pupae were ground in liquid nitrogen, high molecular weight gDNA was isolated, nicked with BssSI (CACGAG, NEB), labeled, and repaired according to the IrysPrep protocol (BNG). For detailed information on all data collection, see Supplemental Materials Section 3.

#### Microscopy

Photos of early embryos were taken with a Zeiss Lumar V12 fluorescence stereomicroscope equipped with NA objective (ApoLumar S1.2X) and AxioCam MRm camera. Images were taken using Zeiss AxioVision 4.8.2 software. The contrast of the TIFF images was adjusted with Adobe Photoshop. Photos of adult flies, larva, and pupa were taken with a Zeiss Stemi SV11 stereomicroscope equipped with NA objective (S 1.0X) and Canon EOS 5D camera (attached to the binocular the tube via Gosky T2 camera mount; 23.2 mm eyepiece port). The contrast of raw images was adjusted with Adobe Photoshop. The size standard was photographed separately in the same condition and the image was merged with Photoshop. No filters are used for any photos. Standard light (halogen gooseneck lamp) was used for both microscopes.

### Genome assemblies

Multiple short-read assemblies (numbers shown in parentheses) were created for each of 7 assemblers: ABySS (8) [61], Megahit (4) [62], Platanus (8; 4 prior to contamination removal, 4 after) [63], SGA (2) [64], SOAP (8) [65], SPAdes (6) [66], and Velvet (8) [67]. Assemblies from the same assembler differed by the parameters used and/or how the data was pre-processed. Illumina data was either provided “raw” or after trimming/filtering with Trimmomatic [68] and/or error-correction with BayesHammer [69]. Similarly, multiple assemblies were generated for each long-read assembler, differing by parameters and input data (PacBio-only or PacBio-and-Nanopore reads, with or without quality filtering). Hybrid assemblies were generated with short-read contigs from Platanus [63] and long reads using DBG2OLC (5) [70] and PBDagCon [71]. Non-hybrid long-read assemblies were generated with ABruijn (3) [72], Canu (18) [73], Falcon (12) [74], Miniasm (8) [75] with RaCon [76], and SMARTdenovo (5) [77]. Long-read assemblies were polished with Quiver [78] and Pilon [79]. BlobTools [80] was used to identify contaminating contigs. For detailed information on data processing, assemblers, parameters used, and contamination filtering for the 44 short-read assemblies see Supplemental Materials Sections 4.1.1–4.1.2 and 4.1.4, and for the 50 long-read assemblies, see Supplemental Materials Sections 4.2.1–4.2.4 and 4.2.7.2.

### Assembly evaluations

Assembly evaluations included contig size statistics (NG50, LG50, maximum length, expected contig size [81]), percent of Illumina reads mapped using Bowtie2 [82], the conditional probability of the reads given each assembly using LAP [83], the Bayesian probability that each assembly is correct given the reads with ALE [84], number of features from FRC^bam^ [85], percent error-free bases and the mean base score from REAPR [86], completeness of gene content with BUSCO [87], percent of long reads that aligned with BWA [88], average number of split alignments per long read, structural variations using Sniffles [89], percent of raw BioNano map alignments using Maligner [90], resulting optical map alignment M-scores, the number of bases covered by optical maps (span), and total coverage from aligned optical maps. The final set of genome assembly evaluations included metrics from RNA-seq and de novo transcriptome alignments, as well as associated evaluations of reference-guided transcriptome assemblies and Maker2 gene annotations. Evaluations were automated and parallelized on SLURM with a custom package: Battery [91]. For detailed information, see Supplemental Materials Section 4.1.3 for the 7 short read assembly evaluations, Section 4.2.4.2 for metrics used in monitoring long-read assembly polishing steps, Section 4.2.5 for the 27 long-read assembly evaluations, Section 4.2.6.4 for scaffold evaluations, Sections 4.3.2 and 4.3.4.15 for evaluations of genome-guided transcriptome assemblies and genome annotations that were used in the final selection of Bcop_v1, and Section 4.3.5 for the final set of 66 evaluations.

### Scaffolding

Optical maps > 150 kb were assembled into consensus maps (CMAPs) using BioNano Pipeline Version 2884 and RefAligner Version 2816 (BNG). Genome-wide hybrid scaffolds were created using hybridScaffold.pl version 4741 (BioNano Genomics). Quiver and PBJelly [92] were used to polish and gap-fill the scaffolds. PBJelly was used to further scaffold with long-reads. Quiver and Pilon were used for final polishing. For more detail, see Supplemental Materials Sections 4.2.6 and 4.2.7.

### Assembly anchoring

Haplotigs were identified using Minimap2 [93] and purge haplotigs [94]. Sequences that were previously mapped to chromosomes experimentally (Table 2) were mapped to the assemblies using BLAST [95]. Differentiating between autosomal and X-linked contigs was performed by requiring haploid coverage levels across at least 80% of a contig to be called as X-linked, using Minimap2 and BEDTools [96]. For more details on anchoring by known sequences and coverage, see Supplemental Materials Section 4.2.7.4 and 4.2.7.5, respectively.

### Transcriptome assemblies

Crosses were designed to yield only male (XX x XO) or only female (X’X x XO) progeny. Poly-A+ RNA was prepared separately for each sex and stage using TRIzol (Invitrogen/ThermoFisher), DNase (Qiagen), RNeasy columns (Qiagen), and Oligo-dT DynaBeads (Life Technologies). RNA integrity was assessed on 1.1% formaldehyde 1.2% agarose gels. Purity and quantity were measured by NanoDrop and Qubit. Strand-specific RNA sequencing libraries were prepared using NEB’s Magnesium Fragmentation Module, SSIII (Invitrogen) first strand synthesis with random primers, NEBNext Second Strand Synthesis module with ACGU nucleotide mix (10 mM each of dATP, dCTP, dGTP, and 20 mM of dUTP), NEBNext End Repair and dA-Tailing (NEB), ligation (NEB: NEBNext Quick Ligation Reaction Buffer, NEB Adaptor, Quick T4 Ligase), and size-selected with AMPure beads (Beckman Coulter). Uracil-cutting for strand-specificity (and hairpin adapter cutting) was done with NEBNext USER enzyme, followed by PCR using NEBNext High-Fidelity 2X PCR Master Mix and NEBNext indexed and universal primers for 12 cycles. PCR products were size-selected with AMPure beads. Purity, quantity, and size of the libraries were checked with NanoDrop, Qubit and Fragment Analyzer (Agilent). The mean estimated fragment sizes was ~ 420 bp (mean insert sizes ~ 300 bp). Libraries were sequenced by Illumina HiSeq 2000 for 100 bp paired-end reads. RNA-seq datasets were combined and assembled with Trinity [97] or HiSat2 [98] and StringTie [99]. Transcriptome assemblies were evaluated with BUSCO [87], RSEM-Eval [100], and TransRate [101]. For more details, see Supplemental Materials Sections 3.7, 4.3.1, and 4.3.2 .

### Repeat and gene annotation

Species-specific repeat libraries were built using RepeatModeler [102] and were combined with previously known repeat sequences from *Bradysia coprophila* and all Arthropod repeats in the RepeatMasker Combined Database: Dfam_Consensus-20,181,026 [103], RepBase-20,181,026 [104]. To predict protein-coding genes, Maker2 [105] was used with (i) transcriptome assemblies for expression evidence, (ii) transcript and protein sequences from related species for homology evidence, (iii) Augustus [106], SNAP [107], and GeneMark-ES [108] as gene prediction engines, and (iv) RepeatMasker [24] to mask repeats. InterProScan [109] was used to identify Pfam domains and GO terms from predicted protein sequences, and BLASTp was used to find best matches to curated proteins in the UniProtKB/Swiss-Prot database [110]. Maker2 transcriptomes were evaluated using annotation edit distances, BUSCO [87], RSEM-Eval [100], and TransRate [101]. For more details, see Supplemental Materials Sections 4.3.3 and 4.3.4.

### DNA modification analyses

For detailed information on all DNA modifications analyses, see Supplemental Materials Section 4.4. For the PacBio analyses specifically, see Supplemental Materials Section 4.4.1. Briefly, DNA modifications were detected based on polymerase kinetics in PacBio data [111, 112]. PBalign [113] with BLASR v2 [114] was used to align PacBio reads to the entire unfiltered assembly to avoid forcing incorrect mappings. Pbh5tools [115] was used to merge and sort the mapped reads. ipdSummary from kineticsTools v0.6.0 [116] was used to predict base modifications across the Canu genome assembly (−-pvalue 0.01 --minCoverage 3 --methylMinCov 10 --identifyMinCov 5). AgIn [33] was also used for CpG methylation. Only primary contigs labeled as Arthopoda were used for these analyses. Kmer enrichment scores for dimers and trimers were obtained from the Chi-square standardized residuals found when comparing the distribution of kmers that had a specific modification at a fixed position with the genome-wide distribution of kmers with the target base at that position. This approach also defined enriched 7-mers for position weight matrix motifs using WebLogo [117]. The 9 bp sequences centered on the top 500 or 5000 scoring specific modification calls were used with MEME [118] to identify motifs using a second order Markov model background file trained on the *Sciara* genome assembly (fasta-get-markov -m 2 -dna). We determined if DNA modifications were enriched/depleted in various genomic regions using binomial models. Salmon [119] was used to quantify expression of annotated genes using male embryo RNA-seq. BEDtools was used to obtain spacing distances between modified bases as well as between random bases of the same type (e.g. m6A vs random A). Periodicities in inter-modification distances between 0 and 200 bp were determined by discrete Fourier transform (DFT) analysis using the Fast Fourier Transform (FFT) from Python’s Numpy package.

For detailed information on the Nanopore analysis, see Supplemental Materials Section 4.4.2. Briefly, datasets generated from the MinION MkI, SQK-MAP006 kit, and R7.3 70 bps 6mer pore model were the only ones supported by Nanopolish [34], and the subset of 2D reads therein was used in this analysis. Reads were mapped to the entire unfiltered assembly to avoid forcing incorrect mappings. Only 2D reads that aligned to primary contigs annotated as Arthropoda were used. The signal distributions for each kmer in native *Sciara* gDNA was compared to the expected kmer models, and to a Nanopore dataset generated from whole genome PCR on *E. coli* genomic DNA using the same kit and pore model (BioProject PRJEB13021; Run ERR1309547; www.ebi.ac.uk/ena; [34]). Nanopore reads were aligned with BWA [88]. Nanopolish [34] was used to learn updated kmer models from the native *Sciara* and *E. coli* PCR datasets. MEME was used to identify short motifs in all 6mers that differed from the expected ONT model.

### Further bioinformatics

Supplemental Materials contains all details to reproduce these analyses. Bioinformatics analyses were aided by custom scripts: Battery, Fast5Tools, fftDNAMods, Lave, Sciara Project Tools [60, 91, 120–122].

## Supplementary Information

**Additional file 1 **: **Supplemental Figure S1:** Comparing evaluations of short read assemblies to long read assemblies. **Supplemental Figure S2:** Assembly ranking correlation matrices. **Supplemental Figure S3:** Filtering out non-Arthropod, contaminating reads using Taxonomy- annotated GC plots. **Supplemental Figure S4:** Length Distributions for Illumina Scaffolds, PacBio Reads and MinION Molecules. **Supplemental Figure S5:** Percent identity of MinION reads compared to a PacBio-only assembly. **Supplemental Figure S6:** Evaluations across Quiver polishing rounds. **Supplemental Figure S7:** Blended assemblies with both PacBio and MinION data tended to receive better ranks than PacBio-alone assemblies. **Supplemental Figure S8:** Metrics comparing assemblies after scaffolding. **Supplemental Figure S9:** Aligning chosen and discarded scaffolds from each assembler (Canu and Falcon). **Supplemental Figure S10:** BlobTools analysis of the Canu scaffolds. **Supplemental Figure S11:** BlobTools analysis and anchoring Falcon scaffolds. **Supplemental Figure S12:** The single locus that contains the full-length Escribá insert. **Supplemental Figure S13:** Pairwise comparisons of final Canu and Falcon scaffolds. **Supplemental Figure S14:** Pairwise comparisons of final Canu and Falcon annotations. **Supplemental Figure S15:** Dosage compensation of X-linked genes in *Sciara coprophila*. **Supplemental Figure S16:** Distribution of DNA modifications across *Sciara* genome (PacBio analysis). **Supplemental Figure S17:** Position weighted 7-mer motifs learned from different filtering and different subsets of the genome sequence (PacBio analysis). **Supplemental Figure S18:** MEME motifs in the PacBio and MinION analyses. **Supplemental Figure S19:** MinION signal distributions for 6mers defined by motifs learned in the PacBio analysis and negative controls. **Supplemental Figure S20:** The GCG trimer is depleted in the genome and transcriptome compared to expectation. **Supplemental Figure S21:** Distribution of distances between adjacent DNA modifications (PacBio analysis) on the same strand shows enrichment of short distances, a 10 bp periodicity, and a spike of enrichment at mono-nucleosome lengths of ~ 175 bp. **Supplemental Figure S22:** Distribution of distances between adjacent DNA modifications (PacBio analysis) on either strand also shows enrichment of short distances, a 10 bp periodicity, and a spike of enrichment at mono-nucleosome lengths of ~ 175 bp for 6mA and 5mC. **Supplemental Table S1 A-E:** Expected genome and chromosome sizes. **Supplemental Table S2:** RNA-seq samples spanning both sexes and 4 life cycle stages. **Supplemental Table S3:** Short read assembly size statistics. **Supplemental Table S4:** Long read assembly size statistics. **Supplemental Table S5:** Pairwise comparisons of size statistics of hybrid scaffolds from pair of Canu or Falcon assemblies. **Supplemental Table S6:** Size statistics of Canu C3.2 across the work flow. **Supplemental Table S7:** Size statistics of Falcon F9 across the work flow. **Supplementary Table 8 A-C:** Gap size statistics. **Supplemental Table S9:** Bacterial contig statistics in each assembly. **Supplemental Table S10:** *Sciara (Bradysia) coprophila* repeat family classes from RepeatModeler. **Supplemental Table S11:** Sub-classification of classified Repeat Families found in *Sciara coprophila* genome with RepeatModeler. **Supplemental Table S12 A-B:** Repeat Masking on Canu. **Supplemental Table S13 A-B:** Repeat Masking on Falcon. **Supplemental Table S14:** Transcriptome Evaluations. **Supplemental Table S15 A-B:** Maker Annotation Transcript Evaluations on Canu and Falcon. **Supplemental Table S16:** Additional characterization and comparisons of the final annotations of Canu and Falcon assemblies. **Supplemental Table S17 A-C:** Putative Sciara homologs for proteins involved in reading, writing, and erasing DNA methylation marks for adenine and cytosine. **Supplemental Table S18 A-F:** DNA modification percentages in male embryonic genomic DNA. **Supplemental Table S19:** Which dimers are observed with modifications more often than expected?. **Supplemental Table S20:** Which trimers are observed with modifications more often than expected?. **Supplemental Table S21 A-F:** Binomial tests for enrichment or depletion of DNA modifications in various genomic features. The Supplement also contains detailed experimental and bioinformatic methods sections, as well as software versions and supplemental references.

## Data Availability

Raw Illumina, PacBio, Nanopore, and BioNano data as well as BioNano CMAPs and PacBio kinetics and DNA modification results have been submitted to the NCBI BioProject database [123] under accession number PRJNA291918. Raw sequence files can be found directly in SRA under study accession SRP218121. This Whole Genome Shotgun project has been deposited at DDBJ/ENA/GenBank under the project accession VSDI00000000. This version of the project (01) has the accession number VSDI01 (nuccore VSDI00000000.1), and consists of sequences VSDI01000001-VSDI01000743. The current assembly name is BU_Bcop_v1, and has GenBank and RefSeq assembly accessions GCA_014529535.1 and GCF_014529535.1. Two sets of annotations can be found: (1) NCBI *Bradysia coprophila* Annotation Release 100 (https://www.ncbi.nlm.nih.gov/genome/annotation_euk/Bradysia_coprophila/100/), and (2) The Bcop_v1.0 Maker2 annotation for the Canu assembly is hosted by the USDA Ag Data Commons (10.15482/USDA.ADC/1522618) and is available at the i5k Workspace [26]. The bacterial contigs separated from Bcop_v1 that feature the Rickettsia endosymbiont of *Bradysia coprophila*, Holo2-sym-1, have been deposited as their own WGS project in DDBJ/ENA/GenBank under the accession JAHXDM000000000 (version JAHXDM010000000), and are associated with the same BioProject (PRJNA291918; also see PRJNA748098, and BioSample SAMN20326103). The de novo transcriptome used to facilitate gene annotation (BU_Bcop_GenTrans_v1) was deposited as a Transcriptome Shotgun Assembly at DDBJ/EMBL/GenBank under the accession GJHU00000000 (version GJHU01000000), and is associated with BioProject PRJNA291918.

## Abbreviations

1D read: One direction read (template or complement only)
2D read: Two direction read (consensus from template and complement)
4mC: 4-methyl-Cytosine
5mC: 5-methyl-Cytosine
6mA: 6-methyl-Adenine
ALE: Assembly Likelihood Evaluation
Bcop_v1: *Bradysia coprophila* genome and annotation version 1
BioNano: BioNano Genomics (BNG) Irys for optical mapping
BLAST: Basic Local Alignment Search Tool
BUSCO: Benchmark Universal Single Copy Orthologs
CMAPs: Consensus maps from multiple alignments of raw optical maps
CRL: Comprehensive Repeat Library
FRC: Feature Response Curve
gDNA: Genomic DNA
GP: Gag-pol
HSMs: Hybrid Scaffold Maps from CMAPs and genome assembly maps
L50/ LG50: Ordering contigs from longest to shortest, the number of the longest contigs needed to reach or exceed 50% of the assembly size (L50) or expected genome size (LG50)
LAP: Log Average Probability
LINEs: Long interspersed nuclear transposable elements
LTR: Long terminal repeat
Nanopore: Oxford Nanopore Technologies MinION
N50 / NG50: The length of the shortest contig (or scaffold or read) such that 50% of the assembly or dataset size (N50) or 50% of the expected genome length (NG50) is on sequences of that length or longer
ONT: Oxford Nanopore Technologies
PacBio: Pacific Biosciences
RC: Rolling Circle family of transposons
REAPR: Recognition of Errors in Assemblies using Paired Reads
RT: Reverse Transcriptase
RTE: Retro-Transposable Element, non-LTR type
ScRTE: RTE-like repeat found in Sciara (Bradysia) coprophila by Escriba et al.
SINEs: Short interspersed nuclear transposable elements
SMRT: Single-Molecule Real-Time (from PacBio)
UTRs: Untranslated regions (of mRNA)
