# Supplementary material for: High contiguity de novo genome assembly and DNA modification analyses for the fungus fly, Sciara coprophila, using single-molecule sequencing
Source: BMC Genomics. 2021 Sep 6;22:643. doi: 10.1186/s12864-021-07926-2 (PMC8419958; doi:10.1186/s12864-021-07926-2)
Supplement: Supplementary file 1 — Additional file 1 : Supplemental Figure S1: Comparing evaluations of short read assemblies to long read assemblies. Supplemental Figure S2: Assembly ranking correlation matrices. Supplemental Figure S3: Filtering out non-Arthropod, contaminating reads using Taxonomy- annotated GC plots. Supplemental Figure S4: Length Distributions for Illumina Scaffolds, PacBio Reads and MinION Molecules. Supplemental Figure S5: Percent identity of MinION reads compared to a PacBio-only assembly. Supplemental Figure S6: Evaluations across Quiver polishing rounds. Supplemental Figure S7: Blended assemblies with both PacBio and MinION data tended to receive better ranks than PacBio-alone assemblies. Supplemental Figure S8: Metrics comparing assemblies after scaffolding. Supplemental Figure S9: Aligning chosen and discarded scaffolds from each assembler (Canu and Falcon). Supplemental Figure S10: BlobTools analysis of the Canu scaffolds. Supplemental Figure S11: BlobTools analysis and anchoring Falcon scaffolds. Supplemental Figure S12: The single locus that contains the full-length Escribá insert. Supplemental Figure S13: Pairwise comparisons of final Canu and Falcon scaffolds. Supplemental Figure S14: Pairwise comparisons of final Canu and Falcon annotations. Supplemental Figure S15: Dosage compensation of X-linked genes in Sciara coprophila. Supplemental Figure S16: Distribution of DNA modifications across Sciara genome (PacBio analysis). Supplemental Figure S17: Position weighted 7-mer motifs learned from different filtering and different subsets of the genome sequence (PacBio analysis). Supplemental Figure S18: MEME motifs in the PacBio and MinION analyses. Supplemental Figure S19: MinION signal distributions for 6mers defined by motifs learned in the PacBio analysis and negative controls. Supplemental Figure S20: The GCG trimer is depleted in the genome and transcriptome compared to expectation. Supplemental Figure S21: Distribution of distances between adjacent DNA modifications [file 12864_2021_7926_MOESM1_ESM.pdf]

Supplemental Materials

Single-molecule sequencing of long DNA molecules allows high contiguity
de novo genome assembly for the fungus fly, *Sciara coprophila*

Table of Contents:
Pages 2-12

Glossary:
Pages 13-14

Section 1: Supplemental Figures
Pages 15-39

Supplemental Figure S1:
Comparing evaluations of short read assemblies to long read assemblies
Page 15

Supplemental Figure S2:
Assembly ranking correlation matrices
Page 16

Supplemental Figure S3:
Filtering out non-Arthropod, contaminating reads using Taxonomy- annotated GC plots
Page 17

Supplemental Figure S4:
Length Distributions for Illumina Scaffolds, PacBio Reads and MinION Molecules
Page 18

Supplemental Figure S5:
Percent identity of MinION reads compared to a PacBio-only assembly
Page 19

Supplemental Figure S6:
Evaluations across Quiver polishing rounds
Page 20

Supplemental Figure S7:
Blended assemblies with both PacBio and MinION data tended to receive better ranks than PacBio-alone assemblies
Page 21

Supplemental Figure S8:
Metrics comparing assemblies after scaffolding
Page 22

Supplemental Figure S9:
Aligning chosen and discarded scaffolds from each assembler (Canu and Falcon)
Page 23

Supplemental Figure S10:
BlobTools analysis of the Canu scaffolds
Page 24

Supplemental Figure S11:
BlobTools analysis and anchoring Falcon scaffolds
Page 25

Supplemental Figure S12:
The single locus that contains the full-length Escribá insert
Page 26

Supplemental Figure S13:
Pairwise comparisons of final Canu and Falcon scaffolds
Page 27

Supplemental Figure S14:
Pairwise comparisons of final Canu and Falcon annotations
Page 28-29

Supplemental Figure S15:
Dosage compensation of X-linked genes in *Sciara coprophila*
Page 30-31

Supplemental Figure S16:
Distribution of DNA modifications across *Sciara* genome (PacBio analysis)
Page 32

Supplemental Figure S17:
Position weighted 7-mer motifs learned from different filtering and different subsets of the genome sequence (PacBio analysis)
Page 33

Supplemental Figure S18:
MEME motifs in the PacBio and MinION analyses
Page 34

Supplemental Figure S19:
MinION signal distributions for 6mers defined by motifs learned in the PacBio analysis and negative controls
Page 35

Supplemental Figure S20:
The GCG trimer is depleted in the genome and transcriptome compared to expectation
Page 36

Supplemental Figure S21:
Distribution of distances between adjacent DNA modifications (PacBio analysis) on the same strand shows enrichment of short distances, a 10 bp periodicity, and a spike of enrichment at mono-nucleosome lengths of ~175 bp
Page 37,39

Supplemental Figure S22:
Distribution of distances between adjacent DNA modifications (PacBio analysis) on either strand also shows enrichment of short distances, a 10 bp periodicity, and a spike of enrichment at mono-nucleosome lengths of ~175 bp for 6mA and 5mC
Page 38-39

Section 2: Supplemental Tables
Pages 40-74

Supplemental Table S1 A-E:
Expected genome and chromosome sizes
Page 40-41

Supplemental Table S2:
RNA-seq samples spanning both sexes and 4 life cycle stages
Page 42

Supplemental Table S3:
Short read assembly size statistics
Page 43

Supplemental Table S4:
Long read assembly size statistics
Page 44

Supplemental Table S5:
Pairwise comparisons of size statistics of hybrid scaffolds from pair of Canu or Falcon assemblies
Page 45

Supplemental Table S6:
Size statistics of Canu C3.2 across the work flow
Page 46

Supplemental Table S7:
Size statistics of Falcon F9 across the work flow
Page 47

Supplementary Table 8 A-C:
Gap size statistics
Page 48

Supplemental Table S9:
Bacterial contig statistics in each assembly
Page 49

Supplemental Table S10:
Sciara (*Bradysia*) *coprophila* repeat family classes from RepeatModeler
Page 50

Supplemental Table S11:
Sub-classification of classified Repeat Families found in *Sciara coprophila* genome with RepeatModeler
Page 51-52

Supplemental Table S12 A-B:
Repeat Masking on Canu
Page 53

Supplemental Table S13 A-B:
Repeat Masking on Falcon
Page 54

Supplemental Table S14:
Transcriptome Evaluations
Page 55

Supplemental Table S15 A-B:
Maker Annotation Transcript Evaluations on Canu and Falcon
Page 56-58

Supplemental Table S16:
Additional characterization and comparisons of the final annotations of Canu and Falcon assemblies
Page 59

Supplemental Table S17 A-C:
Putative Sciara homologs for proteins involved in reading, writing, and erasing DNA methylation marks for adenine and cytosine
Page 60-64

Supplemental Table S18 A-F:
DNA modification percentages in male embryonic genomic DNA
Page 65-67

Supplemental Table S19:
Which dimers are observed with modifications more often than expected?
Page 68

Supplemental Table S20:
Which trimers are observed with modifications more often than expected?
Page 69

Supplemental Table S21 A-F:
Binomial tests for enrichment or depletion of DNA modifications in various genomic features
Page 70-74

Section 3: Detailed experimental methods
Pages 75-80

3.1 Embryo Collection
Page 75

3.2 DNA extraction
Page 75

3.3 Illumina PE Genomic DNA library
Page 75

3.4 PacBio Sequencing Details
Page 75-76

3.5 MinION sequencing details
Page 76-79

3.6 BioNano Irys optical mapping details
Page 79

3.7 Strand-specific RNA-seq details
Page 79-80

Section 4: Detailed Bioinformatic Methods
Pages 81-170

4.1 Illumina assemblies
Page 81-96

4.1.1 Inputs
Page 81-83

4.1.1.1 Trimming and quality filtering
4.1.1.2 Short read error-correction
4.1.1.3 Naming of short read datasets used in assemblies
4.1.1.4 Insert size statistics

4.1.2 Assembling the short reads
Page 84-88

4.1.2.1 Abyss
4.1.2.2 Megahit
4.1.2.3 Platanus
4.1.2.4 SGA
4.1.2.5 SOAPdenovo2
4.1.2.6 SPAdes
4.1.2.7 Velvet

4.1.3 Evaluations of the Initial 40 Short Read Assemblies
Page 89-91

4.1.3.1 Size statistics
4.1.3.2 Mapping the reads back to each assembly
4.1.3.3 Percent mapped
4.1.3.4 ALE
4.1.3.5 FRC^{bam}
4.1.3.6 LAP
4.1.3.7 REAPR
4.1.3.8 BUSCOv1

4.1.4 Contamination analyses for the selected Platanus short read assembly
Page 92-96

4.1.4.1 Obtaining taxonomy IDs from BLAST hits for each contig.
4.1.4.2 Using BlobTools and custom scripts to label each contig with a taxonomy
4.1.4.3 Obtaining contamination-filtered reads for re-assembly
4.1.4.4 Platanus contamination-filtered re-assemblies
4.1.4.5 Evaluations of contamination-filtered re-assemblies

4.2 Long Read Assemblies using PacBio and Oxford Nanopore Data
Page 97-129

4.2.1 Long read inputs to assemblers
Page 98-102

4.2.1.1 Long read set naming
4.2.1.2 Obtaining fastx files from PacBio .bax.h5 files using bash5tools
4.2.1.3 Obtaining fastx files from MinION fast5 files using fast5tools
4.2.1.4 A more detailed description of Fast5Tools and MinION read analyses
4.2.1.5 MarginStats for percent identity of MinION Reads

4.2.2 Hybrid-assembling long reads with short reads
Page 103

4.2.2.1 DBG2OLC with Platanus short read contigs and PBDAGCON

4.2.3 Assembling the long reads alone
Page 104-111

4.2.3.1 ABruijn
4.2.3.2 Canu
4.2.3.3 Falcon
4.2.3.4 Miniasm and RaCon
4.2.3.5 SMARTdenovo

4.2.4 Polishing the long-read assemblies
Page 112-113

4.2.4.1 Iterative polishing with signal-level PacBio data using Quiver
4.2.4.2 Evaluations during Quiver Polishing
4.2.4.3 Iterative polishing with Illumina short reads using Pilon

4.2.5 Evaluations of the Initial 50 long-read assemblies
Page 114-118

4.2.5.1 Size statistics
4.2.5.2 BUSCO completeness metrics
4.2.5.3 Illumina metrics
4.2.5.4 PacBio and MinION long read metrics
4.2.5.5 BioNano optical map metrics

4.2.5.6 Selection for BioNano scaffolding
4.2.5.7 Automating the battery of metrics

4.2.6 BioNano scaffolding
Page 119

4.2.6.1 BioNano CMAP assembly
4.2.6.2 BioNano hybrid scaffolding
4.2.6.3 Obtaining gap intervals for gap sizing
4.2.6.4 Scaffold comparisons and evaluation

4.2.7 Assembly refining: Gap-filling, polishing, contamination removal, haplotig identification, and anchoring
Page 120-129

4.2.7.1 Final polishing, gap-filling, and meta-scaffolding
4.2.7.2 Identifying contaminating contigs and scaffolds
4.2.7.3 Identifying primary and associated sequences
4.2.7.4 Anchoring sequences into chromosomes using known, localized, and/or homologous sequences
4.2.7.5 Classifying/anchoring assembled sequences as autosomal or X based on diploid vs haploid coverage levels

4.3 Transcripts, genes, repeats, and a final genome assembly choice
Page 130-154

4.3.1 Transcriptome Assemblies
Page 130-131

4.3.1.1 De novo Transcriptome assembly using Trinity
4.3.1.2 Genome-guided transcriptome assembly using Stringtie

4.3.2 Evaluations of transcriptome assemblies
Page 132-133

4.3.2.1 BUSCO
4.3.2.2 RSEM-Eval
4.3.2.3 TransRate

4.3.3 Building a comprehensive repeat library for use in Maker2
Page 134-135

4.3.3.1 *De novo* repeat libraries with RepeatModeler
4.3.3.2 Known *Sciara* repeats
4.3.3.3 Known Arthropod Repeats

4.3.4 Maker2 gene annotations
Page 136-151

4.3.4.1 Overview
4.3.4.2 Training GeneMark-ES for the first gene prediction round of Maker
4.3.4.3 Using BUSCO to train Augustus for the first gene prediction round of Maker
4.3.4.4 Using Maker est2genome/protein2genome to train SNAP for the first gene prediction round of Maker
4.3.4.5 Repeat Libraries
4.3.4.6 EST evidence
4.3.4.7 Alternative EST evidence
4.3.4.8 Protein homology evidence
4.3.4.9 Other parameter choices
4.3.4.10 Maker2 Round 1
4.3.4.11 Maker2 Round 2
4.3.4.12 Maker2 Round 3 (keep_preds=1)
4.3.4.13 Maker2 Round 3 with gene-filtered repeat library (keep_preds=1)
4.3.4.14 Maker2 Round 3 combined standard gene sets and finalization
4.3.4.15 Maker2 gene set evaluations

4.3.5 Final assembly selection
Page 152-155

4.3.6 Dosage compensation analysis
Page 156

4.3.7 Characterization of the Lambda Phage Insert Containing ScRTE
Page 157

4.4 DNA modifications
Page 158-170

4.4.1 PacBio Analysis
Page 159-166

4.4.1.1 PacBio Modification Prediction
4.4.1.2 DNA sequence motifs found near sites of predicted DNA modifications
4.4.1.3 Kmers enriched for DNA modifications
4.4.1.4 MEME motifs in the highest scoring sites
4.4.1.5 Enrichment/Depletion of DNA modifications in various genomic regions
4.4.1.6 Binomial tests for higher or lower modification rates in genomic regions
4.4.1.7 Binomial tests for enrichment/depletion of DNA modifications in genomic regions
4.4.1.8 Spacing between DNA modifications

4.4.2 Orthogonal support for PacBio modification results using the MinION data
Page 167-168

4.4.3 Calculating observed:expected ratios of dimers and trimers
Page 169-170

Section 5: Software versions and Supplemental References
Pages 171-180

5.1 Software versions used
Page 171-175

5.2 Supplemental References
Page 176-180

Glossary and Acronyms:

**ALE**	Assembly Likelihood Evaluation. A probabilistic measure of assembly accuracy given read quality, mate pair orientation, insert length, coverage, alignments, and k-mer frequencies. See Clark et al 2013.
**Anchoring**	Anchoring is the process of mapping contigs/scaffolds into chromosomes, typically into specific loci. For flies like Drosophila and Sciara, this is done using data from in situ hybridization experiments on polytene chromosomes.
**BUSCO**	Benchmark Universal Single Copy Orthologs. Sets of genes expected to be present given evolutionarily related species. See Simao et al 2015.
**CMAP**	BioNano **C**onsensus **MAP**s produced by "assembling" raw BioNano optical maps together to produce a consensus optical map representation of the genome.See Lam et al 2012, and other BioNano literature.
**Contig**	Contiguous sequence put together by aligning reads together. The ends of contigs represent areas of uncertainty where multiple choices were present, commonly caused by repeats.
**Coverage**	This is the sum of read lengths divided by the genome size – i.e. the average number of times any given base in the genome is read. Thus, 10X coverage means that the sum of read lengths was 10 times the genome length, and each base is represented 10 times on average in the dataset.
**EGS**	Expected Genome Size – the expected size or length of a haploid (single copy) complement of a genome. The EGS for Sciara was obtained with DNA content measurements by Rasch.
**FRC / FRC^{bam}**	Feature Response Curve. FRC^{bam} is a program that evaluates assemblies using read-layouts of aligned reads. We used the number of features it flags as a proxy for the number of assembly errors. See Vezzi et al 2012.
**Haplotigs**	Diploid genomes can have loci that are represented twice (or more) in an assembly. Each contig representation of the same locus is a haplotig. Common practice is to use only the version on the longest contig as part of the primary assembly. See Roach et al 2018 for more information.
**LAP**	Log Average Probability. LAP is a probabilistic measure of assembly quality given a set of reads. The more an assembly resembles the genome sequence, the higher the score.
**L50 / LG50**	Ordering contigs from longest to shortest, the number of the longest contigs needed to reach or exceed 50% of the assembly size (L50) or expected genome size (LG50). LG50 is used to directly compare different assemblies.
**LINEs**	Long interspersed nuclear elements, a family of transposons.
**LTR**	Long Terminal Repeat – LTR transposons are a family of transposons.
**N50 / NG50**	The length of the shortest contig (or scaffold or read) such that 50% of the assembly or dataset size (N50) or 50% of the expected genome length (NG50) is on sequences of that length or longer. NG50 is used to directly compare different assemblies.
**RC**	Rolling Circle family of transposons.
**SINEs**	Short interspersed nuclear elements, a family of transposons.
**Scaffolds**	A set of contigs ordered and oriented with respect to each other with unknown sequence in between them (gaps). Gaps are usually represented as a stretch of Ns given the estimated gap length.
**Sniffles**	A program to detect structural variants (SVs) with long reads. See Sedlazeck et al 2018.

**Span**	The length of sequence in an assembly covered at least once by reads (or optical maps). Thus, if optical map alignments span 50 Mb of an assembly, then 50 Mb had at least 1 optical map over it.
**SV**	Structural variant – a variation between two genomes involving the structure of the genome rather than a point mutation. Examples are short and long deletions, copy number variations, duplications, insertions, and translocations. Mis-assemblies will increase the number of apparent SVs. So, assemblies that look more like the true underlying genome structure will have fewer SVs than assemblies with many errors.

Section 1: Supplemental Figures

Supplemental Figure S1: Comparing evaluations of short read assemblies to long read assemblies.

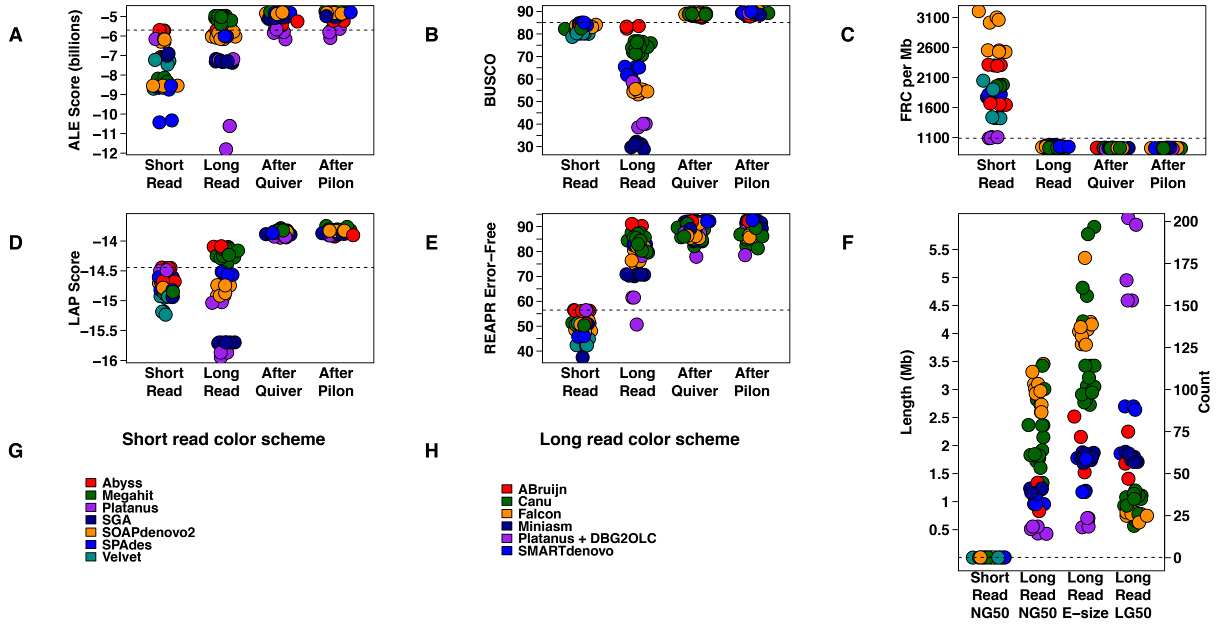

Supplemental Figure S1: Comparing evaluations of short read assemblies to long read assemblies (prior to any scaffolding).
(A) ALE scores. **(B)** BUSCO: percent complete Arthropod BUSCOs. **(C)** Average number of features per Mb detected by FRC^{bam}. **(D)** LAP scores**. (E)** REAPR percent error-free bases. **(F)** Size statistics. NG50 = size of contig such that at least 50% of the expected genome size is on contigs of that size or larger. Expected contig size is as defined in the Genome Assembly Gold-standard Evaluations (GAGE) paper (Salzberg et al. 2012), which is designed to give the expected size of contig containing a randomly selected base in the assembly. The expected genome size was used in the denominator of the equation instead of individual assembly sizes for direct comparisons as done in the GAGE paper. LG50 is the number of contigs (count) it takes to reach at least 50% of the expected genome size when selecting contigs from longest to shortest. Lower LG50s are better. **(G-H)** Legends for A-F. “Short read” indicates assemblies generated using Illumina data. “Long read” indicates assemblies generated using long read data before any polishing. “After Quiver” and “After Pilon” indicate the long read assembly evaluations taken after each of those polishing steps. In (F), the size statistics for long read assemblies are from those after Pilon polishing.

Supplemental Figure S2: Assembly ranking correlation matrices

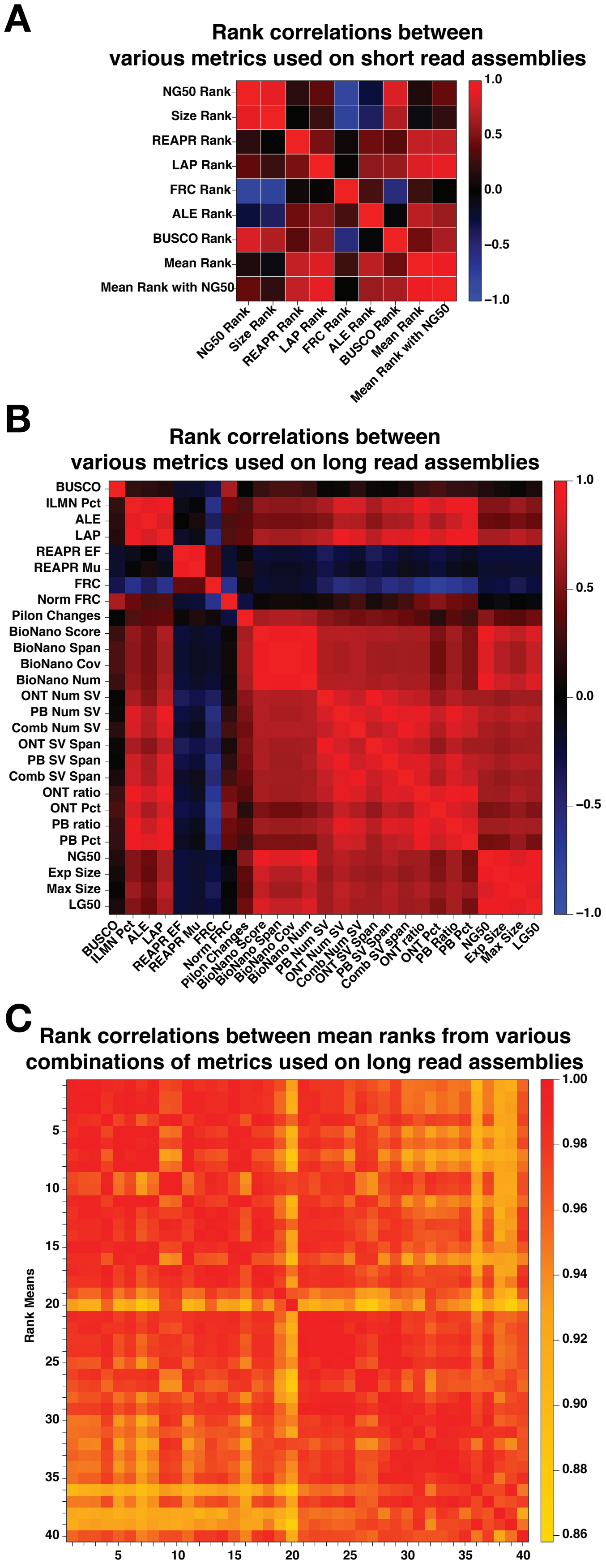

Supplemental Figure S2: Assembly ranking correlation matrices

(A) Matrix of pairwise correlations between short read assembly rankings for given metrics. Also see Figure 2A.

(B) Matrix of pairwise correlations between long read assembly rankings for given metrics. Also see Figure 2E.

(C) Matrix of pairwise correlations between long read assembly rankings for mean ranks from different combinations of metrics. Also see Figure 2E.

Note: These matrices represent both Pearson and Spearman correlation coefficients since the correlations were computed on ranks.

Supplemental Figure S3: Filtering out non-Arthropod, contaminating reads using Taxonomy- annotated GC plots.

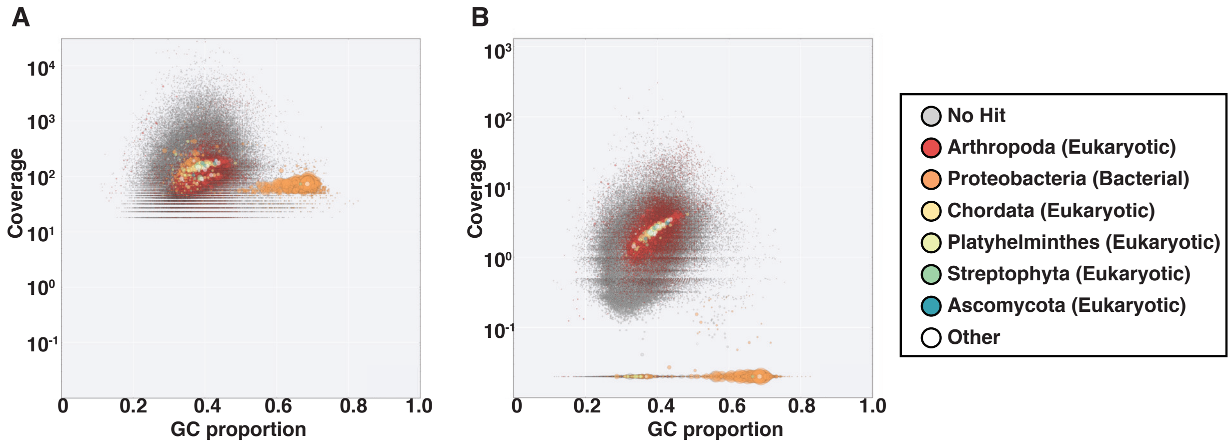

Supplemental Figure S3: Filtering out non-Arthropod, contaminating reads using Taxonomy- annotated GC plots.

Taxonomy-Annotated GC (TAGC) plots can be used to visualize the GC proportion and coverage for each contig in an assembly. In the TAGC plots, each circle represents a contig. The size of each circle is proportional to contig size. The colors correspond to phylum-level taxonomy assignments. Notice that the largest contigs in this Platanus assembly are all bacterial (i.e. the largest circles are orange). Contaminating genomes, from the microbiome on and in embryos for example, are expected to be at different copy numbers and have different GC contents than the target organism. Therefore, when contaminating genomes are present, more than one cluster of contigs is typically seen, allowing coverage and GC proportion cutoffs to be chosen for filtering unannotated contigs.

(A) TAGC plot of the chosen Platanus assembly with kmer coverage from the reads input into the assembly on the y-axis and GC proportion of contigs on the x-axis. The bacterial cluster has similar coverage and though it has a higher average GC content, the clusters overlap in that dimension as well.

(B) TAGC plot with read coverage from pre-amplification stage salivary glands on the y-axis and GC proportion of contigs on x-axis. In this case, using a different sample from a different tissue and prepared by a different person, resulted in differential coverage between the *Sciara* genome and the bacterial cluster.

Supplemental Figure S4: Length Distributions for Illumina Scaffolds, PacBio Reads and MinION Molecules.

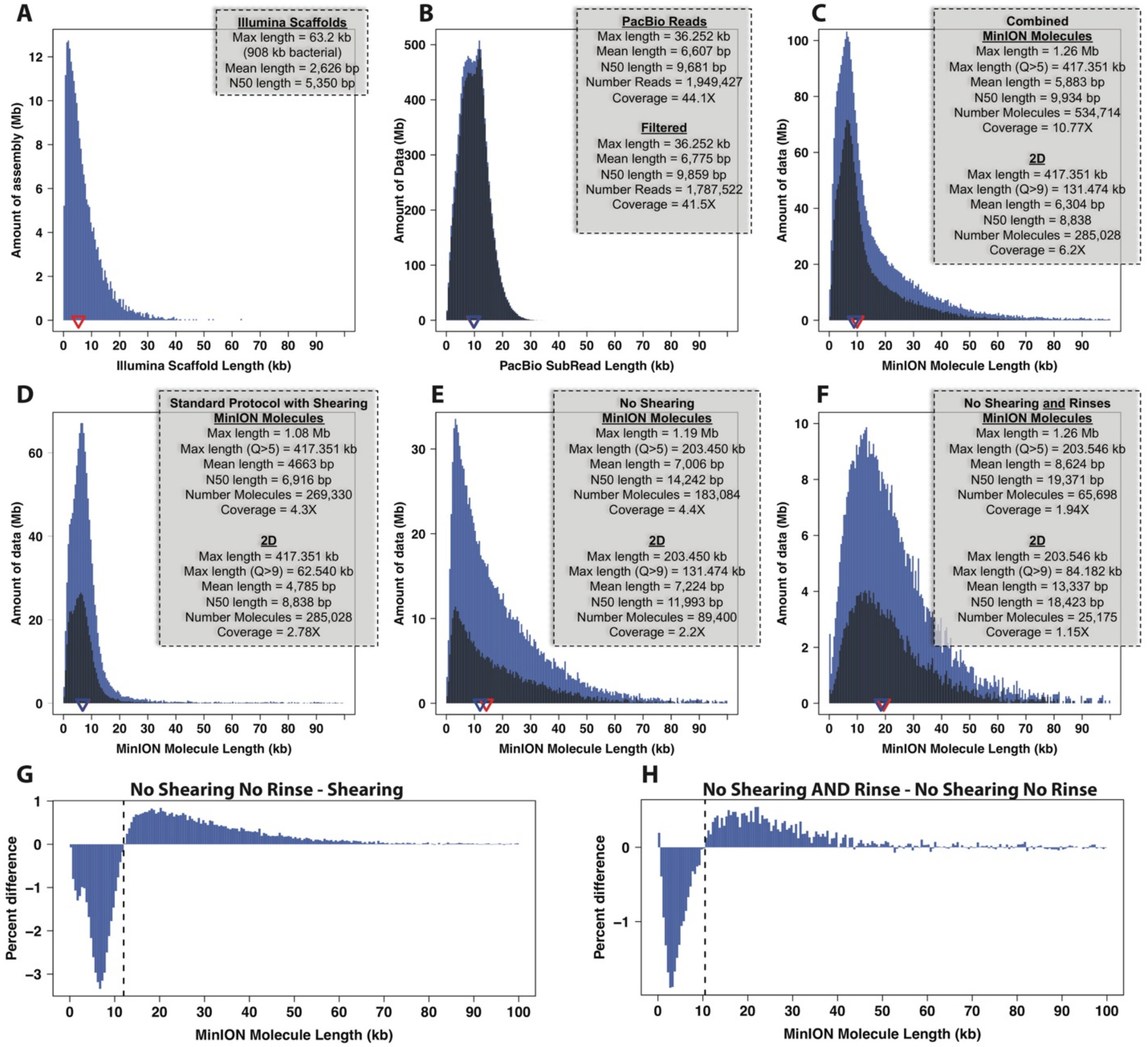

Supplemental Figure S4: Length Distributions for Illumina Scaffolds, PacBio Reads and MinION Molecules.
(A) Platanus scaffold lengths from Illumina data. **(B)** Sub-read length distribution for all PacBio subreads (blue) or filtered (shaded area). **(C-F)** MinION molecule length (blue) and 2D read only (shaded) distributions from **(C)** combining all libraries, **(D)** only libraries that followed the standard ONT protocol with shearing, targeting 8 kb, **(E)** libraries that skipped shearing and used other long read principles, but did not include rinse steps, and **(F)** libraries that skipped shearing AND included rinse steps. **(G)** Libraries that skipped shearing are depleted for molecules around 10-12 kb and enriched for longer molecules with respect to the libraries from the standard protocol. **(H)** Libraries that skipped shearing AND included rinse steps are additionally depleted for molecules <10-12 kb and additionally enriched for longer molecules, as demonstrated by comparing to the libraries that only included shearing, but no rinse. We described the rinse steps during AMPure cleanups elsewhere (Urban et al. 2015) and in the supplemental methods. Note that a single “MinION molecule length” is defined for each sequenced molecule (that can produce up to 3 reads) as described in the supplemental methods and elsewhere (Urban et al. 2015).

Supplemental Figure S5: Percent identity of MinION reads compared to a PacBio-only assembly.

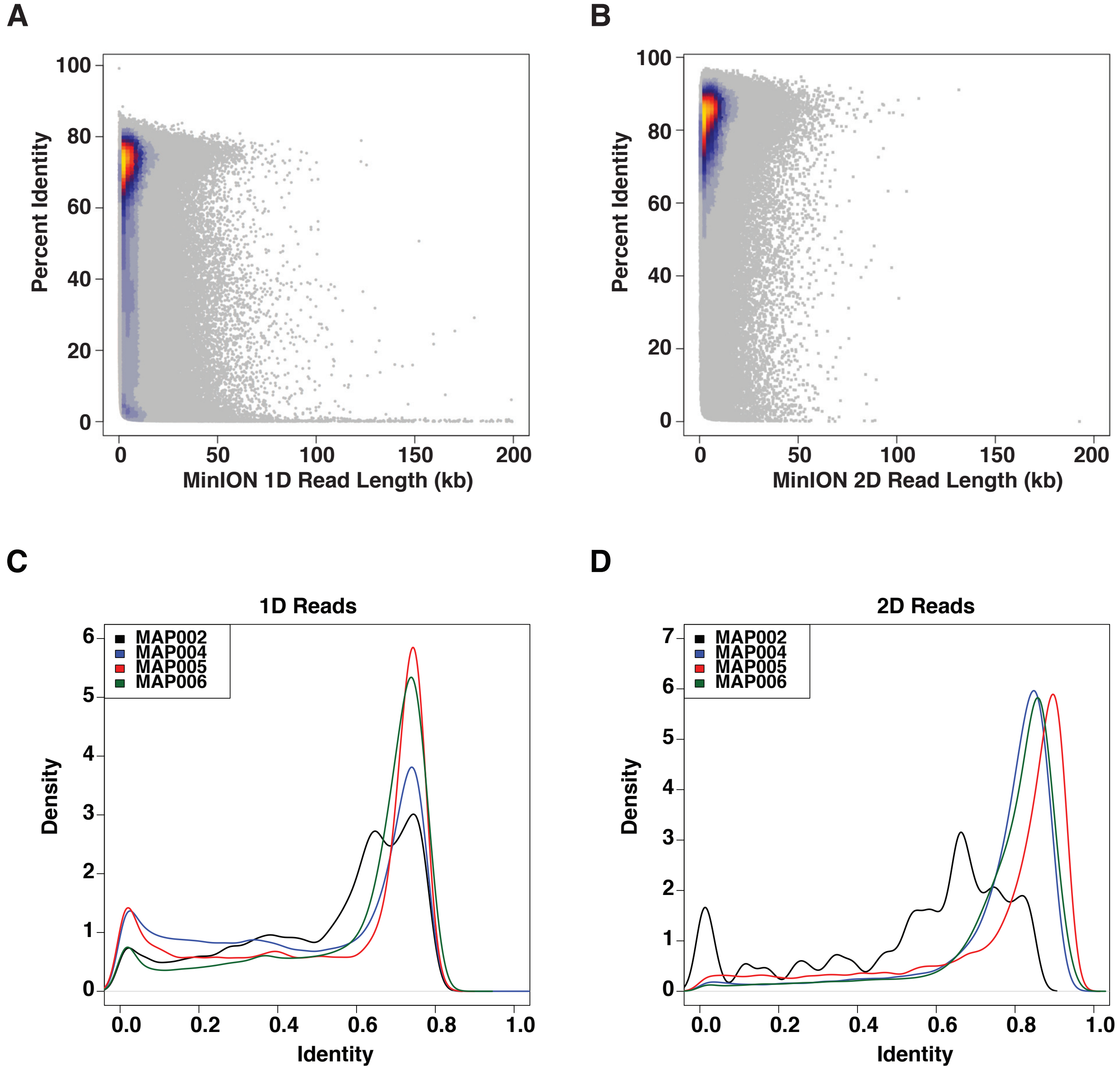

Supplemental Figure S5: Percent identity of MinION reads compared to a PacBio-only assembly.
(A) 1D read length vs percent identity. **(B)** 2D read length vs percent identity. **(C-D)** Percent identity distributions separated by kit version for **(C)** 1D reads and **(D)** 2D reads. In both (A) and (B), the scatter plot has heat colors representing where the least (grey) and most (yellow) data is located. The limits on the x-axes was set to 200 kb to better visualize the data shorter than that length. The several reads longer than 200 kb appear to be quite low quality. In all, the percent identity is the result of summing the identities in the section of the read that aligned and dividing by the length of the read. Since we did not perform a re-alignment step (Jain et al. 2015), low quality reads with only small sections mapped appear to have a much lower percent identity than is likely the case.

Supplemental Figure S6: Evaluations across Quiver polishing rounds.

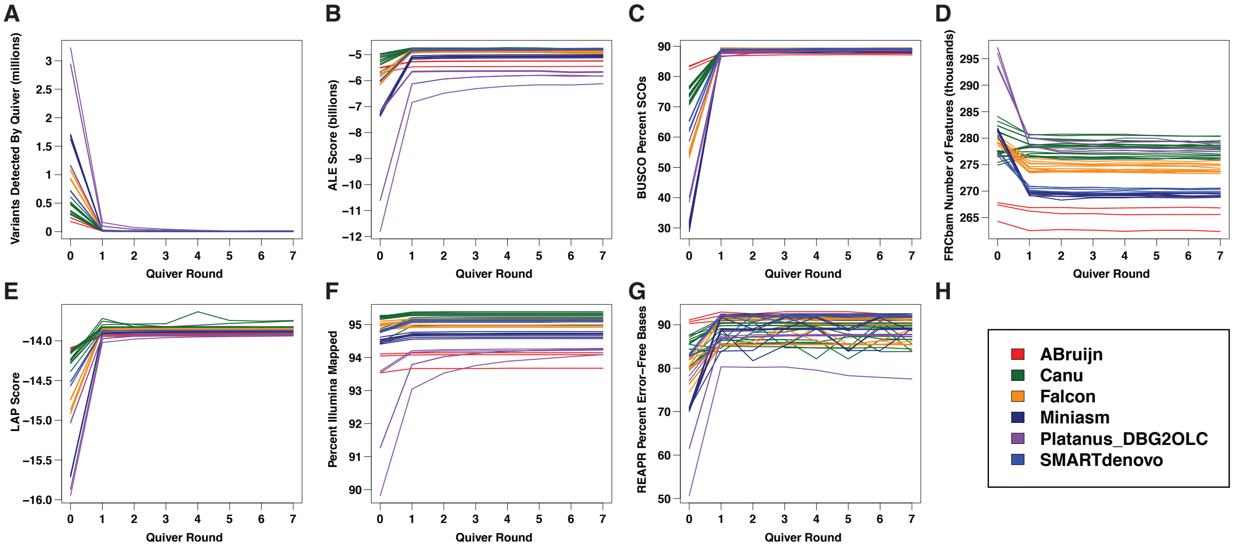

Supplemental Figure S6: Evaluations across Quiver polishing rounds.
(A) Number of variants detected by Quiver. **(B)** ALE scores. **(C)** BUSCO (Benchmark Universal Single Copy Ortholog): percent complete Single Copy Orthologs (SCOs). **(D)** Number of features detected by FRC^{bam}. **(E)** LAP scores. **(F)** Percent of Illumina dataset that maps to the assembly. **(G)** REAPR percent error-free bases. **(H)** The legend for all plots. Note that “round 0” represents the scores of the assemblies being input into the first Quiver round. The largest improvement in all metrics is seen after the first round (at Quiver Round = 1).

Supplemental Figure S7: Blended assemblies with both PacBio and MinION data tended to receive better ranks than PacBio-alone assemblies.

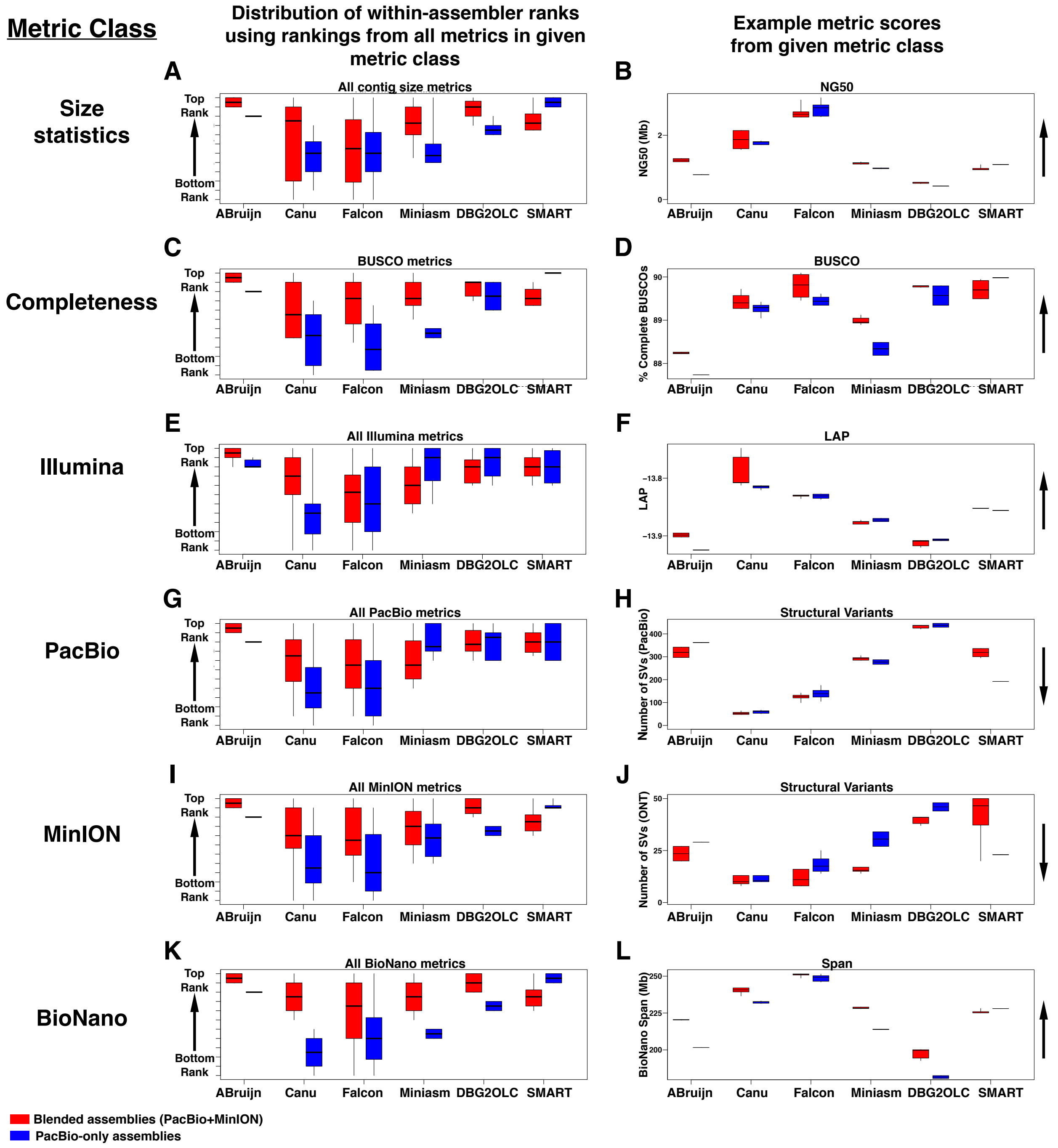

Supplemental Figure S7: Blended assemblies with both PacBio and MinION data tended to receive better ranks than PacBio-alone assemblies.

Box and whisker plots where the boxes show the 25th-75th percentile, the black line is the median, and the whiskers span the range (min to max). **(A, C, E, G, I, K)** Assemblies from a given assembler were ranked using all individual metrics from given metric class (to the left of plots). The ranks from individual metrics were combined and partitioned into those from blended versus PacBio-only assemblies. Better ranks are higher. **(B, D, F, H, J, L)** The actual scores (not ranks) from an example metric from the given metric class is plotted. The arrows to the right of plots show the direction that the given score gets better.

Supplemental Figure S8: Metrics comparing assemblies after scaffolding

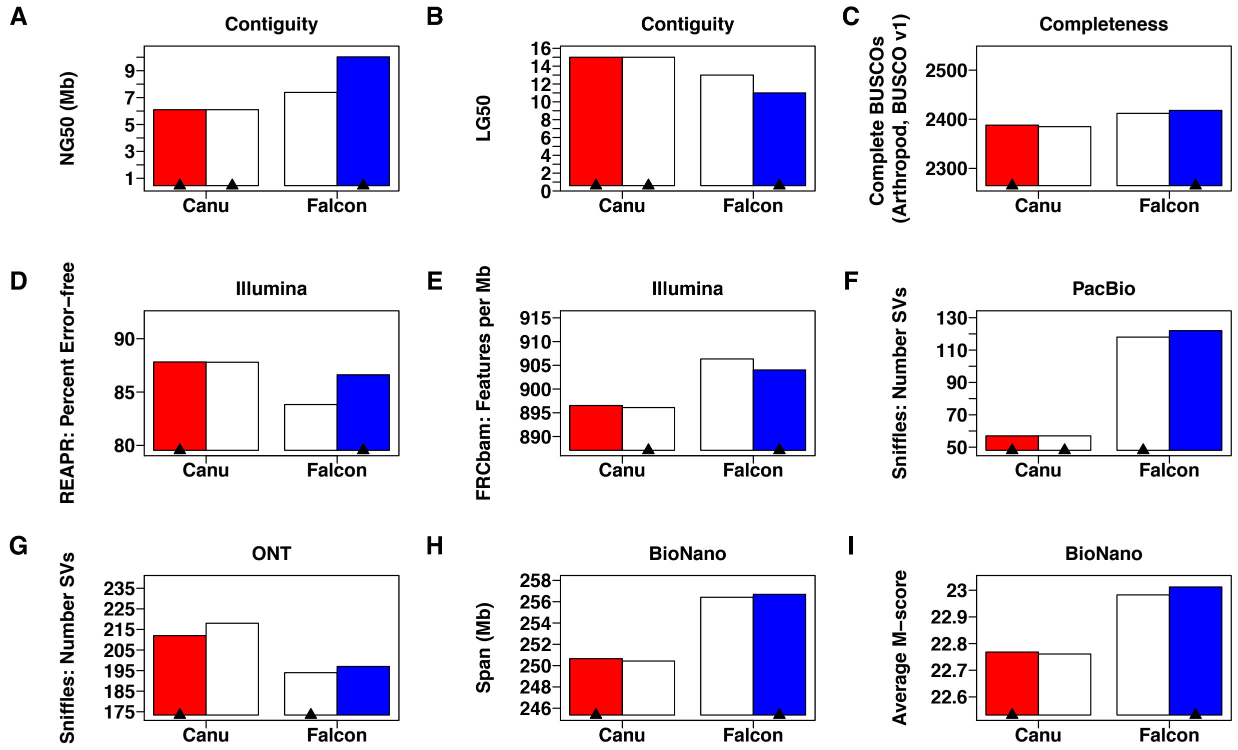

Supplemental Figure S8: Metrics comparing assemblies after scaffolding
We chose two Canu and two Falcon assemblies for BioNano scaffolding. However, we found them to be extremely similar, so used only the one version of each that tended to do slightly better in these metrics. Also see Supplemental Figure S9 and Supplemental Table S5. In each panel, the red bar is the chosen Canu assembly and the blue bar is the chosen Falcon assembly. The white bars are the assemblies that we did not continue working on. For each assembler, the black arrowheads indicate which assembly in the pair did better in that metric (both in a pair have arrowheads in the case of a tie). **(A-B)** Show contiguity metrics: NG50 and LG50. Higher NG50s and lower LG50s are considered better. **(C)** Shows BUSCO as a completeness metric where higher numbers are better. **(D-E)** Show Illumina-based metrics from REAPR and FRC^{bam}, where higher and lower numbers, respectively, are better. **(F-G)** Show long read metrics for PacBio and MinION (ONT) respectively. Both show the number of structural variants (SVs) as detected by Sniffles given those datasets. Lower numbers are better. **(H-I)** Show BioNano optical map metrics. Span is the number of bases in the assembly covered by at least one optical map (higher is better). M-score is a score based on the alignments from Maligner that is typically negative, but is multiplied by -1 here for positive values such that higher is better.

Supplemental Figure S9: Aligning chosen and discarded scaffolds from each assembler (Canu and Falcon)

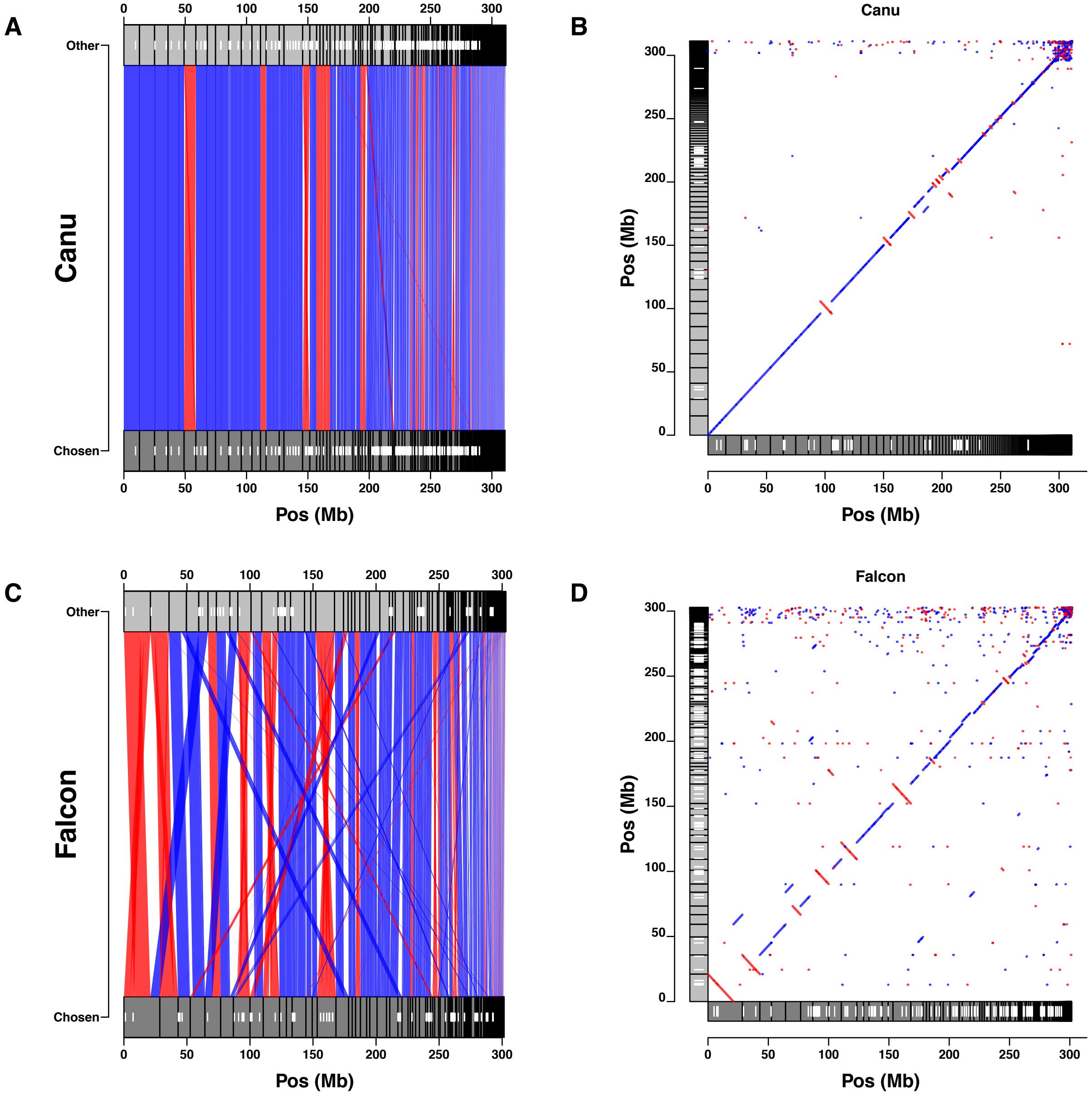

Supplemental Figure S9: Aligning chosen and discarded scaffolds from each assembler (Canu and Falcon)
We took two Canu and two Falcon assemblies into BioNano scaffolding. For a given assembler we aligned the two assembly versions with Minimap2 and visualized the alignments as pairwise plots (A, C) and dot plots (B, D) using our own tools made in R (github.com/JohnUrban/lave). In both types of plots, blue represents that the positive strand from both sequences aligned whereas red indicates that the alignment is in the reverse direction (negative strand with respect to each other). The polygons at the top and bottom of the pairwise plots or the x- and y- axes of the dot plots represent the scaffolds, and the white smaller polygons within them represent gap locations. In both, the chosen assembly is the bottom set of polygons (i.e. x-axis in dot plots). **(A-D)** Pairwise alignment plot and dot plot for the two sets of scaffolds derived from Canu assemblies (A-B) or Falcon assemblies (C-D). For more on analyzing the two versions of scaffolds from each assembler, see Supplemental Figure S8 and Supplemental Table S5.

In the figure labels, "Pos" = Position.

Supplemental Figure S10: BlobTools analysis of the Canu scaffolds

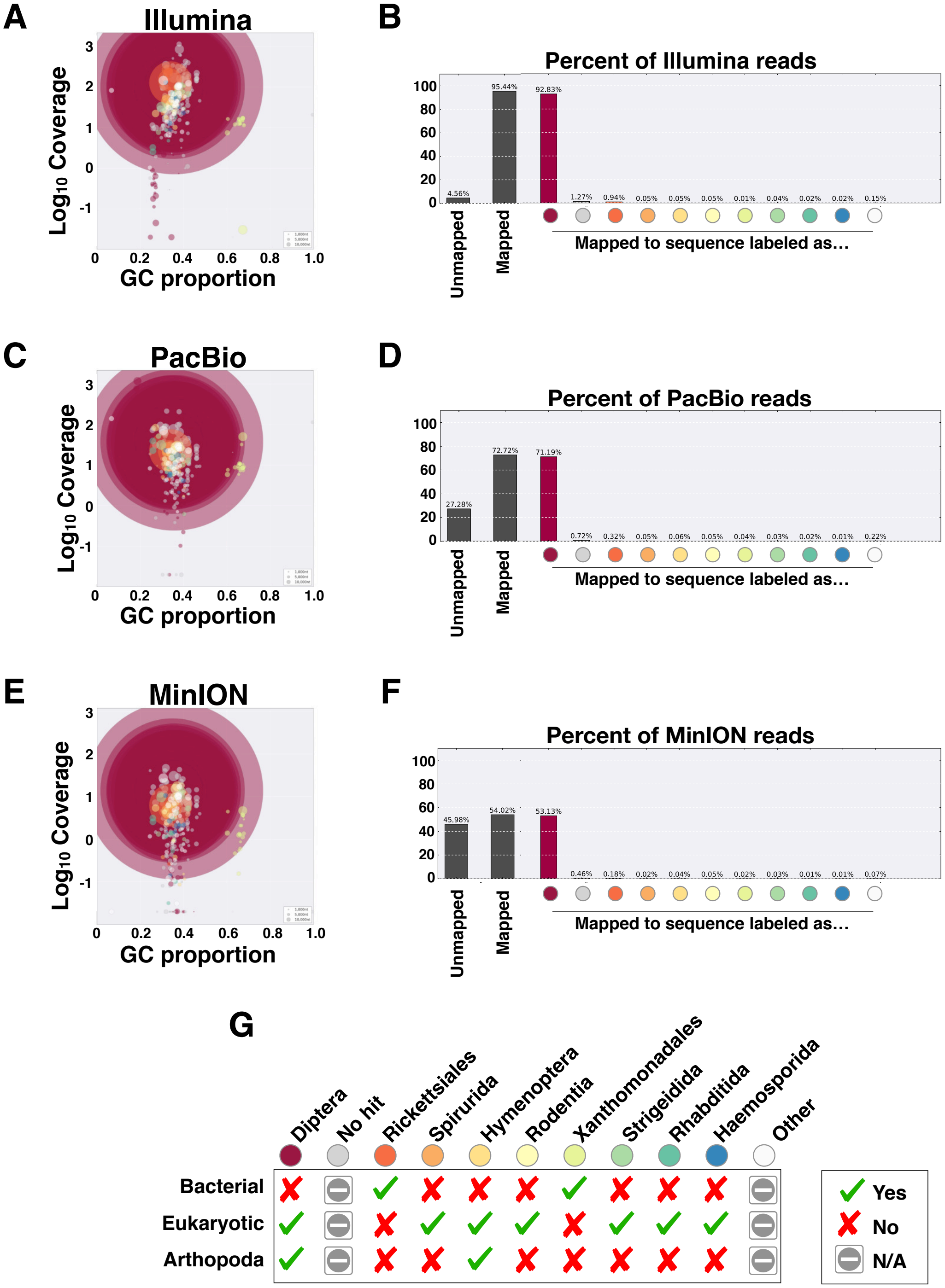

Supplemental Figure S10: BlobTools analysis of the Canu scaffolds

Note that essentially identical results were obtained for the Falcon assembly. **(A, C, E)** These plots show contigs/scaffolds as circles sized relative to the contig size, colored according to taxon information, and positioned according to GC content and read coverage from **(A)** Illumina, **(B)** PacBio, or **(C)** MinION. The coloring scheme is as detailed in (G). The largest contigs (and majority of assembled sequence) are labeled as Dipteran (Arthropoda). **(B, D, F)** Percent of reads that mapped (or did not) to any contig and percent that mapped to contigs labeled as the given taxonomy for the **(B)** Illumina, **(D)** PacBio, and **(F)** MinION datasets. Color scheme of circles on x-axis is as in (G). **(G)** Circle color legend for A-F with some additional taxon information.

Supplemental Figure S11: BlobTools analysis and anchoring Falcon scaffolds

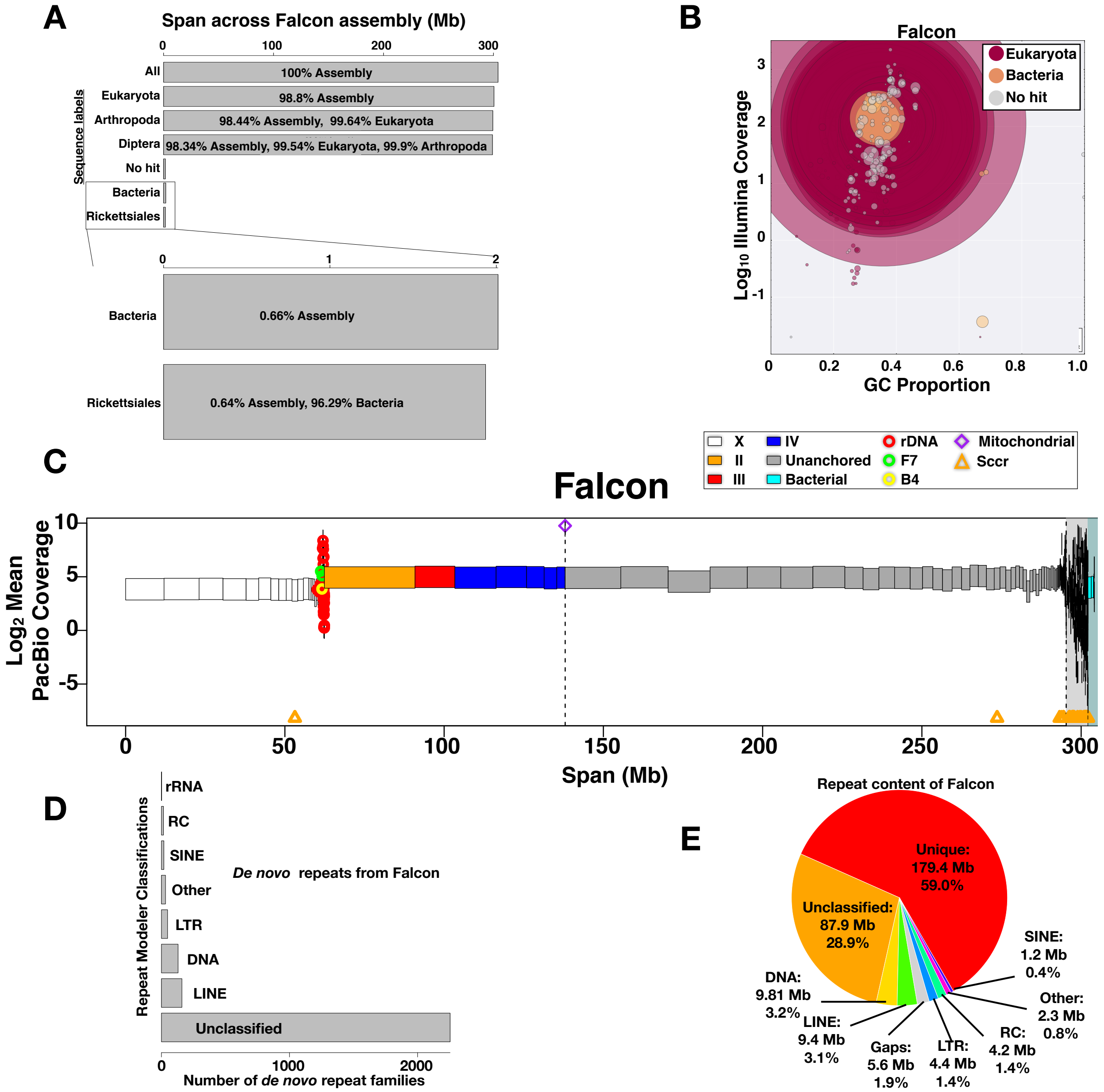

Supplemental Figure S11: BlobTools analysis and anchoring Falcon scaffolds
(A) The proportions of the Falcon assembly taxonomically labeled as Eukaryotic, Arthropoda, Diptera, Bacteria, and Rickettsiales.
(B) Log10 Illumina coverage versus GC content over the Falcon assembly.
(C) The Falcon assembly with scaffolds drawn as rectangles corresponding to their lengths, colored according to the chromosome they were anchored into (or unanchored), and positioned at their mean coverage from PacBio reads, the dataset used to determine X-linked sequences by haploid level coverage. The white background highlights sequences in the primary assembly whereas the grey and cyan backgrounds are set behind associated and bacterial sequences, respectively. All sequences to the left of the first vertical dashed line are anchored.
(D) Repeat modeler family classifications when run on Falcon assembly.
(E) Repeat content of the Falcon assembly from repeat masking. Note that the DNA, LINE, LTR, RC, and SINE are also classes of transposon elements (“DNA” stands for DNA elements).

Supplemental Figure S12: The single locus that contains the full-length Escribá insert

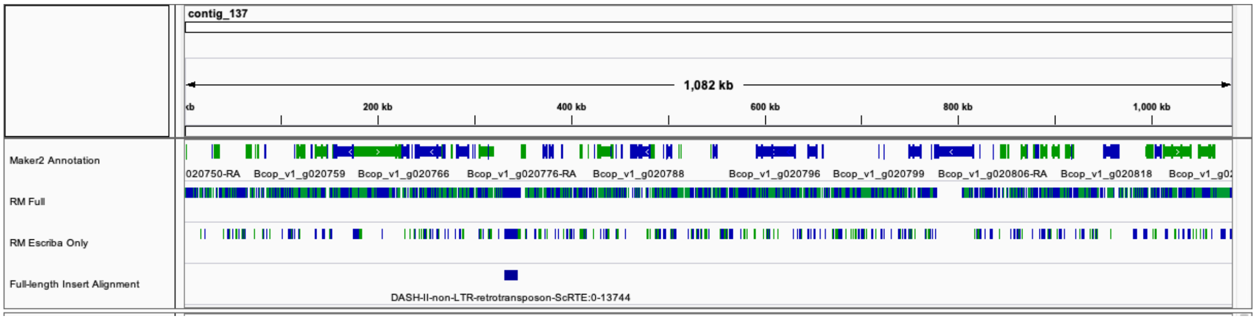

Supplemental Figure S12: The single locus that contains the full-length Escribá insert
This figure shows the ~1.1 Mb contig (contig_137) that harbors the full-length 13.8 kb insert sequence from Escribá et al (2011). In all rows, green shows positive strand alignments and blue shows negative strand alignments. The top row shows the Maker2 annotated regions. “RM Full” shows all regions marked as repeats by repeat masker when using the full comprehensive repeat library. “RM Escribá only” shows regions marked as repeats by repeat masker when using only the 13.8 kb Escribá insert sequence (Escribá et al. 2011). “Full-length Insert Alignment” shows the precise site that the insert corresponds to on this contig. The site is intergenic.

Supplemental Figure S13: Pairwise comparisons of final Canu and Falcon scaffolds

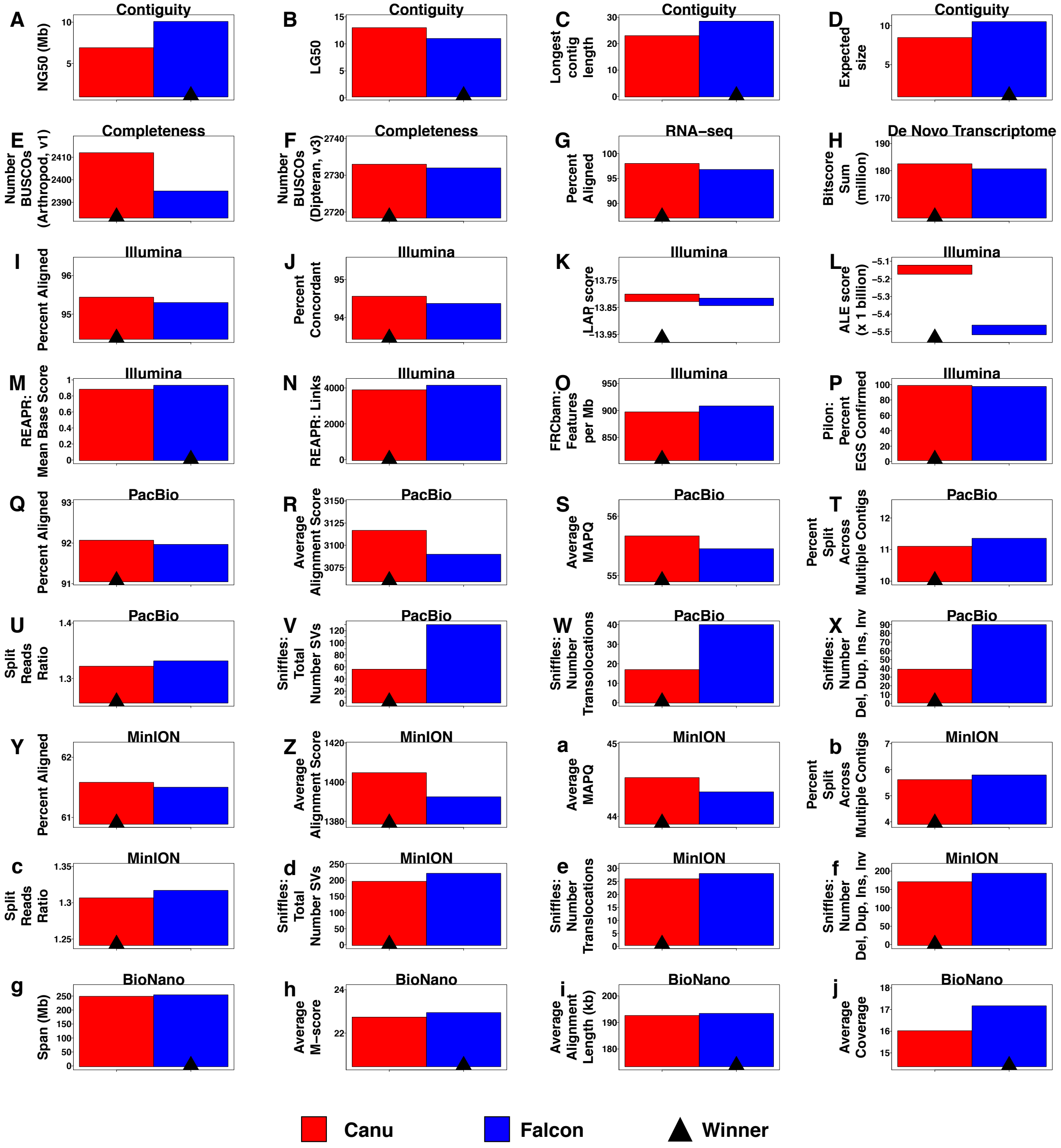

Supplemental Figure S13: Pairwise comparisons of final Canu and Falcon scaffolds. This figure shows actual scores, summarized in Figure 5F, for each of the evaluations of the assemblies for contiguity, completeness, and genome sequencing metrics. Evaluations of the transcriptomes and annotations guided by these assemblies are reported in Supplemental Tables S14, S15, and S16. Overall, the Canu scaffolds and annotation consistently received better scores.

Note that the black arrowhead shows the winner in each category, and that in some categories higher scores are better, and other categories lower scores are better. For example, a better assembly will have fewer putative mis-assembled regions as proxied by Sniffles structural variants, split long read alignments, and FRC^{bam} features.

Supplemental Figure S14: Pairwise comparisons of final Canu and Falcon annotations.

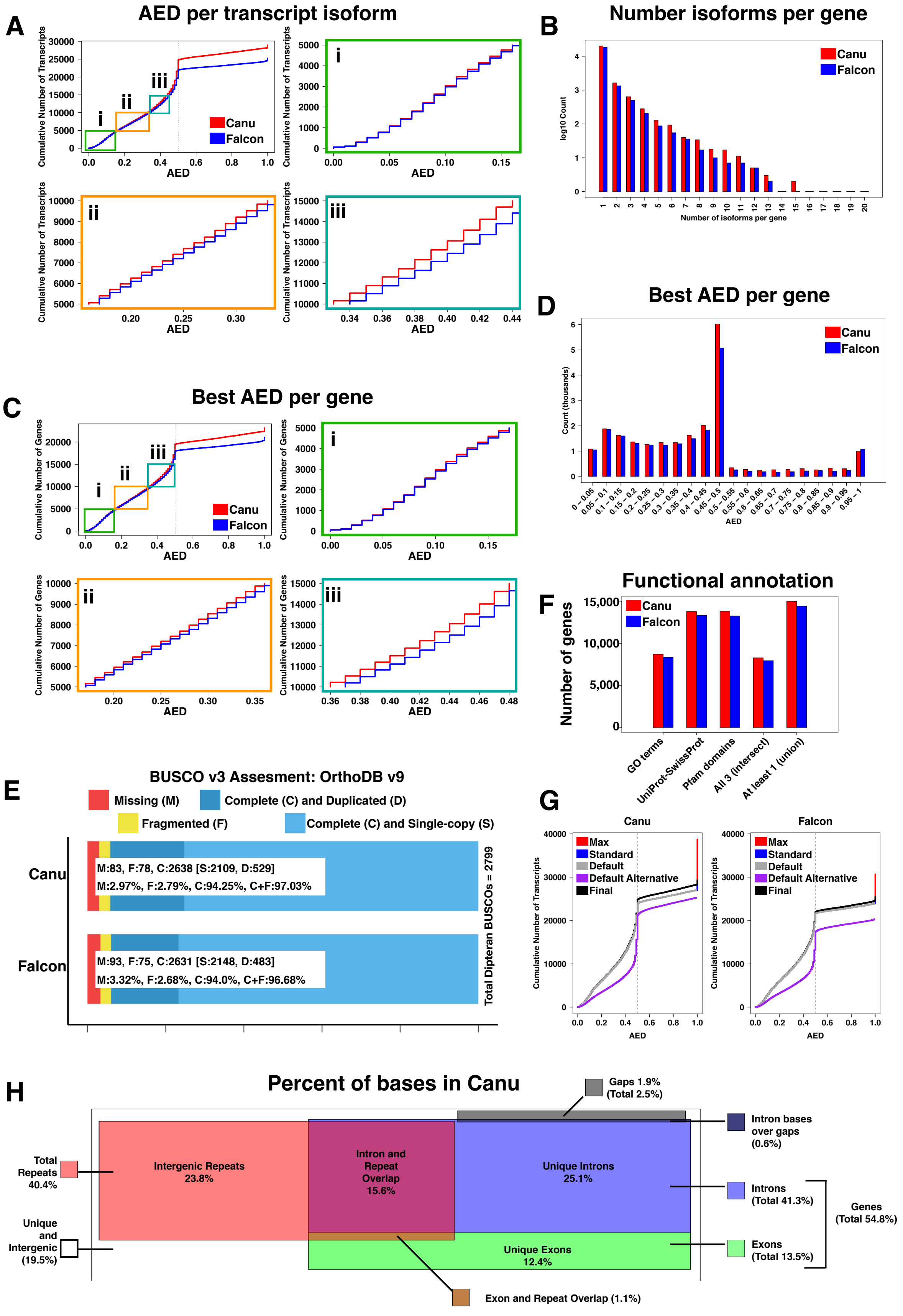

Supplemental Figure S14: Pairwise comparisons of final Canu and Falcon annotations.

(A) Shows the cumulative number of transcripts (y-axis) with respect to annotation edit distance (AED; x-axis). The (i) green, (ii) orange, and (iii) cyan boxes show subsequent subsets of 5000 transcripts up close. Canu had more transcripts with lower AEDs. **(B)** Canu typically has slightly more isoforms per gene than Falcon. **(C)** Shows the cumulative number of genes with respect to the best (lowest) AED used for each gene. Still the Canu annotation tends to have more genes with lower AEDs. **(D)** Shows the number of genes in each AED category. **(E)** Shows the BUSCO assessment results on the annotation transcript sets using all Dipteran BUSCOs (n=2799). Canu's annotation has a slightly higher % of BUSCOs found than Falcon's (97.03% vs 96.68%). **(F)** Visualization of the functional annotation results. The Canu annotation has more genes with GO terms, BLASTp hits to the entire UniProt-SwissProt protein dastabase, and/or with recognizable Pfam protein domains than the Falcon annotation. **(G)** Shows the cumulative number of transcripts with respect to AED for the different subsets (default, standard, and max) in the annotation that used the original repeat library. It also shows the default transcript set (purple) for the annotation that used the alternative/filtered repeat library for masking. Notice that the AED distribution is worse for that annotation. Therefore, only genes that had no overlapping representation in the original annotation were taken from that annotation (and they tended to be repetitive genes such as histones and olfactory receptors). See supplemental methods for more information. **(H)** A Venn-like diagram of boxes representing percentages of the Canu assembly annotated as genes (exons, introns), repeats, gaps, and overlaps amongst features.

Supplemental Figure S15: Dosage compensation of X-linked genes in *Sciara coprophila*

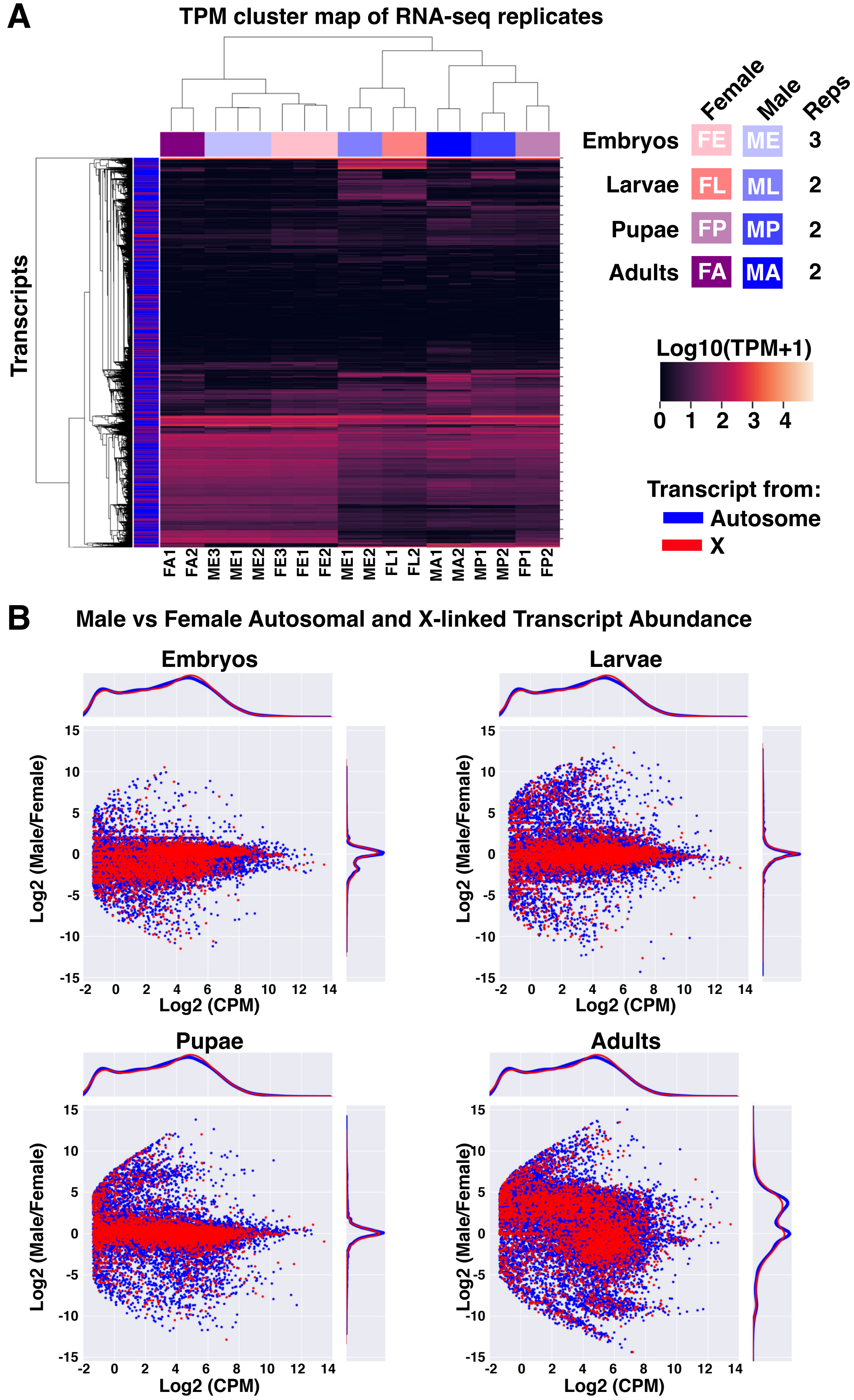

Supplemental Figure S15: Dosage compensation of X-linked genes in *Sciara coprophila*

(A) Clustered heatmap of transcript abundances represented as log10 Transcripts Per Million (TPM) demonstrates that the replicates were highly reproducible, that autosomal and X-linked genes span all expression levels in both sexes, and that transcript abundances of X-linked genes appear similar across sexes in the same stage.

(B) Scatter plots of the Log2 average Count Per Million vs the Log2 ratio of male to female transcript abundances. Dots are colored blue for autosomal genes and red for X-linked genes as in (A).

Supplemental Figure S16: Distribution of DNA modifications across *Sciara* genome (PacBio analysis)

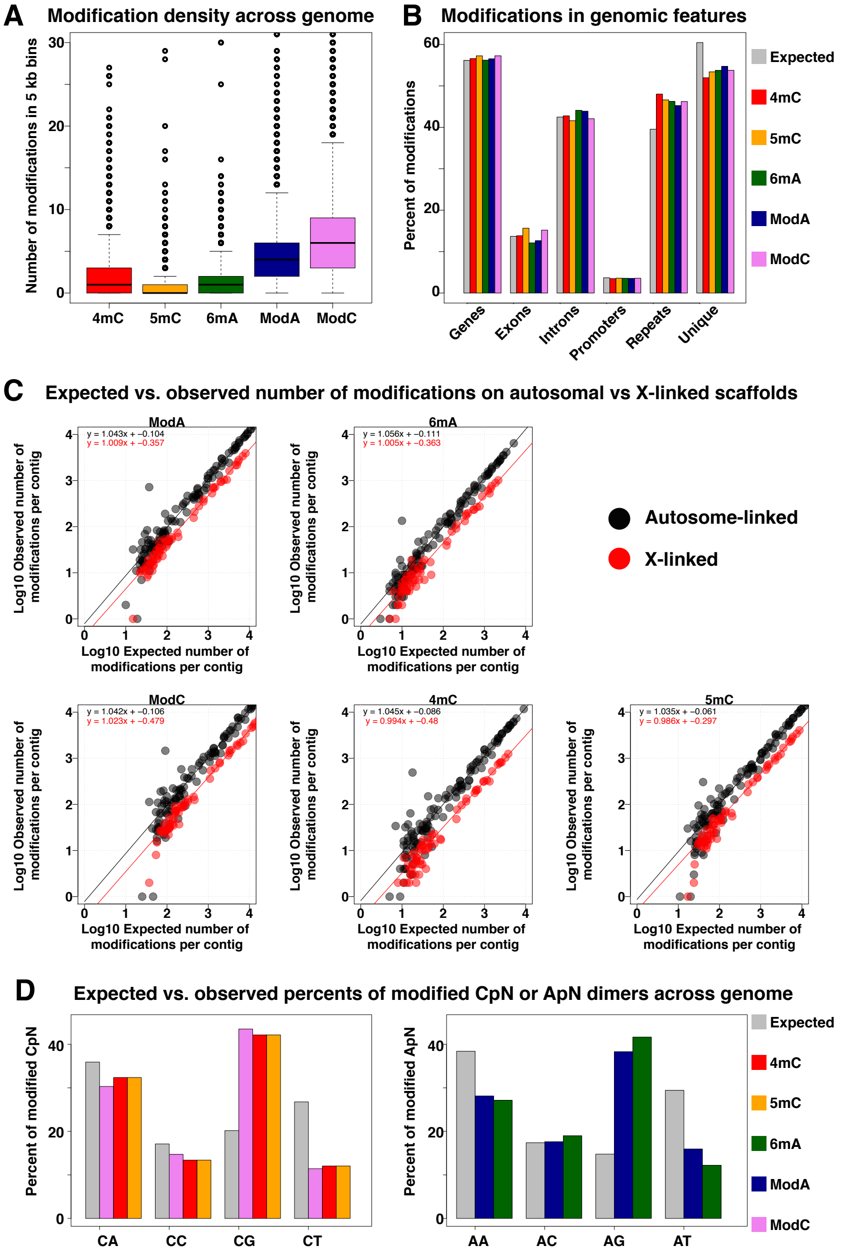

Supplemental Figure S16: Distribution of DNA modifications across *Sciara* genome (PacBio analysis)

(A) Boxplots of the number of modifications in 5 kb bins. Note that ModA and ModC are all adenines and cytosines, respectively, flagged as putative modified bases and 6mA or 4mC/5mC are subsets therein. Boxes span from the 25th-75th percentiles with the median denoted as a black line. Whiskers extend up/down from the box to datapoints no more than an additional 1.5x the interquartile range (IQR). Outliers are shown as circles. **(B)** Expected vs Observed proportion of DNA modifications in genomic features. See supplemental tables S20 A-D as well. **(C)** Expected vs observed counts on contigs/scaffolds in the primary assembly labeled as Arthropoda (phylum). X-linked sequences have fewer modifications than autosomal sequences, but this is likely due to the loss of sensitivity from having half the strand coverage. **(D)** For given C or A modification type, this shows the percent of modified CpN or modified ApN that each dimer makes up for that category. The expected percents are obtained from the percent of all CpN or ApN genome-wide. Also see Supplemental Table S18.

Supplemental Figure S17: Position weighted 7-mer motifs learned from different filtering and different subsets of the genome sequence (PacBio analysis)

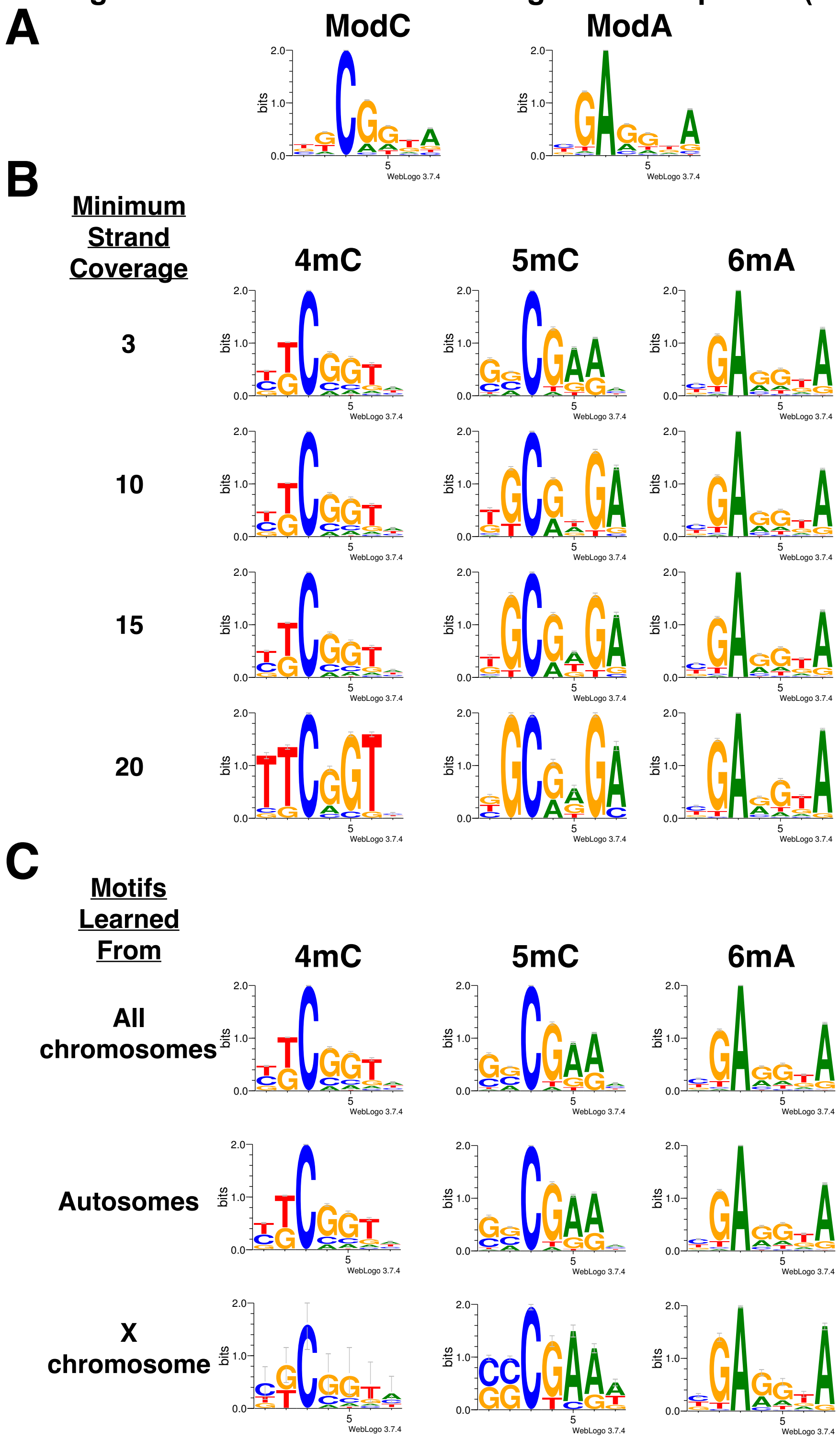

Supplemental Figure S17: Position weighted 7-mer motifs learned from different filtering and different subsets of the genome sequence (PacBio analysis)

(A-C) contain position weighted motifs from the sets of 7-mers (where the modified base occurs at position 3) enriched for 6mA, 4mC, or 5mC in the PacBio analysis. **(A)** Motifs from enriched 7-mers found in the analysis of all adenine or cytosine sites found as putatively modified. **(B)** Motifs learned from putative 4mC, 5mC, and 6mA sites with increasingly stringent filtering. **(C)** Either all sites were considered or the sites were partitioned into those on contigs we classified as chromosome X or autosomal. The motifs are the same in all cases.

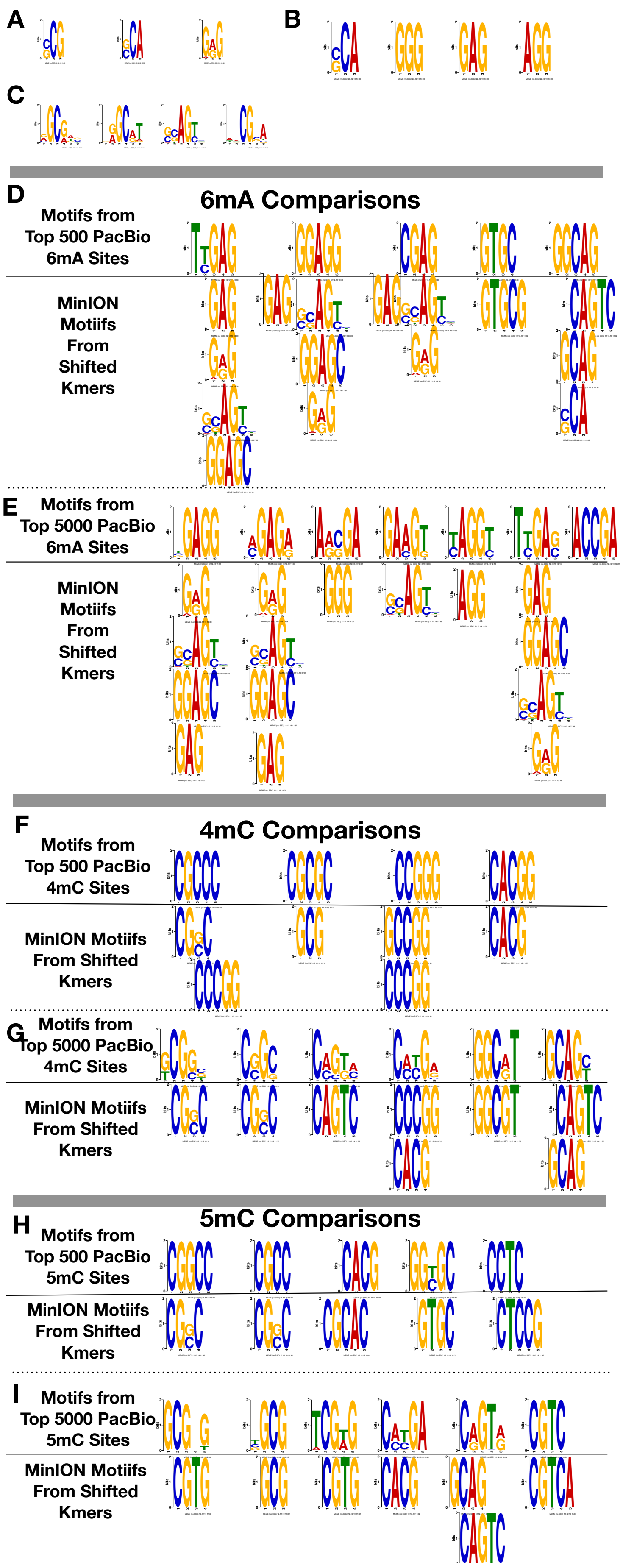

Supplemental Figure S18: MEME motifs in the PacBio and MinION analyses

(A) The top 3 trimer motifs in MEME analysis of kmers with shifted MinION signal.

(B) The top 4 trimer motifs in MEME analysis of kmers with shifted MinION signal when removing kmers with the CG dinucleotide from consideration.

(C) The top 4 6mer motifs in MEME analysis of kmers with shifted MinION signal.

(D) MEME motifs from top 500 PacBio 6mA sites compared to similar 2-6 bp motifs found in kmers with shifted MinION signal.

(E) MEME motifs from top 5000 PacBio 6mA sites compared to similar 2-6 bp motifs found in kmers with shifted MinION signal.

(F) MEME motifs from top 500 PacBio 4mC sites compared to similar 2-6 bp motifs found in kmers with shifted MinION signal.

(G) MEME motifs from top 5000 PacBio 4mC sites compared to similar 2-6 bp motifs found in kmers with shifted MinION signal.

(H) MEME motifs from top 500 PacBio 5mC sites compared to similar 2-6 bp motifs found in kmers with shifted MinION signal.

(I) MEME motifs from top 5000 PacBio 5mC sites compared to similar 2-6 bp motifs found in kmers with shifted MinION signal.

Supplemental Figure S19: MinION signal distributions for 6mers defined by motifs learned in the PacBio analysis and negative controls

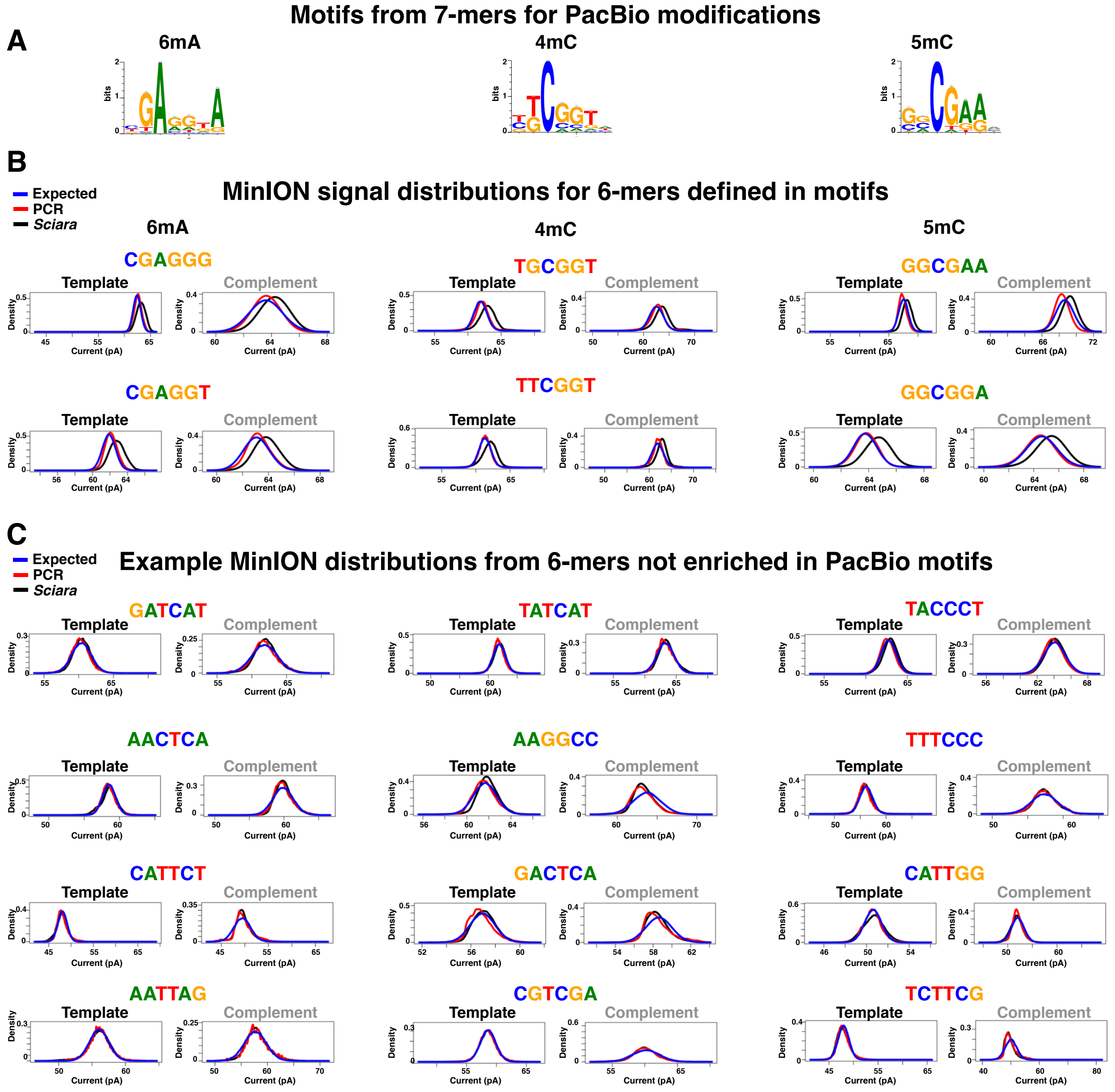

Supplemental Figure S19: MinION signal distributions for 6mers defined by motifs learned in the PacBio analysis and negative controls

(A) The motifs learned from PacBio analysis as shown elsewhere.

(B) MinION signal distributions for 6mers defined by motifs found for each DNA modification type in the PacBio analysis. For each 6mer, the expected distribution given the model provided by Oxford Nanopore Technologies (ONT) is shown in blue; the distribution learned from whole genome PCR of E. coli (using only canonical nucleotides) is shown in red; the distribution learned from native genomic DNA from male *Sciara* embryos is shown in black. Each 6mer shows the the expected and learned distributions for both the template and complement reads (R7.3).The distributions for *Sciara* are slightly offset from the model expectation whereas the learned model from PCR data is not.

(C) MinION signal distributions for 6mers not found enriched in the PacBio analysis. The distributions for *Sciara* match the distributions learned by PCR and expected from the model provided by ONT.

Supplemental Figure S20: The GCG trimer is depleted in the genome and transcriptome compared to expectation.

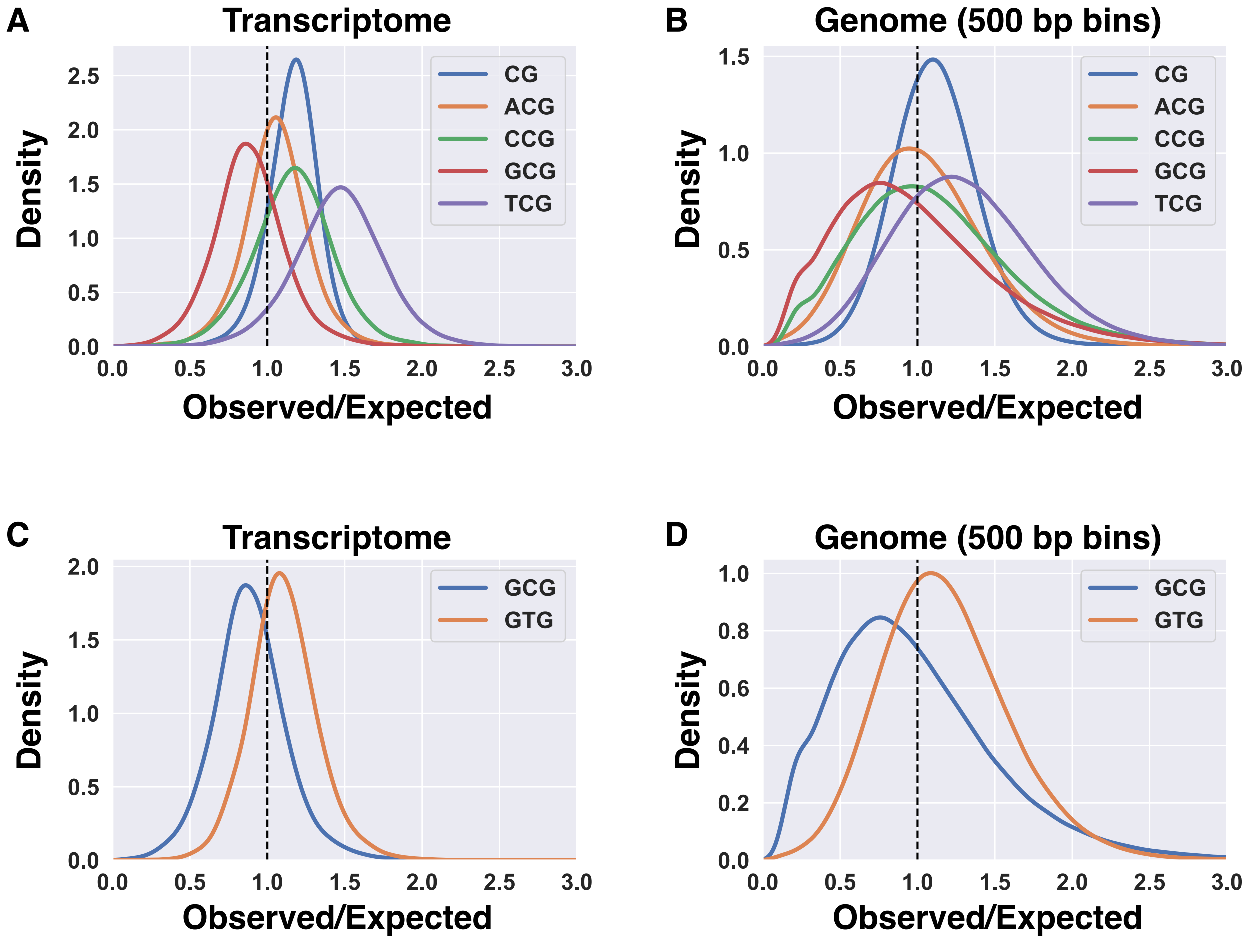

Supplemental Figure S20: the GCG trimer is depleted in the genome and transcriptome compared to expectation.
Methylated cytosines can spontaneously convert to thymine by deamination and thus germline CpG methylation predicts a depletion of CpG dimers in the genome over evolutionary time. In contrast, we found that there are more CpG dimers in the genome sequence than expected given the GC content. This may that the germline methylation and conversion rate(s) may be too low for this analysis or may be offset by other mutagenic forces in the germline genome (e.g. non-CG cytosine methylation). Alternatively, it may imply that CG is not a methylation target in the germline or at least not in all contexts. In support of the latter possibility, we found that GCG trimers were more enriched for DNA methylation than C or CG, which would predict a depletion of GCG trimers specifically. Interestingly, that is what we see. GCG trimers are depleted in the **(A)** transcriptome and **(B)** genome sequences with respect to expected levels (see supplemental methods). GCG depletion in the germline by way of C→T conversion over evolutionary timescales would also predict that GTG is enriched over expectation, which we also see in the **(C)** transcriptome and **(D)** genome sequences.

Supplemental Figure S21: Distribution of distances between adjacent DNA modifications (PacBio analysis) on the same strand shows enrichment of short distances, a 10 bp periodicity, and a spike of enrichment at mono-nucleosome lengths of ~175 bp.

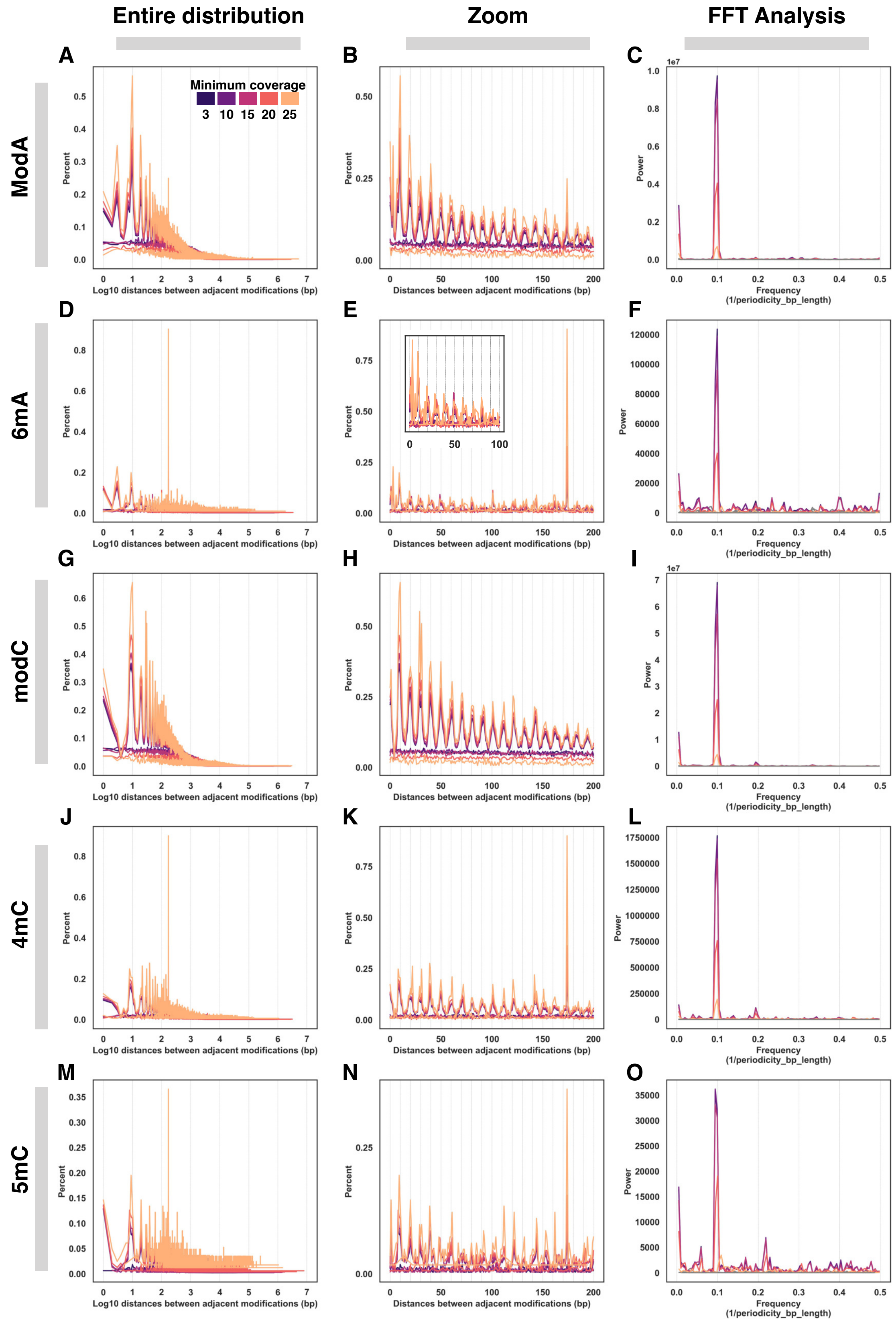

Supplemental Figure S22: Distribution of distances between adjacent DNA modifications (PacBio analysis) on either strand also shows enrichment of short distances, a 10 bp periodicity, and a spike of enrichment at mono-nucleosome lengths of ~175 bp for 6mA and 5mC.

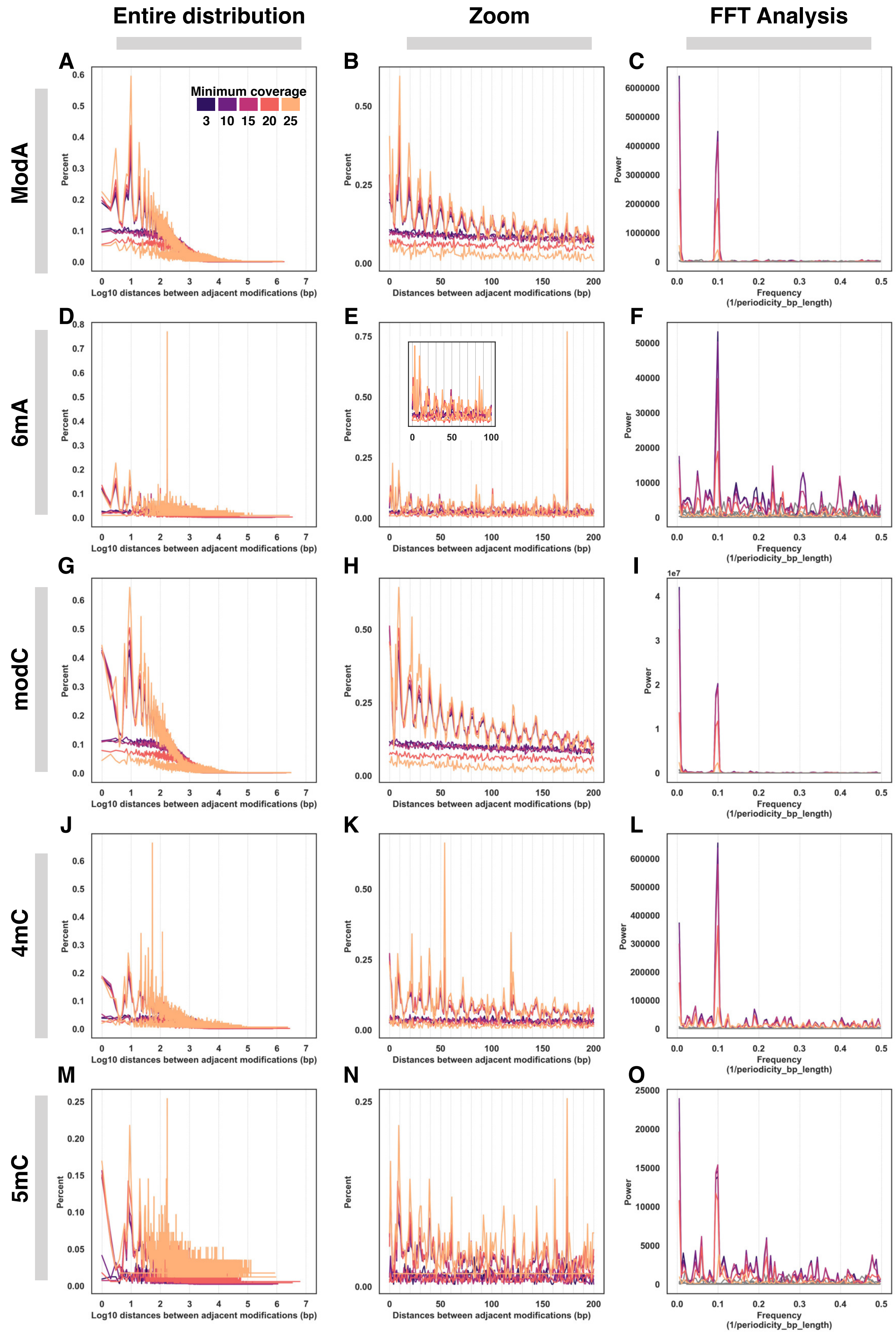

Legend for both Supplemental Figure S21 and S22:
For each row that features a given type of modification identified in the PacBio analysis, the left column shows the entire distribution of log10 distances between adjacent modifications normalized as the percent of all distances, the middle column shows a zoom in of distances between 0-200 bp normalized as the percent of all distances, and the right column shows the fast fourier transform analysis of the distances to identify the frequency with the highest power that is converted to periodicity by 1/frequency (1/0.1 = 10 bp). The inset in the E panels shows a further zoom in to distances between 0-100 to illustrate that the wavelength pattern is there, but looks extremely compressed due to the high spike at ~175 bp.

modA is the set of adenine sites flagged as modified by kineticsTools and 6mA is the subset therein identified specifically as 6mA. modC is the set of cytosine sites flagged as modified by kineticsTools whereas 4mC and 5mC are the subsets therein specifically identified as those modifications. Sites with modification scores (phred-transformed P-value that a kinetic deviation exists) <20 were filtered out and each plot shows the results of further filtering for sites with minimum coverages of 3X, 10X, 15X, 20X, or 25X. Moreover, ModA and ModC can be considered the unfiltered set of all putative modified sites (given the score and coverage cutoffs) whereas 6mA, 4mC, and 5mC can be considered the further filtered subsets of those sites that met classification criteria for those modifications. Finally, we also looked at the distances between neighboring modifications on the same strand or neighboring modifications on either strand. The results and conclusions were highly robust in all scenarios, with slightly stronger and cleaner effects in the strand-specific analysis.

In the left and middle columns that show distributions of distances, there are pairs of tests and controls with matching colors based on the minimum coverage used. The tests are the actual distribution of distances between DNA modifications and are the set of wave-like lines above the flatter ones (controls). The controls are the distances between the same number of “modifications” as the test obtained by randomly selecting adenines for modA/6mA or randomly selected cytosines for modC/4mC/5mC. The control lines are the set of relatively flat lines under the taller waves (tests). Control line colors match the colors for the paired test. For the FFT analysis, test lines are the same colors, but control lines were made grey as they all are essentially flat lines at 0.

Section 2: Supplemental Tables

Supplemental Table S1 A-E: Expected genome and chromosome sizes

Supplementary Table S1A

DNA content measurements (Rasch 2006)							
**Sex**	**Stage / Tissue**	**Cell Type**	**Chromosomes***	**N**	**Mean (pg)**	**Std Error (pg)**	**Total Length (Mb)****
Female	4th instar larvae	Hemocytes	2X+2A	65	0.571	0.004	558.438
Male	4th instar larvae	Hemocytes	1X+2A	77	0.523	0.007	511.494
Female	Pharate pupae	Hemocytes	2X+2A	74	0.591	0.004	577.998
Male	Pharate pupae	Hemocytes	1X+2A	38	0.540	0.003	528.12
Female	Adult	Hemocytes	2X+2A	74	0.554	0.003	541.812
Female	Adult	Hemocytes	2X+2A	62	0.557	0.004	544.746
Male	Adult	Hemocytes	1X+2A	100	0.503	0.002	491.934
Male	Adult 44A testis	Sperm	2X+1A+2L	162	0.491	0.016	480.198
Male	Adult 44A duct	Sperm	2X+1A+2L	60	0.494	0.017	483.132
Male	Adult 43 E testis	Sperm	2X+1A+2L	100	0.519	0.018	507.582
Male	Adult 43 E duct	Sperm	2X+1A+2L	50	0.521	0.013	509.538
Male	Adult 45 testis	Sperm	2X+1A+2L	65	0.535	0.014	523.23
Male	Adult 45 duct	Sperm	2X+1A+2L	50	0.484	0.019	473.352

*Assumes two L chromosomes are present in the sperm. Though this is most common, it can range from 0-4.
** The Mb length of all DNA in the nucleus given pg mean and Dolezel conversion (Dolezel et al. 2003).

Supplementary Table S1B

Chromosome X				
**Stage**	**Female (pg)**	**Male (pg)**	**Chromosome X (pg) (Difference)**	**Chromosome X (Mb)**
Larvae	0.571	0.523	0.048	46.944
Pupae	0.591	0.540	0.051	49.878
Adult*	0.555	0.503	0.052	50.856
Mean	0.572	0.522	0.05	48.9

*Since two female adult values are in Table A, the female adult value was obtained by: (74 ∗ 0.554 + 62 ∗ 0.557)/(74 + 62) = 0.555

Supplementary Table S1C

Expected length of the haploid somatic genome: Chromosomes X, II, III, and IV							
**Stage**	**Sex**	**Total DNA content (pg)**	**X DNA content (pg)**	**Total – X (pg)**	**Diploid length of autosomes: II, III, and IV (Mb)**	**Haploid length of autosomes: II, III, and IV (Mb)**	**Haploid length of X + autosomes (Mb)**
Larvae	Female	0.571	0.1	0.471	460.638	230.319	279.219
Larvae	Male	0.523	0.05	0.473	462.594	231.297	280.197
Pupae	Female	0.591	0.1	0.491	480.198	240.099	288.999
Pupae	Male	0.540	0.05	0.490	479.22	239.61	288.51
Adult	Female	0.555	0.1	0.455	444.99	222.495	271.395
Adult	Male	0.503	0.05	0.453	443.034	221.517	270.417
**Mean**	**-**	**-**	**-**	**0.472**	**461.779**	**230.889**	**279.789**

*Haploid estimates range from ~270-290 Mb

Supplementary Table S1D

DNA content contribution from L chromosomes in sperm and expected germline genome size							
DNA content in sperm*	Length of DNA in Sperm: 2X+1A+2L** (Mb)	Length of haploid autosomes (Mb)	Length of X (Mb)	Length of 2X + 1 set of autosomes (Mb)	Length of contribution from L chromosomes: Total – (2X+A) (Mb)	Length of L	Haploid germline size (1A + 1X + 1L)
0.5053531	494.235	230.889	48.9	328.689	165.546	82.773	362.562

* The weighted average for DNA content in sperm was the sum(N*mean)/sum(N) for the N and M values in Table A.
**Assumes two L chromosomes are present in the sperm. Though this is most common, it can range from 0-4. If one assumed the most majority of sperm had 1, 2, 3, or 4 Ls, the possible L chromosome lengths are between 41.4-165.5 Mb and the haploid genome size is between 321.2-445.3 Mb.

Supplementary Table S1E

Expected X and Autosome Lengths given polytene zones (Gabrusewycz-Garica 1964)					
Chromosome	Number of polytene zones	Expected proportion of somatic genome: (X, II, III, IV)	Expected Length (Mb) given haploid somatic genome size (279.789)	Expected proportion of autosomes: (II, III, IV)	Expected Length (Mb) given sum of haploid autosome sizes (230.889)
X	14	0.222222222	62.17533333	0	(48.9)
II	14	0.222222222	62.17533333	0.285714286	65.96828571
III	15	0.238095238	66.61642857	0.306122449	70.68030612
IV	20	0.317460317	88.82190476	0.408163265	94.24040816
Sum	63	1	279.789	1	279.789

Supplementary Table S1 A-E: Expected genome and chromosome sizes

(A) We wanted to estimate the approximate length of the haploid genome in the germline (ChrII, ChrIII, ChrIV, ChrX, ChrL) as well as the somatic haploid genome length (ChrII, ChrIII, ChrIV, ChrX). To do so, we used started with the values in Table 2 from Rasch (2006) (Rasch 2006), adapted here.
(B) Since male somatic cells have a single X and female somatic cells have two, the pg weight and bp length of a single chromosome X can be inferred by subtracting the male value from the female value.
(C) The diploid autosome DNA content and bp length can be inferred by subtracting the value of 1X for males or 2X for females. One can then deduce the haploid autosome DNA content and length by dividing by 2, and can subsequently deduce the expected somatic haploid genome (chrII, chrIII, chrIV, chrX) DNA content and length by adding the value of a single X. Thus, the genome assemblies we generate should be ~280 Mb reflecting the expected haploid somatic genome length (chrII, chrIII, chrIV, chrX).
(D) The expected length of the haploid germline genome can be deduced by adding the weight of a single L chromosome. The sperm data from sperm in Table S1A represents one set of autosomes, two X chromosomes, and a variable number of L chromosomes (most commonly two). So the L content can be found by subtracting 2X+A from the total. We took the weighted average DNA content in sperm. As discussed by Rasch (Rasch 2006) and demonstrated by Rieffel and Crouse (Rieffel and Crouse 1966), there can be between 0 and 4 copies of the L chromosome in a sperm nucleus, though the majority have 2 copies (78% according to Rasch). The length in the table assumes two L per sperm. Effects from other possibilities are noted too.
(E) Assuming the number of polytene zones correlates with chromosome length in base pairs, one can estimate the relative chromosome sizes compared to each other, and use those relative proportions with the estimated summed length of all four to estimate chromosome lengths. This results in an X chromosome length that longer than DNA content estimates (49 Mb vs 62 Mb). We can also fix the X length as estimated above and use the relative proportions of only chromosomes II, III, and IV with their estimated summed length from DNA content. Overall, we expect X to be 48-62 Mb, II to be 62-66 Mb, III to be 66-71 Mb, and IV to be 88-94 Mb.

Supplemental Table S2: RNA-seq samples spanning both sexes and 4 life cycle stages

Sample	Replicate #	NEB Set #	Barcode Index	inPrimerSeq*	ReadSeq*	Number of paired-reads
**Female embryos**	1	1	index 1	CGTGAT	ATCACG	5,904,586
**Female embryos**	2	1	index 2	ACATCG	CGATGT	6,136,865
**Female embryos**	3	1	index 3	GCCTAA	TTAGGC	4,941,561
**Male embryos**	1	1	index 4	TGGTCA	TGACCA	5,608,743
**Male embryos**	2	1	index 5	CACTGT	ACAGTG	4,891,639
**Male embryos**	3	1	index 6	ATTGGC	GCCAAT	6,942,968
**Female Larvae**	1	1	index 7	GATCTG	CAGATC	5,465,807
**Female Larvae**	2	1	index 8	TCAAGT	ACTTGA	5,776,190
**Male Larvae**	1	1	index 9	CTGATC	GATCAG	6,453,818
**Male Larvae**	2	1	index 10	AAGCTA	TAGCTT	5,694,394
**Female Pupae**	1	1	index 11	GTAGCC	GGCTAC	5,177,694
**Female Pupae**	2	1	index 12	TACAAG	CTTGTA	5,532,750
**Male Pupae**	1	2	index 13	TTGACT	AGTCAA	15,280,707
**Male Pupae**	2	2	index 14	GGAACT	AGTTCC	14,022,001
**Female adults**	1	2	index 15	TGACAT	ATGTCA	5,485,023
**Female adults**	2	2	index 16	GGACGG	CCGTCC	13,704,291
**Male adults**	1	2	index 18*	GCGGAC	GTCCGC	11,741,449
**Male adults**	2	2	index 19	TTTCAC	GTGAAA	10,582,159

Supplemental Table S3: Short read assembly size statistics

Assembly	N	ASM_SIZE	MAX	MIN	MEAN	MEDIAN	N50	L50	E	NG50 280M	LG50 280M	E 280M	Matrix Row	Nickname
**abyss.k55**	303676	277836422	1054107	55	915	109	5938	12383	18271	5880	12566	18130	1	a1
**abyss.k55.bh**	302988	278198827	1054115	55	918	109	5993	12280	18542	5943	12431	18422	2	a2
**abyss.k55.q5**	303987	276373215	1025867	55	909	109	5796	12678	16597	5695	12993	16382	3	a3
**abyss.k55.q5.bh**	303005	276740830	1053964	55	913	109	5851	12559	17462	5764	12840	17258	4	a4
**abyss.k77**	186647	273406983	1060849	77	1465	169	5026	14870	15634	4875	15536	15265	5	a5
**abyss.k77.bh**	186091	275264727	1054512	77	1479	168	5120	14670	16138	5024	15137	15865	6	a6
**abyss.k77.q5**	188860	273874388	707552	77	1450	171	4971	15031	13590	4833	15655	13292	7	a7
**abyss.k77.q5.bh**	186022	273581412	1054487	77	1471	170	5039	14825	15353	4893	15471	15001	8	a8
**megahit**	203144	314764763	805548	200	1549	540	4309	17696	13631	5123	13994	15323	9	m1
**megahit.bh**	200747	312783966	687499	200	1558	539	4340	17552	13114	5104	14065	14650	10	m2
**megahit.q5**	194936	308417993	682464	200	1582	541	4379	17352	12459	5053	14330	13723	11	m3
**megahit.q5.bh**	194640	308016525	682470	200	1582	542	4379	17394	12473	5034	14408	13721	12	m4
**platanus**	88584	235992359	801720	81	2664	1354	5454	11586	17358	4287	16133	14630	13	p1
**platanus.bh**	88132	237450965	936960	96	2694	1359	5536	11484	18261	4418	15787	15486	14	p2
**platanus.q5**	88177	240633956	908679	94	2729	1373	5660	11477	17730	4616	15333	15238	15	p3
**platanus.q5.bh**	87570	241582189	908703	81	2759	1382	5755	11387	17501	4718	15079	15100	16	p4
**sga**	160775	266601738	907684	200	1658	628	3880	18376	12870	3642	20157	12254	17	sg1
**sga.q5**	167119	264933802	907675	200	1585	607	3702	19463	12648	3452	21571	11967	18	sg2
**soapdenovo2.k55**	339967	315872819	907788	100	929	115	6525	12023	20769	7729	9497	23430	19	so1
**soapdenovo2.k55.bh**	357836	318047022	909706	100	889	111	6327	12399	19605	7609	9658	22269	20	so2
**soapdenovo2.k55.q5**	315548	311171324	907784	100	986	126	6596	11897	19175	7630	9700	21310	21	so3
**soapdenovo2.k55.q5.bh**	302550	310634015	907798	100	1027	145	6589	11855	19173	7625	9695	21271	22	so4
**soapdenovo2.k77**	244672	282948474	907817	100	1156	155	5747	13036	17684	5828	12781	17870	23	so5
**soapdenovo2.k77.bh**	261683	285615548	908360	100	1091	155	5710	13165	17883	5869	12681	18242	24	so6
**soapdenovo2.k77.q5**	237972	280534633	1025525	100	1179	155	5621	13222	17567	5635	13175	17600	25	so7
**soapdenovo2.k77.q5.bh**	237446	281739587	907721	100	1187	155	5776	12954	17037	5825	12804	17143	26	so8
**spades.bh.k213355**	380982	347635213	1026350	1	912	146	4229	19112	15992	5882	12309	19855	27	sp1
**spades.bh.k21335577**	286478	348582982	1228455	18	1217	252	5552	14564	22018	7768	9340	27411	28	sp2
**spades.bh.k21335577auto**	279669	345817875	1228455	18	1237	249	5630	14317	22188	7768	9340	27404	29	sp3
**spades.q5.bh.k213355**	382055	344292232	1220242	1	901	152	4214	18998	16637	5803	12479	20457	30	sp4
**spades.q5.bh.k21335577**	278353	343736955	1227608	18	1235	260	5618	14336	20503	7675	9477	25170	31	sp5
**spades.q5.bh.k21335577auto**	271253	340884856	1227608	18	1257	257	5703	14085	20668	7675	9478	25163	32	sp6
**velvet.k55**	160994	233337453	1109576	109	1449	350	4202	15121	18120	3266	21424	15100	33	v1
**velvet.k55.bh**	186320	235611858	1110712	109	1265	337	3515	18288	15265	2780	25390	12845	34	v2
**velvet.k55.q5**	159581	231243264	1108829	109	1449	352	4189	15075	17438	3217	21718	14402	35	v3
**velvet.k55.q5.bh**	159511	233215384	1108932	109	1462	358	4211	15078	17083	3280	21374	14229	36	v4
**velvet.k77**	125780	229591350	1109932	153	1825	823	4075	15985	16289	3158	23010	13356	37	v5
**velvet.k77.bh**	125522	232398455	1110079	153	1851	851	4104	16073	17076	3237	22606	14173	38	v6
**velvet.k77.q5**	124497	226321787	882517	153	1818	824	4045	15879	14163	3066	23502	11448	39	v7
**velvet.k77.q5.bh**	124026	230127881	1111298	153	1855	850	4113	15846	17911	3203	22718	14721	40	v8
**platanus.blobfilt1**	87119	227977694	75265	81	2617	1368	5314	11982	7242	3979	17647	5897	NA	p5
**platanus.blobfilt1.bh**	87696	228227902	75308	96	2602	1355	5299	12005	7233	3985	17647	5895	NA	p6
**platanus.blobfilt1.q5**	86482	232676523	60214	95	2690	1393	5519	11815	7472	4283	16683	6209	NA	p7
**platanus.blobfilt1.q5.bh**	87509	229834980	63160	99	2626	1366	5350	12030	7269	4073	17403	5967	NA	p8

Supplemental Table S3: Short read assembly size statistics
The naming scheme is "*assembler.parameters.input*" where parameters for some assemblers were kmer sizes of 55 or 77 (k55, k77), or up to k=55 or k=77 for SPAdes (e.g. k21335577 used kmer sizes 21, 33, 55, and 77), and where the input was quality-filtered (q5), error-corrected (bh), both (q5.bh), or the raw input reads otherwise. For SPAdes, "auto" means "—cov-cutoff auto" was set. For Platanus, "blobfilt1" indicates that these were "re-assemblies" with contaminating reads removed after blobtools analysis (Laetsch and Blaxter 2017). The assemblies are presented in the same order that their ranks are visualized in the ranking matrix in Figure 2B. The "Matrix Row" and "Nickname" columns additionally help connect the table with that figure.

Supplemental Table S4: Long read assembly size statistics

Assembly	N	ASM_SIZE	MAX	MIN	MEAN	MEDIAN	N50	L50	E	NG50 280M	LG50 280M	E 280M	Matrix Row	Nickname
abruijn.pball.ontmol	544	285817077	10540070	53798	525399	227013	1222757	58	2112258	1281449	56	2156141	1	A1
abruijn.pball	732	281500939	5690677	50217	384564	161202	826831	76	1516868	832247	75	1525000	2	A2
abruijn.pbfilt.ont2d	541	284906646	10747290	48902	526630	210027	1294301	49	2475897	1338369	47	2519284	3	A3
canu10.minrl1000.dip3x.pbfilt	1316	303769988	21228270	1732	230828	21211	2554706	23	5321262	3425454	19	5772999	4	C0.1
canu10.minrl500.dip3x.pball	1236	303631862	26730757	1213	245657	21871	2570070	27	5442641	3008968	22	5901998	5	C0.2
canu11.corcov80minrl500.dip3x.pball.ontmol	1537	296991896	20910109	3770	193228	32147	1188978	42	3975708	1337456	35	4216975	6	C1.1
canu12.corcov80minrl500.dip3x.pball.ontmol	1225	299099912	17969101	4139	244163	34799	2399381	26	4373842	2819977	23	4672199	7	C2.1
canu12.minrl500.dip3x.pball.ontmol	1213	297711070	19547680	4524	245434	39809	1958195	30	4530411	2149314	26	4816976	8	C2.2
canu.corcov500minrl500.aspb-e025.pball.ontmol	1036	302365066	12318386	793	291858	28771	1908694	37	3173265	2367475	31	3426730	9	C3.1
canu.corcov500minrl500.aspb-e02.pball.ont2d	1044	302258594	12317152	671	289520	28311	1908286	37	3173951	2363127	31	3426264	10	C3.2
canu.corcov500minrl500.aspb-e02.pbfilt.ont2d	1044	302239832	12315397	671	289502	28307	1908501	37	3173821	2361875	31	3425912	11	C3.3
canu.corcov500minrl500.pball.ont2d	1104	303257430	12695061	792	274690	31720	1506847	45	2690343	1724831	38	2913809	12	C3.4
canu.corcov500minrl500.pball.ontmol	1107	303232626	12696256	792	273923	31352	1516021	44	2721930	1777033	37	2947779	13	C3.5
canu.corcov500minrl500.pball	1231	300698388	11589796	587	244272	27608	1764129	42	2623585	1918491	36	2817528	14	C3.6
canu.corcov500minrl500.pbfilt	1224	300534892	11589685	793	245535	29497	1654609	43	2722880	1823318	37	2922573	15	C3.7
canu.corcov500.pball	1240	300804020	11589802	1133	242584	28527	1674691	43	2580825	1848220	37	2772581	16	C3.8
canu.corcov500.pbfilt.ont2d	1139	301454870	10743861	1023	264666	30368	1391244	47	2535311	1603630	40	2729578	17	C3.9
canu.corcov500.pbfilt	1232	300545669	11589555	1133	243949	29275	1676452	41	2993594	1823280	35	3213256	18	C3.10
canu.default.pball	1192	298757505	12235276	1189	250635	30880	1649665	42	2860691	1832171	37	3052331	19	C3.11
canu.minrl500.pball	1226	299103658	12235409	792	243967	28570	1636496	42	2868504	1832154	37	3064214	20	C3.12
falcon.default.pball	702	294632888	11528295	406	419705	32855	2516799	29	3755975	2730335	26	3952264	21	F1
falcon.default.pbfilt	704	295192605	12753606	283	419308	30728	2785086	29	3850555	2973441	27	4059483	22	F2
falcon.seed25.pball.ont2d	728	297366589	11078332	519	408471	22849	2546290	30	3585374	2596709	27	3807751	23	F3
falcon.seed25.pball.ontmol	737	296668450	12400955	406	402535	25656	2519867	28	3809884	2730274	25	4036687	24	F4
falcon.seed25.pball	671	295939241	12785397	148	441042	25122	3072189	28	3978720	3456700	25	4205212	25	F5
falcon.seed25.pbfilt.ont2d	724	297400499	11078438	519	410774	22977	2546297	30	3585986	2596645	27	3808836	26	F6
falcon.seed25.pbfilt	712	295735287	9828760	145	415359	21976	2943872	28	3599286	3316978	26	3801557	27	F7
falcon.seed25.relaxed.pball.ont2d	709	296742085	12794951	117	418536	19083	2626834	28	3927881	3100919	25	4162742	28	F8
falcon.seed25.relaxed.pball.ontmol	713	296559753	20165452	119	415932	18659	2878051	24	5050163	3454019	21	5348840	29	F9
falcon.seed25.relaxed.pbfilt.ont2d	705	296734699	12795016	117	420900	19089	2626878	28	3928187	3100874	25	4162962	30	F10
falcon.seed30.pball	703	296128944	15921957	148	421236	19231	2883831	30	3890853	3014947	27	4114979	31	F11
falcon.seed30.pbfilt	713	296392938	12458309	86	415698	19624	2564544	29	3845400	2929617	26	4070533	32	F12
miniasm.racon.pball.ont2d	682	290016397	7561531	4803	425244	150330	1161069	64	1732387	1221342	60	1794359	33	M1
miniasm.racon.pball.ontall	669	289847742	7563691	2427	433255	154789	1179233	62	1736678	1237602	58	1797758	34	M2
miniasm.racon.pball.ontmol	691	289860660	7565108	2576	419480	146701	1095353	67	1682131	1161141	63	1741370	35	M3
miniasm.racon.pball	913	289699308	6296002	1616	317305	103582	1008157	66	1632180	1100088	61	1688720	36	M4
miniasm.racon.pbfilt.ont2d	687	290025924	7561922	4803	422163	146787	1161098	61	1807223	1234830	57	1871934	37	M5
miniasm.racon.pbfilt.ontall	671	289995708	7560375	4803	432184	150177	1161104	62	1794981	1234832	58	1859060	38	M6
miniasm.racon.pbfilt.ontmol	689	290105515	7560362	4803	421053	145499	1161100	61	1816611	1234831	57	1882174	39	M7
miniasm.racon.pbfilt	920	289863434	8266389	4948	315069	104506	962801	67	1724677	1100085	62	1785432	40	M8
platanus.dbg2olc.pball.ont2d	1309	304011299	2365216	1000	232247	104763	519314	176	652377	557486	153	708321	41	PD1
platanus.dbg2olc.pball.ontmol	1385	306594506	2113652	999	221368	102361	473926	192	605128	513068	165	662603	42	PD2
platanus.dbg2olc.pball	1491	302754463	1940333	1479	203055	113097	396110	229	502846	428139	202	543711	43	PD3
platanus.dbg2olc.pbfilt.ont2d	1309	304009150	2364200	1000	232245	104763	519111	176	652333	557461	153	708268	44	PD4
platanus.dbg2olc.pbfilt	1479	302538025	2204874	2336	204556	113907	405052	225	515659	433416	198	557166	45	PD5
smartdenovo.pball.ont2d	713	292871890	3556032	13027	410760	199388	928894	97	1121622	956687	90	1173184	46	S1
smartdenovo.pball.ontmol	698	292525170	3556036	15626	419091	208056	932578	94	1143652	959911	88	1194811	47	S2
smartdenovo.pball	638	291784268	7505804	7277	457342	177734	1098245	67	1689097	1146154	62	1760185	48	S3
smartdenovo.pbfilt.ont2d	713	292871890	3556032	13027	410760	199388	928894	97	1121622	956687	90	1173184	49	S4
smartdenovo.pbfilt	633	291543073	7505612	11967	460574	184935	1083964	66	1708535	1140563	61	1778969	50	S5

Supplemental Table S4: Long read assembly size statistics

The naming scheme is approximately “assembler.[parameters].pacbio-input.[minion-input]” where items inside square brackets are optionally present. The assemblies are presented in the same order that their ranks are visualized in Figure 2E. The “Matrix Row” and “Nickname” columns additionally help connect the table with Figure 2E. The assemblies chosen for scaffolding are in red and green, the latter being the assemblies chosen to move forward with after scaffolding. The Canu assembly in green (C3.2) is the assembly that went on to be chosen as the first released draft genome sequence.

Supplemental Table S5: Pairwise comparisons of size statistics of hybrid scaffolds from pair of Canu or Falcon assemblies

	Canu		Falcon	
**Version**	1 (C3.2)	2 (C3.1)	1 (F8)	2 (F9)
**Scaffold Set**	1	2	3	4
**PacBio**	All	All	All	All
**MinION**	2D	Molecule	2D	Molecule
**Number of sequences**	857	847	603	608
**Total length**	311,020,781	311,186,377	302,764,727	303,252,712
**Max sequence length**	15,214,119	15,220,704	21,064,549	28,442,430
**Min sequence length**	671	793	117	119
**Mean sequence length**	362,918.1	367,398.3	502,097.4	498,770.9
**Median sequence length**	21,642	21,891	16,349	15,738
**Sequence length N50**	5,861,178	5,860,508	7,077,677	9,560,626
**Sequence length L50**	16	16	12	12
**Expected sequence length**	6,349,017	6,378,079	8,115,036	9,440,067
**Sequence length NG50**	6,689,144	6,689,144	7,509,349	10,026,878
**Sequence length LG50**	14	14	12	11
**Normalized expected sequence length**	7,052,416	7,088,469	8,774,810	10,224,021

Supplemental Table S6: Size statistics of Canu C3.2 across the work flow

Canu

	Assembly	Hybrid Scaffolds	Meta-scaffolds	*Meta-scaffolds: Bacterial removed	**Primary assembly	Anchored
Number of sequences	1,044	857	769	744	205	92
Total length	302,258,594	311,020,781	312,049,034	309,775,056	298,965,442	136,508,149
Max sequence length	12,317,152	15,214,119	23,039,227	23,039,227	23,039,227	15,234,923
Min sequence length	671	671	671	671	4264	8,393
Mean sequence length	289,519.7	362,918.1	405,785.5	416,364.3	1,458,368	1,483,784.2
Median sequence length	28,310.5	21,642	21,093	21,428	94,665	76,618
Sequence length N50	1,908,286	5,861,178	6,689,181	6,790,317	6,790,317	6,666,656
Sequence length L50	37	16	15	14	14	7
Expected sequence length	3,173,951	6,349,017	7,930,712	7,982,431	8,269,986	7,447,096
Sequence length NG50	2,363,127	6,689,144	8,288,951	8,288,951	8,288,951	8,393
Sequence length LG50	31	14	12	12	12	92
Normalized expected sequence length	3,426,264	7,052,416	8,838,469	8,831,279	8,830,144	3,630,676

*All sequences here deposited to NCBI. One short contig (< 1kb) was flagged for removal. Thus, one can expect 743 sequences.

**The primary assembly contains contigs such that bacterial, ‘remove tigs’, and ‘redundant contigs’ are removed and those retained are marked as KEEP by purge_haplotigs. When keeping all of the non-bacterial KEEP scaffolds from purge_haplotigs (Roach et al. 2018) there are 265 instead of 205. The difference is that purge_haplotigs retains 54 of the contigs originally marked for removal (max size of 27.4 kb, mean size of 12.5 kb, sum of ~673 kb) and retained 6 contigs our minimap2-alone approach marked as “redundant” (max size of 27.5 kb, mean size of 20.4 kb, and sum of ~122 kb).

Supplemental Table S7: Size statistics of Falcon F9 across the work flow

Falcon

	Assembly	Hybrid Scaffolds	Meta-scaffolds	Meta-scaffolds: Bacterial removed	*Primary assembly	Anchored
Number of sequences	713	608	565	557	138	84
Total length	296,559,753	303,252,712	304,155,157	302,144,604	294,751,939	137,975,970
Max sequence length	20,165,452	28,442,430	28,458,505	28458505	28,458,505	28,458,505
Min sequence length	119	119	889	889	1,079	2,103
Mean sequence length	415,932.3	498,770.9	538,327.7	542,449.9	2,135,883.6	1,642,571.1
Median sequence length	18,659	15,738	17,238	17,226	187,754	29,360
Sequence length N50	2,878,051	9,560,626	10,085,069	10,085,069	10,085,069	11,007,303
Sequence length L50	24	12	11	11	11	5
Expected sequence length	5,050,162	9,440,067	10,090,295	10,152,132	10,405,732	12,395,557
Sequence length NG50	3,454,019	10,026,878	10,522,735	10,522,735	10,522,735	2,103
Sequence length LG50	21	11	10	10	10	84
Normalized expected sequence length	5,348,840	10,224,021	10,960,769	10,955,043	10,953,963	6,108,175

* The primary assembly contains contigs such that bacterial, ‘remove tigs’, and ‘redundant contigs’ are removed and those retained are marked as KEEP by purge_haplotigs. When keeping all of the non-bacterial KEEP scaffolds from purge_haplotigs there are 162 instead of 138. The difference is that purge_haplotigs retains 4 of the contigs originally marked for removal, which were associated-bubble contigs from Falcon (max size of 13.7 kb, mean size of 8.4 kb, sum of ~33.5 kb), and retained 20 contigs that our minimap2-alone approach marked as “redundant” (max size of 20.2 kb, mean size of 9.4 kb, and sum of ~187 kb).

Supplementary Table 8 A-C: Gap size statistics

A. Gap size statistics before and after gap-filling:

	Canu (C3.2)		Falcon (F9)	
	Before	After	Before	After
Number of gaps	187	156	105	91
Total gap length	8,761,718	7,789,542	6,692,707	5,628,294
Max gap size	677,224	674,379	965,004	953,978
Min gap size	500	25	500	25
Mean gap size	46,854.1	499,323	63,740.1	61,849.4
Median gap size	20,800	21,425	30,519	24,983
Gap N50	102,377	119,644	144,312	124,939
Gap L50	22	19	11	8
Expected gap size	167,075	176,849	288,100	314,580

B. Gap filling progress by stage for Canu (C3.2):

CANU								
Stage	Initial	Q1	PBJ1	Q2	PBJ2	PBJScaff	Q3	Pilon
Number	187	186	166	165	162	157	156	156
Total Length	8,761,718	8,761,218	7,952,958	7,952,624	7,819,479	7,789,567	7,789,542	7,789,542
N Diff	-	1	20	1	3	5	1	0
Len Diff	-	500	808,260	334	133,145	29,912	25	0

31 gaps closed totally. 972,176 bp filled overall.

C. Gap filling progress by stage for Falcon (F9):

FALCON								
Stage	Initial	Q1	PBJ1	Q2	PBJ2	PBJScaff	Q3	Pilon
Number	105	104	97	95	93	92	91	91
Total Length	6,692,707	6,692,207	5,778,078	5,777,035	5,669,089	5,628,485	5,628,294	5,628,294
N Diff	-	1	7	2	1	1	1	0
Len Diff	-	500	914,129	1,043	107,946	40,604	191	0

14 gaps closed totally. 1,064,413 bp filled overall.

Supplemental Table S9: Bacterial contig statistics in each assembly

Bacterial contig statistics	Canu	Falcon
Number of contigs	25	8
Total length	2,273,978	2,010,553
Max contig size	1,348,403	1,048,146
Min contig size	4,017	7,239
Mean contig size	90,959.12	251,319.125
Median contig size	13,445	71,061.5
Contig N50	1,348,403	1,048,146
Contig L50	1	1
Expected contig size	885,299	797,499

Supplemental Table S10: *Sciara* (*Bradysia*) *coprophila* repeat family classes from RepeatModeler

Class	From Canu	From Falcon
**Unclassified**	2267	2254
SINEs	15	19
LINEs	186	160
LTR	53	48
DNA elements	131	130
rRNA	2	2
Satellites	4	5
Simple Repeats	22	25
RC	14	16
Buffer	1	1
snRNA	0	1
**TOTAL**	**2695**	**2661**

Supplemental Table S11: Sub-classification of classified Repeat Families found in *Sciara coprophila* genome with RepeatModeler

Class	Sub-class	Canu	Falcon
**LINE**	CRE	36	30
**LINE**	I-Jockey	31	23
**LINE**	CR1	26	25
**DNA**	hAT-Ac	25	22
**Simple repeat**	-	22	25
**LINE**	RTE-BovB	22	12
**LINE**	L2	20	18
**LTR**	Gypsy	19	21
**LINE**	R1-LOA	19	10
**DNA**	Zator	16	17
**LINE**	Penelope	15	16
**DNA**	-	15	15
**DNA**	Crypton-I	15	12
**LTR**	Pao	14	11
**RC**	Helitron	13	16
**LTR**	Copia	12	13
**DNA**	CMC-EnSpm	9	9
**DNA**	hAT	7	3
**DNA**	MULE-MuDR	6	10
**DNA**	P	6	1
**Satellite**	-	4	5
**SINE**	tRNA-V	4	5
**DNA**	hAT-Tag1	4	3
**LINE**	R1	4	3
**SINE?**	-	4	2
**LINE**	RTE-RTE	3	6
**LINE**	R2	3	5
**DNA**	Maverick	3	4
**DNA**	hAT-Tip100	3	3
**LTR**	-	3	2
**SINE**	tRNA	2	6
**DNA**	hAT-Blackjack	2	5
**DNA**	hAT-hATx	2	3
**DNA**	CMC-Chapaev	2	2
**rRNA**	-	2	2
**DNA**	Kolobok-Hydra	2	1
**LTR**	ERV4	2	1
**SINE**	tRNA-RTE	2	1
**LTR**	ERV1	2	0
**DNA**	PIF-Harbinger	1	3

**LINE**	-	1	3
**DNA**	MULE	1	2
**DNA**	Sola-2	1	2
**DNA**	TcMar-Tc4	1	2
**LINE**	I	1	2
**LINE**	L1-Tx1	1	2
**SINE**	5S-Deu	1	2
**buffer**	-	1	1
**DNA**	Crypton-H	1	1
**DNA**	Crypton-V	1	1
**DNA**	Ginger-2	1	1
**DNA**	PiggyBac-X	1	1
**DNA**	TcMar	1	1
**LINE**	CR1-Zenon	1	1
**LINE**	RTE-ORTE	1	1
**DNA**	Dada	1	0
**DNA**	hAT-hATm	1	0
**DNA**	hAT-hobo	1	0
**DNA**	Kolobok-T2	1	0
**DNA**	P-Fungi	1	0
**LINE**	Dualen	1	0
**LINE**	RTE	1	0
**LTR**	ERVK	1	0
**RC**	Helitron-2	1	0
**SINE**	tRNA-Deu-L2	1	0
**SINE**	tRNA-V-RTE	1	0
**DNA**	Novosib	0	2
**LINE**	L1	0	2
**SINE**	tRNA-Meta	0	2
**DNA**	hAT-hAT5	0	1
**DNA**	MULE-NOF	0	1
**DNA**	Sola-1	0	1
**DNA**	TcMar-Tc2	0	1
**LINE**	Dong-R4	0	1
**SINE**	tRNA-Deu-RTE	0	1
**snRNA**	-	0	1
**TOTAL CLASSIFIED**	-	428	407

*The Canu and Falcon columns represent the number of repeat families with the given classification found in the final Canu and Falcon assemblies respectively.

Supplemental Table S12 A-B: Repeat Masking on Canu

Supplemental Table S12A: Summary of Repeat Masking on Canu

Canu:	All Scaffolds	Primary Scaffolds
**Sequences:**	769	205
**Total Length:**	312,049,034	298,965,442
**Percent GC:**	35.56	35.56
**Bases masked:**	126,126,531	118,515,350
**Percent of assembly masked:**	40.42	39.64

Note: Tables describe masking of both primary and associated contigs. The associated sequences that were filtered out during assembly pruning were enriched for repeats containing 7,611,181 masked bases of 13,083,592 bases total (58.17% masked).

Supplemental Table S12B: RepeatMasker classifications from Canu

Class	Sub-class	Number of elements	Length occupied (bp)	Percent of assembly
**SINEs:**	-	6,607	1,244,312	0.4
	ALUs	0	0	0
	MIRs	0	0	0
**LINEs:**	-	21,636	9,813,413	3.14
	LINE1	340	55,161	0.02
	LINE2	2,394	1,168,917	0.37
	L3/CR1	2,581	1,433,910	0.46
**LTR elements:**	-	11,150	4,668,386	1.5
	ERVL	0	0	0
	ERVL-MaLRs	0	0	0
	ERV_classI	47	3,734	0.001
	ERV_classII	572	85,008	0.03
**DNA elements:**	-	5,0957	11,011,885	3.53
	hAT-Charlie	15	849	0.0003
	TcMar-Tigger	12	1,339	0.0004
**Unclassified:**	-	523,519	96,659,741	30.98
**Total interspersed repeats:**	-		123,397,737	39.54
**Small RNA:**	-	1,347	451,713	0.14
**Satellites:**	-	1,722	227,960	0.07
**Simple repeats:**	-	50,235	3,051,062	0.98
**Low complexity:**	-	9,985	460,867	0.15

*Most repeats fragmented by insertions or deletions have been counted as one element.
RepeatMasker Combined Database: Dfam_Consensus-20181026, RepBase-20181026.
Run with rmblastn version 2.2.27+.
The query was compared to classified sequences in the Full Repeat Library constructed for *Sciara coprophila*.
Note: 76.6% of masked bases were unclassified.

Supplemental Table S13 A-B: Repeat Masking on Falcon

Supplemental Table 13A: Summary of Repeat Masking on Falcon

Falcon:	All scaffolds	Primary scaffolds
**Sequences:**	565	138
**Total Length:**	304,155,157	294,751,939
**Percent GC:**	35.54	35.56
**Bases masked:**	121,347,624	116,828,462
**Percent of assembly masked:**	39.9	39.64

Note: Tables describe masking of both primary and associated contigs. The associated sequences that were filtered out during assembly pruning were enriched for repeats containing 4,519,162 masked bases of 9,403,218 bases total (48.06%).

Supplemental Table 13B: RepeatMasker classifications from Falcon

Class	Sub-class	Number of elements	Length occupied (bp)	Percent of assembly
**SINEs:**	-	6,565	1,239,700	0.41
	ALUs	0	0	0
	MIRs	0	0	0
**LINEs:**	-	21,297	9,424,389	3.1
	LINE1	338	55,260	0.02
	LINE2	2,389	1,160,831	0.38
	L3/CR1	2,556	1,401,689	0.46
**LTR elements:**	-	10,976	4,379,978	1.44
	ERVL	0	0	0
	ERVL-MaLRs	0	0	0
	ERV_classI	45	3,474	0.001
	ERV_classII	563	84,866	0.03
**DNA elements:**	-	50,340	10,123,256	3.33
	hAT-Charlie	13	717	0.0002
	TcMar-Tigger	12	1,325	0.0004
**Unclassified:**	-	520,383	93,317,154	30.68
**Total interspersed repeats:**	-	-	118,484,477	38.96
**Small RNA:**	-	1,372	666,998	0.22
**Satellites:**	-	1,733	228,935	0.08
**Simple repeats:**	-	49,811	2,930,470	0.96
**Low complexity:**	-	10,008	460,501	0.15

*Most repeats fragmented by insertions or deletions have been counted as one element.
RepeatMasker Combined Database: Dfam_Consensus-20181026, RepBase-20181026.
Run with rmblastn version 2.2.27+.
The query was compared to classified sequences in the Full Repeat Library constructed for *Sciara coprophila*.
Note: 76.9% of masked bases were unclassified.

Supplemental Table S14: Transcriptome Evaluations

Assembler	StringTie						Trinity
**Reference genome**	**Canu**			**Falcon**			N/A
**Approach**	1	2	**3**	1	2	**3**	***De novo***
**Number Genes**	19248	22520	**22875**	19308	22574	**22958**	**75585**
**Median NTPG***	1	1	**1**	1	1	**1**	**1**
**Max NTPG**	95	54	**137**	96	59	**148**	**22**
**Number Transcripts**	36469	34167	**44987**	36478	34307	**45099**	**93211**
**Median TL**	2081	1721	**2118**	2081	1715	**2118**	**493**
**Max TL**	64293	62815	**64287**	59877	59879	**59897**	**33554**
**BUSCO: C**	2679	2693	**2705**	2677	2693	**2703**	**2694**
**BUSCO: F**	45	41	**40**	44	38	**38**	**53**
**BUSCO: M**	75	65	**54**	78	68	**58**	**52**
**TransRate pRef w/ CRBB (UniProt)**	0.24944	0.25016	**0.25234**	0.24952	0.25089	**0.2521**	**0.26451**
**TransRate Reference Coverage (UniProt/ Arthopod)**	0.36455	0.36275	**0.36898**	0.36434	0.3622	**0.36819**	**0.37041**
**TransRate pRef w/ CRBB (all)**	0.0883	0.08576	**0.0919**	0.08781	0.08584	**0.09188**	**0.09598**
**TransRate Reference Coverage (all)**	0.07644	0.07122	**0.07982**	0.07609	0.07164	**0.07986**	**0.07322**
**TransRate Score**	0.05376	0.08375	**0.0368**	0.05247	0.08278	**0.03599**	**0.08182**
**TransRate Optimal Score**	0.2522	0.28717	**0.21469**	0.25012	0.28662	**0.21232**	**0.29233**
**RSEM-Eval Score (x10^9)**	-5.83677	-5.95996	**-5.73757**	-5.84391	-5.94631	**-5.75274**	**-7.02515**

*NTPG Number Transcripts Per Gene; TL = transcript length

Supplemental Table S14: Transcriptome Evaluations

This table contains results summarized in Figure 5F “Reference-guided transcriptomes” category in the bold columns under Canu and Falcon (Approach 3, emboldened). There were three reference-guided transcriptome assembly approaches: (1) independent assemblies of each RNA-seq sample, followed by assembly merging, (2) read merging, followed by assembly, and (3) assembly merging the independent assemblies with the merged read assembly. The order from least to most sensitive for both assemblies was approach 1, then 2, then 3. This is unsurprising since approach 2 effectively has higher read depth for the assembly step, and since approach 3 merges outputs of approaches 1 and 2. For example, approach 3 had the highest number of complete BUSCOs detected and the fewest missing. Similarly, in the TransRate analysis, approach 3 had the highest proportion of reference proteins with Conditional Reciprocal Best BLAST (CRBB) hits and highest coverage across the reference proteins. Approach 3 also received the best RSEM-Eval scores, but did not fare as well as the other approaches with TransRate scores. Nevertheless, since approach 3 seemed to fare best in most metrics, we chose to include it as part of the EST evidence in the Maker2 gene annotation steps below.

Supplemental Table S15 A-B: Maker Annotation Transcript Evaluations on Canu and Falcon

Supplemental Table S15A: Canu Maker Annotation Transcript Evaluations

	Canu Annotation Rounds					Alternative Round 3			Final Canu Annotation
Round	1	2	3			Alternative Round 3			3*
Repeat Library	Original	Original	Original			Alternative/Filtered			Both*
KeepPred =	0	0	1			1			1
Set	NA	NA	Default	Standard	Max	Default	Standard	Max	Standard*
# genes	15156	18250	21388	22330	33044	18845	21566	35492	23117
# transcripts	22128	22866	27003	27954	38713	25250	28007	42120	28870
Median NTPG	1	1	1	1	1	1	1	1	1
Max NTPG	19	19	19	19	19	16	16	16	19
Median NEPG	5	4	4	4	4	4	4	4	4
Max NEPG	75	131	132	132	132	129	129	129	132
Min GL	18	69	69	69	69	69	69	69	69
Median GL	2524	2677.5	2682	2660.5	2720	2912	2809	2698.5	2630
Max GL	406075	734151	545840	545840	545840	916260	916260	916260	545840
Median TL	1647	1553	1398	1383	900	1371	1332	761	1330.5
Max TL	31218	122902	542627	542627	542627	161972	161972	161972	542627
Median EL	212	192	185	185	158	173	173	147	182
Max EL	13073	122229	541964	541964	541964	161911	161911	161911	541964
Median IL	71	75	79	80	137	88	88	177	80
Max IL	218817	302637	218817	218817	273521	542594	542594	542594	218817
Median 5L	157	159	165	165	165	169	169	169	165
Median 3L	172	184	184	184	184	192	192	192	184
NG 5’ and 3’	12583	10131	10762	10762	10766	8100	8100	8103	10801
NG 5’ OR 3’	14315	12840	13193	13198	13213	10159	10175	10195	13335
BUSCO: C	2511	2601	2611	2638	2641	2265	2531	2562	2638
BUSCO: F	77	74	77	77	77	79	108	112	78
BUSCO: M	211	124	111	84	81	455	160	125	83
BUSCO: % Found	92.46	95.57	96.03	96.0	97.11	83.74	94.28	95.53	97.03
BUSCO: C (proteins)	2504	2585	2593	2620	2623	2248	2514	2544	2620
BUSCO: F (proteins)	81	74	78	78	78	81	111	115	79
BUSCO: M (proteins)	214	140	128	101	98	470	174	140	100
BUSCO: % Found (proteins)	92.35	95.0	95.43	96.39	96.50	83.21	93.78	95.0	96.43
TransRate pRef (UniProt)	0.23549	0.24017	0.24154	0.24396	0.24718	0.22034	0.24057	0.24573	0.2446
TransRate RefCov (UniProt)	0.34797	0.34658	0.34779	0.34993	0.35085	0.32425	0.34383	0.346	0.3503
TransRate pRef (all)	0.07887	0.07725	0.08012	0.08125	0.0851	0.07107	0.07846	0.08412	0.0816
TransRate RefCov (all)	0.06624	0.06359	0.0649	0.06543	0.06587	0.05922	0.06315	0.06425	0.06555
TransRate Score	0.13249	0.12217	0.092	0.08706	0.02364	0.06299	0.06603	0.03388	0.03659
TransRate Optimal Score	0.39399	0.38755	0.41209	0.4118	0.28501	0.34404	0.35499	0.36033	0.28293
RSEM-Eval Score ($x10^9$)	-14.1476	-12.8598	-12.5512	-12.3797	-12.3468	-16.4623	-14.9077	-14.5409	-12.318

*See caption for S15A,B tables for a key to help interpretation of abbreviated metric names.
Note: The final Canu annotation here is Bcop_v1 that was deposited to the i5K workspace.

Supplemental Table 15B: Falcon Maker Annotation Transcript Evaluations

	Falcon Annotation Rounds					Alternative Round 3			Final Falcon Annotation
**Round**	1	2	3			Alternative Round 3			3*
**Repeat Library**	Original	Original	Original			Alternative/Filtered			Both*
**KeepPred =**	0	0	1			1			1
**Set**	NA	NA	Default	Standard	Max	Default	Standard	Max	Standard*
**# genes**	15084	18060	19856	20596	26435	16452	19179	27473	21010
**# transcripts**	22028	22523	24036	24786	30653	20327	23088	31489	25225
**Median NTPG**	1	1	1	1	1	1	1	1	1
**Max NTPG**	19	15	14	14	14	16	16	16	14
**Median NEPG**	5	4	5	5	4	5	5	5	4
**Max NEPG**	63	124	159	159	159	242	242	242	159
**Min GL**	18	100	100	100	100	72	72	72	72
**Median GL**	2542	2770	2988	2982	3462	3441.5	3502	3981	2962.5
**Max GL**	406075	748256	548033	548033	548033	844972	844972	844972	548033
**Median TL**	1653	1560	1491	1475	1202	1506	1471	1089	1447
**Max TL**	31218	146821	543194	543194	543194	99085	99085	99085	543194
**Median EL**	212	190	181	180	159	172	170	145	179
**Max EL**	13073	146569	542688	542688	542688	98842	98842	98842	542688
**Median IL**	71	76	83	85	146	90	98	198	84
**Max IL**	223782	302656	255207	255207	255207	510038	510038	510038	255207
**Median 5L**	157	159	160	160	160	164	164	163	160
**Median 3L**	172	185	185	185	185	197	197	197	185
**NG 5' and 3'**	12549	10104	10083	10083	10084	7179	7179	7182	10119
**NG 5' OR 3'**	14262	12827	12895	12899	12912	9429	9438	9465	12971
**BUSCO: C**	2506	2584	2599	2630	2633	2176	2503	2543	2631
**BUSCO: F**	79	80	75	75	75	83	122	124	75
**BUSCO: M**	214	135	125	94	91	540	174	132	93
**BUSCO: % Found**	92.35	95.18	95.53	96.64	96.75	80.71	93.78	95.28	96.68
**BUSCO: C (proteins)**	2497	2565	2580	2611	2614	2161	2485	2526	2612
**BUSCO: F (proteins)**	85	83	79	79	79	87	127	128	79
**BUSCO: M (proteins)**	217	151	140	109	106	551	187	145	108
**BUSCO: % Found (proteins)**	92.25	94.61	95.0	96.11	96.21	80.3	93.32	94.82	96.14
**TransRate pRef (UniProt)**	0.23493	0.23864	0.24122	0.24371	0.24637	0.21236	0.23751	0.24307	0.24396
**TransRate RefCov (UniProt)**	0.34764	0.34576	0.34671	0.34864	0.34931	0.31321	0.33969	0.34237	0.34875
**TransRate pRef (all)**	0.07854	0.07728	0.07853	0.07959	0.08252	0.06678	0.07543	0.08035	0.07979
**TransRate RefCov (all)**	0.06596	0.06359	0.06359	0.06411	0.06451	0.05674	0.06166	0.06275	0.06421
**TransRate Score**	0.13294	0.12285	0.10809	0.10314	0.06423	0.08193	0.08601	0.04975	0.09869
**TransRate Optimal Score**	0.39056	0.39052	0.39057	0.39166	0.39179	0.32995	0.33836	0.34169	0.39171
**RSEM-Eval Score ($x10^9$)**	- 14.2099	-13.0089	-12.9233	-12.7318	-12.6894	-17.1623	-15.3031	-14.9171	-12.6757

*See caption for S15A,B tables for a key to help interpretation of abbreviated metric names.

Supp Table 15 A and B: Canu and Falcon Maker2 Annotation Transcript Evaluations
These tables contain results summarized in Figure 5F "Maker transcriptomes" category (Final Canu and Falcon annotation columns in A and B, respectively) as well as additional statistics. We evaluated the transcript outputs of the Maker2 annotation process. For both genome assemblies, the gene annotation improved across subsequent rounds. For example, there were more complete BUSCOs and fewer missing BUSCOs in each subsequent round of Maker. Similarly, a Transrate completeness metric was highest after the third round. RSEM-eval scores improved across Maker rounds, and for the Canu assembly, the TransRate Optimal score was highest after the third round. We also saw that the annotation edit distance (AED) scores from Maker2 reflected an improvement in round 3 over round 2 (not shown).

The transcript models learned from the Canu assembly contained more complete BUSCOs, fewer missing BUSCOs, more reference proteins with CRBB hits and higher reference protein coverage, higher TransRate optimal scores, and higher RSEM-Eval scores than those learned from Falcon. We also saw that in the final annotation sets from the Canu and Falcon assemblies there were more Canu models with the lowest annotation edit distances than Falcon models (Supplemental Figure S14). Moreover, the Canu annotation had more functionally-annotated genes (Supplemental Figure S14, Supplemental Table S16). Overall, this suggested Canu had a superior annotation.

Interestingly, the final set of Maker annotations for both assemblies had higher Transrate scores and higher Transrate optimal scores than the input transcriptome assemblies. In contrast, they had lower RSEM-Eval scores and lower completeness scores from both BUSCO and Transrate than the input transcriptomes. The final transcript models from Maker contained 94.2% and 94.0% complete Dipteran BUSCOs for Canu and Falcon, respectively (more when considering fragmented BUSCOs). For comparison, ~93.7% complete Dipteran BUSCOs were detected in both genome assemblies directly and ~96.6% were detected in StringTie transcriptome assemblies.

Key:
NTPG = Number Transcripts Per Gene;
NEPG = Number Exons Per Gene;
GL = Gene Length;
TL = Transcript Length;
EL = Exon Length;
IL = Intron Length;
5L = 5' UTR Length;
3L = 3' UTR Length;
NG 5' and 3' = Number of Genes that have both 5' and 3' UTRs annotated;
NG 5' or 3' = Number of Genes that have 5' and/or 3' UTRs annotated.
BUSCO C, F, M = Complete, Fragmented, Missing.
BUSCO: % Found = 100 * (C+F)/2799 = percent of Dipteran BUSCOs found as complete or fragmented.
TransRate pRef (reference used) = proportion of reference with CRBB.
TransRate RefCov (reference used) = Reference Coverage.
TransRate references used = UniProt (Arthropod proteins from UniProt/SwissProt); All (All proteins gathered for use in Maker2 annotation).

Supplemental Table S16: Additional characterization and comparisons of the final annotations of Canu and Falcon assemblies.

	Final Canu Annotation	Final Falcon Annotation
Number of Genes with Ontology Term	8671	8324
Number of Genes with UniProt hit(s)	13745	13283
Number of Genes with Pfam domain	13789	13248
Number of Genes with All 3 above (intersect)	8252	7933
Number of Genes with $\geq$ 1 of 3 above (union)	14961	14406
Number of Genes with Drosophila hit(s)	9739	9547
Number of Genes with Anopheles hit(s)	9057	8939
Percent Drosophila Proteome with *Sciara* hit(s)	87.3184	87.0166
Percent Anopheles Proteome with *Sciara* hit(s)	89.3487	89.3152

These are the results summarized in Figure 5F “Functional Annotations” category. The Maker2 annotation for the Canu assembly had more genes with GO terms, more genes with UniProt hits, and more genes with recognizable Pfam domains than found in the Maker2 Falcon annotation. The Canu annotation was also judged to be more complete by BUSCO (Supplemental Table S15), as well as through the number of hits back and forth with the proteomes of two other flies: *Drosophila melanogaster* and *Anopheles gambiae*. As with results reported elsewhere (e.g. Supp. Table S15), this suggests the Canu scaffolds had the superior annotation.

Supplemental Table S17A: Putative Sciara homologs for proteins involved in reading, writing, and erasing DNA methylation marks for adenine and cytosine

				Analysis of putative Sciara homolog								
**Protein**	**Protein length (AA)**	**Possible Function**	**Putative Sciara homolog**	**Sciara protein length (AA)**	**Pfam domain**	**Inter Pro Annotation**	**GO term(s)**	**UniProt-SwissProt Functional Annotation: "Similar to:"**	**Drosophila melanogaster Top Hit**	**Homo sapiens Top Hit**	**Other top hit(s) [species]**	**Other Sciara proteins of interest**
Dnmt2	329-369	cytosine methylation	Bcop_v1_g020988-RA	336	C-5 cytosine-specific DNA methylase	C-5 cytosine methyltransferase	0008168	TRDMT1 tRNA (cytosine(38)-C(5))-methyltransferase (Homo sapiens OX=9606)	methyltransferase 2, isoform C	Chain A, DNA CYTOSINE METHYLTRANSFERASE DNMT2	.	.
TET1/2/3 and DMAD (most like DMAD and TET2)	2136, 2002, 1811, 2860	cytosine demethylation; adenine demethylation	Bcop_v1_g016381-RA	1653	Oxygenase domain of the 2OGFeDO superfamily	2OGFeDO, oxygenase domain	.	TET2 Methylcytosine dioxygenase TET2 (Homo sapiens OX=9606)	Ten-Eleven translocation (TET) family protein, isoform D (also called: DNA N6-methyl adenine demethylase (DMAD) and Tet2)	Chain A, Methylcytosine Dioxygenase Tet2	.	.
TET1/2/3 and DMAD (runner up)	2136, 2002, 1811, 2860	cytosine demethylation; adenine demethylation	Bcop_v1_g016384-RA/RB	337-469	CXXC zinc finger domain	Zinc finger, CXXC-type	0003677, 0008270	Tet DNA N6-methyl adenine demethylase (Drosophila melanogaster OX=7227)	Ten-Eleven translocation (TET) family protein, isoform A	Chain C, Methylcytosine dioxygenase (TET3)	TET1 splice variant VP_DE4 [H. sapiens], zf-CpG_bind_C domain-containing protein [Caenorhabditis elegans], methylcytosine dioxygenase TET3 isoform X2 [Mus musculus]	.
TDG	410	cytosine demethylation	Bcop_v1_g006678-RA	1148	Uracil DNA glycosylase superfamily	Uracil-DNA glycosylase-like	.	TDG G/T mismatch-specific thymine DNA glycosylase (Homo sapiens OX=9606)	G/T mismatch-specific thymine DNA glycosylase (AAD33588.1)	G/T mismatch-specific thymine DNA glycosylase (AAC50540.1)	.	.
MBD2	302-411	methylated cytosine binding	Bcop_v1_g000093-RA	185	C-terminal domain of methyl-CpG binding protein 2 and 3; p55-binding region of Methyl-CpG-binding domain proteins MBD	Methyl-CpG-binding domain protein 2/3, p55-binding region; Methyl-CpG binding protein 2/3, C-terminal domain	.	Mbd2 Methyl-CpG-binding domain protein 2 (Mus musculus OX=10090)	Methyl-CpG binding domain protein-like, isoform B	methyl-CpG-binding domain protein 2 isoform 1	.	Bcop_v1_g003486-RA, Bcop_v1_g003486-RB
MBD5	1494-1727	methylated cytosine binding	Bcop_v1_g017396-RD/RE/RH/RI	1394-1557	PWWP domain	PWWP domain	.	Mbd5 Methyl-CpG-binding domain protein 5 (Mus musculus OX=10090)	six-banded, isoform H (Methyl-CpG binding domain, PWWP domain)	methyl-CpG-binding domain protein 5 isoform X1 (and PWWP domain)	.	.
MBD-R2 [D. melanogaster]	1081-1169	methylated cytosine binding	Bcop_v1_g019755-RA	1193	Jumonji domain-containing protein 2A Tudor domain; Methyl-CpG binding domain; THAP domain	Methyl-CpG DNA binding; THAP-type zinc finger	0003677, 0005634; 0003676	PHF20L1 PHD finger protein 20-like protein 1 (Homo sapiens OX=9606)	MBD-R2	PHD finger protein 20-like protein 1 isoform 4	.	.
CFP1	660	Unmethylated CpG binding and recruitment of methylases	Bcop_v1_g003348-RA	477	PHD-finger;CpG binding protein zinc finger C terminal domain	Zinc finger, PHD-finger;CpG binding protein, C-terminal	.	Cfp1 CXXC-type zinc finger protein 1 (Drosophila melanogaster OX=7227)	CXXC finger protein 1, isoform B	CXXC-type zinc finger protein 1 isoform 1	.	.
SETDB1	397-1292	methylated cytosine binding	Bcop_v1_g019039-RA	1325	Histone methyltransferase Tudor domain; Methyl-CpG binding domain; SET domain; Histone methyltransferase Tudor domain 1	Methyl-CpG DNA binding; SET domain	0003677, 0005634; 0005515	egg Histone-lysine N-methyltransferase eggless (Drosophila pseudoobscura pseudoobscura OX=46245)	eggless	histone-lysine N-methyltransferase SETDB1 isoform 4	.	Bcop_v1_g019996-RA
SETDB2*	707-719	methylated cytosine binding	[Bcop_v1_g019996-RA]*	1152	Pre-SET motif; Histone methyltransferase Tudor domain; Methyl-CpG binding domain; SET domain; Histone methyltransferase Tudor domain 1	Methyl-CpG DNA binding; Pre-SET domain; SET domain	0003677, 0005634; 0005634, 0008270, 0018024, 0034968; 0005515	egg Histone-lysine N-methyltransferase eggless (Drosophila pseudoobscura pseudoobscura OX=46245)	eggless	histone-lysine N-methyltransferase SETDB1 isoform 4	.	Bcop_v1_g019039-RA
BAZ2B	2132-2168	methylated cytosine binding	Bcop_v1_g011218-RA/RB	2486-2499	DDT domain; Bromodomain; Methyl-CpG binding domain; PHD-finger; Williams-Beuren syndrome DDT (WSD), D-TOX E motif	Methyl-CpG DNA binding; Zinc finger, PHD-finger; DDT domain; Bromodomain; WHIM2 domain	0003677, 0005634; 0005515	BAZ2B Bromodomain adjacent to zinc finger domain protein 2B (Homo sapiens OX=9606)	toutatis, isoform F	bromodomain adjacent to zinc finger domain protein 2B isoform c (BAZ2B)	.	Bcop_v1_g015386-RA, Bcop_v1_g004566-RB
DAMT-1 and METTL4	365, 419	adenine methylation	Bcop_v1_g005400-RA (DAMT-1/METTL4)	367	MT-A70	MT-A70-like	.	METTL4 Methyltransferase-like protein 4 (Homo sapiens OX=9606)	CG14906	N(6)-adenine-specific DNA methyltransferase METTL4 isoform X2	DNA N6-methyl methyltransferase [Caenorhabditis elegans]	.
N6AMT1	186-214	adenine methylation	Bcop_v1_g003680-RA	233	Methyltransferase small domain	Methyltransferase small domain	0008168	N6AMT1 Methyltransferase N6AMT1 (Homo sapiens OX=9606)	HemK methyltransferase 2 (N6AMT1)	Chain A, Methyltransferase N6AMT1	.	Bcop_v1_g003124-RA/RB
ALKBH1	389	adenine demethylation (6mA)	Bcop_v1_g006515-RA	639	2OG-Fe(II) oxygenase superfamily	Alpha-ketoglutarate-dependent dioxygenase AlkB-like	.	Alkbh1 Nucleic acid dioxygenase ALKBH1 (Mus musculus OX=10090)	AlkB, isoform B	ALKBH1	ALKBH1-like hits [Insecta]	.
ALKBH2	157-261	adenine demethylation (1mA, 3mA)	Bcop_v1_g001386-RA	230	2OG-Fe(II) oxygenase superfamily	Alpha-ketoglutarate-dependent dioxygenase AlkB-like	.	Alkbh2 DNA oxidative demethylase ALKBH2 (Mus musculus OX=10090)	*No significant hits.	DNA oxidative demethylase ALKBH2 isoform 1	ALKBH2-like hits [Insecta]	.
ALKBH6	161-266	adenine demethylation	Bcop_v1_g018977-RA	241	2OG-Fe(II) oxygenase superfamily	Alpha-ketoglutarate-dependent dioxygenase AlkB-like	.	alkbh6 Alpha-ketoglutarate-dependent dioxygenase alkB homolog 6 (Danio rerio OX=7955)	CG6144, isoform B	alpha-ketoglutarate-dependent dioxygenase alkB homolog 6 isoform 3	.	Bcop_v1_g000722-RA
jumu/jumeau	719	methylated adenine binding	Bcop_v1_g008508-RA/Bcop_v1_g008508-RC	540-583	Forkhead domain	Forkhead domain	0003700, 0006355, 0043565	foxn4 Forkhead box protein N4 (Danio rerio OX=7955)	jumeau	forkhead box protein N1	.	Bcop_v1_g008508-RD
ALKBH8	614-664	RNA methylation	Bcop_v1_g000722-RA	602	2OG-Fe(II) oxygenase superfamily; Methyltransferase domain	Alpha-ketoglutarate-dependent dioxygenase AlkB-like;Methyltransferase type 11	0008168	alkbh8 Alkylated DNA repair protein alkB homolog 8 (Xenopus tropicalis OX=8364)	CG17807	alkylated DNA repair protein alkB homolog 8 isoform 1	Fe2OG dioxygenase domain-containing protein [Caenorhabditis elegans]	Bcop_v1_g000775-RA
METTL3	221-580	adenine methylation (RNA)	Bcop_v1_g006197-RA	599	MT-A70	MT-A70-like	.	Mettl3 N6-adenosine-methyltransferase MT-A70-like protein (Drosophila melanogaster OX=7227)	methyltransferase like 3	N6-adenosine-methyltransferase catalytic subunit (METTL3)	.	.
METTL14	456	adenine methylation (RNA)	Bcop_v1_g016925-RA	392	MT-A70	MT-A70-like	.	Mettl14 N6-adenosine-methyltransferase non-catalytic subunit (Drosophila melanogaster OX=7227)	methyltransferase like 14	Chain B, N6-adenosine methyltransferase subunit METTL14	.	.

[Bcop_v1_g019996-RA]* may be another isoform of SETDB1 or SETDB2. Sequences for Sciara homologs reported below and available in the Bcop_v1 annotation at i5k.

Supplemental Table S17B: Other methylation-related proteins

Protein	Length	Putative Function	Best Sciara Hit	Putative homolog for:
MECP2	486-498	metylated cytosine binding	Bcop_v1_g019755-RA	MBD-R2
MBD3	259-291	methylated cytosine binding	Bcop_v1_g000093-RA	MBD2
MBD4	262-580	methylated cytosine binding, cytosine demethylation	Bcop_v1_g019755-RA	MBD-R2
MBD6	1003	methylated cytosine binding	Bcop_v1_g017396-RD/RE	MBD5
MB3L1	194	methylated cytosine binding	Bcop_v1_g000093-RA	MBD2
NMAD-1	208-291	adenine demethylation	Bcop_v1_g000722-RA	ALKBH8
ALKBH3	614-664	adenine/cytosine demethylation (1mA, 3mC)	Bcop_v1_g001386-RA	ALKBH2
BAZ2A	1873-1905	methylated cytosine binding	Bcop_v1_g011218-RA/RB	BAZ2B

These proteins had significant hits to Sciara proteins, but the Sciara proteins were best reciprocal hits with and putative homologs for the proteins named in column 5. The significant hits were likely due to similar methyl-binding domains and other shared domains.

More information on the methylation-related proteins in Tables S17A and S17B can be found in recent reviews (Armstrong et al. 2019; Long et al. 2013; Rausch et al. 2020; Kohli and Zhang 2013; Iyer et al. 2016; Zhu et al. 2018).

Supplemental Table S17C: FASTA sequences of Sciara proteins that were putative homologs

>Bcop_v1_g005400-RA, DAMT-1-like, METTL4-like
MSVVYSEKDCVLINHKEYIDHIYRTTIVNQGDSGSDLKTIYTRLEDVFNFAPKNRNDTDDTKRHKINAKKSSILISQNPELHELNQTIAAKSKEFLEDCKRRNVIFNESSHHTTDSSLSVFKEVCSEET
VNVPLNGKNTLDNAIVTKISGQTYLIPPKCKFTNTCVSAIDKFADEFYDFIVIDPPWWNKYIRRVRTWKREEGYDMMYNDDILKIPLEDLIHPNTIVAIWCTNSPTHSEFVKKIALPKWNLKLLATYYW
VKITKSGDPICEFNAPLKKQPYEQLFLATHIESERLLKDDINLIFSISSVVHSHKPPLLEVFKDYLPDQPKCLEIFARYLLPHFSSIGREVLKLQNTILFEEKEQETVR
>Bcop_v1_g006197-RA, METTL3-like
MSDGTWDQIQEIKIKRNSLREKLEKRKKERQDILLGSTNLAAASSFIKSESSGSEDKKPVLSNIKHEIDADPEVERQLLQILSDNALTLPISSKQLAQRMNLLRFKPVNQDILYYFLQKLVAQSHISIN
NINSSTESGYEVTLVDHSRVQSLFNDIVEETEATVKSEDILKRKLDDDSDEGSGSKFSKNLSNTDKKVSLTKDTSNNNSDIMSLLSMPSTREKHNKQVGEEILELLSKPTAKERSLAEKFKSQGGAQVM
EFCPHGTRIECLRAQQATEELQSVKKADDQTVLIKLEESAEPVVVDSMKCNKLHFKKIIQSHTDESLGDCSFLNTCFHMDSCKYVHYEVDTLLTSEGCKADNNKNNRCIALPKLKTIDPAAKLDPPQWI
QCDLRFLDMTVLGKFAVIMADPPWDIHMELPYGTMSDDEMRQLGVPALQDDGLIFLWVTGRAMELGRECLKLWGYERFDELIWVKTNQLQRIIRTGRTGHWLNHGKEHCLVGTKGNPQNLNRGLDCDVI
VAEVRATSHKPDEIYGIIERLSPGTRKIELFGRPHNVQPNWITLGNQLDGIRLVDPELIAQFQKRYPDGNCMAPSQVAAKPPA
>Bcop_v1_g003348-RA, CFP1-like
MFKKSKRSKEEIAKEFDLPERKSKIATILKQGEQYCLCRSSDCSRFMIGCDGCEEWYHGDCIFVSEKEAKHIKHYYCQRCKENDPSLQTVFRAVPVAAVPSNPSDDRLPKKPKEKVRESDAKEGSKSKG
RCGNCDGCSNKYNCGRCDSCRSTGKKQRCDKRICINQRVKKEKPATSRTKKRRRSPSPEITNPELEGPRQCYGPGCRMTARPQSKYCSDLCGKKLASSRIYQVLPQRIQEWNLSPCIAEEENKVQLDNI
RAKKMTVRATLAQLDERQKELDLLVERAKRCVLDPNASETNEVEDEMSYCVTCLEPIHSRTAIRHMEKCFNKYESQASFGSIFKTRIEGSSMFCDFYNPASKTYCKRLRVLCPEHCKKPKISDTDVCG
CPLVKNTFELTGEFCCAPKKKCFRHFFWEKIRRAEIDCERVRQWLKMDELMEQERQVRSSMASRAGVLHLMLHSTYNHELMEQLCRSGRQ
>Bcop_v1_g003680-RA, N6MAT1-like
MERIRFDQKGFEEYFNEVFQFIYRPDDDSKMLVDAVERLESLIFDHIKPAVCLEIGSGSGYVINSISKNFGSKIYKYYATDINPIAIETTLASGRANGVSNIHVVMTSLVDDIKKNLTHQVDLLVTNPP
FEPSPVEDVGRKGAICAWSAGKNGRAIIDRIIIELPDIMSDHGVALMCCVKENDVNDIIRRMGEQDFYSNIVIEREGTTSSNYNRIYQQYVIAFSKTNYWAEPY
>Bcop_v1_g006515-RA, ALKBH1-like
MFTDSFKYYKSKCPPPSFESVVNFSENDDNLFTRRTTIKAGEEFKHLGLTDPTHWHIYQFSSGLIFIKNPFTPHGQRYWIARCLRDYPKSPHANNLRSKSISPAAIDDWWTEMVSSRTKSDKESLRSAL
RWCTLGYHHQWDTKVYSDEMKHPFPVDLGTLSAYIARTLDFGEYSAEAAIVNFYPIGTTLAGHTDHSERNLDAPLFSFSFGQSAIFLIGGTTKDIKPTAMFLHSGDIVIMSKESRLCYHAVPRVFKSDD
SVWNNTIEIGDDDDRNEMPKKRKIENEAGYMVKDLWNDVDDRFYWEPFNEYVENCRINVNMFTDSFKYYKSKCPPPSFESVLFTRRTTIKTGEEFKHLGLTDPTHWHIYQFSSGLIFIKNPFTPHGQRY
WIARCLRDYPKSPHANNLRSKSISPAAIDDWWTEMVSSRTKSDKESLRSALRWCTLGYHHQWDTKVYSEEMKHPFPVDLGTLSAYIARTLDFGEYSAEAAIVNFYPIGTTLAGHTDHSERNFDAPLFSF
SFGQSAIFLIGGTTKDIKPTAMFLHSGDIVIMSKESRLCYHAVPRVVKSDDSVWNNTIEIGDDDDRNEMPKKRKIDNEAGYMVKDLWNDVDDRFYWEPFNEYVENCRINVNVRQVLRKGEHSL
>Bcop_v1_g006678-RA, TDG-like (TDG G/T mismatch-specific thymine DNA glycosylase)
MELSNNAMTSPMAATNVPYSPAPLQSDLSYSPDHSKLTAPSPASSQNATERPNDSLPKPNPDDQLVSNDHQQHSVRNFINHSNETENETDKSQVKFIEESQLKSTQPSPMDFQSNNQDDDNKQHELKAY
NPINKEASPTHTNHIEHDIKFDKNNEDKNVGEPYTPQYLPPPNNYNNAYVNKPNTTFKPYRMSNIPSNMKHEMSDDGYETSATGDGSHLTQNGSHMMGNQVLMPKCEISPPDDPYNFVDDDMQGSMSPH
LMPSSMQPHMRVPYSQSSLAGGGAGGNIVNNVPKLASMHGNMHAMSTVHSPVLSSPPIDGSTPKKRGRKKKQRDDTGQILNGVVKQIPAKERKKHDRFNGLSEEEVSKRTLPDHLANNLDIIIIGINPG
LFAAYKGHHYAGPGNHFWKCLYLSGLTHEQMNAEEDYKLLDFGIGFTNMVERATKGSADLTRKEIKEGCRLLQDKLRRFRPKIAVFNGKLIFEVFSGKKDFNFGRQPECIEGTNTYMWVMPSSSARCAQ
LPRAADKVPFYTALKKFRDYLNGLIPHLDESECVFSDPKFKACYEIEPKADNISIPSIQSATDITDLSNNHKMLDVPGKKKRGRPKKVRGLDGEEPKVTPRRTPSVVTLDSDGMPKKKRGRPKKVKTDA
INGMSNGMEAISNSIDAMCNTSIGMCSSVNGQNRMYPSPMQSPNPNNTFCGMNSMNPSAYGSMNSMALNPNRPNSYNHQQSPQNLHSQPFSHSDLSSEISAAISSEHLGESPVPTSPSLGPPDFEPPNN
CMPTDDNNTNECGFASPAPSTISEHTGPPLSQLHSNNNSTSYSPYRTTPSNNFIDPRSNDPNVNTTNESYALQAQQRQQQQNGLSNPMLRQTIPTNVDQHSPRHQQQQQQQQQTQQQQQQHSTTGDVY
KLPDVSAKSLSGLESLVDQIPSLNDSESPSSNNSLAGHSLNVSNLTGASVPISDSRTADVSSLSEQYQNSCIYSGYPSNLNSMPGPTSVNPSQIPQFSNSNYSPPNSSSPSNATNAYGSLSSSFSVSSL
TSSYPSSAMNTYHSNLMAASTHHLGGTGAPMYMEPHIPMAVNPLYHPYSQHASYTGYSPIAPPPTIHMPSPNYPYGYTNSTYGQPAHPTTHPAAGYLSNHHSMFDRIKPDIGYGGF
>Bcop_v1_g019755-RA, MBD-R2-like
MAPRRCTIKGCPSVSGRQQHRGVTFHTFPLNPIARQFWLTNCNLPDTKNITKSILVCSRHFRRADFQPLKNNKYLLKQGAVPTIFPWGTLPYVEPTPHSVIESGEPMVIADDKPAEPLPLVNQQLVDEI
VKIKSEMKATIGVKSPGKRSASVDEPGTSEPKKKAIRVSLDSKKLSNEPIATASFDPFSKFSPGARIEAQDFNGAWHTAKVVEVDGDEKEVLINFEKNSKSKAPATATEWIPMNSSRLRQVVKKPIASY
VPGEKCLARWTDSRKFPATIQKVLENNTYEVLFDDGFVKVVRAGHISKVKVPEPKTPDKSGRKSNPHLPIPKFDLAKNLLDVEPIGAEGYLDGPDGHKRPTVLVEDWRLPAGW
TKHLYQRSSVSGKWDVVLVGPTKKRFRSKNDVKVYLEEKGEVYNPDIYDFSIHKRRAKDIGLFVFTDEYKEELKNKQLLAASTAATAASSITPLLLNTSDDAFQGFSSKESSFLSSTLPSTPLSSIGSL
NASAIPSTPQSIPLLQPHPPPPPLPPTDPGYVYVGALKVQLIDNLFHCPEATCNKNFRKENHLQIHVKHYHLDLAKLLGVCPNMSDLAYQRTVGMPIDEAVPKNQIPNSQFFEKVYQSDLSSKLQRKSM
SPSTQVKAEPRSPNFSEKAANISAHSDEVDKPVAEPIAQSQEIANILHQSLSNEKNVEQIIHAQECIGSPKKPEAMPLDADVAIVKKSETPTARTKATKRRPIKPLNRQKARRNIKKAFMKSLQEAKAS
IVKDFGDANEGRVPLSSVSELSPTKSSRLKFLQPVDIGSSKTIAHDRQHVPEMNAPHPKYINENGEVIKIVRMRQEEIINCLCSYVEEDGLMIQCELCLCWQHGICNGIEKESQVPEKYICYICRNPQR
GRESMKYAHDQDWLLAGKLPVANYHPANSKHTERFDMLKQSHTLTGNLIELKRFMHSLNVKINIAENKDHPKMYLWSKKWEKCPTTAELTGIEMLAQSDKSKFVTNEALNELFSNTEVKKDAVKEEELN
TPMEVAADTSDPNEDKTATVPVKEEEAALTEEKPNVFGDDKMIVDQSILAGLLNSSGGVNVEQTQETVAVKIPQPEAAINPSECQLLLLDHIQKQQNLVMGRLQTIEAQIIALECRDDKMDIMEQPESC
SKTKQIISLLLNDLQKMRKIAAINSMENKLEI
>Bcop_v1_g019996-RA, SETDB1-like, SETDB2-like
MEHCEQTSEIMDIEKNAKLSKIIEDNSASNHSNRSDGWSDAITGGHHDVVSLLSDNEGEVMNSNGSADDNAESMGYGLNNEATWTLVEPTIVQRDSNDSDSGIVMPDNSVVNFREQSAERTQNSQKQEA
FNINCTDLQPNCESVTSNILNSLASNEFQHDAGSADDKTTRKKCINSECSSDKKKTTFYDTPIWSLNYFNVPRNYSRRQSVCQRCFDVSINDYERLGAALVNQQPLMLERLPIRPEVVEIIDSEEEDKG
GVGDTEALPSNTLTLLEENVDVVLQETVNRFGIEQQMRWANQILEHRVDVNSAISDRIDEEIRSMDQLRDSMYKNLYSRSRYAIEELPPFDLNTNKRLHMFGPNYPPCGKVVYPPVDKNSFYYAVRTKL
LSTWMPCKVTEYGDGPVDGKPMMFTVQFLRRNDDIKTVPGNHLSFVRVLRVNGTRVIALTMEKSVNVKCLNFHPGIVGETLQEYNRWRYMIFFDDGCVRYVTPDNVRLICSPSPNVWEDVSHDAVAA
AFIKSYLEQYKTERPMVQVKPGQKMLTQWRSEWKSARVANVDASLVHMHFEDVKRWEWIYRGSTRLGPLFKDNHQRQRRFAIAAKGNESFVELVSSDDDDESKNCRETTSPTVKSEDSSVPNTFTIPSQ
VDPERKQENRAIAKKGTGTMPSHPTVQHMNKATIYIDEDSPAKGKVVHYTVKQRILPRKFVSHICGTACLYPVKYNLSSYSPLVKPLLSGWDRKSVMNTRTSRRIVYQTPCGRIMRNMQELHRYLRDTK
CTSLDVDNFDFGGMTSCLAEYLVDNSFIQTKDLSNGCEAMTVSSVNYFDNTTPPPCEYSAQRIPTDGVPLNLDPDFLCGCDCDDDCWDKTKCQCWKLTLEGAKLISPYIPIDRMGYKYKRLLDPIPTGV
YECNAQCKCSKMCLNRVAQHPLQLKLQVFKTKSRGWGLRCLNDVPEGSFVCVYAGDLLTDKSANIAGGEYGDEYFAELDFIEVIESLKEGYEPEAMDVDSGRNSVHVSSETSIDVSKSLKVEPVFNTDD
DEIQVLNEPSLDENASSVCLLRSNSTLRFYDKNEMVFTMDAKKHGNIGRYINHSCDPNLFVQNVFVDTHDLRFPWVAFFAKRYIAAGTELTWDYNYVVGSVPGKRLFCECGAQNCRRRIL
>Bcop_v1_g008508-RA, jumu/jumeau-like
MNQFEEISTGFHDIFLNKFADSSGPILDFYVSDSMQDMLDIDIRSEVATVVGGVSEFTSLMNDLPSLDLESNSNDGDILWSSGQKWPTTNDIYADVGACINPNSVHTIAPVTQSSILMSPKAQLNLNTH
NYHSNNSPLPSPKEKKSHLTFSPNTIKVPVVQENKKQSLLCQPQRTSVEAIKKDLTDDLRDEKVVKLTHSMTVNGQTNGHNTIKLAAGIGGLSFANSQMYAKLKQNNSKIVANGNITHLKRESSPLNNS
SMIGNNGSLNGHSNGQKMFARSISVQNGKSHISTQHILPSNSLSHHNGVNKQMKPKITGQEFPKPAYSYSCLIAMALKNSRAGSLPVSEIYSFMCEHFPYFKTAPNGWKNSVRHNLSLNKCFEKIEKPV
TNGGQRKGCLWAMNPSKITKMDEEIQKWSRKDPMAIRKAMVFPDNLELLEKGEMKHGSTGDSEGENEVGDEDTETNTDSEQDVTELDDTIQNTTEPESCDEDDRPQIDYDIEVTDLYDDIDLNDANQKD
LLSPEDIHPAKRARLDINYSIGPAGTGFQSLNGSQQPQQNRRKMTLVNRIVIRWDHNRKRGSVVCVEQ
>Bcop_v1_g008508-RC, jumu/jumeau-like
MNQFEEISTGFHDIFLNKFADSSGPILDFYVSDSMQDMLDIDIRSEVATVVGGVSEFTSLMNDLPSLDLESNSNDGDILWSSGQKWPTTNDIYADVGACINPNSVHTIAPVTQSSILMSPKAQLNLNTH
NYHSNNSPLPSPKEKKSHLTFSPNTIKVPVVQENKKQSLLCQPQRTSVEAIKKDLTDDLRDEKVVKLTHSMTVNGQTNGHNTIKLAAGIGGLSFANSQMYAKLKQNNSKIVANGNITHLKRESSPLNNS
SMIGNNGSLNGHSNGQKMFARSISVQNGKSHISTQHILPSNSLSHHNGVNKQMKPKITGQEFPKPAYSYSCLIAMALKNSRAGSLPVSEIYSFMCEHFPYFKTAPNGWKNSVRHNLSLNKCFEKIEKPV
TNGGQRKGCLWAMNPSKITKMDEEIQKWSRKDPMAIRKAMVFPDNLELLEKGEMKHGSTGDSEGENEVGDEDTETNTDSEQDVTELDDTIQNTTEPESCDEDDRPQIDYDIEVTDLYDDIDLNDANQKD
LLSPEDIHPAKRARLDINYSIGPAGTGFQSLNGSQQPQQNRRKMTLVNRIV
>Bcop_v1_g000093-RA, MBD2-like
MNVTIERKSDCAALPKGWQREEIMRKSGLSAGKVDVFYYRQTASIFKQPVTVYKTQDSKSKADLKHGTLDKPNQLFWAKRLEGLRACDVDGVELGPVELPKGLKPVGPNVDDGTLLQSVATSLHVTSQA
VTGQQASKAVLQMNPGVFLNPDQPLMHSVNINEDDIRRQEDRVQVARRKLQEALKC
>Bcop_v1_g000722-RA, ALKBH8-like
MTSKSIERKAGRKQKRCQHIITSETGIECNDAPKQYLVVCNAGLSTGLKNDVLLKEAAIFGTVLQIILLPGKSYSFMKCKTIDDAISIYNGMNARSTLGQNGSVLYLLYCEDVPDSVNPWNHQVPPGLI
LIDNFITELEEESLLNRVSDDFTGHDSSLGNSGSMKHRQVYHYGYEFKYDTNNVDATQPLAMQIPDECNRLWPRLRSSMPNLSFQVPDQITVNKYEPGQGIPPHCDTHSAFCDPIFSLSLASGTVMEFR
RPSDGRHISVWLPRRSLLVMSKESRYGWTHGITPRKTDVVPVNEGLTVERLRVSFTFRRLSFDKCRCEFTSLCDTYQKSQQETSENIEKLVLPAELELRNVHKVYDQIADHFSETRHSPWPKVKEFI
ESFPAGSIVVDVGCGNGKYLPLNDSIVKIGCDRSHGLLNVCNQRGFNIFQCDCLHLPIKSNSVDGCISIAVIHHLVTEERRQQAISEMSRILVTGGRCLIYVWAKNQAKGTKSSYLRQNKKNNKVDVVA
DDASSCNAKFCDGLPIHTNRTQFSHSDMLVPWKRKDETGDEQKTFLRYYHVFEEAELQRVCERVSDIAVIETYYDQGNWCVIFEKK
>Bcop_v1_g001386-RA, ALKBH2-like
MMSVIEQLNEIGNKPIETKNLKNYRRIVAEHLDVETAILFDKRLSYEIYKGLDDAVEYYEENSLKVKVFGKWHPVPRKMASLIPFAAYCDEGITYTFSGLTMPTRNWFPLLSQLRDIVTKLTGFDYNFV
LVNRYENGNQHIGEHRDGEKEIDPSSPIVSLSFGQHRDFVFRHSNSKARPDLTPIKMELPNGSILVMNPPTNQFWLHSLPIRKKALHPRINLTFRKIFRSE
>Bcop_v1_g016925-RA, METTL14-like
MGDVLKNLRIRSQKRKKLLAQTLGVSKVEDLKHVLGTAEDVQSTKTQRDDDEPTSSTKKFQSESMFYRDSSTFLKGTQSSNPHNDYCQHFVDTGQRPQNFIRDVGLADRFEEYPKLRELIRLKDELIEQ
TATPPMYLQADLKTLDLKSLGTKFDVILIEPPLEEYARAGAGVAVSSSTRTFWSWDEILSLDIGEVAAHRSFVFLWCGSSEGLDMGRNCLRKWGFRRCEDICWIRTNIENPGHSKILEPKAVFQRTKEH
CLMGIKGTVRRSTDFDFIHANVDIDLIISEEEQLGSFEKPIELFHIIEHFCLGRRRLHIFGRDTSIRPGWLTVGPELTNSNFNGELYASAFADNTTTGCTDRIEALRPKSPPTNGKMMRGRGRGGYVRG
RGRGR
>Bcop_v1_g011218-RB, BAZ2B-like
MDKENGDNNEKGKGGHSGGGSGQDPSSLLDAASLFAYWGRDPTAAQAAAAASSQLFGSPFGAAAAGLGLLPGSGGSSNDRYSMNNHQHHQNTMAVAASQAASLAGLHPASWWSMAQLAAQDYFARLQA
SGLSPFQHPELAAFPGMGMGGNVGGSSSGTGRQGAGEGKSSTKGRKEKKSNNSNNSMGGSSGNASTTVTSSAGTSSAYKQSSSQSYKSAANQYSPATLHKELLAMQAAAAASGSGHSSSRKSSQSSHKQ
HQSSAHHTSSSGHGSMGNHSSGRDSHHGSNSGKESSSKNSSSALATSLASSNATLTMNALNSLSQFGNLGSLSSQHSMQAAMNALAGNSSSKSSKDYIPSGILSAALERNDPSSLLGVRLPPDTEIIKY
TSSIVGPKIPGTTNRGRKKTISLDSNPLLAGIPGLTAGTGVSPAKRARLEAEYGNMVQSAHQSDRDRVEVIKLPTSINSNGAYNLSKSNAQAKDTSFDSSNEWATGLNLSGKASTTSSTDDGDAPLNLC
MKSDGKSGDAGTGTTGSNSLQSLSHITAALGSGSGNDRMLHYKEGRPRNLGRGVSKPKKNTVASLLAQSRAVGLKPMLNTQQLLSQGADLEKIRQAIAEAHLAMEVSTDSESLADASGMSDSEGEEMVN
LAELRAPLDKGWKRETIIRGLTKNMQIRGDVCYYAPGSQMKLKHIGQIQTILEQQSSNFTTDNFSFSARPIIGSFLQPYSSATDAECIRMTDSEVAKRLEELKLYTKQSLNVEQRIEIARQQQALRDAK
KMAKDDMSRTKEKPVSYQRGKQNRDVEKVERLEAQRKERELKNQQALEERERKRVEQEALLQKEQIYLQDLNKQKELLYTVELERERRQHMTLIRQLENRRKYEERERKKHQMVLDKLISREKKLMVKKR
DAEILAELRKPQEDSEIPNQKDLPELQRLPGLKLTGQALSDLLMVFEFLHNFGETLGFDMDSLPTLQSLHQALCSDGTATDAEDELISVMTHLLVCAIEDPGIPNPNRHTTLLGQSLRTADITNSNISE
ILRIYLYAVATGEVRQTSGLTFDRDRERRVADHHSTEQFDISTSKNQQYYEQLHENTTWKLSECLKENPFVSLNSTVKSQILAHLCNDLLLNKAVSKQIEGSLESMAQYKREKYLLDNRIRKYKHLHTR
KIRMEQFEKQQMLAKQSESIPKDGFVEPTRQGPVGDNLVNENNNETPDKLNESVSNSNLIRTASNLTNASSIDNLTDVSDLANHKDDSLHSTDVALTKNGDEMSPVKSVDDIKANKSLDDFSLIDTPRS
LNNGTSTPDMNSLLNKKIINRELNDVSSTADLNLDEDLSDLESEGTILEEDEDNRMTAEEVLKKLEKIMKAGQQNKMLLEQSCNQLRATCYGQDRFWRRYWYLPKAGGIFVEGLESAQPDILKYHSLDD
EVLSTSSHSVSVPVGENTEKKKRGRKRKHQTDEEESSSKNVTEDAQRVNNETKPTNEVKPVTELKPGDEVKATNDIKPTSDMHQNDDGMMDIEDSIPRAILVQKACTIDDVEMNSKISGVSSSVVDHVS
QTATNSTSNPICNQEEGSSNSGASSDVVVVADDTQIAKLENGAVKEEPATETDANPDPSKAGSEVAVKQEIKDEIKNEGDDDTDGVVKSETLMEKWFSIANEELRQLTSTECTPTMACQASFSNVTCDNT
IQYQGNRWEIGNNAQYFNVPVEGLTSTLTFNRDSFLTLSGLDEDMMSQALNSTLKPKDDTLQDVTMVDDEIAKSTINKIDVKQEEMLVPFNLPSFINMSLGNISAYIQCDNPSPLQMTPDEQKLLEEVK
INGLPKRLERNLVPKDLRYGWWKIDDIEVIKEMIQCLHVRGVRERELRQHLLTALSENLDLTTQCHVAHVRAPPPSKGYIDPEPMNAWNPQIARRVELNLLDQVETLEDKIASASMQMKGWQVPNKDDV

ENGMDMIGGISLIRERILGLEAAIERRYLKPPLGTSTADAHIAAIATQNQNQANPPTSTSQAASSSTTPTSSSATSATTNSDQQANDTDQDMDREESPEPEVLPKALQSWREAVARSHTTSQLAMALYV
LESCVAWDKSIMKAPTQNGSNTQRSLQNCQFCSSGENEDKLLLCDGCDKGYHTYCFKPKMVNIPEGDWYCYECVNKATGDRKCIVCGGHRQQPVGKMIYCEICPRAYHHDCYIPPMIKVPRGKWYCHGC
ISKAPPPKKRGPKKGHKEKGEKSEKGEKSDKTEKADKADKSSNSTLTATPTLNSSGQEDGALSPTHSVASTSFEEHSTSVDSKPPSTTTAQTAPIISATTSADIVDEEMPIADDVSDSAAPSSPPHTSQ
TVASVQSPASSPPPSSPPPPPPPQAVQPTTAPITTAAAAMEELNHTAALLNEAKEKLKLEKKERHAAKKLIKELAACKTMLEAMELHEDSWPFLLPVNTKQFPTYKKIIKSPMDLSTIKKRIQDLQYKS
RDDFIADVRLIFDNCEIFNEDDSPVGKAGHGMRKFFEVRWAELTDKHS
>Bcop_v1_g011218-RA, BAZ2B-like
MDKENGDNNEKGKGGGHSGGGSGQDPSSLLDAASLFAYWGRDPTAAQAAAAASSQLFGSPFGAAAAGLGLLPGSGGSSNDRYSMNNHQHHQNTMAVAASQAASLAGLHPASWWSMAQLAAQDYFARLQA
SGLSPFQHPELAAFPGMGMGGNVGGSSSGTGRQGAGEGKSSTKGRKEKKSNNSNNSMGGSSGNASTTVTSSAGTSSAYKQSSSQSYKSAANQYSPATLHKELLAMQAAAAASGSGHSSSRKSSQSSHKQ
HQSSAHHTSSSGHGSMGNHSSGRDSHHGSNSGKESSSKNSSSALATSLASSNATLTMNALNSLSQFGNLGSLSSQHSMQAAMNALAGNSSSKSSKDYIPSGILSAALERNDPSSLLGVRLPPDTEIIKY
TSSIVGPKIPGTTNRGRKKTISLDSNPLLAGIPGLTAGTGVSPAKRARLEAEYGNMVQSAHQSDRDRVEVIKLPSTINSNGAYNLSKSNAQAKDTSFDSSNEWATGLNLSGKASTTSSTDDGDAPLNLC
MKSDGKSGDAGTGTTGSNSLQSLSHITAALGSGSGNDRMLHYKEGRPRNLGRGVSKPKKNTVASLLAQSRAVGLKPMLNTQQLLSQGADLEKIRQAIAEAHLAMEVSTDSESLADASGMSDSEGEEMVN
LAELRAPLDKGWKRETIIRGLTKNMQIRGDVCYYAPGSQMKLKHIGQIQTILEQQSSNFTTDNFSFSARPIIGSFLQPYSSATDAECIRMTDSEVAKRLEELKLYTKQSLNVEQRIEIARQQQALRDAK
KMAKDDMSRTKEKPVSYQRGKQNRDVEKVERLEAQRKERELKNQQALEERERKRQEALLQKEQIYLQDLNKQKELLYTVELERERRRQHMTLIRQLENRRKYEERERKKHQMVLDKLISREKKLMVKKR
DAEILAELRKPQEDSEIPNQKDLPELQRLPGLKLTGQALSDLLMVFEFLHNFGETLGFDMDSLPTLQSLHQALCSDGTATDAEDELISVMTHLLVCAIEDPGIPNPNRHTTLLGQSLRTADITNSNISE
ILRIYLYAVATGEVRQTSGLTFDRDRERRVADHHSTEQFDISTSKNQQYYEQLHENTTWKLSECLKENPFVSLNSTVKSQILAHLCNDLLLNKAVSKQIEGSLESMAQYKREKYLLDNRIRKYKHLHTR
KIRMEQFEKQQMLAKQSESIPKDGFVEPTRQGPVGDNLVNENNNETPDKLNESVSNSNLIRTASNLTNASSIDNLTDVSDLANHKDDSLHSTDVALTKNGDEMSPVKSVDDIKANKSLDDFSLIDTPRS
LNNGTSTPDMNSLLNKKIINRELNDVSSTADLNLDEDLSDLESEGTILEEDEDNRMTAEEVLKKLEKIMKAGQQNKMLLEQSCNQLRATCYGQDRFWRRYWYLPKAGGIFVEGLESAQPDILKYHSLDD
EVLSTSSHSVSVPVGENTEKKKRGRKRKHQTDEEESSSKNVTEDAQRVNNETKPTNEVKPVTELKPGDEVKATNDIKPTSDMHQNDDGMMDIEDSIPRAILVQKACTIDDVEMNSKISGVSSSVVDHVS
QTATNSTSNPICNQEEGSSNSGASSDVVVVADDTQIAKLENGAVKEEPATETDANPDPSKAGSEVAVKQEIKDEIKNEGDDDTDGVVKSETLMEKWFSIANRELQLTSTECTPTMACQASFSNVTCDNT
IQYQGNRWEIGNNAQYFNVPVEGLTSTLTFNRDSFLTLSGLDEDMMSQALNSTLKPKDDTLQDVTMVDDEIAKSTINKIDVKQEEMLVPFNLPSFINMSLGNISAYIQCDNPSPLQMTPDEQKLLEEVK
INGLPKRLERNLVPKDLRYGWWKIDDIEVIKEMIQCLHVRGVRERELRQHLLTALSENLDLTTQCHVAHVRAPPPSKGYIDPEPMNAWNPQIARRVELNLLDQVETLEDKIASASMQMKGWQVPNKDDV
ENGMDMIGGISLIRERILGLEAAIERRYLKPPLGTSTADAHIAAIATQNQNQANPPTSTSQAASSSTTPTSSSATSATTNSDQQANDTDQDMDREESPEPEVLPKALQSWREAVARSHTTSQLAMALYV
LESCVAWDKSIMKANCQFCSSGENEDKLLLCDGCDKGYHTYCFKPKMVNIPEGDWYCYECVNKATGDRKCIVCGGHRQQPVGKMIYCEICPRAYHHDCYIPPMIKVPRGKWYCHGCISKAPPPKKRGPK
KGHKEKGEKSEKGEKSDKTEKADKADKSSNSTLTATPTLNSSGQEDGALSPTHSVASTSFEEHSTSVDSKPPSTTTAQTAPIISATTSADIVDEEMPIADDVSDSAAPSSPPHTSQTVASVQSPASSPP
PSSPPPPPPPQAVQPTTAPITTAAAAMEELNHTAALLNEAKEKLKLEKKERHAAKKLIKELAACKTMLEAMELHEDSWPFLLPVNTKQFPTYKKIIKSPMDLSTIKKRIQDLQYKSRDDFIADVRLIFD
NCEIFNEDDSPVGKAGHGMRKFFEVRWAELTDKHS
>Bcop_v1_g017396-RE, MBD5-like
MAAASSGTLQIQGSVPSISQSHLHQQQQQSFQQRLSYVQRSGSQLNVVSGCGPVTVSAIQQRGPNVAPGWRRQVNNGEIIYISPSGAVLRNLGQIKEYLLSAGTCKCGLPCPLRPDYFFEFNSQVPNVK
LQIPLEDAGTIRTTSCLHQARLLGSNNLLPKDTSPSKKRKLDGAWSPASSISSSDSTRSSQTTALCNYVGSPIPNQMVIANPRVQPTIIQKRQVVGIPAQQHLPQQQSQTVVYQNQNVGENKVANVTVS
RTPPWRKNSIRPNFKEDPTGYLDHQTAILHSSILNVHSPDIAENSNQSTISNSNVSYQSEPKLSNQLNVSTPNNDSVQPHTIGSEQSLSVMQQHQHNQNVFIQQQQQQSGVESAGGKTQPHVIIGSNGQ
PIRILQNGQIIYSNPTMQSVKVQDTQNNASTGIVSSGPRIISDEIIQPVQTTNVHQQVFQQQNAPRMMAPQQIIPQGQMEPVQIQHNRQVLQQQPQQQASSGQTQIITVQQHVPTSQPFAMNQSVRQPH
PQSIVQQQAVNRSHQDHRIIMPSSNQTDSNQSMELSIGGQTVTHLPNGMVQVQQNCDIAHMKQQQQIQLQQQIQQQNMLFRQQQELQQNPRLKDQYQTCNSTQDTIRQQHQVSTRTVATSQESPVSSST
TYRNKIIEKRSSSKGPAQVGAISTSNESPPATSSCSSPADSPESLQARTVTSAAALCPPKIQTGGIVRSQTTGKNTITSVLAGKAMTSTTTTNHGFGKQSGNANEKDTAPNTFDRQIVHVSSSQMVQIP
SSVHQAKISTAGSNQNRPIASMIQNCVPVSAALTNLQLQPICRGEPGSQIIMTSSGQILVMPPQSKSSNNQMIISGGNGSSLVMNNSPQGVVINQQGQQILSGDMIHGINNQVLDGQSANILQNSGNQV
VIQGQNLMSGNGNRVIGSNNSNFIVNSTNNMQPMIINNSNIISHNGNVLQQGQNVIHGNQGNLISGTKVISSNGNILNGSNIISNQSNVLASNNQIIGNSNSTNLISPNGTVVLPNGYVLQPHQFTTVD
GQVVNVINQDNGTHQFIQSTQQRIILSPDSKRRAKKRKSSSGTPPTPHSLSPQQSPTIQQHSPTIVQQNQSGAMLQITPQYQQQSFQISPGMSGITLVQNKPQTNSGPQQQILLQNGQTIIQPLNIIGQ
QLLVPAGLMVAPDTTLLQIQNVGPCGSILTPQGMMIRASSPQNKNFLSPNSGGQQFIVSGNGQISPIGQMYSTPMGLVVPQTNSNNSGPTYVQQNTTILQQQTTMINSANGNGGSSNVDADIRSQNQQS
MSQSQQQQHHHHRTVSVSPPDTTTHSPRSPERPPSQRSNGSDTNMVQCVSSSEPDAVSPVADSARSPSNNSDYERSGGISYQQNTFKPSDAKIRRIQTPTQLSNSHVITQGNQSKMRPDSHVWGAARGH
PAWPGKIVSPPETYKGVATSSDSTWVQWFGGRPNVELVSINSLKSLSEGLEAHHKAQKDTRKSRKLNSQLECAIQEAMAELDRTVTATSSTPTTPSNRNKARTLKPTSTQQRTKPVKIAPAPPNSTTHL
PAKYLRQSK
>Bcop_v1_g017396-RD, MBD5-like
MAAASSGTLQIQGSVPSISQSHLHQQQQQSFQQRLSYVQRSGSQLNVVSGCGPVTVSAIQQRGPNVAPGWRRQVNNGEIIYISPSGAVLRNLGQIKEYLLSAGTCKCGLPCPLRPDYFFEFNSQVPNVK
LQIPLEDAGTIRTTSCLHQARLLGSNNLLPKDTSPSKKRKLDGAWSPASSISSSDSTRSSQTTALCNYVGSPIPNQMVIANPRVQPTIIQKRQVVGIPAQQHLPQQQSQTVVYQNQNVGENKVANVTVS
RTPPWRKNSIRPNFKEDPTGYLDHQTAILHSSILNVHSPDIAENSNQSTISNSNVSYQSEPKLSNQLNVSTPNNDSVQPHTIGSEQSLSVMQQHQHNQNVFIQQQQQQSGVESAGGKTQPHVIIGSNGQ
PIRILQNGQIIYSNPTMQSVKVQDTQNNASTGIVSSGPRIISDEIIQPVQTTNVHQQVFQQQNAPRMMAPQQIIPQGQMEPVQIQHNRQVLQQQPQQQASSGQTQIITVQQHVPTSQPFAMNQSVRQPH
PQSIVQQQAVNRSHQDHRIIMPSSNQTDSNQSMELSIGGQTVTHLPNGMVQVQQNCDIAHMKQQQQIQLQQQIQQQNMLFRQQQELQQNPRLKDQYQTCNSTQDTIRQQHQVSTRTVATSQESPVSSST
TYRNKIIEKRSSSKGPAQVGAISTSNESPPATSSCSSPADSPESLQARTVTSAAALCPPKIQTGGIVRSQTTGKNTITSVLAGKAMTSTTTTNHGFGKQSGNANEKDTAPNTFDRQIVHVSSSQMVQIP
SSVHQAKISTAGSNQNRPIASMIQNCVPVSAALTNLQLQPICRGEPGSQIIMTSSGQILVMPPQSKSSNNQMIISGGNGSSLVMNNSPQGVVINQQGQQILSGDMIHGINNQVLDGQSANILQNSGNQV
VIQGQNLMSGNGNRVIGSNNSNFIVNSTNNMQPMIINNSNIISHNGNVLQQGQNVIHGNQGNLISGTKVISSNGNILNGSNIISNQSNVLASNNQIIGNSNSTNLISPNGTVVLPNGYVLQPHQFTTVD
GQVVNVINQDNGTHQFIQSTQQRIILSPDSKRRAKKRKSSSGTPPTPHSLSPQQSPTIQQHSPTIVQQNQSGAMLQITPQYQQQSFQISPGMSGITLVQNKPQTNSGPQQQILLQNGQTIIQPLNIIGQ
QLLVPAGLMVAPDTTLLQIQNVGPCGSILTPQGMMIRASSPQNKNFLSPNSGGQQFIVSGNGQISPIGQMYSTPMGLVVPQTNSNNSGPTYVQQNTTILQQQTTMINSANGNGGSSNVDADIRSQNQQS
MSQSQQQQHHHHRTVSVSPPDTTTHSPRSPERPPSQRSNGSDTNMVQCVSSSEPDAVSPVADSARSPSNNSDYERSGGISYQQNTFKPSDAKIRRIQTPTQLSNSHVITQGNQSKMRPDSHVWGAARGH
PAWPGKIVSPPETYKGVATSSDSTWVQWFGGRPNVELVSINSLKSLSEGLEAHHKAQKDTRKSRKLNSQLECAIQEAMAELDRTVTATSSTPTTPSNRNKARTLKPTSTQQRTKPVKIAPAPPNSTTHL
PAKYLRQSK
>Bcop_v1_g017396-RI, MBD5-like
MADTSPSKKRKLDGAWSPASSISSSDSTRSSQTTALCNYVGSPIPNQMVIANPRVQPTIIQKRQVVGIPAQQHLPQQQSQTVVYQNQNVGENKVANVTVSRTPPWRKNSIVNPTGYLDHQTAILHSSIL
NVHSPDIAENSNQSTISNSNVSYQSEPKLSNQLNVSTPNNDSVQPHTIGSEQSLSVMQQHQHNQNVFIQQQQQQSGVESAGGKTQPHVIIGSNGQPIRILQNGQIIYSNPTMQSVKVQDTQNNASTGIV
SSGPRIISDEIIQPVQTTNVHQQVFQQQNAPRMMAPQQIIPQGQMEPVQIQHNRQVLQQQPQQQASSGQTQIITVQQHVPTSQPFAMNQSVRQPHPQSIVQQQAVNRSHQDHRIIMPSSNQTDSNQSME
LSIGGQTVTHLPNGMVQVQQNCDIAHMKQQQQIQLQQQIQQQNMLFRQQQELQQNPRLKDQYQTCNSTQDTIRQQHQVSTRTVATSQESPVSSSTTYRNKIIEKRSSSKGPAQVGAISTSNESPPATSS
CSSPADSPESLQARTVTSAAALCPPKIQTGGIVRSQTTGKNTITSVLAGKAMTSTTTTNHGFGKQSGNANEKDTAPNTFDRQIVHVSSSQMVQIPSSVHQAKISTAGSNQNRPIASMIQNCVPVSAALT
NLQLQPICRGEPGSQIIMTSSGQILVMPPQSKSSNNQMIISGGNGSSLVMNNSPQGVVINQQGQQILSGDMIHGINNQVLDGQSANILQNSGNQVVIQGQNLMSGNGNRVIGSNNSNFIVNSTNNMQPM
IINNSNIISHNGNVLQQGQNVIHGNQGNLISGTKVISSNGNILNGSNIISNQSNVLASNNQIIGNSNSTNLISPNGTVVLPNGYVLQPHQFTTVDGQVVNVINQDNGTHQFIQSTQQRIILSPDSKRRA
KKRKSSSGTPPTPHSLSPQQSPTIQQHSPTIVQQNQSGAMLQITPQYQQQSFQISPGMSGITLVQNKPQTNSGPQQQILLQNGQTIIQPLNIIGQQLLVPAGLMVAPDTTLLQIQNVGPCGSILTPQGM
MIRASSPQNKNFLSPNSGGQQFIVSGNGQISPIGQMYSTPMGLVVPQTNSNNSGPTYVQQNTTILQQQTTMINSANGNGGSSNVDADIRSQNQQSMSQSQQQQHHHHRTVSVSPPDTTTHSPRSPERPP
SQRSNGSDTNMVQCVSSSEPDAVSPVADSARSPSNNSDYERSGGISYQQNTFKPSDAKIRRIQTPTQLSNSHVITQGNQSKMRPDSHESPSTPTLCSTATFCIGELVWGAARGHPAWPGKIVSPPETYK
GVATSSDSTWVQWFGGRPNVELVSINSLKSLSEGLEAHHKAQKDTRKSRKLNSQLECAIQEAMAELDRTVTATSSTPTTPSNRNKARTLKPTSTQQRTKPVKIAPAPPNSTTHLPAKYLRQSK
>Bcop_v1_g017396-RH, MBD5-like
MADTSPSKKRKLDGAWSPASSISSSDSTRSSQTTALCNYVGSPIPNQMVIANPRVQPTIIQKRQVVGIPAQQHLPQQQSQTVVYQNQNVGENKVANVTVSRTPPWRKNSIVNPTGYLDHQTAILHSSIL
NVHSPDIAENSNQSTISNSNVSYQSEPKLSNQLNVSTPNNDSVQPHTIGSEQSLSVMQQHQHNQNVFIQQQQQQSGVESAGGKTQPHVIIGSNGQPIRILQNGQIIYSNPTMQSVKVQDTQNNASTGIV
SSGPRIISDEIIQPVQTTNVHQQVFQQQNAPRMMAPQQIIPQGQMEPVQIQHNRQVLQQQPQQQASSGQTQIITVQQHVPTSQPFAMNQSVRQPHPQSIVQQQAVNRSHQDHRIIMPSSNQTDSNQSME
LSIGGQTVTHLPNGMVQVQQNCDIAHMKQQQQIQLQQQIQQQNMLFRQQQELQQNPRLKDQYQTCNSTQDTIRQQHQVSTRTVATSQESPVSSSTTYRNKIIEKRSSSKGPAQVGAISTSNESPPATSS
CSSPADSPESLQARTVTSAAALCPPKIQTGGIVRSQTTGKNTITSVLAGKAMTSTTTTNHGFGKQSGNANEKDTAPNTFDRQIVHVSSSQMVQIPSSVHQAKISTAGSNQNRPIASMIQNCVPVSAALT
NLQLQPICRGEPGSQIIMTSSGQILVMPPQSKSSNNQMIISGGNGSSLVMNNSPQGVVINQQGQQILSGDMIHGINNQVLDGQSANILQNSGNQVVIQGQNLMSGNGNRVIGSNNSNFIVNSTNNMQPM
IINNSNIISHNGNVLQQGQNVIHGNQGNLISGTKVISSNGNILNGSNIISNQSNVLASNNQIIGNSNSTNLISPNGTVVLPNGYVLQPHQFTTVDGQVVNVINQDNGTHQFIQSTQQRIILSPDSKRRA
KKRKSSSGTPPTPHSLSPQQSPTIQQHSPTIVQQNQSGAMLQITPQYQQQSFQISPGMSGITLVQNKPQTNSGPQQQILLQNGQTIIQPLNIIGQQLLVPAGLMVAPDTTLLQIQNVGPCGSILTPQGM
MIRASSPQNKNFLSPNSGGQQFIVSGNGQISPIGQMYSTPMGLVVPQTNSNNSGPTYVQQNTTILQQQTTMINSANGNGGSSNVDADIRSQNQQSMSQSQQQQHHHHRTVSVSPPDTTTHSPRSPERPP
SQRSNGSDTNMVQCVSSSEPDAVSPVADSARSPSNNSDYERSGGISYQQNTFKPSDAKIRRIQTPTQLSNSHVITQGNQSKMRPDSHVWGAARGHPAWPGKIVSPPETYKGVATSSDSTWVQWFGGRPN
VELVSINSLKSLSEGLEAHHKAQKDTRKSRKLNSQLECAIQEAMAELDRTVTATSSTPTTPSNRNKARTLKPTSTQQRTKPVKIAPAPPNSTTHLPAKYLRQSK
>Bcop_v1_g016381-RA, TET-family, TET2-like, DMAD-like
MKEQPQQPMAPMAFYPTWQADPSQGWQNQFIQQIPQNTPAITSLNSIEFQPQGYSYQPNGYVQTGLGFEPNYGRTYATPVQRYEFQTNQMSPINQVSLQSVSFAPAVTYTSQVSTGSTPMLLSSQNDLN
KTVSGNDMPGYPRVNSVPPRSLNCNGYSGDYSGNQNHNLTNNVPQQQQHNQHQQQQQQQMQQHHQQQHNNSNQMHPPQVPQSPNRSNMSTPVSQHMMVPTGPGSNHMSQIPQSPNSLQNNGNQQQIQMQ
SPNHHHSQLQTASRYPSHPGTPHSMQQSPNKNPQMLHSHSGMHTPNPQHTAHQHNMTAMSPNHNMNHVQEWSWNNHTVPAGNNEIFNQSDRVNLNTRLKTMILSKNDQKDHDHQNGDGTASAANQQNTA
QTGHFLSYSHHLRDINPTTNDSRCLQPCSTQVAEPIGGGGESIWKPFGYSKPNDFQYSDQKLENSVRKTDYGTDETKEKNAKGKATAAQKSQNVDGVKVKIEPEKVKPKRQRKKPAAKQTQVNDGPNKS
SNNESCHNANSRQSYSTYGIDHIHPPHEQPMQQQPIPKPYYLPKENETVPTPYDPAIKIKQEPMSFSNYNPTTNHPVENFDLAQVKMEGYERNYQNFINYADYCQSQNPTANRGQQQQPTQNETDYPCF
NPNYNYPSFSTANYQTNYPNYNQYTGQQVQQQPMNIEPVPQATKQEPYLQEQHVKTENESENKIVLTNYEKEIPVYTYPNPSKLPAGDTNQKAEPQKFPYLGEGGPATLSESTGFSCCRQGGTNQPSDE
HIRDGTCVGRQTKDELLLDDDAEKSQDEAGERNAVPSPDRTEKGLKPEVPDCDCFSSDKNPPEPGSYYTHLGSAASLEDLRKDFEQRVGITGRQLRIEKIVYTGKEGKTGQGCPLAKWIIRRVDLEEKI
LCLVKRRQGHRCQASFIVVCIVAWDGLPKYEADNAYKLLVHKLNKFGLPTTRRCATNENRTCACQGLDPETCGASFSFGCSWSMYYNGCKYARSKTVRKFRLSVKSEEPEIEEHMNVLATILSPLYVNV
APKAYENQCKYEQEAPDCRLGLKQGKPFSGVTTCIDFCAHSHRDLHNMQDGCTVQVGLLRQRGPGVAAAAEDEQLHVLPLYTMDTTDEFGSAEAQTEKNKTGAVQVLEKFPCEVRVRATPLQPCRRHGK
KRNGKEVAEDPPPPPTLPAEIQPAKKESSKKSAKNKSKQPPPSSPSPRAQTPQASNTSPSTNNTSAFSTPINQNLMNTNSSLINMASMIDNFTDAQLQSNHISSTVLDSPYSYDYTTGSYIDNRNFYNQ
WPPGSDYGYPDRCEQVKSRGSEENPDSTTLSTANSAFSPKITDPNKFSLLDDSTKDQGFAKPKPPEYPHNYGYHSNKPAYSPMYPPYTPYEHYNNMDYYSNDKLRYNFYNPHHHQPYPQYNMYHSNPAL
SAPSPSPQPNWNMYPSPAPMSMHPMQPQMQQPSPKMPEHITPTSEPIGEITEVNDNLECFQDSQMGGVGIALGHGSVLIECAKHEMHATTALRKPNRLNPTRITLIFYQHRNLNRHKHGIEEWEEKMRL
KKLGITPQDVEEANKEKAIKLEEDIKAEMRDEPPAGEKFELDYEKLNRDKEEVSLRAPTLTTTSWTTLFPMHPCVVTGPYQEGNNPTATQSVAEGSVQQQQQQQQ
>Bcop_v1_g016384-RA, TET-family, TET2-like, DMAD-like
MSVQTTEDGTDNSLPSFSTFSSELDPSWDYYDRDRSSTDVITSQSNNYTRPWEMETKDKGFEPFSKLPSFQSQFHAYSETNLVPEPSLPQITPVPVPVSPASPGNGSLTQLTPINSIQSGLTQLQSPSF
HTLTAVNTRTYPLVPAPIQARDIPAINQQYLEERHIQLYQPISTFPPQNMVTVIKNEPNYEIKNGLHHTNFMNPLIDNGFDIKVEKSESPVVRCDARKKERRKNRASSLESSAESESSAMDMGDGNPGQ
VAAISSTASFKSPMGSMGMNEDSEGNGEKQTKKKRKRCGECIGCQKKDNCGDCAPCRNDKSHQICKQRRCEKLTDKKILLSLCFCILDTLDTTLQQQQKKVTQCLIHQLYTPIGTTPKLESYFDFEKGY
KVEIFWTVDIYCSIGKLICIHQMLALVTLVQSQQSRVLSEILSTKIKLGREAHIILLLRIHFSVMLCKNNLELELENLKMFH
>Bcop_v1_g016384-RB, TET-family, TET2-like, DMAD-like
MSVQTTEDGTDNSLPSFSTFSSELDPSWDYYDRDRSSTDVITSQSNNYTRPWEMETKDKGFEPFSKLPSFQSQFHAYSETNLVPEPSLPQITPVPVPVSPASPGNGSLTQLTPINSIQSGLTQLQSPSF
HTLTAVNTRTYPLVPAPIQARDIPAINQQYLEERHIQLYQPISTFPPQNMVTVIKNEPNYEIKNGLHHTNFMNPLIDNGFDIKVEKSESPVVRCDARKKERRKNRASSLESSAESESSAMDMGDGNPGQ
VAAISSTASFKSPMGSMGMNEDSEGNGEKQTKKKRKRCGECIGCQKKDNCGDCAPCRNDKSHQICKQRRCEKLTDKKSC

```
>Bcop_v1_g020988-RA, DNMT2-like
MDDFRVLELFSGIGGMHYALKSSQIPGTVVAAADINTIANDVYRHNHPDIKVLNNNIQKFTPEFIRTLNVNTILMSPPCQPFTRVGNQMDVEDRRADPFVHICQILPELKCIDWILMENVKGFDTSKAR
EMFVESLRQSEFHYQEFILSPTQLGIPNSRHRYYCLARRSKAFTFASERILTSLPSADTMVPDVQQISQFISSVDANGLPLPEKVLQKYFSVLDICYPDSINSMCFTKAYTHYAEGTGSVFCPLDRNAV
DKCCSQLELIFDDKTTVDLLKSLQLRYFTPAEVSRLMCFPENDFSFPEKISNKSRYRLLGNSINVLVVSELIKLLYED
>Bcop_v1_g019039-RA, SETDB1-like
MDKTPDCNLDRIKDEPAECKNEPLSRCDGQANEFVDNTSIMVSVPLSASEISEIDSIVLNDSVSKEVENDKPADVPPVEPMEVNEQDKSDIPDANLTEDIDLPSTATQSTTVATCDKSIADPSSTQPNI
EKGDDTNVDENDVVMLLSDSEDEDTARKSRLVDTAEMIEKFSTQGVTLEVVKPANDKLRAASVRTENVENKTNDNDTSVIMLDDSPREVEAPTNDRDSAKRNAIEVDEEPPPQTANETDDPAQNALQMI
RQLVDGCYPDDKESTAATTATTTTTTTTASKSTDSPAPNESQKTQSTFRLVGMDKLLETNSVKSGEKRECINFDCKSKKSVEFYKAPLLALSHFNVPRKVNRAQFICQNCFDVSVNDYERMGVALVNQQ
PLLLEEVPVRPEVVEILDSEEEDNGGGSSKYVDDIKPLSLDTLTLLEDHFEDVLKEAFNRINIAQQMAWTDQILNHKIDKNETKTEELELQVKSMQKLADSMYDKLYKNTNFVIEELPPFDLNENKQLH
MYGPNYPPQGELIYPPVDMNSLYYAVRAKLLTTWIPCKVTERVDASVDGKKMFKVRFLRTKGVLNKTVPGNHLAYGTAPSVRLTVGTRVIALFTMEPHDNLRSTFFPGIVAEPLQQYNKWRYLIFFDDG
YAQYVTPDNLRLICSPSPNVWEDVYPDAAEFIKSYLEQYKTQRPMVQVKRGQRMITEWNSEWTHARVHDIDGSLVQMYFENAKRSEWIYRGSTRLGPLYKDRQLQKHSVSSGKRNEPFVEYMTIDDDGK
NKTSTAAKPVQAQKPVAAKPSHTNTPSQTPNPPSHTPNTSSQVAPQSGEEKRSVARKSVTAPQRPQPAVQHMNSATIYIEEDNRPKGKVVYYTAKKHMPPRKFVPHKCGVGCLYEVKHNLSTYSPLAKP
LLSGWERKILRNKTKKSVEYRTPCGRNLRNMAELHRYLRQTKCALNVDNFDFDFMVHCLAEYVIDTCVVQKQDLSDGCEPMVVPCVNSYDNTMPPPCVYSAKRIPNEGFGNPNTPIDQVGYEFKRLPNP
VPTGIYECNTRCKCSKTCLNRVAQHPLQLKLQVFKTVSRGWGLRCLNDVPKGSFICIYAGDLLTEQNANIAGDNYGDEYFAELDYIEVVENLKEGYEPGVMDDDFDDDEFDPDKTKDDSDDDEYVATVS
QGPRQTRYNTRHSVDASNKKKTTETPKLKVDPASGSNSDEEDAQRQLISFMPNADSVEFENNTTNRYRSIRKFYGKNESVFIMDAKKLGNIGRYFNHSCSPNLFVQNVFVDTHDLRFPWVAFFALSHIR
AGSELTWNYNYDVGSVPGKVMYCECGAENCRGRLL
>Bcop_v1_g018977-RA, ALKBH6-like
MDISDFTVQSCPNTVIYIPNFITPAEEQKILTSVYNAPKPKWDQLTHRRLLNYGGVPHKNGMIAEEMPGWLQNYVDKVNNLGIFDTKKANHVLVNEYLPKQGIMPHLDGPLFYPIITTISCGSHTVLEF
LEANDNNPEFSETLMSANDGSSDQMDGKLSRKIMCKLFIEPRSLLILRDDMYHKYLHAICEIDEDVISENVANLQHCSNSYKLGDKIKRETRVSLTIRHVPKTSKMKLKLGN
```

Supplemental Table S18 A-F: DNA modification percentages in male embryonic genomic DNA.
Note in tables A and B below, the effective genome size is doubled since coverage is calculated per strand and counts of how many bases and how many modified bases are from both strands.

S18A: Statistics on all putative modified adenines given minimum strand coverage levels

Min. Strand Coverage	#A = Number of adenines ≥ Min. Coverage	#A as % of all adenines in the genome	#modA = Number of modified adenines ≥ Min. Coverage	#modA as % of #A	#6mA = Number of 6-methyl adenines ≥ Min. Coverage	#6mA as % of #A
3	184940136	98.9739	247015	0.1336	66414	0.0359
5	182482744	97.6587	245841	0.1347	66414	0.0364
10	158406693	84.774	233673	0.1475	63472	0.0401
15	110866645	59.3322	198921	0.1794	53535	0.0483
20	50775937	27.1736	120845	0.238	31325	0.0617
25	12788726	6.8441	41950	0.328	10218	0.0799
50	52445	0.0281	1090	2.0784	210	0.4004

*modA is any adenine that was flagged as "modified_base" by kineticsTools and 6mA is the subset of modA that was predicted to be 6mA by kineticsTools.

S18B: Statistics on all putative modified adenines inside GAG motifs given minimum strand coverage levels

Min. Strand Coverage	#GAG = Number of GAG ≥ Min. Coverage	#GAG as % of all GAG in the genome	#modGAG = Number of modified GAG ≥ Min. Coverage	#modGAG as % of #A	#6mA-GAG = Number of 6mA-GAG ≥ Min. Coverage	#6mA-GAG as % of #GAG
3	5365180	98.9799	47082	0.8775	16464	0.3069
5	5296391	97.7108	47022	0.8878	16464	0.3109
10	4614029	85.1222	45850	0.9937	15875	0.3441
15	3229440	59.5785	40036	1.2397	13314	0.4123
20	1446315	26.6824	24534	1.6963	7447	0.5149
25	347929	6.4188	8261	2.3743	2310	0.6639
50	1218	0.0225	101	8.2923	11	0.9031

*modGAG is an GAG flagged as containing a modified A and 6mA-GAG is the subset of modGAG where the modification was predicted to be 6mA by kineticsTools.

S18C: Statistics on all putative modified cytosines given minimum strand coverage levels

Min. Strand Cover-age	#C = Number of cytosines > Min. Coverage	#C as % of all cytosines in the genome	#modC = Number of modified cytosines > Min. Coverage	#modC as % of #C	#4mC = Number of 4-methyl cyto-sines > Min. Cover-age	#4mC as % of #C	#5mC = Number of 5-methyl cytosines > Min. Coverage	#5mC as % of #C
3	102025314	98.9127	610766	0.5986	115955	0.1137	267966	0.2626
5	100469957	97.4048	609640	0.6068	115955	0.1154	267757	0.2665
10	86136561	83.5087	583489	0.6774	112556	0.1307	253146	0.2939
15	58293210	56.5148	486588	0.8347	98439	0.1689	202877	0.348
20	25049775	24.2856	278927	1.1135	61097	0.2439	108946	0.4349
25	5881088	5.7017	89906	1.5287	21491	0.3654	32944	0.5602
50	22901	0.0222	2347	10.2485	706	3.0828	579	2.5283

*modC is any cytosine that was flagged as "modified_base" by kineticsTools whereas 4mC and 5mC are subsets of modC predicted to be those specific modifications by kineticsTools.

S18D: Statistics on all putative modified cytosines in CpG dimers given minimum strand coverage levels

Min. Strand Cover-age	#CG = Number GCG > Min. Coverage	#CG as % of all CG in the genome	#modCG = Number of modified CG > Min. Coverage	#modCG as % of #CG	#4mCG = Number of 4-methyl CG > Min. Cover-age	#4mCG as % of #CG	#5mCG = Number of 5-methyl CG > Min. Coverage	#5mCG as % of #CG
3	20574879	98.8276	265810	1.2919	53713	0.2611	112978	0.5491
5	20242476	97.231	265491	1.3116	53713	0.2653	112919	0.5578
10	17288660	83.0429	255990	1.4807	52335	0.3027	107824	0.6237
15	11610868	55.7706	215423	1.8554	46055	0.3967	87656	0.7549
20	4973418	23.8889	123759	2.4884	28576	0.5746	47657	0.9582
25	1170657	5.623	39692	3.3906	9986	0.853	14609	1.2479
50	4195	0.0201	777	18.5221	309	7.3659	139	3.3135

*modCG is any CG flagged as containing a modified C and 4mCG of 5mCG are the subsets of modCG where the modification was predicted to be 4mC or 5mC by kineticsTools.

S18E: CpG methylation according to the more sensitive AgIn Algorithm:

CpG Methylation State	Count	Percent
0	10022667	93.6
1	689756	6.4

Blocks containing 6.4% of interrogated CpGs were not considered to be unmethylated. That indicates an upper limit of CpG methylation at 6.4%. This may be an overestimate since the algorithm calls “blocks” of CpGs as either methylated or unmethylated. Each block contains data from both strands of 40 or more consecutive CpGs and the methylation signals may come from only a subset therein. Nevertheless, the less sensitive approach that looked at individual Cs strand-specifically estimated 0.5-1% CpGs containing 5mC or 1.3-2.5% with modification signals in general. Thus, the algorithms together suggest 0.5-6.4%.

S18F: Statistics on all putative modified cytosines in GCG motif given minimum strand coverage level

Min. Strand Cover-age	#GCG = Number GCG ≥ Min. Coverage	#GCG as % of all GCG in the genome	#modGCG = Number of modified GCG ≥ Min. Coverage	#modGCG as % of #GCG	#4mC-GCG = Number of 4-methyl GCG ≥ Min. Cover-age	#4mC-GCG as % of #GCG	#5mC-GCG = Number of 5-methyl GCG ≥ Min. Coverage	#5mC-GCG as % of #GCG
3	3156626	98.9159	80487	2.5498	16971	0.5376	28215	0.8938
5	3113898	97.5769	80450	2.5836	16971	0.545	28213	0.906
10	2701392	84.6507	78284	2.8979	16680	0.6175	27123	1.004
15	1869990	58.5979	67593	3.6146	14997	0.802	22651	1.2113
20	819836	25.6903	39841	4.8596	9480	1.1563	12595	1.5363
25	193764	6.0718	12745	6.5776	3340	1.7237	3764	1.9426
50	945	0.0296	387	40.9524	103	10.8995	68	7.1958

*modGCG is any GCG flagged as containing a modified C and 4mC-GCG of 5mC-GCG are the subsets of modGCG where the modification was predicted to be 4mC or 5mC by kineticsTools.

Supplemental Table S19: Which dimers are observed with modifications more often than expected?

Modification	K	Position	Kmer	Observed	Expected	Chi-Square	P value	Std Residual
m4C	2	1	CA	29905	41657.1	57124.7	0	-57.6
m4C	2	1	CC	22323	19846.1	57124.7	0	17.6
**m4C**	**2**	**1**	**CG**	**53713**	**23404.2**	**57124.7**	**0**	**198.1**
m4C	2	1	CT	10014	31047.7	57124.7	0	-119.4
m5C	2	1	CA	86775	96267.5	88914.7	0	-30.6
m5C	2	1	CC	35919	45863.2	88914.7	0	-46.4
**m5C**	**2**	**1**	**CG**	**112978**	**54085.8**	**88914.7**	**0**	**253.2**
m5C	2	1	CT	32294	71749.5	88914.7	0	-147.3
m6A	2	1	AA	18023	25507.8	41495	0	-46.9
m6A	2	1	AC	12618	11545.7	41495	0	10
**m6A**	**2**	**1**	**AG**	**27676**	**9816.2**	**41495**	**0**	**180.3**
m6A	2	1	AT	8097	19544.3	41495	0	-81.9
modA	2	1	AA	69516	94871.6	114285.5	0	-82.3
modA	2	1	AC	43516	42942.3	114285.5	0	2.8
**modA**	**2**	**1**	**AG**	**94554**	**36509.7**	**114285.5**	**0**	**303.8**
modA	2	1	AT	39429	72691.5	114285.5	0	-123.4
modC	2	1	CA	185193	219419.3	225872.7	0	-73.1
modC	2	1	CC	89933	104534.5	225872.7	0	-45.2
**modC**	**2**	**1**	**CG**	**265810**	**123276**	**225872.7**	**0**	**406**
modC	2	1	CT	69830	163536.3	225872.7	0	-231.7

*modA is any adenine that was flagged as “modified_base” by kineticsTools and m6A is the subset of modA that was predicted to be 6mA by kineticsTools.
*modC is any cytosine that was flagged as “modified_base” by kineticsTools whereas m4C and m5C are subsets of modC predicted to specifically be 4mC and 5mC by kineticsTools.

Supplemental Table S20: Which trimers are observed with modifications more often than expected?

Modification	K	Positon	Kmer	Observed	Expected	Chi-Square	P value	Std Residual
m4C	3	2	ACA	4986	13691.2	104230.7	0	-74.4
m4C	3	2	ACC	3470	6221.9	104230.7	0	-34.9
m4C	3	2	ACG	9484	6881.9	104230.7	0	31.4
m4C	3	2	ACT	1146	9723	104230.7	0	-87
m4C	3	2	CCA	4156	7440.3	104230.7	0	-38.1
m4C	3	2	CCC	3582	3172.8	104230.7	0	7.3
m4C	3	2	CCG	6623	4181.2	104230.7	0	37.8
m4C	3	2	CCT	1669	5051.7	104230.7	0	-47.6
m4C	3	2	GCA	11349	7265.5	104230.7	0	47.9
m4C	3	2	GCC	9415	3911.2	104230.7	0	88
**m4C**	**3**	**2**	**GCG**	**16971**	**3587.5**	**104230.7**	**0**	**223.4**
m4C	3	2	GCT	4966	6028.6	104230.7	0	-13.7
m4C	3	2	TCA	9414	13260.2	104230.7	0	-33.4
m4C	3	2	TCC	5856	6540.2	104230.7	0	-8.5
m4C	3	2	TCG	20635	8753.6	104230.7	0	127
m4C	3	2	TCT	2233	10244.3	104230.7	0	-79.2
m5C	3	2	ACA	24931	31639.6	126488.4	0	-37.7
m5C	3	2	ACC	8954	14378.4	126488.4	0	-45.2
**m5C**	**3**	**2**	**ACG**	**31508**	**15903.7**	**126488.4**	**0**	**123.7**
m5C	3	2	ACT	5767	22469.4	126488.4	0	-111.4
m5C	3	2	CCA	12446	17194.1	126488.4	0	-36.2
m5C	3	2	CCC	8422	7332.2	126488.4	0	12.7
**m5C**	**3**	**2**	**CCG**	**23316**	**9662.6**	**126488.4**	**0**	**138.9**
m5C	3	2	CCT	10670	11674.3	126488.4	0	-9.3
m5C	3	2	GCA	26080	16790.2	126488.4	0	71.7
m5C	3	2	GCC	9145	9038.6	126488.4	0	1.1
**m5C**	**3**	**2**	**GCG**	**28215**	**8290.5**	**126488.4**	**0**	**218.8**
m5C	3	2	GCT	7396	13931.8	126488.4	0	-55.4
m5C	3	2	TCA	23318	30643.6	126488.4	0	-41.8
m5C	3	2	TCC	9398	15114	126488.4	0	-46.5
m5C	3	2	TCG	29939	20229	126488.4	0	68.3
m5C	3	2	TCT	8461	23674	126488.4	0	-98.9
m6A	3	2	AAA	2026	10597	160668.1	0	-83.3
m6A	3	2	AAC	2064	3995.7	160668.1	0	-30.6
m6A	3	2	AAG	3372	3383.1	160668.1	0	-0.2
m6A	3	2	AAT	711	7532	160668.1	0	-78.6
m6A	3	2	CAA	1150	4745.7	160668.1	0	-52.2
m6A	3	2	CAC	1055	2450.6	160668.1	0	-28.2
m6A	3	2	CAG	2649	2185.5	160668.1	0	9.9
m6A	3	2	CAT	1733	3788.8	160668.1	0	-33.4
**m6A**	**3**	**2**	**GAA**	**12857**	**4940.6**	**160668.1**	**0**	**112.6**
**m6A**	**3**	**2**	**GAC**	**7147**	**2136.1**	**160668.1**	**0**	**108.4**
**m6A**	**3**	**2**	**GAG**	**16464**	**1926.6**	**160668.1**	**0**	**331.2**
m6A	3	2	GAT	3828	3263.4	160668.1	0	9.9
m6A	3	2	TAA	1990	5224.5	160668.1	0	-44.7
m6A	3	2	TAC	2352	2963.3	160668.1	0	-11.2
m6A	3	2	TAG	5191	2321.1	160668.1	0	59.6
m6A	3	2	TAT	1825	4960.1	160668.1	0	-44.5

*modA is any adenine that was flagged as “modified_base” by kineticsTools and m6A is the subset of modA that was predicted to be 6mA by kineticsTools.
*modC is any cytosine that was flagged as “modified_base” by kineticsTools whereas m4C and m5C are subsets of modC predicted to specifically be 4mC and 5mC by kineticsTools.

Supplemental Table S21 A-F: Binomial tests for enrichment or depletion of DNA modifications in various genomic features.

S21A: Do gene regions/features as defined by the Maker2 Annotation have higher or lower modification rates than the genome-wide rate?

MAKER2 GENE FEATURES	MODIFICATION	PERCENT MODIFIED ACROSS GENOME	PERCENT MODIFIED ACROSS FEATURE	BINOMIAL P-VALUE FOR ENRICHMENT	BINOMIAL P-VALUE FOR DEPLETION
GENES	6mA	0.0355	0.0352	0.98128157	0.018956188
	modA	0.1322	0.1316	0.964203427	0.036007625
	4mC	0.1124	0.1108	0.999929591	7.15E-05
	5mC	0.2598	0.2589	0.910102733	0.090312522
	modC	0.5921	0.5902	0.970909305	0.029203296
EXONS	6mA	0.0355	0.0329	1	7.54E-13
	modA	0.1322	0.1278	0.999999999	6.76E-10
	4mC	0.1124	0.0984	1	1.02E-66
	5mC	0.2598	0.2572	0.978871468	0.021377372
	modC	0.5921	0.5689	1	4.71E-35
INTRONS	6mA	0.0355	0.0358	0.072865683	0.927941142
	modA	0.1322	0.1327	0.106287986	0.894268744
	4mC	0.1124	0.1154	1.87E-09	0.999999998
	5mC	0.2598	0.2595	0.627989008	0.373145715
	modC	0.5921	0.5983	5.98E-08	0.999999941
PROMOTERS	6mA	0.0355	0.0332	0.999509543	0.000527151
	modA	0.1322	0.1228	1	1.76E-12
	4mC	0.1124	0.1052	0.999990523	1.02E-05
	5mC	0.2598	0.2504	0.999866666	0.000138694
	modC	0.5921	0.5681	1	3.02E-10

S21B: Are gene regions/features as defined by the Maker2 Annotation enriched with or depleted for DNA modifications with respect to the prevalence of those features in the genome?

MAKER2 GENE FEATURES	MODIFICATION	PERCENT OF MODIFICATIONS EXPECTED TO BE IN REGION AT RANDOM	OBSERVED PERCENT OF MODIFICATIONS IN REGION	BINOMIAL P-VALUE FOR ENRICHMENT	BINOMIAL P-VALUE FOR DEPLETION
GENES	6mA	56.1611602	56.2185684	0.38433787	0.61864674
	modA	56.1611602	56.5528409	4.38E-05	0.9999569
	4mC	56.1611602	56.6038549	0.00119808	0.99882524
	5mC	56.1611602	57.2524126	2.26E-30	1
	modC	56.1611602	57.2653029	3.71E-68	1
EXONS	6mA	13.6878976	12.1088927	1	1.18E-33
	modA	13.6878976	12.6514584	1	3.93E-52
	4mC	13.6878976	13.8381269	0.06906262	0.93206208
	5mC	13.6878976	15.6490002	6.89E-185	1
	modC	13.6878976	15.1847025	7.82E-247	1
INTRONS	6mA	42.4732626	44.1096757	8.38E-18	1
	modA	42.4732626	43.9013825	6.39E-47	1
	4mC	42.4732626	42.7657281	0.02214394	0.97816755
	5mC	42.4732626	41.6034124	1	3.91E-20
	modC	42.4732626	42.0806004	1	2.66E-10
PROMOTERS	6mA	3.68304918	3.5655133	0.94778728	0.05447281
	modA	3.68304918	3.54189017	0.99991225	9.16E-05
	4mC	3.68304918	3.48583502	0.99984234	0.00016757
	5mC	3.68304918	3.59038087	0.99477972	0.00537773
	modC	3.68304918	3.57370908	0.99999746	2.63E-06

S21C: Do gene regions/features as defined by StringTie have higher DNA modification rates with respect to the genome-wide average?

STRINGTIE GENE FEATURES	MODIFICATION	PERCENT MODIFIED ACROSS GENOME	PERCENT MODIFIED ACROSS FEATURE	BINOMIAL P-VALUE FOR ENRICHMENT	BINOMIAL P-VALUE FOR DEPLETION
**GENES**	6mA	0.0355	0.0351	0.99619467	0.003862711
	modA	0.1322	0.1314	0.991948166	8.11E-03
	4mC	0.1124	0.111	0.999449561	5.58E-04
	5mC	0.2598	0.2589	0.920825033	0.079543767
	modC	0.5921	0.5915	0.734783388	0.265759097
**EXONS**	6mA	0.0355	0.0337	0.999999995	5.03E-09
	modA	0.1322	0.1291	0.999999449	5.65E-07
	4mC	0.1124	0.1023	1	2.42E-43
	5mC	0.2598	0.2599	0.448100039	0.553623203
	modC	0.5921	0.5812	1	5.51E-11
**INTRONS**	6mA	0.0355	0.0356	0.316477725	0.685634126
	modA	0.1322	0.1323	0.400622005	0.600569585
	4mC	0.1124	0.1153	2.46E-08	0.999999976
	5mC	0.2598	0.2584	0.965035978	0.035199744
	modC	0.5921	0.5966	0.000100661	0.999900138
**PROMOTERS**	6mA	0.0355	0.0341	0.978301218	0.022776113
	modA	0.1322	0.1238	1	3.30E-10
	4mC	0.1124	0.108	0.995606095	0.004597575
	5mC	0.2598	0.247	0.999999654	3.64E-07
	modC	0.5921	0.568	1	3.02E-10

S21D: Are there higher or lower DNA modification rates in genes or promoter regions when partitioned by gene expression?

MAKER2 GENES AND PROMOTERS	MODIFICATION	PERCENT MODIFIED ACROSS GENOME	PERCENT MODIFIED ACROSS FEATURE	BINOMIAL P-VALUE FOR ENRICHMENT	BINOMIAL P-VALUE FOR DEPLETION
**GENES: HIGHLY EXPRESSED**	6mA	0.0355	0.0346	0.999093866	0.000932321
	modA	0.1322	0.1302	0.9997053	0.00029946
	4mC	0.1124	0.1085	0.999999992	8.78E-09
	5mC	0.2598	0.2572	0.993766357	0.006306024
	modC	0.5921	0.587	0.999332119	0.000674214
**GENES: LOWLY EXPRESSED**	6mA	0.0355	0.036	0.051184353	0.949577622
	modA	0.1322	0.1336	0.0021631	0.997862533
	4mC	0.1124	0.1145	0.000372393	0.999635009
	5mC	0.2598	0.2613	0.059873187	0.940559983
	modC	0.5921	0.6003	6.68E-09	0.999999993
**GENES: NOT EXPRESSED**	6mA	0.0355	0.0339	0.99990464	0.000100206
	modA	0.1322	0.1289	0.999953104	4.82E-05
	4mC	0.1124	0.1059	1	2.13E-10
	5mC	0.2598	0.2562	0.98919457	0.010982399
	modC	0.5921	0.5703	1	2.70E-20
**PROMOTERS: HIGHLY EXPRESSED**	6mA	0.0355	0.0341	0.888360539	0.118420887
	modA	0.1322	0.1211	0.99999906	1.03E-06
	4mC	0.1124	0.1066	0.977702806	0.023739974
	5mC	0.2598	0.2423	0.999968501	3.39E-05
	modC	0.5921	0.5625	0.999996261	3.95E-06
**PROMOTERS: LOWLY EXPRESSED**	6mA	0.0355	0.0335	0.952665437	0.050871358
	modA	0.1322	0.1234	0.999932439	7.27E-05
	4mC	0.1124	0.1073	0.959218121	0.043182619
	5mC	0.2598	0.2562	0.792770969	0.212254363
	modC	0.5921	0.5767	0.988893271	0.011450768
**PROMOTERS: NOT EXPRESSED**	6mA	0.0355	0.0322	0.997137664	0.00319297
	modA	0.1322	0.1242	0.999649671	0.000374566
	4mC	0.1124	0.1007	0.999966509	3.76E-05
	5mC	0.2598	0.2532	0.927537438	0.074943257
	modC	0.5921	0.5649	0.999967849	3.38E-05

S21E: Do regions of the genome annotated as repetitive or the unique regions have higher or lower modification rates than the genome-wide average?

REGIONS DEFINED BY REPEAT MASKER	MODIFICATION	PERCENT MODIFIED ACROSS GENOME	PERCENT MODIFIED ACROSS FEATURE	BINOMIAL P-VALUE FOR ENRICHMENT	BINOMIAL P-VALUE FOR DEPLETION
REPEATS	6mA	0.0355	0.0411	1.14E-137	1
	modA	0.1322	0.1496	0	1
	4mC	0.1124	0.1294	1.34E-231	1
	5mC	0.2598	0.2902	0	1
	modC	0.5921	0.6561	0	1
UNIQUE	6mA	0.0355	0.0318	1	1.47E-100
	modA	0.1322	0.1206	1	1.62E-259
	4mC	0.1124	0.1003	1	4.29E-181
	5mC	0.2598	0.238	1	4.87E-249
	modC	0.5921	0.5463	1	0

* Note: the unique regions here are regions not labeled as repeats by RepeatMasker. In other words, the assembly was partitioned into repeat and unique regions.

S20F: Is there enrichment or depletion of DNA modifications in the regions of the genome annotated as repetitive or the unique regions?

REGIONS DEFINED BY REPEAT MASKER	MODIFICATION	PERCENT OF MODIFICATIONS EXPECTED TO BE IN REGION AT RANDOM	OBSERVED PERCENT OF MODIFICATIONS IN REGION	BINOMIAL P-VALUE FOR ENRICHMENT	BINOMIAL P-VALUE FOR DEPLETION
REPEATS	6mA	39.5417134	46.2613304	1.26E-270	1
	modA	39.5417134	45.263648	0	1
	4mC	39.5417134	48.0281144	0	1
	5mC	39.5417134	46.6130031	0	1
	modC	39.5417134	46.2325997	0	1
UNIQUE	6mA	60.4582866	53.7386696	1	1.26E-270
	modA	60.4582866	54.736352	1	0
	4mC	60.4582866	51.9718856	1	0
	5mC	60.4582866	53.3869969	1	0
	modC	60.4582866	53.7674003	1	0

Section 3: Detailed Experimental Methods

3.1 Embryo Collection

Sciara is 'monogenic': the females produce either only male or only female offspring. Male-producing females have 2 copies of the X chromosome while female-producing females have an X and an X'. The two types of females can be differentiated when they are adult flies based on the wavy wing phenotypic marker that is associated with the X': female-producers have wavy wings, whereas male-producers have normal straight wings. However, the different females cannot be distinguished as embryos, our primary target life stage for sequencing. Therefore, we mated males strictly with male-producing females for male-only embryo collection to avoid sequencing the X' in some female embryos that can complicate the assembly. Mass matings consisted of 6 X'X female and 4 XO male flies per vial. One day after combining the flies for mating, females were separated from males. Embryo laying was induced by squishing the thorax with forceps and plating on 2.2% bactoagar (2.2g per 100 mL). After 2-4 hours, adults were removed from the plate and embryos were transferred to an antibiotic/antimycotic plate, where they were incubated at 20-21˚C for up to 2 days, unless early (2-4 hour embryos) were being collected. Prior to DNA extraction, embryos were washed in TE (10 mM Tris-HCl pH 8, 1 mM EDTA) serially 10 times to remove external contamination.

3.2 DNA Extraction

Genomic DNA (gDNA) was isolated from *Sciara* using DNAzol (ThermoFisher) and following the manufacturer's instructions with some modifications. Either a glass dounce homogenizer or a blue pestle was used each with 10 strokes for the homogenization step. Prior to precipitating the DNA, 5 ul RNase A Solution (Qiagen, 4 mg/mL) was added to the DNA sample and incubated at 37˚C for 10 minutes, followed by 5 ul Puregene Proteinase K (Qiagen, 20 mg/mL), incubated at 37˚C for 10 minutes. After adding 100% ethanol, the tube was slowly inverted 50 times, incubated at room temperature for 2 minutes followed by ice for 2 minutes, then centrifuged at 18000g for 10 minutes. The supernatant was removed. The pellet was washed twice with 75% ethanol, very briefly air-dried, and re-suspended in TE. The gDNA was cleaned with AMPure beads (Beckman Coulter) until NanoDrop reported purity levels of A60/280 > 1.8 and A260/230 of ~2.0. It was re-suspended in Tris-HCl (pH 8.0) before beginning library preparations.

3.3 Illumina PE Genomic DNA library

gDNA from mixed stage embryos (2 hour - 2 day old) was sonicated to a size range of 100-600 bp, and the Illumina library were prepared using the NEBNext kit (New England Biolabs) following the manufacturer's directions. The library was run on a 2% NuSieve agarose (Lonza) gel, size-selected near the 500 bp marker, gel purified (Qiagen), and sequenced on the Illumina HiSeq 2000 platform to obtain 100 bp paired-end reads.

3.4 PacBio sequencing details

For Pacific Biosciences RSII Single Molecule Real Time sequencing datasets (P5-C3 chemistry), male embryo gDNA was brought to the Technology Development Group at the Institute of Genomics & Multiscale Biology at the Icahn School of Medicine at Mount Sinai for library construction and sequencing. There, two DNA libraries were prepared and sequenced according to the manufacturer's instructions, reflecting the P5-C3 sequencing enzyme and chemistry, respectively. For each library, 6 µg of extracted, high-quality, genomic DNA isolated from *Sciara coprophila* was diluted in Qiagen elution buffer to 150 µL. The 150 µL aliquots were

individually pipetted into the top chambers of Covaris G-tube spin columns and sheared gently for 60 seconds at 4500 rpm using an Eppendorf 5424 benchtop centrifuge and repeated at 60 seconds at 4500 rpm to further shear the DNA and place the aliquot back into the upper chamber, resulting in a ~20,000 bp DNA shear, verified using a DNA 12000 Agilent Bioanalyzer gel chip. The sheared DNA was then re-purified using a 0.45X AMPure XP purification step (0.45X AMPure beads added, by volume, to each DNA sample dissolved in 200 µL EB, vortexed for 10 minutes at 2,000 rpm, followed by two washes with 70% alcohol and finally diluted in EB).

After purification, 2.7 µg of purified and sheared sample was taken into the DNA damage and end-repair steps. Briefly, the DNA fragments were repaired using DNA Damage Repair solution (1X DNA Damage Repair Buffer, 1X NAD+, 1 mM ATP high, 0.1 mM dNTP, and 1X DNA Damage Repair Mix) with a volume of 21.1 µL and incubated at 37˚C for 20 minutes. DNA ends were repaired next by adding 1X End Repair Mix to the solution, which was incubated at 25˚C for 5 minutes followed by the second 0.45X Ampure XP purification step. Next, 0.75 µM of Blunt Adapter was added to the DNA followed by 1X template Prep Buffer, 0.05 mM ATP low and 0.75 U/µL T4 ligase to ligate (final volume of 47.5 µL) the SMRTbell adapters to the DNA fragments. This solution was incubated at 25˚C overnight followed by a 65˚C 10-minute ligase denaturation step. After ligation, the library was treated with an exonuclease cocktail to remove un-ligated DNA fragments using a solution of 1.81 U/µL Exo III 18 and 0.18 U/µL Exo VII, then incubated at 37˚C for 1 hour. Two additional 0.45X Ampure XP purifications steps were performed to remove <2000 bp molecular weight DNA and organic contaminant.

Upon completion of library construction, the library was validated as ~20 kb using another Agilent DNA 12000 gel chip. All libraries were sufficient for additional size selection to remove any library molecules < 7,000 bp. This step was conducted using Sage Science Blue Pippin 0.75% agarose cassettes to select library in the range of 7,000-50,000 bp. 16% of the input library eluted from the agarose cassette and was available for sequencing. This yield was sufficient to proceed to primer annealing and DNA sequencing on the PacBio RSII machine. Size-selection was confirmed by Bio-Analysis and the mass was quantified using Qubit. Primer was then annealed to the size-selected SMRTbell with the full-length libraries (80˚C for 2 minute 30 seconds followed by decreasing the temperature by 0.1˚ to 25C˚). The polymerase-template complex was then bound to the P5 enzyme using a ratio of 10:1 polymerase to SMRTbell at 0.5 nM for 4 hours at 30˚C and then held at 4˚C until ready for magbead loading, prior to sequencing. The magnetic bead-loading step was conducted at 4˚C for 60-minutes per manufacturer's guidelines. The magbead-loaded, polymerase-bound, SMRTbell libraries were placed onto the RSII machine at a sequencing concentration of 75-100 pM across 24 SMRTcells and configured for 180-minute continuous sequencing runs on each SMRTcell. Sequencing was conducted to ample coverage across 24 SMRTcells.

3.5 MinION sequencing details

We followed similar protocols as reported in our earlier bioRxiv preprint (Urban et al. 2015). There we described our first attempts at sequencing *Sciara* genomic DNA using modifications to the standard protocol that required no additional reagents or equipment and reported that the majority of our data came from reads > 10 kb with many exceeding 50 kb and a high quality 2D read that exceeded 100 kb. In addition to those early datasets, our MinION data were collected over the course of 18 months and span multiple kits (MAP002-MAP006), flow cell/pore (R7.3 – R7.3 70bps 6mer), software, and MinION upgrades (original, MkI). As the ONT protocols changed, we needed to adapt our modifications from that preprint. However, the principles and results of our protocols have stayed the same (e.g. see Supplemental Figures S4 and S5).

The principles we apply to our modified protocols are the following:

(1) Start out with more DNA than required for the standard protocol that assumes 8 kb molecules to target similar molarities,

(2) Skip the Covaris shearing step to keep DNA long,

(3) Always perform a DNA repair step to repair damaged bases and single-stranded nicks,

(4) Use wide-bored tips and very gentle pipetting throughout the protocol,

(5) Use 0.4x ratio of AMPure beads in all clean-up steps,

(6) Add a rinse step before elution of AMPure beads to deplete DNA < 10-12 kb in all clean-up steps, and

(7) Elute DNA off the AMPure beads while adding heat into the system (37-50˚C) for extended periods of time (10-20 minutes) in all clean-up steps.

(8) It is also important to minimize the amount of handling needed, which has been facilitated by updates to the standard protocol by combining End-Repair and dA-tailing into one step.

Reagents, MinION versions, pore models, and protocols for each library:

In total, we prepared 17 libraries for this project. The libraries are named/numbered 01-09, 11-16, 20-21. In general, library preparations have the general workflow of optional shearing, DNA repair, End repair, dA-tailing, adapter ligation, and hairpin enrichment in some cases with various clean up steps in between.

Library 01 used SQK-MAP002 reagents. Libraries 02-08 used SQK-MAP004 reagents. Libraries 09, 11, 12, and 13 used SQK-MAP005 reagents. All aforementioned libraries used the original MinION and the R7.3 pore model. Libraries 14, 15, 16, 20, and 21 used SQK-MAP006 reagents, the MinION MkI, and the R7.3 70 bps 6mer model. Libraries 14, 20, and 21 were constructed following Oxford Nanopore's standard protocol. Libraries 02-09, 11-13, and 15-16 were constructed using modifications from above as described below.

Optional Shearing:

Libraries 14, 20, and 21 followed the standard protocol, and shearing to ~8 kb was performed in Covaris g-TUBEs as instructed.

For libraries prepared with modified protocols (01-09, 11-13, 15-16), shearing was always skipped and we started out with 2.2-17 μg, using the lower end for early libraries (2.2-5 μg in 01-13) and the higher amounts more recent libraries (15-17 μg in 15-16).

DNA Repair:

All libraries except 01 were subject to DNA repair steps. PreCR (NEB) was performed on libraries 02-09 and 11-13. FFPE Repair (NEB) was performed on libraries 14-16 and 20-21.

End repair and dA-tailing:

For most early libraries prior to MAP006 (excluding 01 and 03), End-Repair and dA-tailing were done in separate steps using the NEBNext End-Repair Module (NEB) and the NEBNext dA-Tailing Module (NEB). For libraries 01 and 03, these steps were combined using the NEBNext Ultra End Repair/dA-tailing module (NEB). For all libraries using MAP006 MinION reagents, end repair and dA-tailing were performed as a single step using the NEBNext Ultra II End Repair/dA-tailing module (NEB).

Adapter ligation:

Ligation was always carried out using MinION kit-specific adapters and volumes with Blunt/TA Ligase Master Mix (NEB). We only needed to manually add the tether and motor proteins

for library 01. In all others, the tether and motor proteins were pre-combined with other reagents such as the adapters and elution buffer. The first library did not have a hairpin enrichment step, but all other libraries did concurrent with MinION kits and protocols.

Hairpin enrichment:

Seven libraries (02-08) used His-beads (Dynabeads His-tag Isolation and Pulldown; Life Technologies) with MAP004 reagents and four libraries (09, 11-13) used His-beads with MAP005 reagents according to Oxford Nanopore's instructions. The five MAP006 libraries (14-16, 20-21) used MyOne C1 streptavidin beads (Dynabeads/Thermofisher) for hairpin enrichment following Oxford Nanopore's protocol.

AMPure clean up notes:

Libraries 07, 08, 09, and 15 included rinses in all clean-up steps (Urban et al. 2015) whereas the other libraries did not. Clean up steps for all modified protocols used a 0.4x AMPure ratio. For all AMPure beads steps for our modified protocols, DNA was eluted off the beads by incubating at 37˚C for 20 minutes in libraries 01-09 and 11-13, and at 50˚C for 10 minutes in libraries 15-16. For standard protocols, DNA was eluted off beads following Oxford Nanopore's protocol. Wide-bored tips and gentle pipetting were used throughout.

Sequencing notes:

Sequencing was conducted following standard procedures. For early libraries, smaller amounts of DNA were loaded more frequently (e.g. 4 times) throughout the run. For more recent libraries, half of the library was loaded at the beginning and the second half was added 24 hours in.

Basecalling notes:

Oxford Nanopore's Metrichor 2d basecaller version 1.10.2 was used for libraries 1-8, version 10.13.1 for library 9, version 1.14.4 for library 11, version 1.19.0 for libraries 12-16, and version 1.20.0 for libraries 20-21.

Performance notes:

Libraries 04, 05, 09, and 11 did not perform well due to flow cells with very limited numbers of pores available for sequencing and consequentially gave little data, but we included them anyway.

Library 11 was attempted on two different flow cells that both started with very few available pores. Moreover, though fragmentation was not performed, the DNA was lower molecular weight than expected as viewed on an agarose gel.

Libraries 06, 07, 08, 09, 12, 13 and 14 were all prepared from the same DNA source to be able to directly compare size distributions after different protocols. Similarly, libraries 15 and 16 were prepared from the same source to make direct comparisons of the effects of including rinse steps in the AMPure clean-ups. When comparing modified protocols without rinse steps to the standard protocols, our modified protocols were enriched for reads >10 kb. Modified protocols that also included rinse steps were additionally enriched over all other protocols for reads >10 kb.

The standard protocol for three libraries gave a combined molecule N50 of 6.9 kb and 2D read N50 of 8.8 kb in agreement with the target of 8 kb molecules. Our modified protocols produced molecule N50s and 2D N50s of 19.3 kb and 18.4 kb respectively, over double that from the standard protocol. MarginStats (Jain et al. 2015) was used to calculate the percent identities of MinION reads aligned to a PacBio genome assembly (details in bioinformatics section below). We obtained ultra-long (>100 kb) MinION reads map to PacBio-only genome assemblies with up to 91% identity across the entire length. For example, we obtained 2D reads of lengths 131.5 kb

(Q=10.3), 111.2 kb (Q=9.9), 105 kb (Q=9.3), and 100.7 kb (Q=10.4) that aligned across their full lengths with identities of 91.1%, 88.7%, 63.2%, and 84.2%. The 102.9 kb 2D read that we previously reported as high quality (Q=8.74) (Urban et al. 2015) aligned in full at 84.2% identity, representing a high quality, ultra-long 2D read derived from early MinION sequencing reagents (MAP004). For all nanopore reads, 85.7% of 2D reads and 57.9% of 1D reads aligned to a high quality PacBio-only assembly from Canu (Koren et al. 2017). The median percent identities for aligned 1D and 2D reads was 68% and 82.1%, respectively, with both having examples with >95% identity. Percent identities for both 2D and 1D reads were correlated with mean quality scores (Spearman's rho for 2D = 0.76 and for 1D = 0.70). There were some exceptionally long 1D reads that exceed 200 kb, but those were strictly of low quality and low percent-identity.

3.6 BioNano Irys optical mapping details

To isolate ultra high molecular weight DNA, male pupae were flash frozen and ground in liquid nitrogen. The powder was resuspended in Nuclear Isolation Buffer (NIB)(10 mM Tris pH 9.4, 60 mM NaCl, 10 mM EDTA, 0.15 mM spermidine, 0.15 mM spermine 0.5% Triton-X 100, 1% beta-mercaptoethanol) on ice and filtered through 100 µM mesh cell strainer. Cellular debris was removed by sedimentation for 15 sec at 1K rpm, 4˚C. Nuclei were recovered from the supernatant by sedimentation for 3 minutes at 1800xg, 4˚C. Nuclei washed 3x with NIB, were resuspended in Cell Suspension Buffer (Chef Mammalian Genomic DNA plug kit, Bio Rad) and embedded in low melt agarose (BioRad), final concentration 0.8%. Nuclei were lysed in Alternate Lysis Buffer (BioNano Genomics) containing 1.6 mg Proteinase K (Qiagen) at 50˚C for 24 hr, and RNA degraded by addition of RNAse A (80 µg, Qiagen). Plugs were liquefied using Gelase (Epicentre) and the resulting high molecular weight DNA was membrane dialyzed against 10mM Tris HCL, 1mM EDTA, pH 8.0). DNA was harvested with wide bore tips and stored at 4˚C.

High Molecular Weight (HMW) DNA was nicked, labeled and repaired according to the IrysPrep protocol (BioNano Genomics). In brief, HMW DNA was digested with the single-stranded nicking endonuclease BssSI (CACGAG, NewEngland BioLabs). Fluorescently labeled nucleotides were incorporated by nick translation. The backbone of the labeled DNA was stained with YOYO-1. Individual labeled DNA molecules were imaged on the Irys platform (BioNano Genomics). Images of 438,139 molecules >100 kb (N50 = 173.8 kb) were collected: 217,194 of these were >150 kb (N50 = 214.1 kb).

3.7 Strand-specific RNA-seq details

Total RNA from male and female embryos, larvae, pupae, and adult flies was extracted using TRIzol (Invitrogen/Thermofisher). RNA quantity and purity were measured with the NanoDrop (ThermoScientific) and Qubit (ThermoFisher). Total RNA was treated with DNase (Qiagen) and subject to RNeasy column clean up (Qiagen). The cleaned total RNA quantity and purity were checked using the NanoDrop and Qubit. RNA integrity was evaluated on 1.1% formaldehyde 1.2% agarose gels. Poly-A RNA was enriched using Oligo-dT DynaBeads (LifeTechnologies). Qubit was used to measure the quantity of poly-A RNA. Poly-A RNA was fragmented with NEB's Magnesium Fragmentation Module for 3 minutes at 94˚C, which was selected after optimizing for conditions for 200-500 bp fragments as determined by the Bioanalyzer. Fragmentation reactions were cleaned up with RNeasy columns. First strand synthesis was performed with SSIII (Invitrogen). Briefly, 1 µl Random Primer (3 µg/µl), 1 µ 10 mM dNTP mix, and 10 µl fragmented RNA were incubated at 65˚C for 5-10 minutes and transferred to ice for 5 minutes. A mix of 4 µl 5X First Strand Synthesis (FSS) Buffer, 1 µl 0.1 M DTT, 1 µl Murine RNase Inhibitor, 1 µl 0.5 µg/µl Actinomycin D, and 1 µl SSIII (200 units) was added to the mixture of RNA, dNTPs, and Random Primers. This was incubated in the thermocycler at 25˚C

for 5 minutes to anneal the random primers, 50˚C for 60 minutes to extend from the random primers, and 70˚C for 15 minutes to inactivate SSIII. The reaction was cleaned up with AMPure beads using a ratio of 2.0.

For Second Strand Synthesis (SSS), a mixture of 10 mM each of dATP, dCTP, dGTP, and 20 mM of dUTP (instead of dTTP) was made. For a single reaction, 64 µl of cleaned FSS cDNA:RNA in Ultra Pure Water, was combined with 4 µl ACGU mix, 8 µl of NEB dNTP-free SSS Reaction buffer and 4 µl NEB SSS Enzyme mix from the SSS module. The reaction was incubated for 1 hour at 16˚C, then cleaned with AMPure beads using a 1.0x ratio to begin eliminating DNA smaller than 200 bp, and quantified with the Qubit. There was typically 100-200 ng at this step.

The double-stranded cDNA was then subject to End Repair (NEBNext), and cleaned with AMPure beads using a 0.9x ratio to deplete DNA < 300 bp. The End-Repaired cDNA was then dA-tailed (NEBNext), and cleaned with AMPure beads using a 0.9x ratio. For adapter ligation, 38 µl of DNA was combined with 10 µl 5X NEBNext Quick Ligation Reaction Buffer, 1 µl NEB Adaptor, and 2 µl Quick T4 Ligase. The reaction was incubated at room temperature for 15 minutes, then cleaned with a 0.9x ratio of AMPure beads.

The library was then size-selected with AMPure beads before proceeding to the PCR step. To obtain adapter-ligated fragments in the 300-600 bp range, the DNA was first incubated with 0.6x AMPure beads by adding 60 µl AMPure beads to 100 µl DNA in UPW. The beads were pelleted on a magnet and the supernatant containing DNA smaller than approximately 600 bp was transferred to a new tube (DNA longer than 600 bp stayed on the beads). To make the final ratio 0.9x to select for DNA > 300 bp on the beads, 30 µl more AMPure beads was added to the 160 µl supernatant. From there the AMPure clean-up proceeded as normal.

USER enzyme digestion to cut the DNA at uracils (in the second strand and in the hairpin adapters) and PCR were then performed as follows: 20 µl of cleaned DNA was combined with 25 µl NEBNext High-Fidelity 2X PCR Master Mix and 3 µl NEBNext USER enzyme. This reaction was incubated for 15 minutes at 37˚C to ensure uracil cutting occurs before addition of primers. Then 1 µl indexed primer (NEBNext) and 1 µl universal primer were added. The reaction was put in the thermocycler for 37˚C for 15 minutes to ensure USER digestion went to completion, followed by 98˚C for 30 seconds and 12 cycles of: 98˚C for 10 seconds, 65˚C for 30 seconds, 72˚C for 30 seconds. The PCR products at this stage are approximately 122 bp longer than the target insert size. We adjusted AMPure ratios accordingly for a final clean up and size-selection.

The PCR reactions were cleaned with 0.85x AMPure beads to deplete DNA smaller than 350 bp. The DNA was eluted in 100 µl UPW and AMPure size selection was initiated by incubating with 55 µl AMPure beads (0.55x) to precipitate DNA longer than 650 bp onto the beads. The beads were pelleted on a magnet and the 155 ul of DNA shorter than 650 bp in the supernatant was transferred to a new tube where another 30 µl of AMPure beads was added for a final ratio of 0.85x. The AMPure procedure then continued as normal to obtain DNA > 350 bp. The estimated insert sizes at this step was 230-530 bp. DNA samples were quantified with Qubit and purity was measure by NanoDrop. There was typically 600 ng at the end of this protocol. Bioanalyzer traces suggested the mean estimated fragment sizes was around 420 bp putting the mean insert sizes near 300 bp.

Section 4: Detailed Bioinformatics Methods

Note: for specific software versions used, see Section 5 below.

4.1 Illumina Assemblies

We obtained ~103x coverage of paired-end Illumina HiSeq 2000 data from a ~430 bp insert genomic DNA library. This data was assembled using several popular genome assemblers: ABySS (Simpson et al. 2009), Megahit (Li et al. 2015), Platanus (Kajitani et al. 2014), SGA (Simpson and Durbin 2010), SOAPdenovo2 (Luo et al. 2012), SPAdes (Bankevich et al. 2012), and Velvet (Zerbino and Birney 2008). We also attempted to use MaSuRCA (Zimin et al. 2013). However, after several attempts that failed due to exceeding allotted time limits, with the final attempt given >8 days, we decided to not pursue it further. For all other assemblers, we tried assembling the raw data as well as a read set that was quality-filtered using Trimmomatic (Bolger et al. 2014). Moreover, SPAdes and SGA both error-correct the input reads followed by assembling the error-corrected reads. Therefore, we also tried the error-corrected raw reads and error-corrected quality-filtered reads produced by BayesHammer (Nikolenko et al. 2013) in the SPAdes pipeline with the other assemblers. Finally, some assemblers, such as Megahit, Platanus, and SPAdes, employ pipelines that iterate over multiple k-mer sizes whereas others, such as Velvet, SOAPdenovo2, and Abyss, require a value of K to be selected. Therefore, we tried K=55 and K=77 for each set of reads for the latter set of assemblers.

4.1.1 Inputs

For most assemblers, we tried using all reads, all reads after BayesHammer (Nikolenko et al. 2013) from SPAdes (Bankevich et al. 2012) correction, filtered reads using Trimmomatic (Bolger et al. 2014), and filtered reads that were subsequently corrected with BayesHammer. Note that the BayesHammer corrected reads were produced as part of running SPAdes.

4.1.1.1 Trimming and quality filtering

For trimming, we used Trimmomatic version 0.32:

```
IN="MSF0007_TGACCA_L004_R1_001.fastq.gz
MSF0007_TGACCA_L004_R2_001.fastq.gz"
OUTPRE=maleSciaraTrimmed
OUT="${OUTPRE}_forward_paired.fq.gz ${OUTPRE}_forward_unpaired.fq.gz
${OUTPRE}_reverse_paired.fq.gz ${OUTPRE}_reverse_unpaired.fq.gz"
PRIMERS=neb_primers.fa
MINQUAL=5
MINLEN=100

java -jar $TRIMMOMATIC_BASE/Trimmomatic-0.32/trimmomatic-0.32.jar PE -
trimlog trim.log $IN $OUT ILLUMINACLIP:${PRIMERS}:2:30:10
LEADING:$MINQUAL TRAILING:$MINQUAL SLIDINGWINDOW:4:$MINQUAL
MINLEN:$MINLEN
```

Trimming produced three files:
One file each for the forward and reverse reads that remained paired and one file containing the orphaned reads. In addition to the paired reads, the orphaned reads were used with most assemblers.

4.1.1.2 Short read error-correction

SPAdes runs BayesHammer error-correction as part of its pipeline. The error-corrected reads are stored in three files: forward reads (R1), reverse reads (R2), and orphaned reads (SE). We used these error-corrected reads with other assemblers as described.

4.1.1.3 Naming of short read datasets used in assemblies

Where relevant below:

R1	= Left read/read_1 from paired-end (PE) reads
R2	= Right read/read_2 from PE reads
SE	= reads that were orphaned during quality filtering or error-correction
q5	= Trimmomatic quality-filtered
bh	= BayesHammer error-corrected
cf	= Contamination filtered (cf)
gz	= gzipped extension
fq	= altenrative suffix for fastq

<u>Short Read Set #1</u>
R1.fastq(.gz) and R2.fastq(.gz)
= the set of all PE reads - no quality filtering and no error-correction

<u>Short Read Set #2</u>
R1.q5.fastq(.gz), R2.q5.fastq(.gz), and SE.q5.fastq(.gz)
= the quality-filtered reads from Trimmomatic

<u>Short Read Set #3</u>
R1.bh.fastq, R2.bh.fastq, and SE.bh.fastq
= the BayesHammer-corrected reads (using all reads and no quality filtering)

<u>Short Read Set #4</u>
R1.q5.bh.fastq, R2.q5.bh.fastq, and SE.q5.bh.fastq
= the BayesHammer-corrected quality-filtered reads (quality-filtering and bayeshammer orphans all in same SE file)

<u>Short Read Set #5</u>
R1.cf1.fastq and R2.cf1.fastq
= the set of all reads after contamination filtering

<u>Short Read Set #6</u>
R1.cf1.q5.fastq, R2. cf1.q5. fastq, and SE. cf1.q5.fastq
= the set of all reads after contamination filtering that were subsequently quality-filtered

<u>Short Read Set #7</u>
R1.cf1.bh.fastq, R2. cf1.bh.fastq, and SE.cf1.bh.fastq
= the set of all reads after contamination filtering that were subsequently error-corrected

<u>Short Read Set #8</u>
R1.cf1.q5.bh.fastq, R2.cf1.q5.bh.fastq, and SE.cf1.q5.bh.fastq

= the set of all reads after contamination filtering that were subsequently quality-filtered, then error-corrected

4.1.1.4 Insert size statistics

Where relevant below, mean (432), min (83) and deviation of insert sizes were obtained using Picard-Tools CollectInsertSizeMetrics (http://broadinstitute.github.io/picard) after mapping the paired-end reads to an early long read assembly with Bowtie2 (Langmead and Salzberg 2012).

4.1.2 Assembling the short reads

A total of 44 short read assemblies were reported on in this study: 40 prior to initial evaluations and contamination analysis and filtering, and 4 thereafter using the top performing assembler given the evaluations.

4.1.2.1 ABYSS

A total of 8 Abyss assemblies were made from Short Read Sets #1-4 (commands for each represented below), each with two different kmer sizes.

For all: K=k, where k was either 55 or 77

```
abyss-pe name=Sciara j=16 k=${k} in='R1.fastq  R2.fastq'

abyss-pe name=Sciara j=16 k==${k} lib='pe432' pe432='R1.q5.fastq
R2.q5.fastq' se='SE.q5.fastq'

abyss-pe name=Sciara j=16 k==${k} lib='pe432' pe432='R1.bh.fastq.gz
R2.bh.fastq.gz' se='SE.bh.fastq.gz'

abyss-pe name=Sciara j=16 k==${k} lib='pe432' pe432='R1.q5.bh.fastq.gz
R2.q5.bh.fastq.gz' se='SE.q5.bh.fastq.gz'
```

4.1.2.2 Megahit

Megahit was run on Short Read Sets #1-4, generating four assemblies. Megahit iterates over kmer sizes such that no specific kmer size need be provided. It was run on Short Read Sets #1-4 as follows.

```
megahit -1 R1.fastq -2 R2.fastq

megahit -1 R1.q5.fastq -2 R2.q5.fastq -r SE.q5.fastq

megahit -1 R1.bh.fastq -2 R2.bh.fastq -r SE.bh.fastq

megahit -1 R1.q5.bh.fastq -2 R2.q5.bh.fastq -r SE.q5.bh.fastq
```

4.1.2.3 Platanus

Platanus was first run on Short Read Sets #1-4, generating 4 assemblies. Platanus iterates over kmer sizes such that no specific kmer size need be provided. Evaluations showed that Platanus was a top performing short read assembler given our data, so we subsequently used Platanus with the contamination filtered read sets as well (Short Read Sets #5-8). For all Short Read Sets (#1-8) described in section 4.1.1.3, the commands were as represented below.

```
PREFIX=out
contig=${PREFIX}_contig.fa
bubble=${PREFIX}_contigBubble.fa
kmerfreq=${PREFIX}_kmerFrq.tsv
scaffold=${PREFIX}_scaffold.fa
scafbubble=${PREFIX}_scaffoldBubble.fa
scafcomp=${PREFIX}_scaffoldComponent.tsv
T=16
M=45
PE1 = R1 fastq file for given read set
PE2 = R2 fastq file for given read set
SE = orphaned reads from given read set if applicable
```

```
platanus assemble -f $PE1 $PE2 [$SE] -o $PREFIX -t $T -m $M
platanus scaffold -c $contig -b $bubble -IP1 $PE1 $PE2 -n1 83 -a1 432 -
d1 21 -t $T -o $PREFIX
platanus gap_close -o $PREFIX -c $scaffold -IP1 $PE1 $PE2 -t $T
```

4.1.2.4 SGA

SGA has its own error-correction so only the raw reads and quality-filtered reads were used with it (i.e. Short Read Sets #1-2).

```
SGA -- unfiltered:
#SAMtools 0.1.19 and ABYSS 1.9.0 in PATH.
PE1=R1.fastq
PE2=R2.fastq
See SGA recipe below.

SGA -- filtered:
#SAMtools 0.1.19 and ABYSS 1.9.0 in PATH.
PE1=R1.q5.fastq
PE2=R2.q5.fastq
See SGA recipe below.
Note: SE.q5.fastq not used.

SGA recipe -- same for unfiltered and filtered reads:
# Largely drawn from example here:
https://github.com/jts/sga/blob/master/src/examples/sga-celegans.sh

# SGA has its own read correction, so did not use with BayesHammer
corrected reads

NAME=Sciara-male
OL=75
T=16
D=4000000
CK=51
COV_FILTER=2
MOL=55
R=10
MIN_PAIRS=5
MIN_LENGTH=200
SCAFFOLD_TOLERANCE=1
MAX_GAP_DIFF=0
CTGS=assemble.m$OL-contigs.fa
GRAPH=assemble.m$OL-graph.asqg.gz
SGA_ALIGN=~/software/sga/sga/src/bin/sga-align

sga preprocess --pe-mode 1 $PE1 $PE2 > $NAME.fastq

sga index -a ropebwt --no-reverse -t $T $NAME.fastq

sga preqc -t $T ${NAME}.fastq > $NAME.preqc

sga correct -k $CK --discard --learn -t $T -o ${NAME}.ec.k$CK.fastq
${NAME}.fastq

sga index -a ropebwt -t $T ${NAME}.ec.k$CK.fastq

sga filter -x $COV_FILTER -t $T --homopolymer-check --low-complexity-
check ${NAME}.ec.k$CK.fastq
```

```
sga fm-merge -m $MOL -t $T -o ${NAME}.merged.k$CK.fa
${NAME}.ec.k$CK.filter.pass.fa

sga index -d 1000000 -t $T ${NAME}.merged.k$CK.fa

sga rmdup -t $T ${NAME}.merged.k$CK.fa

sga overlap -m $MOL -t $T ${NAME}.merged.k$CK.rmdup.fa

sga assemble -m $OL -g $MAX_GAP_DIFF -r $R -o assemble.m$OL
${NAME.merged.k$CK.rmdup.asqg.gz

sga assemble -m $OL -g $MAX_GAP_DIFF -r $R -o assemble.m$OL
${NAME}.merged.k$CK.rmdup.asqg.gz

$SGA_ALIGN --name ${NAME}.pe $CTGS $PE1 $PE2

sga-bam2de.pl -n $MIN_PAIRS --prefix libPE ${NAME}.pe.bam

sga-astat.py -m $MIN_LENGTH ${NAME}.pe.refsort.bam > libPE.astat

sga scaffold -m $MIN_LENGTH --pe libPE.de -a libPE.astat -o
scaffolds.n$MIN_PAIRS.scaf $CTGS

sga scaffold2fasta -m $MIN_LENGTH -a $GRAPH -o scaffolds.n$MIN_PAIRS.fa
-d $SCAFFOLD_TOLERANCE --use-overlap --write-unplaced
scaffolds.n$MIN_PAIRS.scaf
```

4.1.2.5 SOAPdenovo2

A total of 8 SOAPdenovo2 assemblies were made from Short Read Sets #1-4 (commands for each represented below), each with two different kmer sizes (K=55 or K=77).

```
PE1=R1.fastq
PE2=R2.fastq
```

Or

```
PE1=R1.q5.fastq
PE2=R2.q5.fastq
SE=SE.q5.fastq
```

Or

```
PE1=R1.bh.fastq
PE2=R2.bh.fastq
SE=SE.bh.fastq
```

Or

```
PE1=R1.q5.bh.fastq
PE2=R2.q5.bh.fastq
SE=SE.q5.bh.fastq
```

<u>Config file for all</u>:

```
#For example config file: http://soap.genomics.org.cn/soapdenovo.html
#maximal read length
max_rd_len=100
```

```
 [LIB]
#average insert size
avg_ins=432
#if sequence needs to be reversed
reverse_seq=0
#in which part(s) the reads are used
asm_flags=3
#use only first 100 bps of each read
rd_len_cutoff=100
#in which order the reads are used while scaffolding
rank=1
# cutoff of pair number for a reliable connection (at least 3 for short
insert size)
pair_num_cutoff=3
#minimum aligned length to contigs for a reliable read location (at
least 32 for short insert size)
map_len=32

#a pair of fastq file, read 1 file should always be followed by read 2
file
q1=${PE1} ## dependent on which set of reads being used
q2=${PE2}  ## dependent on which set of reads being used

#fastq file for single reads
q=${SE} ## dependent on which set of reads being used (some do not
have)
```

Commands:

```
SOAPdenovo-127mer pregraph -s $config_file -K $K -R -o $graph_prefix -p
$P
SOAPdenovo-127mer contig -g $graph_prefix -R -p $P
SOAPdenovo-127mer map -s $config_file -g $graph_prefix -p $P
SOAPdenovo-127mer scaff -g $graph_prefix -F -p $P
```

4.1.2.6 SPAdes

The inputs to SPAdes were Short Read Sets #1-2. Since SPAdes used BayesHammer error-correction by default prior to assembly, this actually means it was run only on Short Read Sets #3-4. For a given read set, we error-corrected the input reads (SRS#1 or #2) in only one of the SPAdes runs and subsequently used the error-corrected reads (SRS #3 or #4) produced in that run with the "--only-assembler" option in subsequent runs. We tried SPAdes in three different ways for each set (see commands below), generating 6 SPAdes assemblies.

[square brackets only applicable when single-end read file used]

```
spades.py --pe1-1 $PE1 --pe1-2 $PE2 [--pe1-s $SE] -o default -t 64 --
only-assembler --cov-cutoff auto

spades.py -k 21,33,55,77 --pe1-1 $PE1 --pe1-2 $PE2 [--pe1-s $SE] -o
k2177 -t 64

spades.py -k 21,33,55,77 --pe1-1 $PE1 --pe1-2 $PE2 [--pe1-s $SE] -o
k2177auto -t 64 --cov-cutoff auto


## Allowing SPAdes to pick kmer size values automatically (it chose
K=21,33,55)
```

```
spades.py --careful --pe1-1 $PE1 --pe1-2 $PE2 [--pe1-s $SE] -o k213355
-t 64 [--only-assembler]

## Specifying kmer size values to go up to k=77 (K=21,33,55,77)
spades.py --careful -k 21,33,55,77 --pe1-1 $PE1 --pe1-2 $PE2 [--pe1-s
$SE] -o k21335577 -t 64 [--only-assembler]

## Specifying kmer size values to go up to k=77 (K=21,33,55,77) and
enabling the automatic coverage cutoff.
spades.py --careful -k 21,33,55,77 --pe1-1 $PE1 --pe1-2 $PE2 [--pe1-s
$SE] -o k21335577auto -t 64 --cov-cutoff auto [--only-assembler]
```

4.1.2.7 Velvet

Velvet was run on Read Sets #1-4 using two different kmer sizes each (K=55 or K-77), generating 8 assemblies.

[square brackets only applicable when single-end read file used]

```
K=55

Or

K=77

DIR=hash${K}

velveth ${DIR} ${K} -fmtAuto -shortPaired -separate $R1 $R2 [-short
$SE]

velvetg ${DIR} -clean yes -exp_cov auto -cov_cutoff auto
```

4.1.3 Evaluations of the Initial 40 Short Read Assemblies

To evaluate the short read assemblies, we used the following 7 metrics: (1) the NG50 length (length of contig such that 50% of the expected genome is contained on contigs that length and longer), (2) the percentage of mapped Illumina reads (a completeness measure), (3) the conditional probability of the reads given each assembly using LAP (Ghodsi et al. 2013), (4) the Bayesian probability that each assembly is correct given the reads with ALE (Clark et al. 2013), (5) the number of potential misassembled regions reported by FRC^{bam} (Vezzi et al. 2012), (6) the percentage of error-free bases from REAPR (Hunt et al. 2013), and (7) BUSCO (Simão et al. 2015). Metrics from LAP, ALE, FRC, and REAPR reflect the consistency of each assembly with the Illumina dataset and BUSCO estimates the completeness of each assembly in terms of gene content. Note that the percentage of mapped reads was a great predictor of how assemblies would be ranked by LAP and ALE.

LAP and ALE provide probability measures that a given set of reads came from each assembly. LAP requires finding all mappings for each read, which can be time-consuming and demanding of computational resources. However, the authors note that fairly small sample sizes of reads tend to correlate well with larger samples for eukaryotic genome assemblies, which can drastically differ from one assembler to the next (Ghodsi et al. 2013). Therefore, we first tried two independent samples of ~15,000 paired reads, followed by a sample of ~150,000, and finally ~1.5 million. All samples largely agreed with each other and with the other metrics, so we used the final sample size of 1.5 million.

FRC^{bam} flags potential errors (called features) throughout each assembly given a set of reads, with lower numbers being better than higher ones. We also checked to see if normalizing to the number of features per megabase changed the conclusions, but found that it was highly correlated with the total number of features (Spearman's rho = 0.88) and that the rank leaders remained the same. Therefore, we used only the number of features in calculating mean rankings.

REAPR outputs the percent of bases in the assembly that are error-free as well as a score for each base in the assembly, which can be used to calculate the mean base score. We found that the percent of error-free bases was correlated with the mean base score (Spearman = 0.62), so chose to use only the former when calculating mean rankings.

BUSCO reports the percentage of complete single-copy orthologs (BUSCOs) found in each assembly given a set of BUSCOs expected to be present in the genome. The higher the percentage of complete BUSCOs found, the more complete and correctly put together an assembly is likely to be. For short read assembly evaluations, we used BUSCO v1.22 and the 2,675 SCOs from arthropods.

4.1.3.1 Size statistics:
Size statics (such as NG50) of the resulting contigs/scaffolds were calculated with asm-stats.py from SciaraTools: https://github.com/JohnUrban/sciara-project-tools

4.1.3.2 Mapping the reads back to each assembly:
With: Bowtie 2 version 2.2.9 (Langmead and Salzberg 2012), samtools v1.3 (Li et al. 2009).

The raw reads (Short Read Set #1) were mapped back to each assembly, and subsequently used to identify the percent that mapped and in ALE and FRC^{bam}. Note: LAP and REAPR have their own mapping strategies.

```
FILE=ASM.name.fasta
BASE=ASM.name

bowtie2-build $FILE $BASE

bowtie2 -p $P -q --very-sensitive -N 1 -x $BASE -1 $R1 -2 $R2 2>
${BASE}.err | samtools sort -o reads.bam

samtools index reads.bam
```

4.1.3.3 Percent of Illumina reads that mapped:
This was taken from the output report of Bowtie2.

4.1.3.4 ALE
With: boost 1.55, ALE (version from Scott Clark 2010)

ALE was used with the raw reads (Short Read Set #1) – see "Mapping reads back to each assembly" above.

```
FILE=ASM.name.fasta
BASE=ASM.name

ALE reads.bam $FILE ${BASE}.ALE.txt >> ${BASE}.err
```

Note: ABySS outputs contigs with non-ACGT IUPAC letters, which was breaking ALE. Therefore, each non-ACGT IUPAC letter was converted to one of the ACGT bases it represented at random with respect to the frequency ACGT occurs in the assembly. This was done using IUPAC-to-ACGT.py from: https://github.com/JohnUrban/sciara-project-tools

```
IUPAC-to-ACGT.py --f abyss.asm.fasta > abyss.asm-acgt.fasta
```

4.1.3.5 FRC^{bam}
FRC^{bam} was used with the raw reads (Short Read Set #1) – see "Mapping reads back to each assembly" above.

```
FILE=ASM.name.fasta
BASE=ASM.name

FRC --pe-sam reads.bam --pe-max-insert 800 --genome-size 292000000 --
output ${BASE}.frc
```

For short read assemblies, normalizing the number of features reported by FRC by the assembly size did not have much of an effect on rankings. Note that an expected genome size of 292Mb was used here rather than 280 Mb based on an earlier estimate.

4.1.3.6 LAP
We used the LAP scores generated for each assembly given the same set of input reads to rank them relative to each other. The raw read set (Short Read Set #1) was down sampled with sampleFastq.py (from: https://github.com/JohnUrban/sciara-project-tools). We tried lap using different proportions (p) of the full dataset, where p was specified during down-sampling as either 0.0001, 0.001, and 0.01 for approximately 15k, 150k, and 1.5 M sampled paired reads

respectively. Note: sampleFastq.py returns "downsampled.1.fastq" and "downsampled.2.fastq". Ultimately, we used the results for 1.5 M reads.

```
R1=R1.fastq
R2=R2.fastq
FILE=ASM.name.fasta
BASE=ASM.name
P=16
PROPORTION=p

sampleFastq.py -1 R1.fastq -2 R2.fastq -wo -p $PROPORTION -o
downsampled

bowtie2-build $FILE $BASE

calc_prob.py -p $P -a $FILE  -q -1 $R1 -2 $R2 -X 800 -I 0 -o fr -m 432
-t 75 -b $BASE > ${BASE}.prob

sum_prob.py -i ${BASE}.prob > ${BASE}.lapscore
```

4.1.3.7 REAPR

REAPR was used with the raw reads (Short Read Set #1). For short read assemblies, we looked at the reported percent error-free bases. We also calculated the mean of the scores over every base in the genome, which gave similar ranking results.

```
FILE=ASM.name.fasta
BASE=ASM.name

reapr facheck ${FILE} ${BASE}_renamed
reapr perfectmap ${BASE}_renamed.fa $R1 $R2 432 perfect
reapr smaltmap ${BASE}_renamed.fa $R1 $R2 mapped.bam  -n $P
reapr pipeline ${BASE}_renamed.fa mapped.bam output_directory perfect
```

4.1.3.8 BUSCO v1:

Short read assemblies were evaluated based on the number of Arthropoda BUSCOs detected in each.

```
LINEAGE=/Path/to/lineages/arthropoda
MODE=genome
BUSCO_v1.22.py -in $ASM -o $OUT -l $LINEAGE -m $MODE --cpu $CPU
```

4.1.4 Contamination analyses for the selected Platanus short read assembly

It has been shown that filtering out the contaminating reads can improve an assembly (Kumar et al. 2013). Therefore, to remove reads from contaminating species, we adopted a procedure similar to that used for the Tardigrade genome (Koutsovoulos et al. 2016) with the help of BlobTools (Laetsch and Blaxter 2017). We focused on the Platanus assembly produced from quality filtered, error-corrected reads. Instead of using the final gap-closed scaffolds, we used the Platanus contigs (largest contig ~77 kb) to allow each contig to be characterized separately and avoid discarding data due to misjoins.

Ideally one could use coverage and/or GC content information associated with annotated bacterial contigs to also eliminate the likely bacterial contigs that were not annotated. Unfortunately, the annotated bacterial contigs were of equivalent coverage compared with those annotated as arthropod (see Supplemental Figure S3). Moreover, the GC content of the bacterial and eukaryotic contig clusters overlapped enough to limit its usefulness. Therefore, we used an alternative set of reads from pre-amplification stage salivary glands (Urban et al. 2016), reasoning that since this dataset was from a different tissue, from a different stage, and prepared by a different person, the contaminating contigs would have much lower coverage. Over 95% of these reads mapped to the assembly, suggesting most or all of the somatic *Sciara* genome is represented in the Platanus contigs. As expected, the BlobPlot demonstrates that the coverage of the bacterial cluster is much lower in this dataset (see Supplemental Figure S3). We therefore marked for removal non-Arthropod-labeled contigs (and associated reads) from the assembly that were either labeled as super-kingdom "Bacteria" or had coverage < 0.1. To filter out contaminating reads for re-assembly, we removed all paired-end reads where both mates mapped to contigs marked for removal, and retained pairs when at least one mate mapped inside a retained contig or when both mates did not map to the assembly. The retained paired-end reads were used for assembly with Platanus in four ways: (i) no quality filtering nor error-correction, (ii) no quality filtering but error-correction with BayesHammer, (iii) quality filtering but no error-correction, and (iv) both quality filtering and error correction.

Overall, the contiguity statistics of the re-assembled Platanus contigs did not improve over simply eliminating the contaminating contigs from the original assembly, with the longest contig being ~28 kb in both scenarios. The scaffold contiguity statistics were slightly smaller than the original Platanus assemblies due to the absence of the very large bacterial scaffolds. Evaluation scores were similar among the four Platanus re-assemblies from different sets of filtered reads. REAPR's percent error-free bases metric improved drastically from ~56% error-free bases in the original assemblies to ~80% error-free bases in the assemblies produced from contamination-filtered reads. The assembly from quality-filtered, error-corrected reads had the highest percentage of error-free bases. We attempted a second round of contamination-filtering on this assembly, but found few additional benefits. There were only 15 contigs labeled as Bacteria, all *Rickettsia* at the genus level, summing in length to only 2,277 bp.

4.1.4.1 Obtaining taxonomy IDs from BLAST hits for each short read Platanus contig:

The Platanus fasta file containing contigs (not scaffolds) from the assembly of quality filtered, error-corrected reads (Short Read Set #4) was subdivided into 1000 different fasta files and submitted to SLURM as a batch script array that ran the following on each:

```
NT=/Path/to/entire/nucleotide/database
blastn -task megablast -query $Q -db $NT \
 -outfmt '6 qseqid staxids bitscore std sscinames sskingdoms stitle' \
 -culling_limit 5 \
 -num_threads $P \
 -evalue 1e-25 \
 -out ${BLASTDIR}/${PRE}.${SLURM_ARRAY_TASK_ID.blastout}
```

After ensuring all jobs completed successfully (and re-running if not), final blast results were all combined into a single file:

```
platanus.q5.bh.blast.results
```

The NCBI Taxonomy database containing nodes.dmp and names.dmp was downloaded into a subdirectory referred to as "./tax/" in commands below. This was used with BlobTools and custom taxonomy scripts as described below to classify contigs given the taxonomy IDs associated with each.

4.1.4.2 Using BlobTools and custom scripts to label each contig with a taxonomy:

Blob plots using platanus kmer coverage from the assemblies:

```
B=platanus.q5.bh.blast.results
BASE=platanus.q5.bh.contig
REF=${BASE}.fasta
DB=${BASE}.BlobDB.json

# Create blob database using kmer coverage from the assemblies
blobtools create -i ${REF} -y platanus --nodes tax/nodes.dmp --names
tax/names.dmp -o ${BASE} -t $B

# Plot
blobtools blobplot -i ${DB} [--format pdf]
blobtools blobplot -i ${DB} -r superkingdom [--format pdf]
blobtools blobplot -i ${DB} -r order -p 10 [--format pdf]
blobtools blobplot -i ${DB} -r family -p 12 [--format pdf]

# Make Table with Taxon Summaries for each contig
blobtools view -o  ${BASE}.table -i $DB -r all
```

Blob plots using read coverage:

The GC content for bacterial contigs was similar to the eukaryotic contigs. Moreover, the Bacterial and Eukaryotic contigs had similar coverage levels given the male embryo dataset used in the assemblies. Therefore, we tried a separate dataset generated from dissected out pre-amplification stage larval salivary glands (Urban et al. 2016). Reads were aligned with Bowtie 2 (Langmead and Salzberg 2012).

```
PFX=pre-amp-sal-gland-reads
READS=${PFX}.fastq.gz
BASE=platanus.q5.bh.contig
REF=${BASE}.fasta
B=platanus.q5.bh.blast.results
DB=${PFX}.${BASE}.BlobDB.json

# Map reads
bowtie2 -p $P -q --very-sensitive -N 1 -x ${BASE} -U ${READS} 2>
${PFX}.${BASE}.bt2.err | samtools sort --threads $P -T ${PFX}.tmp -o
${PFX}.bam

# Get contig coverage levels
blobtools bam2cov -i ${REF} -b ${PFX}.bam -o bam2cov --mq 0
grep -v ^# bam2cov.${PFX}.bam.cov | cut -f 1,3 > ${PFX}.cov

# Create Blob database
blobtools create -i ${REF} --cov ${PFX}.cov --nodes tax/nodes.dmp --
names tax/names.dmp -o ${PFX}.${BASE} -t $B

# Plot
blobtools blobplot -i ${DB} [--format pdf]
```

```
blobtools blobplot -i ${DB} -r superkingdom [--format pdf]
blobtools blobplot -i ${DB} -r order -p 10 [--format pdf]
blobtools blobplot -i ${DB} -r family -p 12 [--format pdf]
```

We also used custom scripts (https://github.com/JohnUrban/sciara-project-tools/tree/master/taxon) that segregate sequence names based on the closest taxonomy level (species, genus, family, order, class, phylum, kingdom, superkingdom, othersuperkingdoms) to our target species (Bradysia coprophila, Bradysia, Sciaridae, Diptera, Insecta, Arthropoda, Metazoa, Eukaryota, Bacteria/Archaea) that is found in the BLAST hits for each sequence. We required only 1 BLAST hit for a given level for it to be considered the closest level (i.e. if 1 BLAST hit says "Bradysia coprophila", then it will be assigned to the species level as the closest level even if other levels have more hits).

```
NODES=tax/nodes.dmp
NAMES=tax/names.dmp
BFILE=platanus.blobfilt1.q5.bh.contig.blast.results
TAX=$BFILE.taxonomy.out
PRE=platanus.blobfilt1.q5.bh.contig.taxsum
ALL=q5-bh/platanus.blobfilt1.q5.bh_contig.names
taxonomyFromTaxID.py -i $BFILE -nc 1 -tc 2 -no $NODES -na $NAMES > $TAX
taxonomy-summarizer.py -i $TAX -o $PRE -a $ALL
```

This reproduced the BlobTools approach, returning the same contigs labeled as Bacterial.

4.1.4.3 Obtaining contamination-filtered reads for re-assembly:
The set of male embryo paired-end reads (Short Read Set #1) was filtered as follows, making use of BEDtools (Quinlan and Hall 2010), SAMtools (Li et al. 2009), and linux commands.

```
# Make Table with Taxon Summaries for each contig
blobtools view -o  ${PFX}.${BASE}.table -i $DB -r all

## Grab contigs that are not labeled Bacterial at Super Kingdom level
that are above a coverage level of 0.1
grep -v ^#  ${PFX}.${BASE}.table | \
      awk '$6 != "Bacteria" && $5 >= 0.1' | \
      awk 'OFS="\t" {print $1,0,$2}' | \
      sortBed -i - > contigs.to.keep.bed

## Add to those, contigs below the coverage cutoff of 0.1 that are
explicitly labeled as Arthropoda sequences
grep -v ^#  ${PFX}.${BASE}.table | \
      awk '$5 < 0.1' | \
      grep Arthropoda | \
      awk 'OFS="\t" {print $1,0,$2}' | \
      sortBed -i - >> contigs.to.keep.bed

# Get read pairs from male embryo dataset (SRS#1 above) where at least
one read in the pair maps to one of the contigs-to-keep
BAM=${PFX}.bam
BED=contigs.to.keep.bed
bedtools pairtobed -abam ${BAM} -b ${BED} | \
      samtools sort -n | \
      samtools fastq -1 ${PFX}.m.R1.fq -2 ${PFX}.m.R2.fq -n -

#Also identify reads where neither maps to the assembly that might be
rescued in a subsequent assembly
samtools view -bS -f12  ${BAM} | \
```

```
        samtools sort -n | \
        samtools fastq -1 ${PFX}.u_u.R1.fastq -2 ${PFX}.u_u.R2.fastq -n –

    # Combine ${PFX}.m.R1.fq and ${PFX}.u_u.R1.fastq
    # Combine ${PFX}.m.R2.fq and ${PFX}.u_u.R2.fastq
```

4.1.4.4 Platanus contamination-filtered re-assemblies:

Four new Platanus assemblies were made using the set of contamination-filtered reads as well as quality-filtered and/or error-corrected derivatives of it (Short Read Sets #5 - #8).

(i) Contamination-filtered reads (Short Read Set #5) were assembled as described in Platanus sub-section of Section 2.1.2.

(ii) Contamination-filtered reads that were quality-filtered using the Trimmomatic command below (Short Read Set #6) were subsequently assembled as described in Platanus sub-section of Section 2.1.2.

```
java -jar $TRIMMOMATIC_BASE/Trimmomatic-0.32/trimmomatic-0.32.jar PE -
trimlog trim.log $IN $OUT ILLUMINACLIP:${PRIMERS}:2:30:10 LEADING:5
TRAILING:5 SLIDINGWINDOW:4:5 MINLEN:100
```

(ii) Contamination-filtered reads that were then error-corrected with BayesHammer using the SPAdes command below (Short Read Set #7) were subsequently assembled as described in Platanus sub-section of Section 2.1.2.

```
spades.py --pe1-1 $PE1 --pe1-2 $PE2 -o assembly -t 16 --only-error-
correction
```

(iv) Contamination-filtered reads that were already quality-filtered (SRS#6) were then subsequently error-corrected with the following SPAdes command to produce Short Read Set #8, which was subsequently assembled as described in Platanus sub-section of Section 2.1.2.

```
spades.py --pe1-1 $PE1 --pe1-2 $PE2 --pe1-s $SE -o assembly -t 16 --
only-error-correction
```

4.1.4.5 Evaluations of contamination-filtered re-assemblies:

The four Platanus assemblies (gap-closed scaffolds) from contamination-filtered datasets were evaluated in the same way as the evaluations of the first 40 assemblies were as described in **Section 4.1.3** (Size statistics, Percent reads mapped, ALE, FRC^{bam}, ALE, REAPR, BUSCOv1) except that the set of contamination filtered reads (Short Read Set #5) was used for these evaluations. This means some of the scores based on reads are not directly comparable to the first 40 assemblies (and do not appear in the ranking matrices, etc). Nevertheless, for our datasets, this re-assembly approach yielded assemblies that were extremely similar the original set of Platanus assemblies after simply removing bacterial labeled contigs/scaffolds. It did not improve upon the size statistics.

Assembly scores were similar among the four assemblies in **Section 4.1.4.4** ("i", "ii", "iii","iv"). Assembly "ii" (quality filtered) received the best LAP and ALE scores. Assembly "i" had the fewest

number of features detected by FRC^{bam}. Assembly “iv” had the highest percentage of error-free bases as determined by REAPR. Since “iv” had the highest REAPR score and since the combination of quality-filtering and error-correction also won out in the previous set of Platanus evaluations before contamination filtering, we chose this assembly as the final/only Platanus assembly to take into a second round of contamination filtering. The contigs and scaffolds were separately BLAST for taxonomy information as above. BlobTools databases and plots were constructed as above for kmer coverage as well as coverage from pre-amplification salivary gland reads. However, there was no additional contamination filtering needed.

4.2 Long Read Assemblies using PacBio and Oxford Nanopore Data

Summary:
We generated five hybrid assemblies (that combined short and long read technologies) starting with our final Illumina-based Platanus contigs using DBG2OLC (Ye et al. 2016) as well as 45 long-read-only assemblies with several long read assemblers including Canu (Koren et al. 2017), Falcon (Chin et al. 2016), Miniasm (Li 2016), ABruijn (Lin et al. 2016), and SMARTdenovo (https://github.com/ruanjue/smartdenovo). Assembling the *Sciara* genome with HINGE (Kamath et al. 2016) was also attempted, but it required more computational resources than we had available. For each assembler we tried different inputs (see supplemental figure S4 as well): (i) quality-filtered PacBio subreads , (ii) all PacBio subreads, (ii) quality-filtered PacBio subreads with all ONT 2D reads, (iii) all PacBio subreads with one ONT read per molecule, and (iv) only for Miniasm, all PacBio subreads and all ONT reads for each molecule ("PBall+ONTall") where each molecule can have up to three reads (template, complement, 2D). In total, there were 50 assemblies chosen to polish and evaluate after selecting several of the best candidates from each assembler for each dataset using size statistics.

Canu and Falcon follow similar paradigms where long reads are first error-corrected by mapping them against each other to construct higher quality consensus reads. They both then use the error-corrected reads to find overlaps and ultimately generate contigs. Miniasm, ABruijn, and SMARTdenovo skip an initial error-correction step and find overlaps in the raw long reads instead. ABruijn, Canu, Falcon, and SMARTdenovo have consensus steps built into the end of their assembly pipelines. RaCon, a rapid consensus caller (Vaser et al. 2016), was used with Miniasm assemblies to generate a consensus and the consensus approach using pbdagcon (https://github.com/PacificBiosciences/pbdagcon) (Chin et al. 2013) was used for Platanus+DBG2OLC assemblies as recently described (Chakraborty et al. 2016). All evaluation metrics for Miniasm, which skips both pre-assembly error-correction and post-assembly consensus steps, improved with RaCon as expected. For example, BUSCO found 0 BUSCOs in Miniasm assemblies before RaCon and 29-32% afterward. Similarly, only 29-31% of Illumina reads mapped and 0% of bases were judged error-free by REAPR in Miniasm assemblies prior to RaCon whereas 94-95% of Illumina reads mapped and 70-71% of bases were judged error-free after RaCon.

The number of assemblies from each assembler was mostly determined by how easy, fast, or resource-efficient a given assember was to run different combinations of parameters or reads with. The number for each was:

Abruijn = 3
Canu = 17
DBG2OLC (hybrid) = 5
Falcon = 12
Miniasm = 8
SMARTdenovo = 5

Total = 50

4.2.1 Long read inputs to assemblers

We tried different levels of quality filtering of the long reads. For example, we either used all or a filtered subset of the PacBio reads, and we either blended in a read from every molecule sequenced on the MinION, which included 1D reads when 2D reads were not present, or blended in only 2D reads. Details are below.

4.2.1.1 Long read set naming

Long Read Set #1 (PBall)
= All PacBio subreads

Long Read Set #2 (PBfilt)
= PacBio reads filtered to be have minReadScore > 0.75

Long Read Set #3 (PBall+ONTmol)
= All PacBio subreads (LRS#1)
+ Oxford Nanopore "molecule" reads (one read per molecule)

Long Read Set #4 (PBall+ONT2d)
= All PacBio subreads (LRS#1)
+ Oxford Nanopore 2D reads

Long Read Set #5 (PBfilt+ONT2d)
= PacBio reads filtered to be > 0.75 (LRS#2)
+ Oxford Nanopore 2D reads

Long Read Set #6 (PBall+ONTall)
= All PacBio subreads (LRS#1)
+ All Oxford Nanopore reads (one, two, or three per molecule)
Note: Only used by Miniasm and RaCon

Long Read Set #7 (PBfilt+ONTall)
= PacBio reads filtered to be > 0.75 (LRS#2)
+ All Oxford Nanopore reads (one, two, or three per molecule)
Note: Only used by Miniasm

Long Read Set #8 (PBfilt+ONTmol):
= PacBio reads filtered to be > 0.75 (LRS#2)
+ Oxford Nanopore "molecule" reads (one read per molecule)
Note: Only used by Miniasm

4.2.1.2 Obtaining fasta/fastq files from PacBio .bax.h5 files using bash5tools

Bash5tools.py from pbh5tools (https://github.com/PacificBiosciences/pbh5tools) was used. Either all PacBio sub-reads were used, or quality filtered subreads with a cutoff of 0.75.

```
# Long Read Set #1 (PacBioAll)
bash5tools.py --readType subreads \
      --outType fasta \
      --outFilePrefix $PREFIX file.bax.h5


# Long Read Set #2 (PacBioFilt)
bash5tools.py --readType subreads --outType fasta \
      --minReadScore 0.75 \
      --outFilePrefix $PREFIX file.bax.h5
```

4.2.1.3 Obtaining fasta/fastq files from MinION fast5 files using fast5tools

In summary, Fast5Tools (https://github.com/JohnUrban/fast5tools) was used to extract and analyze reads from the many different MinION libraries we generated. Examples of how one molecule per read or only 2D reads were extracted are show here and described more below:

```
# Extract all reads (up to three per molecule) from fast5s
# This gives ONTall as used with Long Read Set #6

fast5tofastx.py -r all -o fasta --tarlite minIONreads.tar.gz >
ONTall.fa


# Get one read per molecule(Urban et al 2015, and described below)
# Can derive from the fast5 files as above or from ONTall.fa
# This gives ONTmol as used in Long Read Set #3

fast5tofastx.py -r molecule -o fasta --tarlite minIONreads.tar.gz >
ONTmol.fa
# OR:
filterFast5DerivedFastx.py -r molecule ONTall.fa > ONTmol.fa


# Extract only the 2D reads from that previous dataset (or from fast5s)
# This produces ONT2d as used in Long Read Sets #4 and #5

fast5tofastx.py -r 2d -o fasta --tarlite minIONreads.tar.gz > ONTmol.fa
# OR:
filterFast5DerivedFastx.py -r 2d ONTmol.fa > ONT2d.fa
```

4.2.1.4 A more detailed description of Fast5Tools and MinION read analyses:

Fast5 files were base-called with Oxford Nanopore's Metrichor cloud base-caller at the time the data was produced. See information on libraries elsewhere for dates and MAP kit versions. Metrichor (ONT base-caller) returned updated fast5 files into two folders: "pass" and "fail". "Pass" contained only fast5 files where 2D base-calling was successful and the mean quality of the 2D read was >9. Everything else (including other fast5s containing 2D reads with Q<9, fast5s with only 1D base-calling, and fast5s that failed base-calling) was put into the "fail" folder by Metrichor. We made use of data from both folders where possible. For libraries 01, 02, 03, 04, 05, 06, 07,

08, and 09, our toolset called poreminion (https://github.com/JohnUrban/poreminion) as used previously (Urban et al. 2015) to categorize the fast5s in the “fail” folder that were not base-called due to various reasons: no template, too few events, too many events, or contained an error where blocks of events are artificially repeated. The latter error was present for a minority of fast5 files in the early days of the MinION Access Program and was fixed by ONT before sequencing of Library09. Library09 and all subsequent libraries did not have any of these errors as expected.

```
#To categorize and rescue reads from the fail folder
poreminion uncalled -m -o fail-filter fail/

# To remove reads from early libraries that contained artificial
repeats of events
poreminion timetest -m -o fail-filter fail/
```

For all libraries, the pass and fail folders were individually archived and compressed with:

```
tar -xzf pass.tar.gz pass/
tar -xzf fail.tar.gz fail/
```

As this data was collected over the course of many months (2014-2016) and ONT was rapidly developing and changing the innards of the HDF5 fast5 files, we were presented with the need for a tool that can handle any version of a fast5 file given to it. At this point, we created another toolset called Fast5Tools (https://github.com/JohnUrban/fast5tools) that is able to deal with all of the different fast5 versions used in this study. Fast5Tools maintains all or most of the functionality of poreminion, although pre-filtering is no longer necessary.

For all MinION libraries, Fast5Tools was used to extract fasta and fastq information from the tar archived pass.tar.gz and fail.tar.gz files. Using the “--tarlite” option, Fast5Tools is able to work on files directly from the gzipped tar archive without extracting it all at once. This prevents issues of disk space that may arise from fully extracting the contents of the giant gzipped tar files before performing the needed operations. First to extract either all fasta or all fastq reads from every fast5 file, fast5tofastx.py was used:

```
fast5tofastx.py -r all -o fasta --tarlite pass.tar.gz > pass.all.fa

fast5tofastx.py -r all -o fasta --tarlite fail.tar.gz > fail.all.fa
```

The fast5 files can be filtered for length and quality information in the previous step (`fast5tofastx.py`). However, it can be done in subsequent steps as well as the headers of the resulting fasta/fastq returned from “`fast5tofastx.py`” contain all the information necessary to filter for reads using length, mean quality score, and other information using “`filterFast5DerivedFastx.py`”. Therefore, when the “`-r all`” option is used as above, filtering can be done on the fasta/fastq files exactly as from fast5 files. When one does not need access to the signal level of the data, this can be used to keep the smaller fasta/fastq file while storing the larger tarchives in longer term storage if necessary. The basic header structure for each fasta entry looks like this:

```
>readtype|len:LEN|Q:Q|channel:CHANNEL|Read:READNUMBER|asic:ASIC_ID|run:RUN_ID
|device:DEVICE_ID|model:BASECALL_MODEL
```

An example of the 3 read types (template, complement, and 2d) from a passing molecule:

```
>template|len:2781|Q:4.45259884791|channel:508|Read:80|asic:3372918206|
run:609a1b4408f2200edefabc0d97b202bcaccf94db|device:MN15216|model:r7.3_
e6_70bps_6mer
```

```
>complement|len:2783|Q:4.43810484237|channel:508|Read:80|asic:337291820
6|run:609a1b4408f2200edefabc0d97b202bcaccf94db|device:MN15216|model:r7.
3_e6_70bps_6mer

>2d|len:3248|Q:10.8914446375|channel:508|Read:80|asic:3372918206|run:60
9a1b4408f2200edefabc0d97b202bcaccf94db|device:MN15216|model:r7.3_e6_70b
ps_6mer
```

The fasta/fastq derived from the fast5 files with Fast5Tools can then be filtered using filterFast5DerivedFastx.py. We obtained all 2D reads from the fasta/fastq file by:

```
filterFast5DerivedFastx.py -r 2d libX.pass.all.fa > libX.pass.2d.fa

filterFast5DerivedFastx.py -r 2d libX.fail.all.fa > libX.fail.2d.fa
```

High quality pass 2D reads can be obtained by filtering just the pass.all.fa file for each library (using the above command) or if all reads are combined in a single fasta, doing:

```
filterFast5DerivedFastx.py -r 2d libX.all.fa --minq 9 > libX.pass.2d.fa
```

We selected one read per molecule using the "molecule" definition that we used previously (Urban et al. 2015). This takes the 2D read if one is present, the longer of template or complement if 2D is not present (but complement is), or the template read if it is the only one present.

```
filterFast5DerivedFastx.py -r molecule libX.pass.all.fa >
libX.pass.molecule.fa

filterFast5DerivedFastx.py -r molecule libX.fail.all.fa >
libX.fail.molecule.fa
```

To obtain statistics from each fast5 file on the reads present, Fast5Tools can work directly with the fast5 files (or tarchive of fast5 files) as well as from the fast5-derived fasta files (particularly when all reads are dumped out from each file as above). Both approaches give the same results concerning length and mean quality scores (more information about events can be obtained directly from the fast5 file):

```
fast5stats.py --standard --errfile libX.errfiles.txt --tarlite
libX.pass.tar.gz libX.fail.tar.gz > stats.txt

fast5DerivedFastxMoleculeStats.py libX.pass.all.fa libX.fail.all.fa >
stats.txt
```

Note that "--errfile errfiles.txt" is populated with information on what files had errors or were not base-called for various reasons (as stated above). There is no option for this when grabbing the statistics from fasta files as any erroneous or un-basecalled fast5 is ignored during extraction with "`fast5tofastx.py`". The output of both commands (`fast5stats.py`, `fast5DerivedFastxMoleculeStats.py`) is just one line per molecule (i.e. one line per original fast5 file) even if the information is coming from a fasta multiple reads per molecule. This is possible because `fast5DerivedFastxMoleculeStats.py` unites different reads from the same

molecule by using their shared molecule names derived from the channel, the read number, the asic ID, the run ID, and device ID). The output of both commands also share the first 11 columns:

1 = molecule name
2 = molecule length
3 = molecule mean q score
4 = has complement
5 = has 2d
6 = 2d seq len
7 = template seq len
8 = complement seq len
9 = 2d mean q score
10 = template mean q score
11 = complement mean q score

The first 11 columns of the output table was summarized with fast5standardSummary.py:

```
fast5standardSummary.py -V -f libX.stats.txt
```

At the moment, this outputs 264 pieces of information, including molecule length statistics (minimum, maximum, mean, median, N25, N50, N75, expected length) on molecules as well as the various read types. One can simply combine all stats.txt tables from each library to compute the summary on all molecules from all libraries.

4.2.1.5 MarginStats for percent identity of MinION Reads

The BAM file containing the alignments of all ONT reads mapped to the highest ranked PacBio-only assembly was filtered to remove unmapped reads using SAMtools. Then the resulting BAM files was partitioned into 400 smaller files using splitSAM.py from https://github.com/JohnUrban/sciara-project-tools. This enabled us to compute percent identities in parallel in the next step. MarginStats (Jain et al. 2015) was then used to calculate the end-to-end percent identities of the reads in each file. Optimized re-alignment was not performed, which can increase the percent identity reported. For this reason and since the assembly may have some errors itself, the percent identities reported here are conservative estimates. Since reads were extracted using our set of tools for working with fast5 files from ONT called fast5tools (https://github.com/JohnUrban/fast5tools), read names contained mean quality scores, which could then be paired with the percent identities from MarginStats.:

```
samtools view -bh -F4
canu10.minrl500.dip3x.pball.quiverfinal1.pilon2x/ont2d.bam >
reads/ont.bam
splitSAM.py --bam reads/ont.bam --nfiles 100 --nreads 1382636
```

On each BAM:

```
marginStats --noStats --printValuePerReadAlignment --identity $BAM $fq
$ref > wd/${pre}.txt
```

On each MarginStats output text file:

```
awk `{sub(/\ /,"\n"); gsub(/\t/,"\n"); print\' wd/${PRE}.txt | grep -v
\^{ValuesIdentity | paste - <(grep -v \^{@ <(samtools view
splitfiles/${PRE.bam)) | awk `OFS="\t" {print $2,$1\' >
pairs/${PRE}.txt
```

4.2.2 Hybrid-assembling long reads with short reads

4.2.2.1 DBG2OLC with Platanus short read contigs and PBDAGCON

We used the contigs from our “final” platanus assembly, which were assembled with a set of reads that went through one round of contamination-filtering, followed by quality filtering with Trimmomatic, and error-correction with BayesHammer from SPAdes.

Five hybrid assemblies were generated using Long Read Sets #1-5 (in place of longreads.fasta in commands below).

```
./DBG2OLC k 17 KmerCovTh 2 MinOverlap 20 AdaptiveTh 0.002 LD1 0 MinLen
200 Contigs platanus.contigs.fa RemoveChimera 1 f longreads.fasta

cat platanus.contigs.fa longreads.fasta > ctg_pb.fasta

mkdir consensus_dir

split_and_run_pbdagcon.path.sh backbone_raw.fasta
DBG2OLC_Consensus_info.txt ctg_pb.fasta consensus_dir >
consensus_log.txt
```

4.2.3 Assembling the long reads alone

4.2.3.1 ABRUIJN
Three ABruijn assemblies were generated using Long Read Sets #1, #3, and #5.

```
abruijn.py longreads.fasta Sciara $COV -t 32 --iterations 1
```

PBall (Long Read Set #1) : COV=42
PBall+ONTmol (Long Read Set #3) : COV=54
PBfilt+Ont2d (Long Read Set #5) : COV=47

At the time of this work, ABruijn runs required allocating 512 GB RAM and 32 CPUs, and 7-8 days of running time. Thus, it was impractical to try exhaustive combinations of reads, filtering, etc.

4.2.3.2 Canu
Canu is very sophisticated in automatically requesting appropriate resources and distributing/parallelizing its jobs across as many CPUS/nodes available on our compute cluster. It therefore finishes within a day or 2, and it was possible to test many different combinations of reads, filtering, parameters, etc. Indeed, we have also tested many earlier versions of Canu (not reported here). We report on 17 Canu assemblies in this study, generated using Long Read Sets #1-5. Note that an expected genome size of 292Mb was used here rather than 280 Mb based on an earlier estimate. That will have no effect on assemblies that used all of the data (when setting an extremely high coverage), and little effect on the amount of data used otherwise.

Below, variables that show up in the Canu commands are defined. Not all variables are used in all commands.

```
PBall=all_subreads.fasta  # LRS#1/PBall
PBfilt=subreads.gt0.75.fasta  # LRS#2/PBfilt
ONTmol=ontmolecule.fasta #Combine w/ PBall for LRS#3
ONT2d=ont2d.fasta #Combine w/ PBall for LRS#4 or w/ PBfilt for LRS#5
PBONT2D=allPBsubreads_ont2d.fasta # LRS#4/PBallONT2d
PBFILTONT2D=pbsubreads.gt0.75_ont2d.fasta # LRS#5/PBfiltONT2d
PBMOL=allPBsubreads_ontmolecule.fasta # LRS#3/PBallONTmol
COROUTCOV=500 # excessively high to include all reads
T=24:00:00
G=292m
```

Canu_1 = default.pball = Canu 1.3 using only PacBio reads (LRS#1/PBall):

```
NAME=g292-default-all
canu  -p $NAME -d $NAME  genomeSize=$G  \
      -pacbio-raw $PBall  "gridOptions=--time $T"  oeaMemory=8
```

Canu_2 = minrl500.pball = Canu 1.3 using only PacBio reads (LRS#1/PBall) and specifying a minimum read length of 500 bp:

```
NAME=g292-default-all-min500
canu -p $NAME -d $NAME genomeSize=$G \
      -pacbio-raw $PBall "gridOptions=--time $T" \
      minReadLength=500  oeaMemory=8
```

Canu_3 = corcov500.pball = Canu 1.3 using only PacBio reads (LRS#1/PBall) and fixing coverage parameters to use all reads (not just a subset of the longest sampled by Canu):

```
NAME=g292-default-all-corcov500
canu -p $NAME -d $NAME genomeSize=$G -pacbio-raw $PBall \
```

```
        "gridOptions=--time $T" oeaMemory=8 \
        corOutCoverage=$COROUTCOV
```

Canu_4 = corcov500minrl500.pball = Canu 1.3 using only PacBio reads (LRS#1/PBall), specifying a minimum read length of 500 bp, and fixing coverage parameters to use all reads (not just a subset of the longest sampled by Canu):

```
NAME=g292-default-all-corcov500-min500
canu -p $NAME -d $NAME genomeSize=$G \
        -pacbio-raw $PBall "gridOptions=--time $T"  \
        minReadLength=500 oeaMemory=8 corOutCoverage=$COROUTCOV
```

Canu_5 = corcov500.pbfilt = Canu 1.3 using only filtered PacBio reads (LRS#2/PBfilt) and fixing coverage parameters to use all reads (not just a subset of the longest sampled by Canu):

```
NAME=g292-default-q0.75-corcov500
canu -p $NAME -d $NAME genomeSize=$G
        -pacbio-raw $PBfilt "gridOptions=--time $T" \
        oeaMemory=8 corOutCoverage=$COROUTCOV
```

Canu_6 = corcov500minrl500.pbfilt = Canu 1.3 using only filtered PacBio reads (LRS#2/PBfilt), specifying a minimum read length of 500 bp, and fixing coverage parameters to use all reads (not just a subset of the longest sampled by Canu):

```
NAME=g292-default-q0.75-corcov500-min500
canu -p $NAME -d $NAME genomeSize=$G \
        -pacbio-raw $PBfilt "gridOptions=--time $T"  \
        minReadLength=500 oeaMemory=8 corOutCoverage=$COROUTCOV
```

Canu_7 = corcov500minrl500.pball.ontmol = Canu 1.3 using all PacBio reads and one read per molecule from ONT (LRS#3/PBall+ONTmol), specifying a minimum read length of 500 bp, and fixing coverage parameters to use all reads (not just a subset of the longest sampled by Canu):

```
NAME=g292-default-pball-ontmol-corcov500-min500
canu -p $NAME -d $NAME genomeSize=$G -pacbio-raw $PB \
        -nanopore-raw $ONTmol  "gridOptions=--time $T" \
        minReadLength=500 oeaMemory=8 corOutCoverage=$COROUTCOV \
        corMemory=40
```

Canu_8 = corcov500minrl500.aspb-e025.pball.ontmol = Canu 1.3 using all PacBio reads and one read per molecule from ONT (LRS#3/PBall+ONTmol), specifying a minimum read length of 500 bp, fixing coverage parameters to use all reads (not just a subset of the longest sampled by Canu), and combining the PB and ONT data into a single file treated by Canu as PacBio data:

```
NAME=g292-default-pball-ontmol-corcov500-min500-aspb
canu -p $NAME -d $NAME genomeSize=$G errorRate=0.02 \
        -pacbio-raw $PBall_ONTmol "gridOptions=--time $T" \
        minReadLength=500 oeaMemory=8 \
        corOutCoverage=$COROUTCOV corMemory=40
```

Canu_9 = corcov500minrl500.pball.ont2d = Canu 1.3 using all PacBio reads and 2D nanopore reads (LRS#4/PBall+ONT2d), specifying a minimum read length of 500 bp, and fixing coverage parameters to use all reads (not just a subset of the longest sampled by Canu):

```
NAME=g292-default-pball-ont2d-corcov500-min500
```

```
canu -p $NAME -d $NAME genomeSize=$G -pacbio-raw $PB \
      -nanopore-raw $ONT2d "gridOptions=--time $T" \
      minReadLength=500 oeaMemory=8  corOutCoverage=$COROUTCOV \
      corMemory=30
```

Canu_10 = corcov500minrl500.aspb-e02.pball.ont2d = Canu 1.3 using all PacBio reads and 2D nanopore reads (LRS#4/PBall+ONT2d), specifying a minimum read length of 500 bp, fixing coverage parameters to use all reads (not just a subset of the longest sampled by Canu), and combining the PB and ONT data into a single file treated by Canu as PacBio data:

```
NAME=g292-default-pball-ont2d-corcov500-min500-aspb
canu -p $NAME -d $NAME genomeSize=$G errorRate=0.02 \
      -pacbio-raw $PBall_ONT2d "gridOptions=--time $T" \
      minReadLength=500 oeaMemory=8 \
      corOutCoverage=$COROUTCOV corMemory=30
```

Canu_11 = corcov500.pbfilt.ont2d = Canu 1.3 using filtered PacBio reads and 2D nanopore reads (LRS#5/PBfilt+ONT2d), specifying a minimum read length of 500 bp, fixing coverage parameters to use all reads (not just a subset of the longest sampled by Canu):

```
NAME=g292-default-pbfilt-ont2d-corcov500-min500
canu -p $NAME -d $NAME genomeSize=$G -pacbio-raw $PBFILT \
      -nanopore-raw $ONT2d  "gridOptions=--time $T" minReadLength=500 \
      oeaMemory=8 corOutCoverage=$COROUTCOV corMemory=30
```

Canu_12 = corcov500minrl500.aspb-e02.pbfilt.ont2d = Canu 1.3 using filtered PacBio reads and 2D nanopore reads (LRS#5/PBfilt+ONT2d), specifying a minimum read length of 500 bp, fixing coverage parameters to use all reads (not just a subset of the longest sampled by Canu), and combining the PB and ONT data into a single file treated by Canu as PacBio data:

```
NAME=g292-default-pbfilt-ont2d-corcov500-min500-aspb
canu -p $NAME -d $NAME genomeSize=$G errorRate=0.02 \
      -pacbio-raw $PBfilt_ONT2d "gridOptions=--time $T" \
      minReadLength=500 oeaMemory=8 \
      corOutCoverage=$COROUTCOV corMemory=30
```

Canu_13 - 17 were run in similar ways as above with earlier versions of Canu. We used optimization scripts for the bogart step for a diploid genome: https://github.com/JohnUrban/sciara-project-tools/blob/master/canu-utilities/optimization-loop-2.sh

Canu_13 = canu10.minrl500.dip3x.pball = Canu 1.0 using PBall and a minimum read length of 500 bp.

Canu_14 = canu10.minrl1000.dip3x.pbfilt = Canu 1.0 using PBfilt, a minimum read length of 1000 bp, and the diploid 3X optimization.

Canu_15 = canu11.corcov80minrl500.dip3x.pball.ontmol = Canu 1.1 with PBall+ONTmol specifying a high coverage cutoff to use all or most of the data from reads >500 bp. The diploid optimization was run and we selected the B=3X assembly.

Canu_16 = canu12.corcov80minrl500.dip3x.pball.ontmol = Canu 1.2 with PBall+ONTmol specifying a high coverage cutoff to use all or most of the data from reads >500 bp. The diploid optimization was run and we selected the B=3X assembly.

Canu_17 = canu12.minrl500.dip3x.pball.ontmol = same as Canu_16, but with the default coverage cutoff.

4.2.3.3 Falcon

Assemblies were generated using Long Read Sets #1-5. Similar to Canu, since Falcon was able to launch jobs on SLURM in an efficient way, we were able to test many different sets of parameters and combinations of reads. Twelve assemblies are reported in this study that could be referred to as:

With LRS#1/PBall:

Falcon_1: default.PBall
Falcon_2: seed25.PBall
Falcon_3: seed30.PBall

With LRS#2/PBfilt:

Falcon_4: default.PBfilt
Falcon_5: seed25.PBfilt
Falcon_6: seed30.PBfilt

With LRS#3/PBall+ONTmol

Falcon_7: seed25.PBall.ontmol
Falcon_8: seed25.relaxed.PBall.ONTmol

With LRS#4/PBall+ONT2d

Falcon_9: seed25.PBall.ONT2d
Falcon_10: seed25.relaxed.PBall.ONT2d

With LRS#5/PBfilt+ONT2d

Falcon_11: seed25.PBfilt.ONT2d
Falcon_12: seed25.relaxed.PBfilt.ONT2d

The headers for fast5-derived fasta files from the MinION data are not appropriate for use with falcon, whereas PacBio headers are. To use our MinION data with the Falcon assembler, we used Fast5Tools to generate Falcon-acceptable headers as such:

```
filterFast5DerivedFastx.py -o falcon file.fa > file.forfalcon.fa
```

All assemblies were initiated via:

```
fc_run.py fc_run.cfg logging.ini
```

The “logging.ini” file for all Falcon assemblies contained these lines:

```
[loggers]
keys=root,pypeflow,fc_run

[handlers]
keys=stream,file_pypeflow,file_fc_run,file_all
```

```
[formatters]
keys=form01,form02

[logger_root]
level=NOTSET
handlers=stream,file_all

[logger_pypeflow]
level=NOTSET
handlers=file_pypeflow
qualname=pypeflow
propagate=1

[logger_pwatcher]
level=NOTSET
handlers=file_pwatcher
qualname=pwatcher
propagate=1

[logger_fc_run]
level=NOTSET
handlers=file_fc_run
qualname=fc_run
propagate=1

[handler_stream]
class=StreamHandler
level=INFO
formatter=form02
args=(sys.stderr,)

[handler_file_pypeflow]
class=FileHandler
level=DEBUG
formatter=form01
args=('pypeflow.log',)

[handler_file_pwatcher]
class=FileHandler
level=DEBUG
formatter=form01
args=('pwatcher.log',)

[handler_file_fc_run]
class=FileHandler
level=DEBUG
formatter=form01
args=('fc_run.log',)

[handler_file_all]
class=FileHandler
level=DEBUG
formatter=form01
args=('fc.log',)

[formatter_form01]
format=%(asctime)s - %(name)s:%(lineno)d - %(levelname)s - %(message)s

[formatter_form02]
format=[%(levelname)s] %(message)s
```

Falcon Default Configuration:
The “default” configuration file (fc_run.cfg) for “default.PBall” and " default.Pfilt " assemblies (Falcon_1 and Falcon_4) contained the following lines:

```
[General]

use_tmpdir = false
job_type = slurm
jobqueue = production
#stop_all_jobs_on_failure = true

# list of files of the initial bas.h5 files
input_fofn = input.fofn
input_type = raw

# The length cutoff used for seed reads used for initial mapping
length_cutoff = -1
genome_size = 292000000

# The length cutoff used for seed reads usef for pre-assembly (>0, was
not able to do -1)
length_cutoff_pr = 500

#Pre-Assembly
sge_option_da = --cpus-per-task 8 --mem 30g --time 48:000:00 --
qos=ccmb-condo
sge_option_la = --cpus-per-task 2 --mem 30g --time 48:00:00 --qos=ccmb-
condo
pa_DBsplit_option = -a -x500 -s200

pa_HPCdaligner_option = -v -B128 -e0.70 -M24 -l1000 -s100
pa_concurrent_jobs = 1000

#consensus for error correction
sge_option_cns = --cpus-per-task 8 --mem 60g --time 48:00:00 --
qos=ccmb-condo
falcon_sense_option = --output_multi --min_idt 0.70 --min_cov 4 --
max_n_read 200 --n_core 8
cns_concurrent_jobs = 1000

# overlap detection for assembly
sge_option_pda = --cpus-per-task 8 --mem 30g --time 48:00:00 --
qos=ccmb-condo
sge_option_pla = --cpus-per-task 2 --mem 30g --time 48:00:00 --
qos=ccmb-condo
ovlp_concurrent_jobs = 1000
ovlp_DBsplit_option = -x500 -s200
ovlp_HPCdaligner_option = -v -B128 -e.96 -M16 -l500 -s100

# overlap filtering
overlap_filtering_setting = --max_diff 40 --max_cov 80 --min_cov 2 --
n_core 12

sge_option_fc = --cpus-per-task 16 --mem 30g --time 48:00:00 --
qos=ccmb-condo
```

Falcon Seed=25 Configuration:
The "seed25" configuration files (fc_run.cfg) for "seed25.PBall", "seed25.PBfilt", "seed25.PBall.ontmol", "seed25.PBall.ont2d", "seed25.PBfilt.ont2d" (falcon assemblies #2, 5, 7, 9, and 11) were similar to the "default" configuration with the following line added:

```
seed_coverage = 25
```

Falcon Seed=30 Configuration:
The "seed25" configuration files (fc_run.cfg) for "seed25.PBall", "seed25.PBfilt", "seed25.PBall.ontmol", "seed25.PBall.ont2d", "seed25.PBfilt.ont2d" (falcon assemblies #2, 5, 7, 9, and 11) were similar to the "default" configuration with the following line added:

```
seed_coverage = 30
```

Falcon Relaxed Seed=25 Configuration:
The "relaxed seed 25" configuration file (fc_run.cfg) for "seed25.relaxed.PBall.ONTmol", "seed25.relaxed.PBall.ONT2d", and "seed25.relaxed.PBfilt.ONT2d" (Falcon assemblies 8, 10, and 12) was almost the same as "seed25" configuration above, but with the following lines added/changed:

specified a length cut-off
```
length_cutoff = 7500
```

In section titled "consensus for error correction", changed --min_idt from 0.70 to 0.65:
```
falcon_sense_option = --output_multi --min_idt 0.65 --min_cov 4 --
max_n_read 200 --n_core 8
```

In section titled "overlap detection for assembly", changed -e from .96 to .70
```
ovlp_HPCdaligner_option = -v -B128 -e.70 -M16 -l500 -s100
```

4.2.3.4 Miniasm and RaCon
Miniasm required < 64 GB RAM, 8 threads, and < 3 hours to complete. It was therefore practical to try many combinations of reads, filtering, etc. Since Miniasm does not have its own consensus step, RaCon was used. RaCon required < 150 GB RAM, 8 threads, and < 4.5 hours to finish. In total, Miniasm+RaCon took < 7.5 hours and is by far the fastest combination of assembly and consensus steps we tested. We generated eight miniasm assemblies using different combinations of reads.

Miniasm was used with Long Read Sets #1-8

RaCon used LRS#1/PBall for assemblies that used only PacBio data.
RaCon used LRS#6/PBallONTall for assemblies that used both PacBio and ONT data.

All Miniasm assemblies and RaCon polishing followed these commands:

```
minimap -Sw5 -L100 -m0 -t8 longreads.fasta longreads.fasta | \
      gzip -1 > reads.paf.gz

miniasm -f longreads.fasta reads.paf.gz > reads.gfa

awk '/^S/{print ">"$2"\n"$3}' reads.gfa > asm.fasta

minimap asm.fasta longreads.fastq > overlaps-for-racon.paf
```

```
racon -t 8 reads.fastq overlaps-for-racon.paf asm.fasta asm.racon.fasta
```

4.2.3.5 SMARTdenovo

Five SMARTdenovo assemblies were generated using Long Read Sets #1-5.

```
smartdenovo.pl -c 1 longreads.fasta > wtasm.mak
make -f wtasm.mak
```

SMARTdenovo required <48 GB RAM, 8 threads, and <15 hours to complete. It was therefore practical to try more combinations of reads, filtering, etc.

4.2.4 Polishing the Long Read Assemblies

Quiver polishing (Chin et al. 2013) was performed iteratively to take advantage of the possibility of more (and higher quality) alignments to the updated consensus sequence from each previous round under the assumption that the number of variants reported by Quiver in each round reflected, in part, the number of errors left to correct in addition to true variants.

In the first round, Quiver dropped out contigs that have no coverage from the raw PacBio reads and we chose to leave them out. We noticed that, although contigs were dropped from Canu, DBG2OLC, Falcon, and Miniasm assemblies, there were no contigs dropped from ABruijn and SMARTdenovo assemblies. Moreover, Quiver reports lower-case letters for bases that do not have enough coverage (< 5 here) to compute a consensus on whereas consensus-computed bases are uppercase. Most assemblers contained contigs that were 100% lower-case, though these were typically quite small (e.g. $\leq$ 20 kb). ABruin and SMARTdenovo did not contain any contigs that were reported as 100% lower-case. These initial observations suggested that ABruijn and SMARTdenovo these assemblers already dispensed with low coverage contigs whereas the other assemblers were more conservative and retained some.

For the 6th and 7th round of Quiver polishing we lowered the minimum coverage required for Quiver to compute a consensus and identify variants from 5 to 3. This did not seem to impact most metrics for most assemblies in a negative or positive way. However, ALE measures for Canu and Falcon seemed to degrade whereas the measures for all metrics except REAPR continued to improve for PlatanusDBG2OLC assemblies. Therefore, for each assembly (for all 50 assemblies), we compared the evaluation metrics from each Quiver round and selected the assembly version from the Quiver round with the highest average rank, opting for later rounds in cases of a tie. This resulted in selecting assemblies from Quiver rounds 3-7, with 13 and 19 of the 50 assemblies from rounds 5 and 7, respectively. Canu and Falcon assemblies tended to be selected for earlier rounds whereas ABruijn, Miniasm, PlatanusDBG2OLC, and SMARTdenovo assemblies tended to be selected in later rounds, seemingly reflecting whether or not the assemblers had pre-assembly read correction steps. After selecting the best version of each assembly from the Quiver rounds, all 50 assemblies were subject to 2 rounds of diploid-aware Pilon polishing with our Illumina dataset.

4.2.4.1 Iterative polishing with signal-level PacBio data using Quiver

The basic approach was to align all raw PacBio reads using PBalign, merging and sorting the alignments, and using Quiver. Each round used the previous round's polished output assembly as the input assembly. The number of variants in the GFF output file was counted after each round.

```
pbalign $BAX $REF $OUTPRE.cmp.h5 --forQuiver --tmpDir $TMPDIR --nproc
$THREADS

cmph5tools.py merge --outFile ${MERGEDCMP} ${CMPDIR}/*.cmp.h5

cmph5tools.py sort --deep ${IN_CMP} --tmpDir ${TMP_SORT}

samtools faidx $REF

quiver -j${THREADS} ${INPUT} -r ${REF} -o $OUTGFF -o $OUTFASTQ -o
$OUTFASTA --noEvidenceConsensusCall=lowercasereference --verbose
```

Prior to all alignment/polishing steps, for Canu assemblies, contigs in the unassembled file that consisted of at least 2 reads were added back to the assembly, and for both Canu and Falcon

assemblies, associated bubble contigs were added to the assembly. All contigs that were added back to the assemblies were given names to identify them after polishing for optional removal. For all assemblies that had spaces in the contig names, the first "word" before the first space was used as the contig name. All of these operations were accomplished with python scripts found at https://github.com/JohnUrban/sciara-project-tools:

"filterCanuFasta.py" and "fasta_name_changer.py".

This pipeline was automated for SLURM to process all 50 assemblies:
https://github.com/JohnUrban/sciara-project-tools/tree/master/slurmgear/pacbiotools/quiver

4.2.4.2 Evaluations during Quiver Polishing

Before Quiver polishing and after each round, the assemblies were evaluated with the same 7 metrics as described for short-read assemblies in Section 4.1.3. Briefly, those were (1) NG50, (2) the percentage of Illumina reads that mapped to the assembly, (3) LAP, (4) ALE, (5) FRC^{bam}, (6) REAPR, and (7) BUSCO. Importantly, where relevant, the metrics used the same input Illumina reads as was used for evaluating the short read assemblies to allow a direct comparison of scores.

2.2.4.3 Iterative polishing with Illumina short reads using Pilon

The basic approach was to build a bowtie2 index (Langmead and Salzberg 2012) for each assembly, map the paired-end illumina reads to each assembly with bowtie2, index the BAM alignment files with SAMtools, mark duplicates with Picard Tools, and use Pilon (Walker et al. 2014) to polish. Each round used the previous round's polished output assembly as the input assembly.

```
bowtie2-build $ASM $BASE

bowtie2 -p $P --very-sensitive -N 1 --minins 0 --maxins 1000 -x $BT2 -1
$R1 -2 $R2 | samtools sort --threads $P -o ${PRE}.bam

samtools index ${PRE}.bam

java -Xmx${JX} -jar $JAR MarkDuplicates INPUT=${PRE}.bam
OUTPUT=${PRE}.markdup.bam METRICS_FILE=${PRE}.metrics.txt
REMOVE_DUPLICATES=false ASSUME_SORTED=true

samtools index ${PRE}.markdup.bam

java -Xmx${JX} -jar $PILONJAR --genome $ASM --output $PRE --changes --
frags ${READS} --diploid --fix bases --nostrays
```

This Pilon pipeline was automated for SLURM to process all 50 assemblies using shell scripts also found at:
https://github.com/JohnUrban/sciara-project-tools/tree/master/slurmgear/pilon

4.2.5 Evaluations of the Initial 50 long read assemblies

We used an expanded set of evaluations at this point to leverage the long-read and optical map datasets. More information on each metric listed below can be found in the relevant sub-sections that follow. Note that the "*" indicates metrics also used in Sections 4.1.3 and 4.2.4.2. All metrics used at this stage were (in order of appearance of matrixes in Figure 2E):

(01) *BUSCO for gene content,
(02) *the percent of Illumina reads that mapped,
(03) *LAP with Illumina data,
(04) *ALE with Illumina data,
(05) *REAPR percent error-free bases with Illumina data,
(06) REAPR mean base score with Illumina data,
(07) *FRC^{bam} number of features with Illumina data,
(08) FRC^{bam} number of features per megabase with Illumina data,
(09) Number of changes made by Pilon in the final polishing round using Illumina data,
(10) the sum of scores when aligning BioNano optical maps,
(11) the number of bases in the assembly covered by optical maps,
(12) the total optical map coverage,
(13) the percent of optical maps that mapped,
(14) the number of Structural Variants (SVs) found with using the Nanopore data,
(15) the number of SVs found with PacBio data,
(16) the number of SVs with combined long read data,
(17) the span of all SV lengths found with Nanopore data,
(18) the span of all SV lengths found with PacBio data,
(19) the span of all SV lengths found with combined data,
(20) the average number of split alignments per Nanopore read,
(21) the percent of Nanopore reads that mapped,
(22) the average number of split alignments per PacBio read,
(23) the percent of PacBio reads that mapped,
(24) *NG50 contig length,
(25) Expected contig length,
(26) Max contig length,
(27) LG50 (minimum number of contigs that contain $\geq$ 50% of the genome)

The categories of Illumimna and length metrics were slightly expanded, and new categories of long-read and optical map metrics were added. For the long-read metrics, percent mapped serves as a completeness measure (as was the case for short reads as well). Moreover, SVs and the average number of split-read alignments serve as proxies that are proportional to the number and extent of mis-assemblies in each assembly since, although all assemblies (and the data) may share some real SVs, increasing numbers of mis-assembled regions will result in higher numbers of SVs detected. We aligned the raw optical maps to the assemblies, and used alignment statistics (scores, span, coverage, and percent mapped) as proxies for evaluating the long-range structural integrity of each assembly as described below.

4.2.5.1 Size statistics
Used asm-stats.py from https://github.com/JohnUrban/sciara-project-tools

We looked at four statistics:
- The number of contigs
- The maximum contig length
- The NG50 given the expected genome size of 280 Mb
  - Half of the expected genome size is contained on contigs of this length and longer
- The LG50
  - Half of the expected genome size is on this many contigs
- The expected contig length (E) given the expected genome length
  - The expected contig length statistic is an alternative to NG50 (Salzberg et al. 2012)

4.2.5.2 BUSCO completeness metrics

4.2.5.3.1 BUSCOv1
```
BUSCO_v1.22.py -in $ASM -o $OUT -l $BV1_LINEAGE -m $BV1_MODE --cpu $CPU
```

4.2.5.3.2 BUSCOv3 with ODB9

```
LINEAGE=/Path/to/diptera_odb9
run_BUSCO.py --in $ASM -o $OUT -l $LINEAGE -m $BV3_MODE --cpu $CPU --limit
$REGIONLIMIT
```

We also tried Eukaryota, Metazoa, Arthropoda, Insecta, and Endopterygota. Results were similar in all cases. We used the Diptera results as it had the largest set of BUSCOs.

4.2.5.3 Illumina Metrics

4.2.5.3.1 Mapping Illumina reads back to each assembly
Same as for Illumina assemblies: percent Illumina reads mapped taken from Bowtie2 output.

4.2.5.3.2 ALE

```
ALE ${BAM} $REF ${BASE}.ALE.txt
```

4.2.5.3.3 FRC^{bam}

```
FRC --pe-sam $BAM --pe-max-insert 800 --genome-size 292000000 --output
${BASE}.frc
```

We looked at both the number of features detected as well as the rate, number of features per megabase to normalize by assembly size.

4.2.5.3.4 LAP

```
calc_prob.py -p $P -a $REF  -q -1 $R1 -2 $R2 -X 800 -I 0 -o fr -m 432 -
t 75 -b $BT2 > ${BASE}.prob

sum_prob.py -i ${BASE}.prob > ${BASE}.lapscore
```

4.2.5.3.5 REAPR

```
reapr facheck $REF ${BASE}_renamed
reapr perfectmap ${BASE}_renamed.fa $R1 $R2 432 perfect
reapr smaltmap ${BASE}_renamed.fa $R1 $R2 mapped.bam  -n $P
reapr pipeline ${BASE}_renamed.fa mapped.bam output_directory perfect

zcat 03.score.per_base.gz | awk '{s+=$3}END{print s/NR} > per-base-
mean-score.txt
```

We looked at:
- Percent error-free bases
- Mean base score

4.2.5.3.6 Pilon
We looked at the number of changes made in the final round of Pilon polishing (Walker et al. 2014) with the assumption that fewer changes needed to polish the assembly, the higher the quality of the assembly consensus. We found that re-running Pilon to count the number of variants was correlated with the number of changes made (assemblies with fewer variants generally needed fewer changes). For subsequent evaluations we also looked at the number of confirmed bases (supported by provided evidence) from Pilon's output in each assembly as a percent of the expected genome size.

4.2.5.4 PacBio and MinION long read metrics
We used the long reads from PacBio and Oxford Nanopore both separately and in combination to evaluate the 50 assemblies. We used the long read aligner BWA (Li and Durbin 2009) and the structural variation (SV) algorithm called "Sniffles" (Sedlazeck et al. 2018) [https://github.com/fritzsedlazeck/Sniffles] to detect the number SVs in each assembly. Reasoning that the number of SVs is a combination of both real variants and errors in the assemblies, assemblies were ranked higher for having lower numbers of reported SVs. We also separately considered the sum of the interval lengths reported to be involved in SVs and refer to this as SV span. For the same reasoning as above, assemblies were ranked higher for having shorter SV spans. In some cases, assemblies can have short spans, but high numbers of reported SVs and vice versa. We called SVs separately for PacBio and ONT reads as well as when combining them.

```
# Index assembly with BWA
bwa index $ASM -p $BASE

# Align reads to assembly with BWA
## TYPE=pacbio or TYPE=ont2d
bwa mem -t $MTHREADS -M -x $TYPE $BWAIDX $FASTQ | samtools sort -T
$TYPE --threads $MTHREADS -o $TYPE.bam
```

```
# Optionally merge pacbio and ont alignments
samtools merge --threads $P combined.bam $PBBAM $ONTBAM

# Call SVs with Sniffles
# BAM=pacbio.bam or BAM=ont.bam or BAM=combined.bam
sniffles -m $BAM -b $BEDPE.bedpe
```

We additionally considered (i) the percentage of PacBio and Oxford Nanopore reads that aligned to each assembly, ranking assemblies higher for having a higher percentage, and (ii) the ratio of the number of alignments to the number of unique reads represented in those alignments. The latter can be thought of as the expected number of split alignments per read. Since reads are sometimes split to align in separate places or in different orientations, the ratio is nearly always greater than 1. Similar to the more formal SV results from Sniffles above, we reasoned that the number of split alignments for a given read is a result from true biological variation, errors in the reads or alignments, and mis-assemblies. Since the first two are constant across all conditions, the only variable is the number of mis-assemblies. Thus, the closer to 1 this ratio is, the lower the rate of split alignments, and the higher a given assembly was ranked. This pipeline was automated using shell scripts found at https://github.com/JohnUrban/sciara-project-tools.

4.2.5.5 BioNano optical map metrics

We used the restriction map aligner called "maligner" (Mendelowitz et al. 2015) to align the raw BioNano optical maps to all 50 assemblies. Maligner reports an "M-score" for each alignment. We ranked assemblies using combined M-scores, total span across the assembly from alignments (sum of coordinates with at least 1 alignment over it), total coverage as determined by summing the reference interval lengths for each alignment, and the number of BioNano maps that could be aligned (normalized as percent of all maps).

Aligning BioNano Maps with Maligner

Merge all RawMolecule BNX files (all.bnx) using RefAligner utility from BioNano Genomics

```
./RefAligner -bnx -merge -i scia_copr_2013_031_1P_2016-04-
13_15_45/Detect\ Molecules/RawMolecules.bnx -i
Scia_copr_2013_032_1P_2016-04-12_10_39/Detect\
Molecules/RawMolecules.bnx -i Scia_copr_2013_032_1P_2016-04-
12_15_55/Detect\ Molecules/RawMolecules.bnx -i
Scia_copr_2013_032_1P_2016-04-13_11_27/Detect\
Molecules/RawMolecules.bnx -i Scia_copr_2013_032_1P_2016-04-
14_11_49/Detect\ Molecules/RawMolecules.bnx -o all -minSNR 2.75 -minlen
150 -minsites 8 -MaxIntensity 0.6
```

Convert BNX to Maligner input (bnx2maligner.py from https://github.com/JohnUrban/sciara-project-tools)

```
bnx2maligner.py -b all.bnx > bionano.maps
```

smooth the maps for maligner alignment using utilities from Maligner

```
smooth_maps_file -m 1000 bionano.maps > bionano.smoothed.maps
```

Split up maps to align them to assemblies in parallel (split into 48 files)

```
mkdir split
cd split
split -l 4079 -d ../bionano.smoothed.maps bionano.smoothed.maps
```

Convert assembly to smoothed map for Maligner using Maligner utilities (BssSI = CACGAG)

```
    make_insilico_map -o $ASM_OUT_PFX $ASM_FASTA CACGAG
smooth_maps_file -m 1000 ${ASM_OUT_PFX.maps > ${ASM_OUT_PFX.smoothed.maps
```

4.2.5.6 Selection for BioNano scaffolding

To select a final subset of assemblies for BioNano hybrid scaffolding, we sorted the assemblies by taking mean ranks across combinations of all 27 evaluation metrics used on the assemblies. Since it was possible that some assemblies have higher mean ranks due to a intrinsic weighting biases present across this set of 27 evaluations, we tried 40 different combinations of the 27 metrics with as few as 6 metrics in one combination and with as many as all 27 in another. The first twenty combinations did not include the size statistics ranks of NG50, LG50, expected size, and longest contig. This was done to uncover assemblies that ranked high without giving preference to more contiguous assemblies. The second set of 20 combinations did include size rankings. For both the first and second sets of twenty, the trend was roughly to use fewer metrics from the first to last combination. Moreover, since different categories (BUSCO, Illumina, PacBio, ONT, BioNano, size statistics) tended to favor different assemblers (mainly Canu or Falcon), the trend was to converge to more similar numbers of representatives from each category. At least one metric from each category was represented in all combinations except in the first twenty where size statistics were excluded.

4.2.5.7 Automating the battery of metrics

As there were many assemblies and a battery of metrics, JMU created scripts to automate launching jobs in SLURM to do them. This eventually evolved into a tool called “Battery” (https://github.com/JohnUrban/battery) that works with the SLURM job manager to launch all the jobs needed for each evaluation metric (with jobs further along a pipeline given dependencies on upstream jobs completing successfully) given a list of assemblies. The ‘battery’ tool includes the metrics we used in this paper in addition to exploratory options, such as using REAPR with long reads, that we introduced later. This tool (Battery) was designed only to facilitate our own analyses and will not necessarily work out of the box on other systems where SLURM is configured differently. Others interested in using Battery may also need to change some hard-coded defaults. The outputs from Battery can be collected, then analyzed and visualized in R.

4.2.6 BioNano Scaffolding

4.2.6.1 BioNano CMAP assembly:
BioNano genomic consensus maps (CMAPs) were assembled using an overlap layout consensus method with either all molecules >100 kb or all molecules >150 kb as input. A p-value threshold of 2.6e-9 was used with BioNano Pipeline Version 2884 and RefAligner Version 2816 (BioNano Genomics). The genome-wide optical maps assembled with the molecules >150 kb were slightly longer than when using all molecules >100 kb, with a map N50 of 712 kb and a cumulative length of 325.5 Mb. Thus, we chose to use only molecules >150 kb. There were 585 consensus genome maps (CMAPs) constructed from the BioNano image data, using the Canu assembly as a reference to estimate noise parameters during construction. Canu long read assembly contigs were converted into in silico maps and aligned to the BioNano CMAPS revealing a 266 Mb breadth of alignment (unique alignments) and 278 Mb total length of alignment (which includes redundant alignments of possibly repetitive DNA). A majority of unique alignments as seen here typically indicates a high quality sequence assembly.

4.2.6.2 BioNano hybrid scaffolding:
In hybrid scaffolding, BioNano CMAPs (assembled above) are linked when supported by long read contig overlaps, and long read contig maps are linked when supported by BioNano CMAP overlaps. The result is a genome-wide map, or hybrid scaffold, that is typically more contiguous than either of the input maps. The long read sequence assemblies and BNG optical maps were used with hybridScaffold.pl version 4741 (BioNano Genomics) to create genome-wide hybrid scaffolds. BssSI restriction maps of long read sequence contigs were generated in silico. In silico Consensus Maps (in silico CMAP) were only created for scaffolds > 20 kbp that contained > 5 BssSI sites. A p-value of 1e-10 was used as a minimum confidence value to output initial alignments (BNG CMAP to in silico CMAP) and final alignments (in silico CMAP to final hybrid CMAP). A p-value of 1e-13 was used as minimum confidence value to flag chimeric/conflicting alignments and to merge alignments. The final assemblies include all super-scaffolds from hybridScaffold.pl and contigs that were not super-scaffolded.

4.2.6.3 Obtaining gap intervals for gap sizing:
We used a custom script from https://github.com/JohnUrban/sciara-project-tools to define gap intervals. Since gap intervals from BNG scaffolding include recognition sequences we merged gaps were within 50 bp of each other using BEDTools.

```
scf-N-to-BED.py $f | sortBed -i - | mergeBed -i - -d 50
```

4.2.6.4 Scaffold comparisons and evaluation
Scaffolded assemblies were evaluated with the 27 metrics used to evaluate the 50 long-read assemblies as described in Section 4.2.5 above (automated with Battery as described above).

To compare, scaffolded assemblies were aligned together using Minimap2:

```
minimap2 --secondary=no -x asm20 ${A} ${B} B-to-A-asm20-nosecondary.paf
```

Overall, the metrics and assembly alignments indicated that scaffolds derived from the same assembler were very similar. Only one representative of each was chosen to move forward with. We also noted that many of the BioNano joins unique to one assembly were already present as a single longer contig in the other, thereby supporting the BioNano join.

4.2.7 Assembly refining:
Gap-filling, polishing, contamination removal, and haplotig identification

4.2.7.1 Final polishing, gap-filling, and meta-scaffolding

Scaffolds were iteratively polished and gap-filled as described below. Polishing steps were used to polish newly filled gaps and facilitate further gap filling.

Upper case note: Some steps, such as Quiver polishing, output assemblies that include both lower- and upper-case letters. Since some programs (e.g. BLAST) may treat upper- and lower-case letters differently, we converted assemblies to 100% uppercase prior to BNG scaffolding and between all rounds of polishing, gap-filling, and meta-scaffolding, and before any/all evaluations:

```
fastaCaseMaker.py -f input.fasta > output.fasta
```

First set of Quiver rounds on hybrid scaffolds:
We worked with two sets of BioNano hybrid-scaffolded long read assemblies: one from Canu, and one from Falcon. Both were iteratively polished and gap-filled in the following way. Bionano scaffolds were polished with Quiver and the PacBio datasets five times to help correct the consensus near gaps. In the first round, Quiver detected 2177 and 3167 in the Canu and Falcon assemblies respectively, whereas there were 1723 and 2338 variants detected in each after the fifth round. Thus, the number of variants were similar to that found prior to BioNano scaffolding as one might expect.

Operations were the same as in section "**4.2.4.1 Iterative polishing with signal-level PacBio data using Quiver**".

First set of PBJelly rounds on Quiver-polished hybrid scaffolds:
The hybrid scaffolds were then subject to three rounds of gap filling with PBJelly (PBSuite_15.8.24) (English et al. 2012), using the combined set of PacBio and MinION reads in a fastq file. PBJelly was instructed to use only BLASR alignments with 75% identity or greater, and to only fill N-gaps (i.e. not attempt to further scaffold the assemblies).

BLASR instructions in Protocol.xml for all 3 rounds:

```
-minMatch 8 -sdpTupleSize 8 -minPctIdentity 75 -bestn 1 -nCandidates 10
-maxScore -500 -nproc 48 -noSplitSubreads
```

Commands:

```
Jelly.py setup Protocol.xml
Jelly.py mapping Protocol.xml -x "-n 20"
Jelly.py support Protocol.xml -x "--capturedOnly"
Jelly.py assembly Protocol.xml -x "--nproc=20"
Jelly.py output Protocol.xml
```

Second set of Quiver rounds:
To polish the consensus of the filled-gaps, we again ran Quiver for 3 rounds (Canu) or 4 rounds (Falcon). In the first round, Quiver detected 4369 and 8360 in the Canu and Falcon assemblies respectively, with the increases detected here resulting from new alignment regions within filled and partially-filled gaps. There were 1564 and 2962 variants detected in each after the final rounds, back to numbers similar to those pre-gap filling.

Operations were the same as in section "**4.2.4.1 Iterative polishing with signal-level PacBio data using Quiver**".

Second set of PBJelly rounds:
Next, given that the filled-in and and partially-filled-in gaps were now polished, we did three extra rounds of PBJelly gap filling. In the first round, PBJelly was instructed exactly as above (to use only BLASR (Chaisson and Tesler 2012) alignments with 75% identity or greater, and to only fill N-gaps). In the second round, that was adjusted to use BLASR alignments with 65% identity or greater, but otherwise the same. In the third round, 65% identity was again used as the cutoff, but we allowed PBJelly to link contigs together in this round (i.e. gap filling and scaffolding). In this last round, 83 of the 88 "filled" were contig joins for the Canu-derived assembly and 27 of the 45 "filled" were contig joins for the Falcon-derived assembly.

BLASR options in round 1:
```
-minMatch 8 -sdpTupleSize 8 -minPctIdentity 75 -bestn 1 -nCandidates 10
-maxScore -500 -nproc 48 -noSplitSubreads
```

BLASR options in rounds 2 and 3:
```
-minMatch 8 -sdpTupleSize 8 -minPctIdentity 65 -bestn 1 -nCandidates 10
-maxScore -500 -nproc 48 -noSplitSubreads
```

Commands for rounds 1 and 2:
```
Jelly.py setup Protocol.xml
Jelly.py mapping Protocol.xml -x "-n 20"
Jelly.py support Protocol.xml -x "--capturedOnly"
Jelly.py assembly Protocol.xml -x "--nproc=20"
Jelly.py output Protocol.xml
```

Commands for round 3 differed only at the "support" step where "--capturedOnly" was left off to allow meta-scaffolding:
```
Jelly.py support Protocol.xml
```

Final Rounds of Quiver and Pilon Polishing:
The updated gap-filled scaffolds were polished with another three rounds of Quiver and twelve rounds of Pilon. Generally speaking, for both Quiver and Pilon, the polishing rounds after the first round resulted in relatively few additional changes. In the first round, Quiver detected 4408 and 10992 variants in the Canu and Falcon assemblies respectively, with the increases detected here again resulting from new alignment regions within filled and partially-filled gaps. Again, there were similar numbers of variants in the final Quiver round to the quantities seen prior to any gap-filling (1652 and 2488 for Canu and Falcon, respectively). We also monitored the number of changes to the consensus sequence in each Pilon round. In the first, there were 23361 and 20638 changes to the consensus for Canu and Falcon, but only 18 and 27 in the final round. This translates to approximately 1 change per 16.9 Mb and per 11 Mb of non-gap sequence in the final round for Canu and Falcon, respectively.

Quiver: Operations were the same as in section "2.2.4.1 Iterative polishing with signal-level PacBio data using Quiver". Three rounds were performed.

Pilon: Operations were the same as in section "2.2.4.2 Iterative polishing with Illumina short reads using Pilon". Twelve rounds were performed.

Overall, we observed that various metrics improved from the scaffolds to the meta-scaffolds. For example, the number of "translocations" detected by Sniffles as well as the number of short and long reads spanning more than one contig all went down.

4.2.7.2 Identifying contaminating contigs and scaffolds

Labeling bacterial contigs:
Bacterial and other contaminating contigs and scaffolds were independently identified in both the final Canu and Falcon scaffolds in the following way. Here we will refer to all separate sequences in the final scaffolded assembly as "scaffolds" even though some contigs were not scaffolded, and might more correctly be still called contigs.

The final scaffolds were first split up into one scaffold per fasta file:

```
multiFasta2manyFastas.py -f ${ASM} -d ${TIGDIR}
```

This was done such that issues with BLAST (Altschul et al. 1990) on one contig, such as hanging on one contig until it was killed for exceeding an allotted time limit on SLURM, would not affect the the analysis of subsequent contigs in a file.

Next each scaffold was broken up into 1000 bp windows, and the windows were partitioned into their own separate files with a maximum of 292 windows per FASTA file. This was done for similar reasons as were provided above for splitting the assembly into its component scaffolds. Specifically, it was done to prevent situations where one area of a scaffold causing BLAST issues prevents adequate taxonomy information being collected for that scaffold. Moreover, it exhaustively collects taxonomy information in discrete 1 kb intervals across the scaffold, allowing us to confirm that each contig component of a scaffold was in agreement with taxonomy identification later on. For example, we could make sure that a scaffold labeled as BlobTools (Laetsch and Blaxter 2017) as Arthropod/Dipteran did not contain a bacterial contig within it that was erroneously joined during BioNano or long read scaffolding steps.

For each scaffold file, the following was done:

```
# There were giant N-gap sequences that were pre-filtered out of the
windows
# First gaps were found
scf-N-to-BED.py ${ASM} > gap.bed

# Then the scaffold was windowed, and only windows that had less than
75% overlap with a gap were retained (allowing the windows nearest to
gap edges to have no less than 250 bp) using BEDTools.
bedtools makewindows -w 1000 -s 1000 -g scaf.size | intersectBed -f
0.75 -v -a - -b gap.bed > windows.bed

# The sequence for each retained window was then obtained in a single
FASTA (with BEDTools).
fastaFromBed -fi ${ASM} -bed windows.bed > windows.fasta

# The windowed scaffold FASTA was then split into multiple files such
that they contained no more than 292 window sequences for the given
scaffold.
splitFastA.py -f windows.fasta -n 292 -o winFaDir
```

Finally, all of the windowed fastas from all of the scaffolds were subject to independent BLASTN runs against the entire nucleotide database:

```
blastn -task blastn -query $Q -db $NT \
 -outfmt '6 qseqid staxids bitscore std sscinames sskingdoms stitle' \
 -culling_limit 5 \
 -num_threads 8 \
 -evalue 1e-10 \
 -out ${BLASTDIR}/${BASE}.blastout
```

The BLASTN results from all were then combined into a single file and processed to have the correct scaffold names and coordinates:

```
# Combine raw results
cat blast_results/* > all_windows.blastout


# Process results into a taxonomy file for BlobTools

awk 'OFS="\t" {gsub(/\ /,"_"); gsub(/__/,""); sub(/:/,"\t"); sub(/-
/,"\t"); print $1,$4,$5}' all_windows.blastout | sort -k1,1 -k2,2n -
k3,3nr > all_windows_blastn_NT_qseqTaxBit.txt


# Process results into a BED file of taxonomy annotations in 1 kb
windows across the assembly (for visual inspection, etc)

awk 'OFS="\t" {gsub(/\ /,"_"); gsub(/__/,""); sub(/:/,"\t"); sub(/-
/,"\t"); print $1,$2+$11-
1,$2+$12,$6";"$4";"$5";"$7";"$8";"$9";"$10";"$13"-
"$14";"$15";"$16";"$17";"$18}' all_windows.blastout | sortBed -i - >>
all_blastn_NT_annotation.bed
```

BlobTools was then run the same as for short read assemblies described in section 2.1.4 (*Contamination analyses for the selected Platanus short read assembly*). We looked at the taxonomy information when using all of the BLAST results as well as when removing any BLAST hit labeled as Bradysia coprophila (*Sciara*). Both of gave essentially identical taxonomy-labeling results at the phylum level with the only differences being that some contigs that only had Bradysia hits were now unlabeled. For both approaches and for both the final Canu and Falcon scaffolds, 96.6-98% of the assembly was labeled as Arthopoda, ~98% of which was also labeled as Dipteran. We also looked at coverage levels from our male embryo Illumina, PacBio, and MinION data. All gave similar BlobPlot results. Moreover, we looked at male pupal genome coverage levels from BioNano optical maps, noting that there were no bacterial-labeled contigs with any BioNano coverage (consistent with no bacterial-labeled contigs being scaffolded by this dataset). Finally, we also performed these BlobTools analyses on the component contigs of the scaffolds independently to ensure that there weren't scaffolds labeled as Arthropod that had a bacterial contig within them nonetheless. There were no instances of this. Bacterial and arthropod-labeled sequences scaffolded independently of each other.

Overall, we used only the taxonomy information to filter scaffolds. Bacterial-labeled scaffolds (predominantly labeled as Rickettsia) were removed from the final scaffolded assemblies. Everything else was kept in with the caveat that only arthropod-labeled scaffolds were used to train gene predictors and to find motifs associated with DNA modification (see those sections below for more details).

The custom python scripts used above and SLURM scripts to automate the analysis can be found at: https://github.com/JohnUrban/sciara-project-tools

4.2.7.3 Identifying primary and associated sequences

Identifying "remove_tigs" in the final assemblies

As discussed previously (**Section 4.2.4.1 Iterative polishing with signal-level PacBio data using Quiver**), both Canu and Falcon assemblies were fed back additional contigs to ensure all haplotypes/etc were present to avoid forcing incorrect alignments that lead to errors in polishing. For Canu the additional contigs were contigs composed of 2 or more reads. For Falcon, these were the associated bubble contigs. While their names were marked for removal in early steps, subsequent scaffolding steps obfuscated the naming scheme. To re-identify them, we chose to align the original contigs marked for removal back to the final assemblies with minimap2 (Li 2018) to re-identify them therein:

```
X=asm10
minimap2 --secondary=no -x ${X} -c ${ASM} rmseq_tigs.fasta >
rmseq2step01scaffs-${X}.paf
```

Adjacent query alignments from the same query mapping to the same target were merged using our custom script: filterpaf.py (https://github.com/JohnUrban/sciara-project-tools), and further merged with BEDtools (Quinlan and Hall 2010).

```
filterpaf.py -i rmseq2step01scaffs-${X}.paf --merge > merge-${X}.paf

awk 'OFS="\t" {print $6,$8,$9,$7,$1,$3,$4,$2, $5, $10,$11,$12}' merge-
${X}.paf | sortBed -i - | mergeBed -d 1000 -i - -c 4,5,6,7,8,9,10,11,12 -o
distinct,collapse,min,sum,sum,collapse,sum,sum,mean | awk '{OFS="\t"; print
$5,$8,$6,$7,$9,$1,$4,$2,$3,$10,$11,$12}' > targetmerged-${X}.paf
```

We classified the merged alignments as being the "same" contig (i.e. it did not change much through scaffolding steps) or as a scaffold containing the contig. To be called "same" we required either (i) the smaller of the query or target contig sizes to be at least 80% the size of the longer one and required at least 50% identity overall or (ii) at least 60% for size and at least 60% for identity.

```
awk '($2/$7 >= 0.8 && $2/$7 <= 1.2 && $10/$2 >=0.5 && $10/$11 >= 0.5) ||
($2/$7 >= 0.6 && $2/$7 <= 1.66 && $10/$2 >=0.75 && $10/$11 >= 0.75)'
targetmerged-${X}.paf > same-${X}.paf
```

Other alignments were marked as containments:

```
cut -f 1 same-${X}.paf | tr "," "\n" | sort | uniq > found-${X}.txt
grep -w -f <(grep -v -w -f found-${X}.txt <(grep ">" rmseq_tigs.fasta ) | awk
'{sub(/>/,""); print}') targetmerged-${X}.paf | sort -k1,1 -k3,3n >
containments.paf
```

All 339 and 184 contigs marked for removal in the initial Canu and Falcon assemblies, respectively, were found in the final scaffolds mostly unchanged. For both, the majority of contigs remained as singletons through the scaffolding steps (i.e. were not part of longer scaffolds).

Identifying candidate redundant contigs/haplotigs using Minimap2 alignments.

Long read assemblies tend to output many relatively short contigs that map within longer contigs, and these might be considered redundant. Moreover, assemblies of diploid genomes can produce two contigs for heterozygous loci. Often associated/bubble contig are shorter than the primary contig they map inside (though these bubbles can also span > 100 kb). Falcon marks most of these in a separate file. Although we added them back, we re-identified them in the previous step. Therefore, we expect fewer 'redundant contigs' to be found in this step for Falcon than Canu, the latter which outputs all primary and associated contigs in a single fasta file.

We used Minimap2 to find mappings from all contigs to all other contigs. In latter steps we eliminated any self-self mappings present, merged nearby alignments from the same query on the same strand, and focused on shorter contigs that mapped inside longer contigs. For simplicity, we settled on "-x ava-ont" below. We found that this was the most sensitive way to identify candidate redundant contigs. However, "-x asm5", "-x asm10", and "-x asm20" combined yielded 3 additional hits. Manually inspecting them showed that they were typically on the borderline of what one might accept as redundant. We also tried other parameters such as both "-N 1000" and "-N 100 -p 1e-5 -f 0.001" with each "–x" value from above, and "-N 10000 -p 1e-5 -f 0.001" without specifying a "-x" value. Overall, we went with the option (`-x ava-ont`) that appeared to give our downstream steps the highest sensitivity, missing only 3 hits across all conditions combined.

For mapping:

```
minimap2 -c -t ${T} -N 1 -X -x ava-ont ${ASM} ${ASM} > ${B}
```

Alignments were then re-written such that every line had the longer contig first and shorter second, then clustered in a way to facilitate alignment merging:

```
awk '$1!=$6 {OFS="\t"; R=$2/$7; if (R>=1)
print $0; else print
$6,$7,$8,$9,$5,$1,$2,$3,$4,$10,$11,$12,$13,$14,$15,$16}' ${B} | sort -
k5,5 -k1,1 -k6,6 -k3,3n -k8,8n > sort.${B}

##rename
mv ${B} del.${B}
mv sort.${B} ${B}
```

A first pass of obtaining "redundant contig" names was performed by looking for alignments of shorter to longer contigs where at least 80% of the shorter contig was represented with at least 70% identity:

```
awk '($9-$8)/$7 >= 0.8 && $10/$11 >= 0.7' ${B} | cut -f 6 | sort | uniq >
names-pass-1.${B}
```

As a second pass, alignments were merged given the sort order above. The shorter contig alignments needed to occur on the same target. Gaps in the alignment along the query or target could not exceed 10 kb. Backtracking in a subsequent alignment, where the subsequent alignment starts at a position prior to the previous alignment's end position, could not exceed 10 kb, nor was it allowed to beyond the previous start site. To be considered as redundant, alignments (whether from merging or not) needed to span at least 80% of the shorter contig with at least 70% identity. Moreover, the ratio of the alignment size along the target and along the query had to be such that the shorter alignment was at least 70% the size of the longer alignment.

```
filterpaf.py --targetmerge --presorted --merge \
      --merge_rules 10e3,10e3,10e3,10e3,1 \
      -i ${B} > tmerge-10k-${B}
```

```
awk '($9-$8)/$7 >= 0.8 && $10/$11 > 0.7' tmerge-10k-${B} \
      > filt-tmerge-10k-${B}

awk '($9-$8)/($4-$3) >=0.7 && ($4-$3)/($9-$8) >=0.7' \
      filt-tmerge-10k-${B} | cut -f 6 |  sort | \
      uniq > names-similar-spans.filt-tmerge-10k-${B}
```

Size statistics for redundant contigs found by this method:

	**Canu**	**Falcon**
**Number of contigs**	336	348
**Total length**	5,571,839	5,111,051
**Max contig size**	53,932	109,991
**Min contig size**	1,134	889
**Mean contig size**	16,582.9	14,686.9
**Median contig size**	15,404.5	12,879.5
**Contig N50**	18,877	18,688
**Contig L50**	110	92
**Expected contig size**	20,371	23,840

For Canu, 190 of the 336 redundant contigs were also labeled as "remove tigs" from above, and these made up 45.6% of the total length.
For Falcon, 164 of the 348 redundant contigs were also labeled as "remove tigs" from above, and these made up 54.1% of the total length.

Further exploration of candidate redundant contigs with incorporation of coverage from each dataset using "purge_haplotigs"

a. PacBio reads were aligned to the assembly with Minimap2:

```
minimap2 -a -t ${THREADS} -x map-pb ${ASM} ${PACBIO} | \
samtools view -hF 256 | \
samtools sort -m 1G -T pacbio --threads $THREADS -o pacbio-minimap2.bam
```

b. Purge_haplotigs (Roach et al. 2018) was used to obtain coverage data:

```
purge_haplotigs  readhist  -b ${BAMPATH}  -g ${ASM} -t 12
```

c. The coverage data was used to define the means and standard deviations of the mixture of normal distributions seen in the data using mixtools in R. With these analyses we settled on appropriate coverage cutoffs for the next step.

d. Purge_haplotigs was used to flag contigs:

```
purge_haplotigs  contigcov  -i ${GENCOV}  -l 4  -m 33  -h 75  \
      -o coverage_stats.csv -j 80  -s 80
```

e. Purge_haplotigs was used to classify contigs as KEEP, HAPLOTIG, REPEAT, JUNK, and UNCLASSIFIED. Providing BED file with repeat locations from repeat annotation step did not change results.

```
purge_haplotigs  purge  -g ${ASM}  -c ${COVSTATS} -t ${THREADS} \
   -b ${BAM} -d [-repeats repeats.bed]
```

This approach largely agreed with our original minimap2 approach in step 3. Specifically, for both Canu and Falcon, 93-94% of the contigs called redundant in step 3 were identified as a category other than "KEEP" by purge_haplotigs (i.e. HAPLOTIG, JUNK, or REPEAT). As the "remove tigs" (Step 2) from Falcon were all associated haplotigs output in a separate FASTA file as part of the Falcon assembly process, we expected them to have been marked for removal by purge_haplotigs. In agreement with this expectation, of the 173 "remove tigs" not contained within a larger scaffold, 169 were identied as a class other than "KEEP", and all 173 were identified as "redundant" in step3.

Assigning contigs to the primary or associated assembly.
Overall, contigs labeled as "bacterial" are considered bacterial contigs and were not deposited on NCBI. Contigs that were not bacterial, not "remove contigs", not redundant, and were labeled as KEEP by purge_haplotigs are considered the primary set of contigs (primary assembly). All other non-bacterial sequences are considered "associated contigs". All primary and associated sequences were deposited on NCBI as a single assembly. Please contact us for a text file that partitions the contigs into primary and associated assemblies if interested.

4.2.7.4 Anchoring sequences into chromosomes using known, localized, and/or homologous sequences

The following previously known nucleotide sequences were aligned to the genome with BLAST:

- DNA Puff II/9A (DiBartolomeis and Gerbi 1989; Bienz-Tadmor et al. 1991; Urnov et al. 2002; Foulk et al. 2006)
- RNA Puff III/9B (Wu et al. 1993; Foulk et al. 2006)
- Ecdysone Receptor/minor DNA puff IV/12A (Foulk et al. 2013)
- Ultraspiracle/RNA puff IV/10A (Foulk et al. 2013)
- Hsp70 (Mok et al. 2001)
- ScoHet1 (Greciano et al. 2009)
- ScoHet2 (Greciano et al. 2009)
- rDNA (Pardue et al. 1970; Gerbi 1971; Crouse et al. 1977; Kerrebrock et al. 1989)
- B4/F7/Sccr (Escribá et al. 2011)
- Drosophila Mitochondrial Sequence (chrM from dm3/BDGP Release 5)

Regarding Hsp70:
In previous work (Mok et al. 2001), *Sciara* Hsp70 was cloned, though not sequenced, and hybridized to two sites on chromosome IV: regions 4A and 17C. All *Drosophila melanogaster* Hsp70 protein sequences were downloaded from FlyBase and aligned to the *Sciara* genome assemblies with tblastn, and all identified the same genomic regions and mapped across at least 90% of their lengths. There were four Hsp70 regions on three different primary contigs in both the Canu and Falcon assemblies rather than the expected two regions. Both assemblies have two unanchored contigs with an Hsp70 site and two other Hsp70 regions on a single contig associated with chromosome II (identified with DNA puff II/9A sequence), the latter of which was surprising given the hybridization results showed only two regions on chromosome IV. This result was not due to a misjoin introduced in any of the scaffolding steps since puff II/9A and the two flanking Hsp70 regions were part of the same contig prior to scaffolding in both assemblies. It also seems unlikely to be a mis-assembled contig as the genome structure around this locus is consistent in long read assemblies from each algorithm we used. Since the hybridization results that mapped Hsp70 to two sites on chromosome IV would have been dependent on nucleotide sequence similarity to the probe and to each other, we reasoned that the two chromosome IV regions should cluster together by similarity in sequence alignments. To test this, the coordinates for the four Hsp70 genomic regions were used to extract the associated Hsp70 sequences with BEDtools

(fastaFomBed) (Quinlan and Hall 2010). These were aligned to each other with BLAST using "-task megablast –qcov_hsp_perc X" where "X" was 75, 90, or 99. Under those conditions only sequences from the two unanchored two regions mapped to each other. Under more sensitive conditions (using "-task blastn" instead of "-task megablast and "–qcov_hsp_perc 75") we were able to get all to align. The two unanchored loci had the highest similarity, aligning across their full lengths at ~88.2% similarity. The two putative-chromosome-II Hsp70 sites each shared less than 70% DNA sequence similarity with any of the other three Hsp70 regions, one sharing only up to ~65% similarity to other Hsp70 sites across ~75% of its length. Since the hybridization results showed only two loci that must have been more similar to both the probe and to each other than to other Hsp70 sites, the most likely candidate locations in the genome assembly for the two loci seen in the hybridization results are the two yet-unanchored regions that share over 88% similarity with each other across their full lengths, and 70% or less similarity with other sites. Moreover, the lower similarity of the Hsp70-like sites anchored to chromosome II explains their absence in the hybridization results.

For rDNA, only small subsequences from *Sciara* are known. Therefore, in addition to these, a single unit of Drosophila rDNA (NW_007931121.1, positions 43508-55989) was used to identify all rDNA-containing contigs with BLAST (-evalue 1e-10 –qcov_hsp_perc 5).

Similarly, the Drosophila mitochondrial sequence (dm3) was used with Minimap2 (Li 2018) and BLAST (Altschul et al. 1990) to identify mitochondrial contigs. For the Canu assembly, Minimap2 identified a single candidate whereas BLAST identified four, one being the Minimap2 candidate. Two of the others were extraneous small contigs marked for removal and one was a tiny hit to a massive contig anchored to chromosome II. Of the three chrM candidates, all were considered JUNK by purge_haplotigs due to extremely high coverage. The minimap2 candidate was added back to primary assembly. For Falcon, Minimap2 identified three candidates whereas BLAST identified many more mitochondrial hits on many small contigs. We also inferred there must be a high number of mitochondrial sequences during repeat modeling when we noticed some de novo repeats from the Falcon assembly contained mitochondrial genes. We focused on the three Minimap2 hits. One was considered redundant and marked for removal in the above pruning analyses. Of the remaining two, one was more consistent with the structure from the Canu assembly. The other seemed to have inversions when compared to the Canu structure as well as to the other Falcon candidate. Therefore, the Falcon candidate most consistent with the Canu candidate was chosen to put back in the primary assembly.

4.2.7.5 Classifying/anchoring assembled sequences as autosomal or X based on diploid vs haploid coverage levels

X-linked contigs were contigs from the primary set in the pipeline below that had haploid level coverage across 80% of their length after (i) using Minimap2 to map PacBio reads back to the given assembly, (ii) using BEDtools genomecov to get coverage distributions for each contig, and (iii) defining cutoffs for haploid and diploid coverage levels based on the genomic distribution of coverage levels. Otherwise, contigs/scaffolds were called as autosomal. We tried this on the full set of scaffolds as well as the filtered "primary" set of contigs and scaffolds. There were a few examples of contigs that appeared to have mostly haploid level coverage in the full set, but diploid level coverage when reads were mapped only to the primary set after removing haplotigs, etc. However, the majority of the total length of sequence labeled as X-linked at the end of the pipeline was the same in both cases.

```
# Map reads
minimap2 -a -t ${THREADS} -x map-pb ${PRIMARY_ASM} ${PACBIO} | samtools
view -hF 256 | samtools sort -m 1G -T pacbio --threads $THREADS -o
pacbio-minimap2.bam
```

```
# Get coverage
bedtools genomecov -split -ibam pacbio-minimap2.bam > cov-split.hist
```

The genomic coverage distribution was viewed in R. We observed what appeared to be approximately 3 modes between the coverage levels of 0 and 65 (not unlike in step 4 above). We used the normalmixEM() from the "mixtools" package in R to learn the means and standard deviations of the three mixed distributions. The diploid coverage mean was between 40-42 (standard deviation ~5) and haploid coverage mean was approximately half the diploid mean as expected at ~20-22 (sigma ~8.2). The third mode was near 3-4x coverage, and was modeled to have mean of 3.2 (sigma ~2.2). We chose to set the lower cutoff to approximately three standard deviations below the haploid mean (7), the mid cutoff between haploid and diploid to 32 (~2 sigma above haploid mean and a little over 1 standard deviation under the diploid mean), and the high cutoff to around 4 standard deviations above the diploid mean (74).

The output from "bedtools genomecov" was parsed through to determine the proportion of sequence bases that had 0 coverage (0 coverage due to gaps was subtracted out), that were between 1 and the low cutoff, that were between the low cutoff and mid cutoff (haploid), that were between the mid cutoff and high cutoff (diploid), and that were above the diploid cutoff (collapsed repeat). If the majority of sequence bases were attributed to the haploid coverage level, we consider it a putative X. If $\geq$ 80% of the sequence bases for a contig were attributed to haploid coverage, we considered it a confident X call. We only considered confident X calls as X-linked/anchored moving forward.

4.3 Transcripts, genes, repeats, and a final genome assembly choice

4.3.1 Transcriptome Assemblies

4.3.1.1 De novo Transcriptome assembly using Trinity

A total of 18 strand-specific, 100 bp paired-end RNA-seq datasets were collected (see Supplemental Table S2). For each sex there were three mixed stage embryo replicates, and two replicates each of 4th instar larvae, mixed stage pupae, and mixed adults. All 18 samples were combined to generate a single transcriptome assembly with Trinity (version: trinityrnaseq_r20140413p1) (Grabherr et al. 2011) that we refer to as the “general transcriptome”.

```
Trinity --seqType fq --JM $JM --left $L --right $R --SS_lib_type RF --
CPU $P --trimmomatic --quality_trimming_params
"ILLUMINACLIP:/Path/to/neb_primers.fa:2:30:10 LEADING:5 TRAILING:5
MINLEN:36" --bflyHeapSpaceMax 28G --bflyCPU 8 --bflyHeapSpaceInit 10G
```

This transcriptome assembly was used both in genome assembly evaluations and in gene annotation.

4.3.1.2 Genome-guided transcriptome assembly using HiSat2 and Stringtie

We had 18 RNA-seq samples as described in Supplemental Table S2. For assembling with StringTie (Pertea et al. 2015), we tried a few approaches and used BUSCO (Simão et al. 2015), RSEM-Eval (Li et al. 2014), and Transrate (Smith-Unna et al. 2016b), to help choose an assembly for use with Maker2 (Holt and Yandell 2011) gene annotation below. First, each RNA-seq sample was independently aligned to either the the Canu or Falcon genome assembly (with all scaffolds and contigs present) with HiSat2 (Kim et al. 2019) and assembled with StringTie in the following way:

```
hisat2 --rna-strandness RF -p ${THREADS} --dta -x ${HIDX} -1 ${R1} -2
${R2} -S ${R}.sam
samtools sort -@ ${THREADS} -o ${R}.bam ${R}.sam
rm ${R}.sam
stringtie --rf -p ${THREADS} -o ${R}.gtf -l ${R} ${R}.bam
```

The independent assemblies were merged as following (approach 1):

```
readlink -f *.gtf > mergelist.txt
stringtie --merge -p ${THREADS} -o approach_1.gtf mergelist.txt
```

We also tried merging alignments prior to assembly the following way (approach 2):

```
samtools merge -@ ${THREADS} -b bam.fofn merged.bam
stringtie --rf -p ${THREADS} -o approach_2.gtf -l merged merged.bam
```

Finally, we also tested merging independent assemblies into the assembly from merged reads (approach 3):

```
readlink -f independent_asms/*.gtf > mergelist.txt
readlink -f asms_from_merged_reads.gtf >> mergelist.txt
stringtie --merge -c 0 -F 0 -T 0 -f 0 -p ${THREADS} -o approach_3.gtf
mergelist.txt
```

We tested these three approaches on both the Canu and Falcon assemblies, generating six total genome-guided transcriptome assemblies that we then pared down to only 2 (one for each assembly) using transcriptome evaluations.

Transcript sequences were obtained from the outputs by:

```
gffread ${MERGED} -w ${TRANS} -g ${ASM}
```

When needed, GTFs were converted to GFFs by:

```
gffread -E file.gtf -o- > file.gff
```

4.3.2 Evaluations of transcriptome assemblies

We generated seven transcriptome assemblies: the de novo Trinity assembly and the six StringTie assemblies of which three were guided by the Canu genome assembly, and three were guided by the Falcon assembly. We evaluated the transcriptome assemblies using BUSCO (Simão et al. 2015), RSEM-Eval (Li et al. 2014), and TransRate (Smith-Unna et al. 2016) in the following ways.

4.3.2.1 BUSCO :
We determined transcriptome completeness by searching for the 2799 Dipteran BUSCOs expected.

```
run_BUSCO.py --in ${ASM} -o busco -l ${DIPTERA} -m transcriptome --cpu
9 -sp fly -z --augustus_parameters="--progress=true"
```

4.3.2.2 RSEM-Eval:
Transcriptomes from Drosophila melanogaster and Anopheles were used to get transcript length parameters:

```
rsem-eval-estimate-transcript-length-distribution anopheles-gambiae-
pesttranscriptsagamp46.fa agam.txt

rsem-eval-estimate-transcript-length-distribution dmel-all-transcript-
r6.14.fasta dmel.txt

cat agam.txt dmel.txt | awk '{s+=$1; e+=$2}END{print s/NR"\t"e/NR}' >
agam_dmel_means.txt
```

Parameters from each:

	MEAN TRANSCRIPT LENGTH	STANDARD DEVIATION
**DROSOPHILA**	2881.22	2921.29
**ANOPHELES**	1860.19	1912.83
**MEAN**	2370.71	2417.06

SAMtools (Li et al. 2009) was used to estimate the average insert sizes for each RNA-seq sample from properly aligned read pairs, from reads aligned with HiSat2 for StringTie assemblies as described above. Given the set of average insert sizes from all samples, the average of averages (330 bp) was used.

```
samtools stats ${reads.bam} > ${b}.stats
```

Finally RSEM-Eval was run given (i) the Read1/Left and Read2/Right reads for all paired-end RNA-seq samples, (ii) the transcript length parameters estimated from Drosophila and Anopheles transcriptomes, and (iii) the insert size average estimated from all RNA-seq samples.

```
rsem-eval-calculate-score -p 9 --transcript-length-parameters
agam_dmel_means.txt --paired-end ${LEFT} ${RIGHT} ${ASM} Sciara 330
```

4.3.2.3 TransRate:

TransRate (Smith-Unna et al. 2016b) can be provided a reference proteome to provide a completeness measure. For example, it reports the number and proportion of transcripts or reference proteins that contain Conditional Reciprocal Best BLAST (CRBB) hits. It also reports the proportion of amino acids in the reference proteome covered by transcripts. For direct comparison between transcriptome assemblies, we focused on the proportion of reference proteins with CRBB hits given each transcriptome assembly (similar to the number or proportion of complete BUSCOs) as well as the proportion of the reference proteome covered by transcripts from each assembly. As a reference proteome we tried both the entire set of proteins that we provided Maker2 during the annotation step as part of the homology evidence (see below) and a subset of that, which included all reviewed UniProt arthropoda proteins. Both references yielded the same trends. TransRate also calculates an assembly score based on alignments of the RNA-seq data. The Read1/Left and Read2/Right reads for all RNA-seq samples were provided for this.

```
transrate --assembly ${ASMS} --left ${LEFT} --right ${RIGHT} --threads
${NTHREADS} --reference ${REF}
```

4.3.3 Building a comprehensive repeat library for use in Maker2

4.3.3.1 *De novo* repeat libraries with RepeatModeler

Building a species-specific repeat library with RepeatModeler (Smit and Hubley 2008):
Repeat libraries were built on both the final Canu and Falcon scaffolds independently using RepeatModeler-open-1.0.11. The libraries were then combined. For each, the following commands were used.

```
BuildDatabase -name ${DBPRE} -engine ncbi ${ASM}

RepeatModeler -engine ncbi -pa 24 -database ${DBPRE} 2>&1 | tee
repeatmodeler.log
```

The resulting RepeatModeler libraries from both assemblies were then combined. Canu contained 2695 repeat families (2,267 Unknown), and Falcon contained 2,661 repeat families (2254 Unknown).

4.3.3.2 Known *Sciara* repeats

Adding previously known repeat sequences from Bradysia (*Sciara*) coprophila:
To ensure the completeness of the species-specific repeat library, the following 27 known repeat sequences for Bradysia coprophila (including centromeric, rDNA, tandem repeat, and retrotransposon sequences) were added:

gi|160853|gb|L00951.1|SCIRTRANSB Sciara coprophila retrotransposable element R2 reverse transcriptase gene, 3' end
gi|160851|gb|L00945.1|SCIRTRANSA Sciara coprophila retrotransposable element R1 reverse transcriptase gene, 3' end
gi|332655545|gb|HQ883513.1| Bradysia coprophila Sccr15 centromeric repeat
gi|332655542|gb|HQ883512.1| Bradysia coprophila Sccr14 centromeric repeat
gi|332655541|gb|HQ883511.1| Bradysia coprophila Sccr13 centromeric repeat
gi|332655540|gb|HQ883510.1| Bradysia coprophila Sccr12 centromeric repeat
gi|332655537|gb|HQ883509.1| Bradysia coprophila Sccr11 centromeric repeat
gi|332655536|gb|HQ883508.1| Bradysia coprophila Sccr10 centromeric repeat
gi|332655532|gb|HQ883507.1| Bradysia coprophila Sccr09 centromeric repeat
gi|332655531|gb|HQ883506.1| Bradysia coprophila Sccr08 centromeric repeat
gi|332655530|gb|HQ883505.1| Bradysia coprophila Sccr07 centromeric repeat
gi|332655526|gb|HQ883504.1| Bradysia coprophila Sccr06 centromeric repeat
gi|332655525|gb|HQ883503.1| Bradysia coprophila Sccr05 centromeric repeat
gi|332655524|gb|HQ883502.1| Bradysia coprophila Sccr04 centromeric repeat
gi|332655520|gb|HQ883501.1| Bradysia coprophila Sccr03 centromeric repeat
gi|332655519|gb|HQ883500.1| Bradysia coprophila Sccr02 centromeric repeat
gi|332655518|gb|HQ883499.1| Bradysia coprophila Sccr01 centromeric repeat
gi|332655517|gb|HQ883498.1| Bradysia coprophila X.01F4 non-LTR retrotransposon ScRTE, partial sequence
gi|332655514|gb|HQ883497.1| Bradysia coprophila X.01F7 tandem repeat
gi|332655513|gb|HQ883496.1| Bradysia coprophila X.01B4 tandem repeat
gi|332655512|gb|HQ883495.1| Bradysia coprophila X.01G2 centromeric repeat
gi|332655506|gb|HQ883494.1| Bradysia coprophila clone lambda DASH II phage clone 8.4.3.2 non-LTR retrotransposon ScRTE, complete sequence
gi|10115|emb|V01340.1| Sciara coprophila genes for 5.8S and 2S ribosomal RNAs
gi|10114|emb|X02482.1| Sciara coprophila 28S-alpha/beta rDNA gap region
gi|2190334|emb|X93402.1| B.coprophila 28S rRNA gene, expansion segment D7
gi|2190333|emb|X93385.1| B.coprophila 28S rRNA gene, expansion segment D1
gi|2190332|emb|X93391.1| B.coprophila 28S rRNA gene, expansion segment D3-5

4.3.3.3 Known Arthropod Repeats

Adding the arthropoda library from the RepeatMasker (Smit et al. 2013) Combined Database: Dfam_Consensus-20181026 (Hubley et al. 2016) and RepBase-20181026 (Bao et al. 2015)

In an early Maker trial run, despite specifying that Maker use “arthropoda” as the “species” argument to RepeatMasker, we noticed that only “Simple” and “Low Complexity” repeats were being identified and masked. There were no arthropoda repeats identified in the assembly although we could identify many repeats from the arthopoda library generated by RepeatMasker (RepeatMasker/Libraries/dc20181026-rb20181026/arthropoda/specieslib) in the assemblies using Minimap2. We tried running RepeatMasker directly and were able to reproduce the problem.

```
RepeatMasker ${ASM} -species arthropoda -dir soft -pa 1 –xsmall
```

When inspecting the arthropoda library at RepeatMasker/Libraries/dc20181026-rb20181026/arthropoda/specieslib, we noticed the sequences were entirely lower-case. We found that converting the sequences to upper-case resolved the issue completely. Therefore, we converted the arthropoda library to uppercase and added it to our species-specific library described above. When we ran Maker (described later), we provided this comprehensive combined species-specific and arthropoda library instead of specifying a species. We also generated soft-masked versions of the final Canu and Falcon scaffolds:

```
RMLIB=arthropoda_and_species-specific-repeat-library.fasta

RepeatMasker ${ASM} -lib ${RMLIB} -dir ./masked -xsmall -pa
${SLURM_NTASKS} -gff -html -source
```

4.3.4 Maker2 gene annotation

4.3.4.1 Overview:

Maker2 (Holt and Yandell 2011) was used for protein-coding gene annotation of the two final genome assembly candidates. The gene set for a given assembly was produced using gene predictors trained only on the given assembly. In all cases, training was performed using only contigs labeled as Arthropoda in the BlobTools analysis. GeneMark-ES (Ter-Hovhannisyan et al. 2008) was trained on soft-masked contigs. Augustus (Hoff and Stanke 2019) was trained using BUSCO v3 using the 2799 genes in the Dipteran lineage from OrthoDB v9. SNAP (Korf 2004; Campbell et al. 2014) was first trained using gene models output from the first round of Maker2 that used only transcript and protein evidence (est2genome=1, protein2genome=1), and then re-trained on the output of the second Maker round that included gene predictors as described below.

There were essentially three Maker2 rounds. Transcript evidence was provided to Maker2 as the Trinity de novo transcriptome and StringTie transcriptome constructed on the given genome assembly being annotated. Alternative transcript evidence was provided from fly species spanning the Nematocera and Brachycera suborders of Diptera, including the transcriptomes from *Drosophila melanogaster* and *Anopheles gambiae*. Protein homology evidence was provided using all Swiss-Prot proteins from Arthropoda (The UniProt Consortium 2019), as well as from proteomes spanning the holometaboulous insects, including *Drosophila melanogaster* (fruit fly), *Anopheles gambiae* (mosquito), *Apis mellifera* (honey bee), *Bombyx mori* (silkmoth), and *Tribolium castaneum* (red flour beetle). Maker2 was first run to create gene models from the transcript evidence alone to use as an initial training set for gene prediction with SNAP. For the second round of Maker2, gene models were created from the three gene predictors (SNAP, Augustus, GeneMark-ES) using the RNA-seq and homology evidence. SNAP was re-trained using the output of the second round, and a third round of Maker was run. In the third round, Maker2 was instructed to output all models and predictions (keep_preds=1). InterProScan (Quevillon et al. 2005) was subsequently run on all of the protein models to annotate them with protein domain information. Models were kept if they had an Annotation Edit Distance (AED) between 0 and 1 (inclusive) and/or had a recognizable protein domain as recommended (Campbell et al. 2014).

To rescue gene models buried inside masked repetitive regions, we also re-ran the third round of Maker2 exactly as above, but using a repeat library that was filtered to remove repeat models that contained genes, such as Odorant Receptor or Histone genes. We compared the Maker2 transcriptome outputs from using the original repeat library and the filtered repeat library by looking at their AED distributions, and using BUSCO, RSEM-Eval, and TransRate (see Supplemental Table S15A-B). For both Canu and Falcon, the gene sets produced using the original repeat library were higher quality as they had more genes, better AED distributions, more complete (e.g. more BUSCOs), and better evaluations. Therefore, we chose to use the transcript models generated using the original repeat library (stringent masking) as the primary set of annotations, and included transcript models generated when using the filtered repeat library (less masking) only if those gene structures had no overlaps with the primary set (using BEDtools intersect). This resulted in adding 1132 and 607 gene models to the primary Canu and Falcon gene model sets, respectively. We found that of these additional genes added back to the Canu set, for example, 30 were labeled as “Core histone H2A/H2B/H3/H4” and 11 were labeled as “7tm Odorant receptor”. Thus, we were able to rescue many genes originally modeled as repeats. Moreover, this resulted in an increased number of BUSCOs detected as well as higher scores in other metrics such as the RSEM-Eval Score.

The combined gene sets were then finalized for both the Canu and Falcon assemblies by removing gene models corresponding to contaminating (bacterial) contigs to give the final sets specific to *Sciara coprophila* (*Bradysia coprophila*). Gene models were named Bcop_v1_gXXXXXX and isoforms tagged with -RA/RB/RC/etc. Proteins were functionally

annotated, where possible, by using InterProScan (Quevillon et al. 2005) for protein domain information and BLASTp against the entire UniProt/SwissProt protein database (Consortium 2019). The steps described here are detailed further below.

4.3.4.2 Training GeneMark-ES for the first gene prediction round of Maker

GeneMark-ES (Ter-Hovhannisyan et al. 2008) was trained separately on the final Canu and Falcon scaffolds. For both, the scaffolds labeled as Arthropod in the BlobTools analysis were extracted from the soft-masked assemblies (soft-masked using the arthropoda and species-specific library as described above). These sequences made up over 96.6% of each assembly and the majority was also labeled as Dipteran (>98%). This was done to avoid training on any bacterial or any other contaminating sequences present. GeneMark-ES was trained on the soft-masked arthropod-labeled scaffolds with the following command:

```
gmes_petap.pl --ES --max_contig 50000000 --cores 9 --max_intron 500000
--sequence ${ASM} --soft_mask 5000
```

The output file produced located under the working directory at "./output/gmhmm.mod" was used as the GeneMark HMM in all Maker runs involving gene prediction (see below).

4.3.4.3 Using BUSCO to train Augustus for the first gene prediction round of Maker

Augustus (Hoff and Stanke 2019) was trained separately on the final Canu and Falcon scaffolds using BUSCO (Simão et al. 2015). For both, we used BUSCOv3 with the Dipteran lineage from ODB9, specifying the "--long" option, and starting with "fly" as the species (for Augustus). "Canu" is used in the examples below – the same was done for Falcon.

```
run_BUSCO.py --in ${ASM} -o Sciara_canu -l ${DIPTERA} -m genome --cpu 9
--limit 10 --long -sp fly -z --augustus_parameters="--progress=true"
```

The optimized re-training parameters were then copied into the species directory for Augustus in subdirectory named "Sciara_long_unmasked_rl10_canu":

```
cp run_Sciara_canu/augustus_output/retraining_parameters/*
/Path/to/augustus-3.2.2/config/species/Sciara_long_unmasked_rl10_canu/
```

The files therein were then re-named such that their prefixes matched the directory name (Sciara_long_unmasked_rl10_canu) instead of the prefixes given by BUSCO. Finally, the innards of the "*_parameters.cfg*" files that pointed to those files needed to be changed from the BUSCO prefix to the new prefix as well.

The name "Sciara_long_unmasked_rl10_canu" was provided as the Augustus species in the first round of gene prediction in Maker (see below).

4.3.4.4 Using Maker est2genome/protein2genome to train SNAP for the first gene prediction round of Maker

SNAP (Korf 2004) was trained separately on the final Canu and Falcon scaffolds starting with evidence-only gene models from Maker2 (Holt and Yandell 2011; Campbell et al. 2014). Maker2 is an annotation tool that can be run iteratively (with training and re-training of gene predictors) to improve annotations. In the first round of Maker (described in more detail in the Maker section below), we did not use any gene predictors, instead generating gene models from EST and protein evidence alone. We used our Trinity and StringTie assemblies for EST evidence and a comprehensive set of arthropod/insect proteins (detailed in the Maker section) as protein homology evidence. Maker gene models were then extracted from only scaffolds labeled as Arthropod/Diptera and used to train SNAP in the following way:

In a directory outside of the directory Maker was launched in:

```
ln -s /Path/to/*maker.output/*datastore .
grep -w -f arthropod_scaffs.txt
/Path/to/*.maker.output/*_master_datastore_index.log > arthropod.log
gff3_merge -o Sciara_rnd1.arthropod.gff -d arthropod.log

maker2zff Sciara_rnd1.arthropod.gff
fathom genome.ann genome.dna -gene-stats > gene-stats.log 2>&1
fathom genome.ann genome.dna -validate > validate.log 2>&1
fathom -categorize 1000 genome.ann genome.dna > categorize.log 2>&1
fathom -export 1000 -plus uni.ann uni.dna > uni-plus.log 2>&1
mkdir pars
cd pars
forge ../export.ann ../export.dna > ../forge.log 2>&1
cd ..
hmm-assembler.pl Sciara_rnd1 pars > Sciara_rnd1.hmm
```

The file “Sciara_rnd1.hmm” was provided to Maker for the first round of gene prediction (second Maker round).

4.3.4.5 Repeat Libraries

Repeat masking inside Maker was performed with the combined set of repeats from (i) all Arthropoda in Dfam/RepBase (Bao et al. 2015; Hubley et al. 2016), (ii) the species-specific repeat library generated from RepeatModeler, and (iii) known repeat sequences. The “te_proteins.fasta” that comes with Maker2 was used as the set of repeat proteins for repeat masking.

4.3.4.6 EST evidence

Both the Trinity and StringTie assemblies constructed from both sexes across multiple life stages were passed as EST evidence, Trinity as a fasta and StringTie as a GFF.

4.3.4.7 Alternative EST evidence

The following 68,299 Dipteran transcript sequences were passed to Maker as alternative EST evidence.

- Nematocera suborder of Diptera
    - Bibionomorpha infraorder
        - Known *Bradysia coprophila* mRNA sequences from NCBI nucleotide database (txid38358; downloaded on Sep 01, 2016)
            - There were 14 mRNA sequences
        - All 22,152 mRNA sequences under Bibionomorpha from NCBI nucleotide/EST database (txid43784, downloaded on Dec 15, 2018)

- Cecidomyiidae family (13,582 sequences; 5 species)
  - 449 Mayetiola avenae (Oat midge)
  - 458 Mayetiola hordei (Barley midge)
  - 1546 Sitodiplosis mosellana (Wheat midge)
  - 9866 Mayetiola destructor (Hessian fly)
  - 1263 Orseolia oryzae (Asian rice gall midge)
- Sciaridae family
  - 8570 Rhynchosciara Americana

- Culicomorpha infraorder
  - *Anopheles gambiae* transcriptome (downloaded May 4, 2017):
    - https://www.vectorbase.org/download/anopheles-gambiae-pesttranscriptsagamp46fagz
    - 15,648 sequences

- Brachycera suborder of Diptera
  - *Drosophila melanogaster* transcriptome (Downloaded Jan 20, 2015):
    - ftp://ftp.flybase.net/genomes/Drosophila_melanogaster/current/fasta/dmel-all-transcript-r6.03.fasta.gz
    - 30,485 sequences

4.3.4.8 Protein homology evidence

The following protein sequences from the Arthropoda phylum were passed to Maker as protein homology evidence:

UniProt Arthropoda:
All protein sequences available for Arthropoda (the phylum in which the class Insecta resides) from UniProt (12,408 fasta entries) were passed to maker for protein homology evidence.

- https://www.uniprot.org/uniprot/?query=reviewed:yes%20taxonomy:6656
- This spanned 843 species from 463 genera with a median of 2 sequences per species and 3 per genus. There were 3541 sequences from Drosophila melanogaster, an order of magnitude higher than that from the next highest (Drosophila pseudoobscura). The UniProt Arthropoda dataset contained at most 7812 differently-named protein sequences, about half of which must have had an entry from Drosophila.

All other protein evidence came from representatives from four major orders of the Insecta class.

- Diptera order
  - Nematocera suborder
    - Bibionomorpha infraorder
      - Known *Bradysia coprophila* mRNA sequences from NCBI nucleotide database (txid38358; downloaded on Sep 01, 2016)
        - There were 16 protein sequences
      - Rhynchosciara proteins
        - 20
    - Culicomorpha infraorder
      - *Anopheles gambiae* proteome (downloaded May 4, 2017):
        - https://www.vectorbase.org/download/anopheles-gambiae-pestpeptidesagamp46fagz
        - 14,909 sequences
  - Brachycera suborder
    - *Drosophila melanogaster* proteome (Downloaded Jan 20, 2015):
      - ftp://ftp.flybase.net/genomes/Drosophila_melanogaster/current/fasta/dmel-all-translation-r6.03.fasta.gz
      - 30,485 sequences

    - Drosophila represents a selection from the Culicomorpha infraorder under the Nematocera suborder of the order Diptera

- Hymenoptera order
    - *Apis mellifera* protein sequences
        - https://www.ncbi.nlm.nih.gov/protein; txid7460[Organism:exp]; RefSeq entries only; downloaded Dec 15, 2018
        - 23,491 protein sequences
- Lepidoptera order
    - *Bombyx mori* protein sequences
        - https://www.ncbi.nlm.nih.gov/protein; txid7091[Organism:noexp]; RefSeq entries only; downloaded Dec 15, 2018
        - 22,590 protein sequences
- Coleoptera order
    - *Tribolium castaneum* proteins
        - https://www.ncbi.nlm.nih.gov/protein?LinkName=bioproject_protein&from_uid=15718; clicking RefSeq; RefSeq entries only; downloaded Dec 15, 2018
        - 22,597 protein sequences

4.3.4.9 Other parameter choices

- The maximum DNA length was set to 2 Mb so contigs and scaffolds would mostly not be split up.
- The minimum contig size was kept as default (1 bp).
- Maker2 was instructed to look for alternative splicing transcripts.
- Our choices for the minimum exon size (200 bp) and maximum intron size (500 kb) that Maker2 was instructed to expect were guided by the minimum exon and maximum intron sizes found in the StringTie transcriptome assembly.
- Similarly, Maker2 was instructed to use single exon ESTs as evidence given the that a fair number of transcripts appeared to be single exon (~3500; ~10%) from the StringTie assemblies.
- est2genome and protein2genome were both turned on in the first round of Maker2 to generate gene models strictly from EST and protein homology evidence for training SNAP prior to the second round of Maker2.

4.3.4.10 Maker2 Round 1

The maker_opts.ctl file for Maker Round 1:

```
#-----Genome (these are always required)
genome=/path/to/asm.fasta #genome sequence (fasta file or fasta embeded
in GFF3 file)
organism_type=eukaryotic #eukaryotic or prokaryotic. Default is
eukaryotic

#-----Re-annotation Using MAKER Derived GFF3
maker_gff= #MAKER derived GFF3 file
est_pass=0 #use ESTs in maker_gff: 1 = yes, 0 = no
altest_pass=0 #use alternate organism ESTs in maker_gff: 1 = yes, 0 = no
protein_pass=0 #use protein alignments in maker_gff: 1 = yes, 0 = no
rm_pass=0 #use repeats in maker_gff: 1 = yes, 0 = no
model_pass=0 #use gene models in maker_gff: 1 = yes, 0 = no
pred_pass=0 #use ab-initio predictions in maker_gff: 1 = yes, 0 = no
other_pass=0 #passthrough anyything else in maker_gff: 1 = yes, 0 = no

#-----EST Evidence (for best results provide a file for at least one)
```

```
est=/Path/To/Trinity.fasta #set of ESTs or assembled mRNA-seq in fasta
format
altest=/Path/To/combined_transcript_sequences.fasta #EST/cDNA sequence
file in fasta format from an alternate organism
est_gff=/Path/To/stringtie_merged_Sciara_general_transcriptome.gff
#aligned ESTs or mRNA-seq from an external GFF3 file
altest_gff= #aligned ESTs from a closly relate species in GFF3 format

#-----Protein Homology Evidence (for best results provide a file for at
least one)
protein=/Path/To/combined_protein_sequences.fasta  #protein sequence file
in fasta format (i.e. from mutiple oransisms)
protein_gff=  #aligned protein homology evidence from an external GFF3
file

#-----Repeat Masking (leave values blank to skip repeat masking)
model_org= #select a model organism for RepBase masking in RepeatMasker
rmlib=/Path/to/combined_arthopoda_species-specific-library.fa #provide an
organism specific repeat library in fasta format for RepeatMasker
repeat_protein=/Path/To/maker/data/te_proteins.fasta #provide a fasta
file of transposable element proteins for RepeatRunner
rm_gff= #pre-identified repeat elements from an external GFF3 file
prok_rm=0 #forces MAKER to repeatmask prokaryotes (no reason to change
this), 1 = yes, 0 = no
softmask=1 #use soft-masking rather than hard-masking in BLAST (i.e. seg
and dust filtering)

#-----Gene Prediction
snaphmm= #SNAP HMM file
gmhmm= #GeneMark HMM file
augustus_species= #Augustus gene prediction species model
fgenesh_par_file= #FGENESH parameter file
pred_gff= #ab-initio predictions from an external GFF3 file
model_gff= #annotated gene models from an external GFF3 file (annotation
pass-through)
est2genome=1 #infer gene predictions directly from ESTs, 1 = yes, 0 = no
protein2genome=1 #infer predictions from protein homology, 1 = yes, 0 =
no
trna=0 #find tRNAs with tRNAscan, 1 = yes, 0 = no
snoscan_rrna= #rRNA file to have Snoscan find snoRNAs
unmask=0 #also run ab-initio prediction programs on unmasked sequence, 1
= yes, 0 = no

#-----Other Annotation Feature Types (features MAKER doesn't recognize)
other_gff= #extra features to pass-through to final MAKER generated GFF3
file

#-----External Application Behavior Options
alt_peptide=C #amino acid used to replace non-standard amino acids in
BLAST databases
cpus=1 #max number of cpus to use in BLAST and RepeatMasker (not for MPI,
leave 1 when using MPI)

#-----MAKER Behavior Options
max_dna_len=100000000 #100mil to notdivide up -- length for dividing up
contigs into chunks (increases/decreases memory usage)
min_contig=1 #skip genome contigs below this length (under 10kb are often
useless)
pred_flank=200 #flank for extending evidence clusters sent to gene
predictors
pred_stats=0 #report AED and QI statistics for all predictions as well as
```

```
models
AED_threshold=1 #Maximum Annotation Edit Distance allowed (bound by 0 and
1)
min_protein=0 #require at least this many amino acids in predicted
proteins
alt_splice=1 #Take extra steps to try and find alternative splicing, 1 =
yes, 0 = no
always_complete=0 #extra steps to force start and stop codons, 1 = yes, 0
= no
map_forward=0 #map names and attributes forward from old GFF3 genes, 1 =
yes, 0 = no
keep_preds=0 #Concordance threshold to add unsupported gene prediction
(bound by 0 and 1)
split_hit=500000 #length for the splitting of hits (expected max intron
size for evidence alignments)
single_exon=1 #consider single exon EST evidence when generating
annotations, 1 = yes, 0 = no
single_length=200 #min length required for single exon ESTs if
'single_exon is enabled'
correct_est_fusion=0 #limits use of ESTs in annotation to avoid fusion
genes
tries=4 #number of times to try a contig if there is a failure for some
reason
clean_try=0 #remove all data from previous run before retrying, 1 = yes,
0 = no
clean_up=0 #removes theVoid directory with individual analysis files, 1 =
yes, 0 = no
TMP= #specify a directory other than the system default temporary
directory for temporary files
```

Obtaining outputs from Maker Round 1:

```
fasta_merge -o Sciara_rnd${N} -d
../../*.maker.output/*_master_datastore_index.log

gff3_merge -o Sciara_rnd${N}.all.gff -d
../../*.maker.output/*_master_datastore_index.log

gff3_merge -n -g -o Sciara_rnd${N}.makermodels.gff -d
../../*.maker.output/*_master_datastore_index.log
```

Running subsequent rounds of Maker2 with gene predictors:

As mentioned above, SNAP was trained using the output of Maker round1; Augustus was trained with BUSCO; and GeneMark-ES was self-trained. As we ran this annotation pipeline on the two final assembly candidates, all training was specific to the assembly being annotated. Thus, the Canu assembly annotations were trained on the Canu assembly, and the Falcon assembly annotations were trained on the Falcon assembly.

4.3.4.11 Maker2 Round 2

The maker_opts.ctl file for Round 2 was the same as Round 1 except for the following:

```
#-----Gene Prediction
snaphmm=/Path/to/Sciara_rnd1.hmm #SNAP HMM file
gmhmm=/Path/to/gmhmm.mod #GeneMark HMM file
augustus_species=busco_trained_Sciara #Augustus gene prediction species
model
est2genome=0 #infer gene predictions directly from ESTs, 1 = yes, 0 = no
```

```
protein2genome=0 #infer predictions from protein homology, 1 = yes, 0 =
no
```

Outputs were obtained as in round 1.

To be confident the BUSCO-trained Augustus model was most appropriate, we also compared AED distributions when specifying:

```
augustus_species=fly
```

and:

```
augustus_species=aedes
```

The overall AED distributions were similar with a slight advantage to using the BUSCO-trained *Sciara* model. However, when looking at the AED distributions of only Augustus predicted genes, the *Sciara* model definitively out-performed the "fly" and "aedes" models. The BUSCO-trained *Sciara* model was used going forward.

4.3.4.12 Maker2 Round 3 (keep_preds=1):

SNAP was trained one more time with the output from Maker Round 2 to create a new SNAP HMM for Maker Round 3 (Sciara_rnd2.hmm). Thus, the maker_opts.ctl file for Maker Round 3 was the same as for Maker Round 2, except:

```
#-----Gene Prediction
snaphmm=/Path/to/Sciara_rnd2.hmm #SNAP HMM file
```

In previous rounds, we had keep_preds=0. However, some predictions that are not supported by available RNA-seq and homology evidence may have recognizable protein domains. Thus, Maker can be run such that predictions are retained, and gene sets are constructed afterward with a post-hoc script that filtered based on AED and/or containment of protein domains (Campbell et al. 2014). The initial output containing everything is the "max" set. Filtering for only genes that are supported by the evidence within the Maker run (AED in 0 to 1) gives the default set. The standard set is intermediate and contains the default set plus any additional genes from the max set with recognizable protein domains. To take advantage of assessing the three different levels of gene sets from Maker (default, standard, and maximum: (Campbell et al. 2014), Maker Round 3 was re-run with:

```
#-----MAKER Behavior Options
keep_preds=1
```

Outputs were obtained as in round 1 and with these additions:

```
# InterProScan was run on all Maker Proteins
PROT=Sciara_rnd3.all.maker.proteins.fasta
interproscan.sh -dp -cpu ${SLURM_NTASKS} \
      -appl PfamA -iprlookup -goterms -f tsv -i ${PROT}

GFF=Sciara_rnd3.all.gff
TSV=${PROT}.tsv
OUT=Sciara_rnd3.max.functional_ipr.gff

# Update GFF to new GFF
ipr_update_gff ${GFF} ${TSV} > ${OUT}

## Make default build (should be same as rnd3 run)
quality_filter.pl -d ${OUT} > Sciara_rnd3.default.functional_ipr.gff

## Make standard build
quality_filter.pl -s ${OUT} > Sciara_rnd3.standard.functional_ipr.gff
```

```
## Count number of genes between both files to find out how many were
rescued
DEF=$( awk '$3=="gene"' Sciara_rnd3.default.functional_ipr.gff | wc -l
)
STD=$( awk '$3=="gene"' Sciara_rnd3.standard.functional_ipr.gff | wc -l
)
DIFF=$( echo $STD - $DEF | bc )
H="Default\tStandard\tDifference\n${DEF}\t${STD}\t${DIFF}"
echo -e ${H} > def-std-diff.txt

## Get names of transcripts in default and standard sets
awk '$3=="mRNA" {print $9}' Sciara_rnd3.default.functional_ipr.gff |
awk '{gsub(/=|;/,"\t"); print $2}' | sort | uniq > default-
transcripts.txt
awk '$3=="mRNA" {print $9}' Sciara_rnd3.standard.functional_ipr.gff |
awk '{gsub(/=|;/,"\t"); print $2}' | sort | uniq > standard-
transcripts.txt

# Get number of rescued transcripts
python -c "default=set([e.strip() for e in open('default-
transcripts.txt').readlines()]); std=set([e.strip() for e in
open('standard-transcripts.txt').readlines()]); ans =
std.difference(default); print( len(ans) )"

# Get names of rescues
python -c "default=set([e.strip() for e in open('default-
transcripts.txt').readlines()]); std=set([e.strip() for e in
open('standard-transcripts.txt').readlines()]); ans =
std.difference(default); print( '\n'.join(list(ans)) )" > rescues.txt

# Get transcripts and proteins from default and standard sets
extractFastxEntries.py -n standard-transcripts.txt -f
Sciara_rnd3.all.maker.transcripts.fasta > standard-transcripts.fasta
extractFastxEntries.py -n default-transcripts.txt -f
Sciara_rnd3.all.maker.transcripts.fasta > default-transcripts.fasta
extractFastxEntries.py -n standard-transcripts.txt -f
Sciara_rnd3.all.maker.proteins.fasta > standard-proteins.fasta
extractFastxEntries.py -n default-transcripts.txt -f
Sciara_rnd3.all.maker.proteins.fasta > default-proteins.fasta

# Get rescue transcripts
extractFastxEntries.py -n rescues.txt -f
Sciara_rnd3.all.maker.transcripts.fasta > rescued-transcripts-for-
analysis.fasta

# Get functions of rescues
grep -f rescues.txt Sciara_rnd3.all.maker.proteins.fasta.tsv  > rescue-
functions.txt
```

4.3.4.13 Maker2 Round 3 with gene-filtered repeat library (keep_preds=1)

We noticed that the histone genes, odorant receptors, and other genes were part of the Repeat Modeler species-specific libraries, and were potentially under-represented in our gene annotation set after the original "Round 3". Therefore, we set out to re-run Maker Round 3 using a filtered repeat library where repeat models with matches to protein genes were excluded (unless they were transposon related).

To construct the filtered set of repeats, we used BLASTX to compare the protein database we constructed for Maker (described above) against the two Repeat Modeler libraries that we

created separately on the final Canu and Falcon assemblies (-culling_limit 5 -qcov_hsp_perc 1 -evalue 1e-5). We then manually went through the results to determine whether repeats with protein hits were related to transposons/retrotransposons or not. Transposon hits were kept in the repeat libraries. This effort was aided using NCBI's online blast suite, the full nucleotide database, non-redundant protein database, and RNA refseq database. After splitting the repeat libraries into "repeats for masking" and "contains genes", we used BLAST iteratively to identify repeats in the libraries that did not have initial BLASTX hits, but that were highly similar to repeats regarded as "contains genes" (at least 50% of the query or subject was overlapped). We used BLASTX/BLASTN against the nucleotide and RNA RefSeq databases to ensure that the majority that had hits, had hits to genes, not transposons/retrotransposons. These filtered Repeat Modeler libraries were then combined with Arthropoda in Dfam/RepBase and known repeat sequences as done above, and subsequently used in Maker2 for less aggressive repeat masking.

InterProScan annotation and all outputs of this "alternative round 3" from Maker were obtained as in the section above, "Maker Round 3 keep_preds=1".

4.3.4.14 Maker2 Round 3 combined standard gene sets and finalization

We evaluated the various Maker Round 3 outputs with AED distributions, BUSCO, RSEM-Eval, and TransRate as described elsewhere. The gene sets produced using the original Repeat Library had more genes, better AED distributions, more BUSCOs, and better evaluations otherwise. Therefore, we used only gene annotations generated when using the filtered repeat library only if they had no overlaps with the set of annotations generated when using the original repeat library (using BEDtools). This resulted in adding 1132 and 607 gene models to the original Canu and Falcon gene model sets, respectively. We found that of these additional genes added back to the Canu set, for example, 30 were labeled as "Core histone H2A/H2B/H3/H4" and 11 were labeled as "7tm Odorant receptor". Thus, this increases the representation of those gene families in our final set as we set out to do in the first place as described above.

The combined gene sets were then finalized by removing gene models corresponding to contaminating (bacterial) contigs to give the final set specific to *Sciara* coprophila (Bradysia coprophila). The Maker2 gene names were changed to have a consistent naming scheme (e.g. for Canu all were given the prefix: Bcop_v1_g for Bradysia coprophila, version 1, gene number). In addition to InterProScan and Gene Ontology information obtained previously, proteins were functionally annotated, where possible, by using BLASTp against the entire UniProt/SwissProt protein database. For every *Sciara* protein, we kept only the BLASTp hit with the highest bitscore. Best hits were only retained if they had a bitscore >= 50 or an expected value <= 5e-4. The GFF and Fasta files were updated with the BLASTp hit and all other functional information obtained. For further evaluation of the Maker gene sets for the Canu and Falcon assemblies, we also found the number of genes with BLASTp hits (performed as above) in the *Drosophila melanogaster* and *Anopheles gambiae* proteomes. Gene statistics (on isoforms, exons, introns, etc) were obtained from both the final Maker2 gene model sets as well as the input StringTie transcriptome assemblies using a custom script.

Commands used to find and combine non-overlapping gene models:

```
## Note: find the following at: https://github.com/JohnUrban/sciara-
project-tools
##    parseFamiliesfromGFF.py
##    fasta_name_changer.py
##  Other scripts:
##    gff3sort.pl (https://github.com/billzt/gff3sort; Zhu et al, 2017)
##    BEDtools (Quinlan and Hall, 2010)
```

```
# VARIABLES
PRE=sciara_rnd3
ORIGDIR=../rnd3
ALTDIR=../../with_filtered_repeats/outputs_keeppred1/rnd3
ORIGGFF_PRECLEAN=${ORIGDIR}/${PRE}.standard.functional_ipr.gff
ORIGGFF=cleaned-original-${PRE}.standard.functional_ipr.gff
ORIGTRANS=${ORIGDIR}/standard-transcripts.fasta
ORIGPROT=${ORIGDIR}/standard-proteins.fasta
ORIGIPS=${ORIGDIR}/sciara_rnd3.all.maker.proteins.fasta.tsv
ALTGFF_PRECLEAN=${ALTDIR}/${PRE}.standard.functional_ipr.gff
ALTGFF=cleaned-alt-${PRE}.standard.functional_ipr.gff
ALTTRANS=${ALTDIR}/standard-transcripts.fasta
ALTPROT=${ALTDIR}/standard-proteins.fasta
NONGFF=${PRE}.nonoverlapping-genes.gff
OUTGFF=${PRE}.nonoverlapping-families.gff
OUTTRANS=${PRE}.nonoverlapping-families.transcripts.fasta
OUTPROT=${PRE}.nonoverlapping-families.proteins.fasta
OUTGFFMOD=${PRE}.nonoverlapping-families-modifiedNames.gff
OUTTRANSMOD=${PRE}.nonoverlapping-families.transcripts-
modifiedNames.fasta
OUTPROTMOD=${PRE}.nonoverlapping-families.proteins-modifiedNames.fasta
COMBGFF=${PRE}.combined.standard.gff
COMBTRANS=${PRE}.combined.standard.transcripts.fasta
COMBPROT=${PRE}.combined.standard.proteins.fasta
COMBIPS=${PRE}.interproscan.results.tsv

#0. Clean up Parent attribute in Maker Standard GFF (it has references
to mRNAs that did not make the cut, which can cause issues for some
programs) - cleanParentSlotInGFF.py found at
https://github.com/JohnUrban/sciara-project-tools

cleanParentSlotInGFF.py -g ${ORIGGFF_PRECLEAN} --notfound cleaned-
original-notfound.txt > ${ORIGGFF}

cleanParentSlotInGFF.py -g ${ALTGFF_PRECLEAN} --notfound cleaned-alt-
notfound.txt  > ${ALTGFF}



#1. Obtain gene locations from alternative gene set that have no
overlaps with the original set

intersectBed -v -a <(awk '$3=="gene"' ${ALTGFF} ) -b <( awk
'$3=="gene"' ${ORIGGFF} ) > ${NONGFF}


#2. Create a text file with just the "parents" (gene names) of the non-
overlapping add-ons from alternative gene set.

awk '$3=="gene" {print $9}' ${NONGFF} | awk '{gsub(/=|;/,"\t"); print
$2}' | sort | uniq > parents.txt


#3. Take all GFF entries that correspond to the given parents from
alternative GFF

parseFamiliesfromGFF.py -i ${ALTGFF} -p parents.txt > ${OUTGFF}


#4. Obtain all non-overlapping transcript names, and extract the
transcript and protein sequences corresponding to them
```

```
awk '$3=="mRNA" {print $9}' ${OUTGFF} | awk '{gsub(/=|;/,"\t"); print
$2}' | sort | uniq > transcripts.txt

extractFastxEntries.py -n transcripts.txt -f ${ALTTRANS} > ${OUTTRANS}

extractFastxEntries.py -n transcripts.txt -f ${ALTPROT} > ${OUTPROT}


#5. Modify the names of the genes/transcripts/proteins so they do not
collide with names in the original gene set

parseFamiliesfromGFF.py -i ${OUTGFF} -p parents.txt --namechanger
addons_ > ${OUTGFFMOD}

fasta_name_changer.py -f ${OUTTRANS} -F addons_ > ${OUTTRANSMOD}

fasta_name_changer.py -f ${OUTPROT} -F addons_ > ${OUTPROTMOD}



#6. COMBINE the name changed "Add-ons" with the original gene set and
sort

cat ${ORIGGFF} ${OUTGFFMOD} > tmp.gff

gff3sort.pl --precise --chr_order natural tmp.gff > ${COMBGFF}


#7. COMBINE the name changed "Add-on" transcripts and proteins with
original sets

cat ${ORIGTRANS} ${OUTTRANSMOD} > ${COMBTRANS}

cat ${ORIGPROT} ${OUTPROTMOD} > ${COMBPROT}


#8. COMBINE the InterProScan domain/functional information

grep –w -f parents.txt
${ALTDIR}/sciara_rnd3.all.maker.proteins.fasta.tsv  | awk '{print
"addons_"$0}' > parent-functions-modifiedNames.txt

cat ${ORIGIPS} parent-functions-modifiedNames.txt > ${COMBIPS}
```

Commands used to finalize gene sets:

```
## Note: find the following at: https://github.com/JohnUrban/sciara-
project-tools
##    grep.py : This utility was created for simple tasks one might
normally perform with grep. However, we noticed that some grep
commands/options had different behaviors/results when performed on Mac
OS vs Linux. This utility had stable results across platforms for the
tasks below.
##    extractFastxEntries.py : Utility to manipulate FASTA files
##    tableFilter.py : Utility to extract the best BLAST hit for each
gene model
##    pfammer.py : Utility to add PFAM descriptions to GFF/Fasta
```

#1. Make copies of final files into new directory such that in-place name-changing will be fine.

```
mkdir -p newdir && cd newdir
cp ../*combined* .
cp ../sciara_rnd3.interproscan.results.tsv  .
```

#2. Partition Maker GFF into bacterial and nonbacterial (Sciara) given the set of contigs classified as bacterial

```
grep.py -p bacterial.txt -f sciara_rnd3.combined.standard.gff -c 1 -C 1
> bacterial.sciara_rnd3.combined.standard.gff

grep.py -p bacterial.txt -f sciara_rnd3.combined.standard.gff -c 1 -C 1
-v > nonbacterial.sciara_rnd3.combined.standard.gff
```

#3. Identify mRNA names in Maker GFF associated with bacterial contigs

```
awk '$3=="mRNA" {print $9}' bacterial.sciara_rnd3.combined.standard.gff
| awk '{gsub(/;|=/,"\t"); print $2}' > bacterial-mRNA-names.txt
```

#4. Partition InterProScan Results

```
grep.py -p bacterial-mRNA-names.txt -f
sciara_rnd3.interproscan.results.tsv -c 1 -C 1 >
bacterial.sciara_rnd3.interproscan.results.tsv

grep.py -p bacterial-mRNA-names.txt -f
sciara_rnd3.interproscan.results.tsv -c 1 -C 1 -v >
nonbacterial.sciara_rnd3.interproscan.results.tsv
```

#5. Partition Protein Fasta

```
extractFastxEntries.py -n bacterial-mRNA-names.txt -f
sciara_rnd3.combined.standard.proteins.fasta >
bacterial.sciara_rnd3.combined.standard.proteins.fasta

extractFastxEntries.py --exclude -n bacterial-mRNA-names.txt -f
sciara_rnd3.combined.standard.proteins.fasta >
nonbacterial.sciara_rnd3.combined.standard.proteins.fasta
```

#6. Partition Trascript Fasta

```
extractFastxEntries.py -n bacterial-mRNA-names.txt -f
sciara_rnd3.combined.standard.transcripts.fasta >
bacterial.sciara_rnd3.combined.standard.transcripts.fasta

extractFastxEntries.py --exclude -n bacterial-mRNA-names.txt -f
sciara_rnd3.combined.standard.transcripts.fasta >
nonbacterial.sciara_rnd3.combined.standard.transcripts.fasta
```

#7. Change the names to consistent formatting: Bcop version 1 gene number

```
#7a. Make kev:value maps between old names and new names for Sciara
models (nonbacterial)
maker_map_ids --prefix Bcop_v1_g --justify 6
nonbacterial.sciara_rnd3.combined.standard.gff >
nonbacterial.sciara_rnd3.combined.standard.map

#7b. Make kev:value maps between old names and new names for
contaminating/removed models (bacterial)
maker_map_ids --prefix Bcop-bacterial_v1_bg --justify 6
bacterial.sciara_rnd3.combined.standard.gff >
bacterial.sciara_rnd3.combined.standard.map


#7c. Rename in place using Maker-provided utilities
for PRE in bacterial nonbacterial; do
      map_gff_ids ${PRE}.sciara_rnd3.combined.standard.map
      ${PRE}.sciara_rnd3.combined.standard.gff

      map_fasta_ids ${PRE}.sciara_rnd3.combined.standard.map
      ${PRE}.sciara_rnd3.combined.standard.transcripts.fasta

      map_fasta_ids ${PRE}.sciara_rnd3.combined.standard.map
      ${PRE}.sciara_rnd3.combined.standard.proteins.fasta

      map_data_ids ${PRE}.sciara_rnd3.combined.standard.map
      ${PRE}.sciara_rnd3.interproscan.results.tsv

done



#8. Perform BLASTP of proteins against entire UniProt/SwissProt
Database(Optionally also do on contaminating/bacterial gene models);
These commands were also used to look at the Maker proteins compared to
Drosophila and Anopheles proteomes

#8a. Download uniprot_sprot FASTA and make BLAST database
wget
ftp://ftp.uniprot.org/pub/databases/uniprot/current_release/knowledgeba
se/complete/uniprot_sprot.fasta.gz

gunzip uniprot_sprot.fasta.gz

makeblastdb -in uniprot_sprot.fasta -dbtype prot -out uniprot_sprot


#8b. BLASTp – the BLAST command used is given here although we broke
the gene sets up to compute the results in parallel. (Below PRE is
either nonbacterial or bacterial corresponding to files made above)

blastp -query $Q -db $DB -num_threads $P -outfmt 6 -evalue 1e-2 -out
output.blastp

#8c. Take only the best hit for each gene model
tableFilter.py -n 1 -s 12 output.blastp > ${PRE}.filtered-
sciara_rnd3.combined.standard.proteins.all.output.blastp

#8d. Keep hits only if they meet bitscore or e-value thresholds
(bitscore >= 50 or evalue <= 5e-4)
awk '$12>=50 || $11 <= 5e-4' ${PRE}.filtered-
sciara_rnd3.combined.standard.proteins.all.output.blastp >
```

```
${PRE}.final-filtered-
sciara_rnd3.combined.standard.proteins.all.output.blastp



#9. Update GFF and FASTA with BLAST info -- finalize

for PRE in nonbacterial bacterial; do
	## variables
	UNIPROT=uniprot_sprot.fasta
	BLASTP=${PRE}.final-filtered-
	sciara_rnd3.combined.standard.proteins.all.output.blastp
	GFF=${PRE}.sciara_rnd3.combined.standard.gff
	BASE=$( basename ${GFF} .gff )
	PROT=${BASE}.proteins.fasta
	TRANS=${BASE}.transcripts.fasta
	NEWGFF=${BASE}.putative_function.gff
	NEWPROT=${BASE}.proteins.putative_function.fasta
	NEWTRANS=${BASE}.transcripts.putative_function.fasta

	## Annotate Maker GFF with BLASTp information
	maker_functional_gff ${UNIPROT} ${BLASTP} ${GFF} > ${NEWGFF}

	## Annotate Protein Fasta with BLASTp information
	maker_functional_fasta ${UNIPROT} ${BLASTP} ${PROT} > ${NEWPROT}

	## Annotate Transcript Fasta with BLASTp information
	maker_functional_fasta ${UNIPROT} ${BLASTP} ${TRANS} >
	${NEWTRANS}


	## Create GFF annotating genome with InterProScan Domain/GO
	Information
	iprscan2gff3 ${PRE}.sciara_rnd3.interproscan.results.tsv
	${PRE}.sciara_rnd3.combined.standard.putative_function.gff >
	${PRE}.sciara_rnd3.combined.standard.putative_function.visible_ip
	rscan_domains.gff

	## Annotate Protein Fasta with InterProScan Domain/GO/Pfam info
	pfammer.py -p ${PRE}.sciara_rnd3.interproscan.results.tsv -f
	${NEWPROT} > final.${NEWPROT}

	## Annotate Transcript Fasta with InterProScan Domain/GO/Pfam
	info
	pfammer.py -p ${PRE}.sciara_rnd3.interproscan.results.tsv -f
	${NEWTRANS} > final.${NEWTRANS}

	## Annotate Maker GFF with InterProScan Domain/GO/Pfam info
	pfammer.py -p ${PRE}.sciara_rnd3.interproscan.results.tsv -g
	${NEWGFF} > final.${NEWGFF}
done
```

Commands used to get gene statisitcs from Maker and StringTie GFFs:

```
## Note: find the following at: https://github.com/JohnUrban/sciara-
project-tools
## annotationGeneStats.py


annotationGeneStats.py –m /path/to/maker.gff
annotationGeneStats.py –s /path/to/stringtie.gff
```

4.3.4.15 Maker2 gene set evaluations

Maker2 transcriptome evaluations were performed the same as for transcriptome assemblies in "4.3.2 Evaluations of transcriptome assemblies". We also compared the distributions of Annotation Edit Distances (AED) to determine the number of genes with AEDs less than or equal to given cut offs. The Maker2 proteome was also analyzed with BUSCO. We also looked at the Pfam domains, GO terms, and BLASTp results (to UniProt-SwissProt) from Maker2 analyses above in the Maker2 proteomes as well as compared them to known proteomes for *Drosophila melanogaster* and *Anopheles gambiae*.

4.3.5 Final Assembly Selection

There were a total of 66 metrics (reported/described below) weighing in on the final evaluations between Canu and Falcon. Canu outperformed in the majority of individual metrics, winning in 54 of the 66 (81.8%). The scores for individual metrics can be found in Supplemental Figures S13 and S14, and Supplemental Tables S14, S15, and S16. That Canu won the majority of metrics could have been an artifact of simply outperforming in "categories" that had more metrics than categories that Falcon won. To discount this, we partitioned the 66 metrics into 12 categories as organized below, and as represented in Figure 5F. Briefly, categories were organized by dataset used (e.g. Illumina, PacBio, Nanopore, BioNano, RNA-seq, de novo transcriptome), by the parameter being tested (e.g. contig length, gene content), or by the object being tested (e.g. reference-guided transcriptome), and/or by target objective (e.g evaluation of Maker2 genes by external tools, evaluation of the annotation by Maker2 internal metrics, evaluation of annotation according to homology and predicted functions). After partitioning the metrics into categories we determined which assembler won the majority of metrics in each category, then which assembler won the majority of categories. Canu won the majority of metrics in 10 of the 12 categories (83.3%). There are no logical ways to collapse, reorganize, or further split the metrics into sensible categories to overturn this result. For example, since Falcon won just 11 of the 66 metrics, all 11 metrics would need to be split into their own single-metric categories to beat the 10 existing categories won by Canu. However, that categorization would lack obvious logical organizing principles and would be equivalent to arbitrarily multiplying the weight of a subset of the previously-logical categories, with 4 single-metric categories for size statistics and 4 single-metric categories for BioNano optical map metrics, for example. Overall, the Canu assembly was chosen over Falcon based on these analyses. Nonetheless, despite winning the majority of metrics, the differences in scores were often small, suggesting either assembly would be fine as a first draft.

Each metric below is described in previous sections (see subsections within each):

- Section 4.1.3 Evaluations of the Initial 40 Short Read Assemblies
- Section 4.2.5 Evaluations of the Initial 50 long read assemblies
- Section 4.3.2 Evaluations of transcriptome assemblies
- Section 4.3.4.15 Maker2 gene set evaluations
- Also see papers describing (and other documentation associated with):
    - LAP (Ghodsi et al. 2013),
    - ALE (Clark et al. 2013),
    - REAPR (Hunt et al. 2013),
    - FRC^{bam} (Vezzi et al. 2012),
    - BUSCO (Simão et al. 2015),
    - Pilon (Walker et al. 2014),
    - Sniffles (Sedlazeck et al. 2018),
    - TransRate (Smith-Unna et al. 2016),
    - RSEM-Eval (Li et al. 2014), and
    - Maker2 (Holt and Yandell 2011)

<u>Category 1</u>: Size statistics (4) (see Supplemental Figure S13 A-D)

(01) NG50
(02) LG50
(03) Max contig length
(04) Expected contig length (normalized to expected genome size)

<u>Category 2</u>: Gene content in genome assembly sequence (2) (see Supplemental Figure S13 E-F)

(05) Number of Arthropod BUSCOs (v1)
(06) Number of Dipteran BUSCOs (v3)

<u>Category 3</u>: Illumina DNA-seq dataset metrics (8) (see Supplemental Figure S13 I-P)

(07) Percent Illumina DNA-seq reads aligned with Bowtie2
(08) Percent Illumina paired-end DNA-seq reads aligned concordantly
(09) LAP
(10) ALE
(11) REAPR Mean Base Score
(12) REAPR Links
(13) FRC^{bam} – number of features per Mb
(14) Pilon – percent of expected genome size confirmed

Category 4: PacBio metrics (8) (see Supplemental Figure S13 Q-X)
(15) PacBio percent reads aligned with BWA
(16) PacBio average alignment score
(17) PacBio average MAPQ
(18) PacBio percent reads split across multiple contigs
(19) PacBio average number of split alignments per read
(20) PacBio total number SVs reported by Sniffles
(21) PacBio number translocations reported by Sniffles
(22) PacBio number other SVs (del, dup, ins, inv) reported by Sniffles

Category 5: Nanopore metrics (8) (see Supplemental Figure S13 Y-f)
(23) Nanopore percent reads aligned with BWA
(24) Nanopore average alignment score
(25) Nanopore average MAPQ
(26) Nanopore percent reads split across multiple contigs
(27) Nanopore average number of split alignments per read
(28) Nanopore total number SVs reported by Sniffles
(29) Nanopore number translocations reported by Sniffles
(30) Nanopore number other SVs (del, dup, ins, inv) reported by Sniffles

Category 6: BioNano optical map metrics (4) (see Supplemental Figure S13 g-j)
(31) BioNano Span
(32) BioNano average M-score
(33) BioNano average alignment length
(34) BioNano average coverage

Category 7: RNA-seq evaluation of genome assembly (1) (see Supplemental Figure S13 G)
(35) Percent RNA-seq reads aligned with HiSat2 (completeness)

Category 8: De novo transcriptome support for genome assembly (1) (see Supplemental Figure S13 H)
(36) Bitscore sum of de novo transcript BLAST alignments

Category 9: Evaluations of genome-guided transcriptome assemblies (9) (see Supplemental Table S14)
(37) Number of Complete Dipteran BUSCOs (v3) (more is better)
(38) Number of Missing Dipteran BUSCOs (v3) (fewer is better)
(39) TransRate - proportion of reference proteins with Conditional Reciprocal Best BLAST (CRBB) hits given the transcriptome assembly (Reference = UniProt Arthropod Proteins)
(40) TransRate - proportion of reference proteome covered by transcripts from the transcriptome assembly (Reference = UniProt Arthropod Proteins)
(41) TransRate - proportion of reference proteins with CRBB hits given the transcriptome assembly (Reference = Maker2 Input)
(42) TransRate - proportion of reference proteome covered by transcripts from the transcriptome assembly (Reference = Maker2 Input)

(43) TransRate Score
(44) TransRate Optimal Score
(45) RSEM-Eval Score

Category 10: External evaluations of Maker2 gene annotations (11) (see Supplemental Table S15A-B, and Supplemental Figure S14 A, C-E)

(46) Number of Complete Dipteran BUSCOs (v3) found in transcripts
(47) Number of Missing Dipteran BUSCOs (v3) found in transcripts
(48) Number of Complete Dipteran BUSCOs (v3) found in proteins
(49) Number of Missing Dipteran BUSCOs (v3) found in proteins
(50) TransRate - proportion of reference proteins with Conditional Reciprocal Best BLAST (CRBB) hits given the Maker2 annotation (Reference = UniProt Arthropod Proteins)
(51) TransRate - proportion of reference proteome covered by transcripts from the Maker2 annotation (Reference = UniProt Arthropod Proteins)
(52) TransRate - proportion of reference proteins with Conditional Reciprocal Best BLAST (CRBB) hits given the Maker2 annotation (Reference = Maker2 Input)
(53) TransRate - proportion of reference proteome covered by transcripts from the Maker2 annotation (Reference = Maker2 Input)
(54) TransRate Score
(55) TransRate Optimal Score
(56) RSEM-Eval Score

Category 11: Maker2-internal evaluation of Maker2 gene annotations (11) (see Supplemental Table S15A-B, and Supplemental Figure S14 A, C-E)

(57) Annotation Edit Distances (AED) output by Maker2

Category 12: Functional analysis of Maker2 gene annotations (9) (see Supplemental Table S16):

(58) Number of Genes with Ontology Term
(59) Number of Genes with UniProt hit(s)
(60) Number of Genes with Pfam domain
(61) Number of Genes with All 3 above (intersect)
(62) Number of Genes with $\geq$ 1 of 3 above (union)
(63) Number of Genes with Drosophila hit(s)
(64) Number of Genes with Anopheles hit(s)
(65) Percent Drosophila Proteome with Sciara hit(s)
(66) Percent Anopheles Proteome with Sciara hit(s)

Notes on the newer metrics above (as compared to previous genome evaluation sections):

- Categories 1-6 feature datasets and many metrics used in previous evaluations. Other metrics therein have similar objectives.
    - For example, Pilon was used to compute the number of bases in the genome that are confirmed (supported by evidence provided), which was represented as a percent of the expected genome size (to have the same denominator across assemblies).
    - As another example, Sniffles was used to report not only on the number of SVs, but also the sub-categories (i) translocations (proportional to mis-assemblies) and (ii) other (deletions, insertions, duplications, inversions).
    - Long read alignment and MAPQ scores were used to measure agreement with or support from the long read data, and the percent of reads that were involved in split alignments across multiple contigs was used as a measure proportional to mis-assemblies.

- Categories 7-8 essentially leveraged the RNA-seq data to evaluate the genome assembly directly.

- As with the various DNA-seq datasets (Illumina, PacBio, Nanopore), the percent of RNA-seq reads that map is a measure of completeness, but restricted to transcribed regions.
- Mapping the de novo assembled transcripts (see Section 4.3.1.1) with blast (-culling_limit 1) and taking the sum of bitscores is a measure of completeness and of agreement or quality (better alignments). Note that the bitscore sum is used here, but the following metrics gave the same results: the percent of de novo transcripts that aligned, the percent of the total length of de novo transcripts that aligned, the percent of the total length of de novo transcripts that matched (i.e. not only aligned, but matched).

- Categories 9-12 transfer to each genome assembly the evaluation results obtained from evaluating products associated with each genome assembly.
  - Evaluating reference-guided transcriptomes that were guided by each genome assembly is, overall, an evaluation of how well each genome assembly was at guiding the transcriptome assembly given the same dataset and analysis pipeline.
    - The BUSCO and TransRate scores associated with a reference proteome were measures of completeness of the transcriptome assembly (and thereby transcribed regions of the genome assembly).
    - The other TransRate scores and the RSEM-eval score are measures of how well the RNA-seq data support the transcriptome assembly (and thereby the genome reference that guided its assembly).
  - Similar to evaluating reference-guided transcriptome assemblies, evaluating the Maker2 gene annotations that were built iteratively and independently on each genome (e.g. gene predictors were trained separately on each genome, RNA-seq data and homology evidence were aligned to each genome, etc) also reflects on the underlying genome assembly, and is important anyway since the genome and its gene annotation are a package deal.
  - Annotation Edit Distance measures agreement between annotated gene structures and overlapping evidence (de novo transcripts, reference-guided transcripts, homologous transcripts, homologous proteins) for the gene structures. See Maker2 papers and documentation for more information. Smaller AEDs are better than larger ones, 0 being best. We compared the number of genes (using the transcript with the best AED) or the cumulative number of transcripts in the annotation (y-axis) as a function of AED (x-axis). Higher accumulations of genes (or transcripts) at lower AEDs indicated higher overall agreement with the evidence. See Supplemental Figure S14.
  - For TransRate, the reference proteomes used are described in the TransRate analysis section (see Section 4.3.2.3). Either the entire set of proteins used as protein homology evidence input into Maker2 was used or a subset of those proteins limited to UniProt Arthropoda proteins (see Section 4.3.4.8 “Protein homology evidence”).

4.3.6 Dosage compensation analysis

Transcripts Per Million (TPM) and estimated read counts per transcript were quantified for each RNA-seq sample using Salmon (Patro et al. 2017).

```
salmon quant -i ${SIDX} -l A \
         -1 ${R1} \
         -2 ${R2} \
         -p 1 --validateMappings -o quants/${PRE}_quant
```

Reproducibility amongst replicates was determined by clustering the samples on log10(TPM+1), which showed the replicates of each stage and sex clustering together. Differential expression analyses were performed with EdgeR (Robinson et al. 2010).

```
x <- read.delim("quants/all-salmon-counts-for-
EdgeR.txt",row.names="Name")

group <- factor(c(rep("FE",3),rep("FL",2),rep("FP",2), rep("FA",2),
rep("ME",3),rep("ML",2),rep("MP",2),rep("MA",2)),
                levels = c("FE","FL","FP","FA","ME","ML","MP","MA"))
y <- DGEList(counts=x,group=group)
keep <- filterByExpr(y, min.count = 1, min.total.count=10,
min.prop=0.25)#; summary(keep)
y <- y[keep, , keep.lib.sizes=FALSE]
y <- calcNormFactors(y);
design <- model.matrix(~ 0 + group)
colnames(design) <- levels(group)
y <- estimateDisp(y, design, robust=TRUE)
fit <- glmQLFit(y,design, robust=TRUE)

conE <- makeContrasts(ME - FE, levels=design)
qlfE <- glmQLFTest(fit, contrast=conE)
conL <- makeContrasts(ML - FL, levels=design)
qlfL <- glmQLFTest(fit, contrast=conL)
conP <- makeContrasts(MP - FP, levels=design)
qlfP <- glmQLFTest(fit, contrast=conP)
conA <- makeContrasts(MA - FA, levels=design)
qlfA <- glmQLFTest(fit, contrast=conA)
```

4.3.7 Characterization of the Lambda Phage Insert Containing ScRTE

We characterized the repeat content of the 13.8 kb lambda phage insert sequence (called Escribá insert here) that contains two copies of the non-LTR retrotransposon named ScRTE (Escribá et al. 2011). The Escribá insert sequence was aligned to itself with BLAST to find direct and inverted repeats within it (see Figure 5E). It was also BLAST against the Canu scaffolds to determine the copy number over each base. The Escribá insert sequence was aligned to the entire NCBI nucleotide database and nonredundant protein databases to annotate it. Our Illumina 100 bp paired-end DNA dataset was aligned with Bowtie2 to indexes of the Canu scaffolds that were masked with the Escribá insert sequence followed by adding one copy of the Escribá insert sequence back in. RepeatMasker was used to classify repeats along the Escribá insert using the Arthropod repeat database. BLAST was used with our de novo repeat families from RepeatModeler to determine what percent of the top ten most highly abundant repeat sequences in the genome aligned to the Escribá insert by at least 75% across their lengths.

4.4 DNA modifications

Both PacBio SMRT and Oxford Nanopore single molecule sequencing technologies come with raw signal information that can be used to identify base modifications (Flusberg et al. 2010; Clark et al. 2012; Loman et al. 2015; Simpson et al. 2017; Rand et al. 2016; Suzuki et al. 2016) and even secondary structure formation (Sawaya et al. 2015; Urban et al. 2015). Since we had high coverage from PacBio, we used that dataset for site-specific modification calls in the assembly for 5mC, 4mC, and 6mA. We subsequently used those sites to learn sequence motifs associated with each modification. Our 2D read strand coverage from MinION datasets was too low to reliably perform methylation calls at specific sites. Thus, to make use of the low coverage nanopore data, we used Nanopolish (Simpson et al. 2017) to learn kmer models specific to the *Sciara* dataset and determined kmers with observed shifted signal distributions compared to the expected models from Oxford Nanopore. Such a difference between models for a given kmer can arise from the effects of modifications within a subset of observed kmers. The set of kmers determined to have different means than the expected model were used to find sub-motifs within them to compare to the set of motifs obtained by PacBio. For both PacBio and MinION analyses, motifs were analyzed only for data from contigs in the Primary assembly labeled as Arthropoda in the blobtools analysis.

Regarding the detection of DNA modifications in the signal level for both PacBio and Nanopore, we note that reads were mapped to the full assembly that contained Primary, Associated, and Bacterial contigs to avoid forcing incorrect mappings using the following logic. The Associated Contigs are essentially small contigs that represent alternate versions of sequences in the Primary contigs. When the “correct” sequence to align to is not present, but a similar sequence is, the reads from the “correct” sequence will align to the wrong-but-similar sequence with some discrepancies, such as SNPs, indels, or split aligning. In our case, if the Associated Contigs are not present during alignment, the reads that would map to them can be “forced” to map to similar regions in the Primary contigs instead. After reads are aligned, the program(s) interested in DNA modifications then align the signal level in each read to the genome where the read mapped, and since the reads are error-prone, the genome sequence is often assumed to be the true sequence (not the read). Anomalies in the signal over each base in the genome sequence are flagged as potential DNA modificatons. Reads that are force-aligned to “incorrect” places will inevitably have anomolous signal over the region(s) of variation that may act like false positive signals for DNA modifications. To avoid this, we included all Primary, Associated, and Bacterial contigs. The bacterial sequences we know to be present in the datasets will align to the Bacterial Contigs. More importantly, the read sequences that are more similar to Associated Contig sequences will be attracted to the Associated Contigs rather than force-aligned to Primary contigs. As a result, the anomalous deviations from the expected signal in the effected regions on the Primary Contigs are less likely to be from these issues. We then further analyzed only Primary Contigs that were defined as related to Arthropods/Dipterans.

4.4.1 PacBio Analysis

4.4.1.1 PacBio Modification Prediction

PacBio reads were aligned in the presence of the entire unfiltered assemblies (all primary, associated, and bacterial contigs) to avoid forcing reads to align incorrectly. Pbalign 0.2.0.141024 (https://github.com/PacificBiosciences/pbalign) was used with BLASR v2 to map reads in bax.h5 files, outputting cmp.h5 files for each. Pbh5tools v0.8.0 (https://github.com/PacificBiosciences/pbh5tools) was used to merge and sort the mapped reads in cmp.h5 files, outputting a single cmp.h5 file that retained the raw signal information. "ipdSummary" from kineticsTools v0.6.0 (https://github.com/PacificBiosciences/kineticsTools) was used to predict base modifications across the Canu genome assembly. At the ipdSummary step, each contig was processed separately in parallel. The output from ipdSummary includes a CSV with kinetics statistics for each position and a GFF with DNA modification predictions (both deposited on NCBI for others to use). The CSV output for each contig was also used with AgIn (Suzuki et al. 2016; https://github.com/hacone/AgIn) to look at CpG methylation. AgIn makes the assumptions that CpG methylation status is the same on both strands and that neighboring CpGs are likely to have the same methylation status. These assumptions allow it to increase the effective coverage used to detect CpG methylation status by aggregating data from both strands and multiple neighboring CpG dinucleotides to determine methylation statuses of blocks of CpGs. Note methylation sites do tend to cluster in our dataset (see spacing analysis), which supports these assumptions.

```
## Align
pbalign $BAX $REF $OUTPRE.cmp.h5 --forQuiver --tmpDir $TMPDIR --nproc
$THREADS --metrics
DeletionQV,IPD,InsertionQV,PulseWidth,QualityValue,MergeQV,Substitution
QV,DeletionTag

## Merge alignments into single cmp.h5 file
cmph5tools.py merge --outFile ${MERGEDCMP} ${CMPDIR}/*.cmp.h5

## Sort merged alignments
cmph5tools.py sort --deep ${IN_CMP} --tmpDir ${TMP_SORT}

## Predict base modifications on each contig (command applied
separately for each contig)
ipdSummary ${MERGED_SORTED_CMPH5} -v -v -v --reference ${CANU_ASM} --
identify m6A,m4C,m5C_TET --methylFraction --gff basemods.gff --csv
kinetics.csv --pvalue 0.01 --minCoverage 3 --methylMinCov 10 --
identifyMinCov 5 -j ${THREADS} --maxAlignments 1000000 --ms_csv
multisite.csv --bigwig ipd.bigWig --refContigs $contigName

## A minority of contigs have a window or windows that causes the
algorithm to not finish the classification/identification step. These
windows were found through iteration and processed separately to skip
the classification step in the following way:

# Windows that have no issues are processed as usual with the
identification step
ipdSummary ${INPUT} -v -v -v --identify m6A,m4C,m5C_TET --
methylFraction --gff basemods-identified.gff --csv kinetics-
identified.csv --bigwig ipd-identified.bigWig --reference ${REF} --
pvalue 0.01 --minCoverage 3 --methylMinCov 10 --identifyMinCov 5 -j
${THREADS} --maxAlignments 1000000 --referenceWindows ${REFWINDOWS}

# Windows that with issues that cause the algorithm to freeze are
processed without identification step
```

```
ipdSummary ${INPUT} -v -v -v --gff basemods-unidentified.gff --csv
kinetics-unidentified.csv --bigwig ipd-unidentified.bigWig --reference
${REF} --pvalue 0.01 --minCoverage 3 --methylMinCov 10 --identifyMinCov
5 -j ${THREADS} --maxAlignments 1000000 --referenceWindows
${PROBLEM_REFWINDOWS}

# AgIn was also used with author-recommended settings for P5C3
chemistry on the kinetics.csv files for each contig to predict
methylated/unmethylated blocks of CpGs
/path/to/AgIn/target/dist/bin/launch -i kinetics.csv -f $REF -o
${OUT_PREFIX}-b P5C3_HdrR -g -0.88 -l 40 -c predict
```

4.4.1.2 DNA sequence motifs found near sites of predicted DNA modifications

Sequence motifs associated with PacBio modifications were searched for in two ways: an enriched kmer approach as well as using MEME (Bailey and Elkan 1994). For all analyses on modifications, we used only modifications from primary contigs labeled as Arthopoda in the Blobtools analysis to ensure that we were working with *Sciara* sequences.

4.4.1.3 Kmers enriched for DNA modifications

For each modification type, we looked for the dimer and trimer that were most enriched in predicted modifications with respect to their frequency in the genome sequence. We also generated motifs from all 7-mers that were enriched with predicted modifications for more sequence context. Kmer enrichment was determined by comparing observed and expected kmer frequencies as described below:

```
Let:
K = kmer size chosen as 2, 3, 4, 5, 6, or 7

B = base of interest that can be modified; either A or C

J = position where base B occurs in kmer (i.e. where a modification may
occur on B)

S = set of all kmers size K that have base B in position J

F_background = the expected background frequency of each kmer in S (for
a given set S) obtained by counting the number of occurrences of each
kmer in the genome sequence from both strands (as either strand can be
modified).

P_background = the expected background proportions of each kmer in
F_background obtained from dividing the frequency of each kmer in
F_background by the sum of the frequencies of all kmers in
F_background.

F_modifications = the observed modification frequency of each kmer in S
obtained by counting the number of times it was classified as modified
in the genome sequence on either strand.

P_modifications = the observed modification proportions of each kmer in
F_modifications obtained by dividing each the frequency of each kmer in
F_modifications by the sum of all kmer frequencies in F_modifications.
```

For a given set, **S**, that contains the subset of all kmers of size **K** with a given position, **J**, where the target base **B** occurs (e.g. C for mC or A for 6mA), we counted the number of times each kmer in S appeared in the genome on both strands (**F_background**), and defined the proportion of F_background that each kmer count made up as described above (**P_background**). Then for each kmer in S, we counted the subset of occurences in the genome on both strands where base B at position J of the kmer was determined to be modified (**F_modifications**), and defined the proportion of F_modifications that each kmer count made up as described above (**P_modifications**). We tested whether the observed proportions of the kmers with modifications to base B at position J (P_modifications) differed from the expected proportions (P_background). If modifications occurred with kmers at random, then the proportions of kmers in the set of kmers that are modified should be the same as the background proportions of those kmers. If DNA modifications occur on some kmers more than others, then the proportions of each kmer in the modified set will differ from background, and it would be possible to determine which kmers are over- and under-represented in the modified set. We used a Chi-square goodness of fit test and used the Chi-square standardized residuals that are interpreted similar to z-scores to determine which kmers occurred at much higher frequencies in F_modifications than expected given the background frequencies from F_background:

```
Standardized Residual for a kmer x
      = ((Observed_x-Expected_x)/square_root(Expected_x))
```

For each modification type, we determined the most prevalent dimers and trimers. Specifically, we focused on the set of dimers with the target base B at position J=1 and the set of trimers with the target base B at position J=2. For each modification type, we also filtered the sets of 7-mers with the target base B at position J=3 for enriched kmers using P-value and residual value cutoffs ($p < 0.000001$, residual > 50). The enriched 7-mers, all with base B at position J=3, were used with WebLogo (Crooks et al. 2004) to construct a position weight matrix (PWM) motif using the frequencies at which the kmers occurred (weighted) as well as with uniform weighting, both yielding nearly identical results. These logos show what bases tend to be nearest neighbors to base B, and the results reflect the enriched dimers and trimers we found with slightly more context in each direction.

4.4.1.4 MEME motifs in the highest scoring sites

The 9 bp centered around the modified bases were extracted for the top 500 scoring modifications for each modification type. These were used with MEME 5.0.2 (Bailey and Elkan 1994) to identify motifs using a second order Markov model trained on the primary arthropod scaffolds.

```
fasta-get-markov -m 2 -dna ${ASM} > bg.txt

for M in m6A m5C m4C; do
      SEQS=${M}.top500.primary-AND-arthropod-labeled.9mers.fasta

      meme $SEQS -norand -dna -oc ${M}_w2-5_bgcanu -time 18000 -maxsize
      60000 -mod anr -nmotifs 10 -minw 2 -maxw 5 -minsites 2 -maxsites
      600 -wnsites 0 -bfile bg.txt
done
```

4.4.1.5 Enrichment/Depletion of DNA modifications in various genomic regions

We searched for relationships between modification sites and the following features:

1. Repeats as defined by the regions of the genome that were masked as repeats for annotation purposes.
2. Gene regions defined by our Maker2 protein-coding gene annotations. We looked at all gene locations as one set as well as partitioned the genes based on expression levels in our male embryo RNA-seq as quantified by Salmon (Patro et al. 2017):
    a. Not expressed (0 TPM)
    b. Lowly expressed – defined as the 50% of genes with lowest non-zero expression levels (bottom 50% of TPM values > 0).
    c. Highly expressed – defined as the 50% of genes with the highest non-zero expression levels (top 50% of TPM values > 0).
3. Exons as defined by our Maker2 annotations.
4. Introns as defined as the complement intervals with respect to exons inside gene regions defined by our Maker2 annotation.
5. Promoter regions as defined as the 500 bp upstream of genes on the positive strand or 500 bp downstream of genes on the negative strand for gene intervals defined in our Maker2 annotation. We looked at all promoter regions together as well as partitioned into expression levels as with gene bodies above.

For all features, BEDtools merge was used to ensure any interval was represented only once.

4.4.1.6 Binomial tests for higher or lower modification rates in genomic regions

We determined if the DNA modification rate of a given base B was enriched in various genomic features of interest using a binomial model. Specifically, we tested whether there was a higher frequency of modifications to the B bases in the regions of interest than expected by chance given the background rate in the genome. These numbers are reflected in Supplemental Tables S20A, C, D, and E. Regions were tested for enrichment of modifications to a given base using a binomial model the following way:

```
Let:
B = base that may or may not be modified (A or C).

N_B_genome = Number of times base B occurs in the genome on both
strands.

N_mod_genome = Number of sites in genome on both strands where base B
was determined to be modified (given desired cut-offs for considering
modifications).

P_genome = expected probability of base B being modified in the genome
             = N_mod_genome/N_B_genome

R = regions of interest - i.e. intervals from the genome sequence such
as gene locations.

N_B_R = Number of times base B occurs in R, the regions of interest, on
both strands.

N_mod_R = Number of sites in R on both strands where base B was
determined to be modified.
```

```
P_R = observed probability of base B being modified in R
        = N_mod_R/N_B_R

Binomial P-value that modifications are enriched in R with respect to
the background genome probability:
      Let:
      n = N_B_R
      k = N_mod_R
      p = P_genome
```

$$P\ Value = \Pr(X \geq k) = \sum_{k}^{n} \binom{n}{k} p^k (1-p)^{n-k}$$

```
Binomial P-value that modifications are depleted in R with respect to
the background genome probability:
      Let:
      n = N_B_R
      k = N_mod_R
      p = P_genome
```

$$P\ Value = \Pr(X \leq k) = \sum_{0}^{k} \binom{n}{k} p^k (1-p)^{n-k}$$

For a given modification to base B, we counted the number of base B in the genome on both strands (**N_B_genome**) and the number of times base B was modified on either strand (**N_mod_genome**) to get the expected background probability that base B is modified (**P_genome**). We then followed the same procedure for the subset of B bases in the given regions of interest to get the number of B bases and observed modification count therein (**N_B_R and N_mod_R**). We calculated the binomial p-value as above given these parameters to test if there were significantly more modifications to base B in the regions of interest than expected by chance given the background rate of modifications to base B across the genome.

Binomial results were supported by permutation tests the following way:

1. P_R (proportion of B bases modified in R) was calculated as described above
2. The regions of interest, R, were shuffled throughout the genome with BEDtools 1000 times, and the proportion of B bases modified in the shuffled regions was calculated the same way for each (P_R_shuff).
3. P-values testing if modifications were enriched in the regions of interest (R) were approximated to be the proportion of the 1000 P_R_shuff values that were as high or higher than the observed P_R value. Alternatively, p-values testing if modifications were depleted in R were approximated to be the proportion of the 1000 P_R_shuff values that were as small or smaller than the observed P_R value.

4.4.1.7 Binomial tests for enrichment/depletion of DNA modifications in genomic regions

We also used binomial statistics to look at the problem a different way. We determined if the DNA modifications were enriched (or depleted) in various genomic features of interest by testing whether there was a higher frequency of modifications to the B bases in the regions of interest than expected by chance given the prevalence of the regions of interest in the genome. These numbers are reflected in Supplemental Tables S20B and S20F as well as Supplemental Figure S15B. The tables and figures report the expected vs observed percents to simplify the interpretation.

Regions were tested for enrichment of modifications to a given base using a binomial model the following way:

```
Let:
B = target base

G = total number of bases in the genome or assembly.

R = total number of bases in the regions of interest.

N_G = total number of bases in the genome or assembly.

N_R = total number of bases in the regions of interest.

p = proportion of bases in the genome that are in regions of interest
    = N_R/N_G

n = number of modifications in the genome

k = number of modifications in the regions of interest


Binomial P-value that modifications in G are enriched in R given n
total modifications in G, k modifications in R, and the probability, p,
of randomly being in R:
```

$$P\,Value = \Pr(X \geq k) = \sum_{k}^{n} \binom{n}{k} p^k (1-p)^{n-k}$$

```
Binomial P-value that modifications in G are depleted in R given n
total modifications in G, k modifications in R, and the probability, p,
of randomly being in R:
```

$$P\,Value = \Pr(X \leq k) = \sum_{0}^{k} \binom{n}{k} p^k (1-p)^{n-k}$$

4.4.1.8 Spacing between DNA modifications

BEDtools was used to obtain spacing distances between modified bases in subsets of the GFF file output by Kinetics Tools. For strand-agnostic spacing, “bedtools spacing” was used directly on the input. For strand-specific spacing, “bedtools spacing” was used on subsets of the input separated by which strand the modification was on, positive or negative. Then the two sets of strand-specific distances were combined. “shuffleBed” and “nucBed” were used to find N shuffled positions that occurred over the target base (e.g. A for 6mA or C for 4mC) in strand-agnostic and strand-specific manners, where N is the number of DNA modifications in the test set being compared. Commands used are below.

For strand-agnostic spacing (observed and expected/shuffled):

```
# Get spacing between given set of DNA modifications
bedtools spacing -i <( sortBed -i ${GFF} ) | awk '$10!="."
{a[$10]+=1}END{for (e in a) print e"\t"a[e]}' | sort -k1,1n > ${NAME}

# Get spacing between the same number of shuffled “modifications” over
the same base
## N = number of GFF entries for modified base
N=$( grep -c -v "^#" ${GFF} )
```

```
## Identify nucBed column to look at
  if [ $TBASE == A ]; then COL=12; elif [ $TBASE == C ]; then COL=13;
elif [ $TBASE == G ]; then COL=14; elif [ $TBASE == T ]; then COL=15; fi

## Shuffle 4*N entries, take only N shuffled features that correspond to
the target base
cat ${GFF} ${GFF} ${GFF} ${GFF} | bedtools shuffle -chrom -noOverlapping
-g ${G} -i - | sortBed -i - | nucBed -fi ${REF} -bed - -s | awk -v
"COL=$COL" '$1!~/^#/ && $COL>0 {OFS="\t"; print
$1,$2,$3,$4,$5,$6,$7,$8,$9}' | shuf -n ${N} | sortBed -i - >
${RANDOM_NAME}

## Get random spacing
bedtools spacing -i ${RANDOM_NAME} | awk '$10!="." {a[$10]+=1}END{for (e
in a) print e"\t"a[e]}' | sort -k1,1n > ${RANDOM_SPACING_NAME}
```

For strand-specific spacing (observed and expected/shuffled):

```
# Get spacing between given set of DNA modifications
awk '$7=="+"' ${GFF} | sortBed -i - > ${POS_GFF}
awk '$7=="-"' ${GFF} | sortBed -i - > ${NEG_GFF}

## Positive strand
bedtools spacing -i ${POS_GFF} | awk '$10!="." {a[$10]+=1}END{for (e in
a) print e"\t"a[e]}' | sort -k1,1n > ${POS_NAME}

## Negative strand
bedtools spacing -i ${NEG_GFF} | awk '$10!="." {a[$10]+=1}END{for (e in
a) print e"\t"a[e]}' | sort -k1,1n > ${NEG_NAME}

## Combine
cat ${POS_NAME} ${NEG_NAME} | awk '{a[$1]+=$2}END{for (e in a) print
e"\t"a[e]}' | sort -k1,1n > ${NAME}


# Get spacing between the same number of shuffled "modifications" over
the same base
## N = number of GFF entries for modified base
NPOS=$( grep -c -v "^#" ${POS_GFF})
NNEG=$( grep -c -v "^#" ${NEG_GFF})

## Identify nucBed column to look at
  if [ $TBASE == A ]; then COL=12; elif [ $TBASE == C ]; then COL=13;
elif [ $TBASE == G ]; then COL=14; elif [ $TBASE == T ]; then COL=15; fi

## POS STRAND
## ## Shuffle 4*N entries, take only N shuffled features that correspond
to the target base
cat ${POS_GFF} ${POS_GFF} ${POS_GFF} ${POS_GFF} | bedtools shuffle -chrom
-noOverlapping -g ${G} -i - | sortBed -i - | nucBed -fi ${REF} -bed - -s
| awk -v "COL=$COL" '$1!~/^#/ && $COL>0 {OFS="\t"; print
$1,$2,$3,$4,$5,$6,$7,$8,$9}' | shuf -n ${N} | sortBed -i - >
${POS_RANDOM_NAME}

## ## Get random spacing
bedtools spacing -i ${POS_RANDOM_NAME} | awk '$10!="." {a[$10]+=1}END{for
(e in a) print e"\t"a[e]}' | sort -k1,1n > ${POS_RANDOM_SPACING_NAME}

## NEG STRAND
```

```
## ## Shuffle 4*N entries, take only N shuffled features that correspond
to the target base
cat ${NEG_GFF} ${NEG_GFF} ${NEG_GFF} ${NEG_GFF} | bedtools shuffle -chrom
-noOverlapping -g ${G} -i - | sortBed -i - | nucBed -fi ${REF} -bed - -s
| awk -v "COL=$COL" '$1!~/^#/ && $COL>0 {OFS="\t"; print
$1,$2,$3,$4,$5,$6,$7,$8,$9}' | shuf -n ${N} | sortBed -i - >
${NEG_RANDOM_NAME}

## ## Get random spacing
bedtools spacing -i ${NEG_RANDOM_NAME} | awk '$10!="." {a[$10]+=1}END{for
(e in a) print e"\t"a[e]}' | sort -k1,1n > ${NEG_RANDOM_SPACING_NAME}

## Combine random spacing from both strands
cat ${POS_RANDOM_SPACING_NAME} ${NEG_RANDOM_SPACING_NAME} | awk
'{a[$1]+=$2}END{for (e in a) print e"\t"a[e]}' | sort -k1,1n >
${RANDOM_SPACING_NAME}
```

We observed approximately 10 bp periodicities in the distribution of distances between adjacent DNA modifications. To formally identify the periodicity, using the Fast Fourier Transform (FFT) provided in Python's Numpy package, we ran a discrete fourier transform (DFT) analysis on the distributions of distances between DNA modifications. Prior to computing the FFT, the data was first mean-subtracted and tapered with a Hanning window. The spectral power over 100 discrete frequency bins was then computed by squaring the modulus of the transformed data. For each set of modifications, we recorded the bp spacing periodicity (< 100 bp) with the maximum power. Spectral power was fairly monochromatic at the 10-bp periodicity across all subsets of the DNA modification datasets (all modified A, 6mA, all modified C, 4mC, 5mC) and was robust to various filtering strategies from using all sites to requiring 10X, 15X, 20X, or 25X coverage per strand and modification scores greater than 20. The python code for automating this can be found at: https://github.com/JohnUrban/fftDnaMods.

4.4.2 Orthogonal support for PacBio modification results using the MinION data

For the MinION analysis, we only used the subset of our MinION data generated using the MkI with the SQK-MAP006 kit and R7.3 70 bps 6mer pore model (libraries 14, 15, 16, 20, 21). We used Nanopolish (Simpson et al. 2017) to learn updated pore models given the data. Briefly, in MinION sequencing, the raw ionic current data is segmented at sites where the picoamp (pA) level shifts thus compressing the data into a sequence of "events", each of which includes a mean, standard deviation, and duration. Each event roughly corresponds to a stretch of 6 bases called a 6-mer. Oxford Nanopore has expected models of the mean pA level and standard deviation for each 6-mer that are used for base-calling. With Nanopolish, the basecalled MinION reads are first aligned to a reference, subsequently allowing the ionic current events to be aligned to 6-mers in the reference. The collection of events aligned over any site of a given 6-mer in the reference is used to update the models and re-align the events in an iterative process (Simpson et al. 2017). The final updated model and event distributions for a given 6-mer can then be compared to the original model, and differences may imply the presence of DNA modifications in a subset of the data. Conversely, there should not be differences (or only small differences) when performing this procedure on PCR amplified genomic DNA that would only contain canonical bases. We were interested in (i) testing whether short 2-5 bp motifs consistently arose in the 6-mers that differed from the ONT models, and (ii) looking at the event distributions from 6-mers suggested to be modified often in the PacBio analysis.

We compared the event distributions for each of the 4096 6mers in our *Sciara* dataset to the expected ONT kmer models extracted from the basecalled fast5 files using our Fast5Tools scripts, and to a MinION dataset generated from whole genome PCR on *E. coli* genomic DNA using the same kit and pore model (BioProject PRJEB13021; Run ERR1309547; www.ebi.ac.uk/ena; Simpson et al. 2017). As PCR was used for the latter dataset, the signal distributions for each kmer should closely match the expected ONT kmer model. BWA (Li and Durbin 2009) was used to align MinION reads. For our *Sciara* data, although reads were initially aligned to the unfiltered assembly (all primary, associated, and bacterial contigs) to avoid forcing incorrect alignments, only reads aligned to primary contigs annotated as Arthropoda were used for further analysis. Nanopolish (Simpson et al. 2017) was used to learn updated kmer models from the native *Sciara* and *E. coli* PCR MinION datasets. For mapping *E. coli* reads, the K12 MG1655 reference was used (Accession: NC_000913.3). Example commands for *Sciara* data:

```
G=assembly.fasta
R=reads.fasta
B=reads.bam
samtools faidx $G
bwa index $G
for lib in lib14  lib15  lib16  lib20  lib21; do
 nanopolish extract $lib/pass/ -o $lib.pass.fa -t 2d
 nanopolish extract $lib/fail/ -o $lib.fail.fa -t 2d
done

cat lib*pass.fa > $R   # Or cat lib*pass.fa lib*fail.fa > $R

bwa mem -M -x ont2d $G $R -t $T | samtools sort -T $lib.pass --threads
$T | samtools view -F 4 -q ${Q} -bSh - > $B

samtools index $B

nanopolish methyltrain --reads $R --bam $B --genome $G -t $T --models-
fofn=ont.alphabet_nucleotide.R7.fofn --train-kmers all --rounds=5 --
progress
```

MEME 5.0.2 (Bailey and Elkan 1994) was used with a second order Markov model trained on the primary arthropod scaffolds to find 3 bp, 6 bp, and 2-5 bp motifs from the sets of 6-mers that that differed from the expected ONT model mean by more than 0.7-1 pA for comparison to the MEME motifs learned from the PacBio results. When we tested this procedure on a native *E. coli* dataset (BioProject PRJEB7385; Run ERR1147227; file MAP006-1.tar; www.ebi.ac.uk/ena), 32 of 34 6-mers that had differences in the emission means >1 pA contained GATC, the known motif for adenine methylation. When we learned new models given the *Sciara* data using only the passing quality 2D reads, there were 196 6-mers in the template strand model and 108-111 6-mers in each of the two complement models that had means that differed by more than 1 pA from the ONT model. If the motifs from PacBio or from the MinION results were random, we did not expect to find shared motifs. If DNA modifications were present non-randomly in both datasets, we expected to find shared motifs.

```
## Trimer motifs
SEQS= # all 6mers with absolute difference > 0.7
      # or all 6mers without CG in them with absolute difference > 0.7
meme $SEQS -bfile canu01.m2.txt -dna -maxsize 60000 -mod zoops -nmotifs
10 -minw 3 -maxw 3 -minsites 96 -maxsites 400

## 6 bp motifs
SEQS= # all 6mers with absolute difference > 0.8; 6mer sequences were
padded with NNN on each side to allow flexibility in 6 bp windows

meme $SEQS -bfile canu01.m2.txt -dna -maxsize 60000 -mod anr -nmotifs 10
-minw 6 -maxw 6 -minsites 30 -maxsites 600

## 2-5 bp motifs
SEQS= # all 6mers with absolute difference > 1
meme $SEQS -bfile canu01.m2.txt -dna -maxsize 60000 -mod anr -nmotifs 20
-minw 2 -maxw 5 -minsites 2 -maxsites 600
```

We also visualized the final event distributions from Nanopolish to interrogate the motifs learned from the PacBio medication results. We selected 6mers defined by the PWM WebLogo motifs from enriched 7-mers in the PacBio analysis. We also selected 6-mers that were not enriched to visualize for comparison. We compared the event distributions from our *Sciara* data to the expected Oxford Nanopore model and MinION data generated from PCR of E. coli genomic DNA that used the same reagents, kit, MinION, and pore model (Simpson et al. 2017): BioProject PRJEB13021; Run ERR1309547; www.ebi.ac.uk/ena. Kmers shown to be enriched with modifications in the PacBio analysis of *Sciara* were expected to have shifts in the distribution whereas other kmers from *Sciara* and all kmers from the PCR E. coli dataset were expected to have the same distribution as the expected model.

4.4.3 Calculating observed:expected ratios of dimers and trimers

Observed:expected (O:E) ratios were computed on each Maker2-defined transcript that had a minimum length of 500 bp and at most 5% N bases. Genome-wide, O:E ratios were computed on all 500 bp bins with <5% N bases as defined by BEDtools.

The observed:expected ratio for dimers and trimers were computed the following way(s). The observed probability of base b in the genome was calculated simply as the number of b bases divided by the sum of all bases, B:

$$O(b) = b/B$$

The expected probability of a dimer with bases b1 and b2 was calculated as:

$$\begin{aligned} E(b1b2) &= O(b1)*O(b2) \\ &= (b1/B) * (b2/B) \\ &= (b1*b2)/(B^2) \end{aligned}$$

The observed probability of a dimer with bases ordered as b1 and b2 was calculated as the number of the specific dimer, b1b2, divided by the total number of dimers, D, which works out to be the total number of bases minus 1 (B-1):

$$\begin{aligned} O(b1b2) &= (b1b2)/D \\ &= (b1b2)/(B-1) \end{aligned}$$

Thus, the observed:expected ratio of a dimer was:

$$\begin{aligned} OE(b1b2) &= O(b1b2)/E(b1b2) \\ &= ((b1b2)/(B-1)) \ / \ ((b1*b2)/(B^2)) \\ &= ((B^2)/(B-1)) * (b1b2)/(b1*b2) \end{aligned}$$

The expected probabilities for trimers, b1b2b3, were calculated two ways, but the resulting distributions of observed:expected ratios were virtually the same. The first way assumes complete independence of the bases in the trimer:

$$\begin{aligned} E_1(b1b2b3) &= O(b1)*O(b2)*O(b3) \\ &= (b1/B) * (b2/B) * (b3/B) \\ &= (b1*b2*b3)/(B^3) \end{aligned}$$

The second way models in the observed dimer probabilities to determine the expected probability of a trimer, b1b2b3, given the observed probabilities of the b1b2 dimer and b3 monomer:

$$\begin{aligned} E_2(b1b2b3) &= O(b1b2)*O(b3) \\ &= (b1b2/(B-1)) * (b3/B) \\ &= (b1b2*b3)/((B-1)*B) \end{aligned}$$

The observed probability of a trimer with bases ordered as b1, b2, and b3 was calculated as the number of the specific trimer, b1b2b3, divided by the total number of trimers, T, which works out to be the total number of bases minus 2 (B-2):

$$\begin{aligned} O(b1b2b3) &= (b1b2b3)/T \\ &= (b1b2b3)/(B-2) \end{aligned}$$

Thus, the observed:expected ratios for trimers were calculated as:

$$OE_1(b1b2b3) = ((b1b2b3)/(B-2)) / ((b1*b2*b3)/(B^3))$$
$$= (B^3/(B-2)) / (b1b2b3/(b1*b2*b3))$$

Or:

$$OE_2(b1b2b3) = ((b1b2b3)/(B-2)) / ((b1b2*b3)/((B-1)*B))$$
$$= ((B*(B-1))/(B-2)) / (b1b2b3/(b1b2*b3))$$

Section 5: Software versions and Supplemental References

5.1 Software versions used

ABruijn v0.3b
https://github.com/fenderglass/ABruijn
Git commit: 250761bac5589d83eeb1e00e19ba7fe78cbc1738

ABYSS v1.9.0
https://github.com/bcgsc/abyss.git
Git commit: 21106a4d0ad4b550da6f5b77b00620e530ca5037

AgIn
git commit 22e347cbbc34280ee085acdf41761f401f864609
https://github.com/hacone/AgIn.git

ALE
(C) 2010 Scott Clark
https://github.com/JohnUrban/sciara-project-tools/tree/master/alenano

Augustus v3.2.2 (used with Maker, BUSCO evaluations, and with BUSCO for training)
http://bioinf.uni-greifswald.de/augustus/binaries/augustus-3.2.2.tar.gz

BAMtools v 2.4.1 (used with Augustus and BUSCO)
git://github.com/pezmaster31/bamtools.git

BEDtools v2.26.0
https://github.com/arq5x/bedtools2.git

BLAST v 2.2.30+ (for evaluations and used with Maker)
ftp://ftp.ncbi.nlm.nih.gov/blast/executables/blast+/2.2.30/

BLASR v2 (git commit 8cc8621, 2015-10-22)
https://github.com/PacificBiosciences/blasr

BlobTools v0.9.17
https://github.com/DRL/blobtools.git
383552447b2015620917aab8120699ae2d9d44be

Bowtie2 v2.2.9
https://sourceforge.net/projects/bowtie-bio/files/bowtie2/2.2.9/bowtie2-2.2.9-linux-x86_64.zip

BUSCO
v3.0.1 https://gitlab.com/ezlab/busco.git
ODB9: http://busco.ezlab.org/datasets/diptera_odb9.tar.gz

v.1.22 http://busco.ezlab.org/files/BUSCO_v1.22.tar.gz
http://busco.ezlab.org/files/arthropoda_buscos.tar.gz

BWA v 0.7.14-r1136
https://github.com/lh3/bwa.git
ee730f3832314fb70d369526275f2aadab064470

Canu v1.0, 1.1, 1.2 and several commits after v1.3 released
https://github.com/marbl/canu.git
4d07faca1d54852966e8ca9f4bbd4bf607b521d3

Detonate 1.11 (used RSEM-Eval for transcriptome comparisons)
http://deweylab.biostat.wisc.edu/detonate/detonate-1.11.tar.gz

DBG2OLC (last update: Jun 11, 2015)
Original: https://github.com/yechengxi/DBG2OLC.git
3e577397c0c2ad2a4b61da29be59572cef6e3086
JMU Version: I forked DBG2OLC and made/used a branch -- obtain by:
git clone -b split_reads_by_backbone_minimize_open_files
https://github.com/JohnUrban/DBG2OLC.git

Dfam_Consensus-20181026
- used with RepeatMasker/RepeatModeler
http://www.dfam.org/web_download/Current_Release/Dfam.hmm.gz

EdgeR 3.28.0
https://bioconductor.org/packages/release/bioc/html/edgeR.html

Exonerate v2.2.0 (used with Maker)
http://ftp.ebi.ac.uk/pub/software/vertebrategenomics/exonerate/exonerate-2.2.0-x86_64.tar.gz

Falcon v 0.7.3
git://github.com/PacificBiosciences/FALCON-integrate.git
c275aaac952ee1fd830dbe32ddc3bfb4e929fcb0

Fast5Tools
https://github.com/JohnUrban/fast5tools
c6e6026769457cbde57636f7044e288302d22946

FRC^{bam} v1.3.0
https://github.com/vezzi/FRC_align.git
5b3f53e01cb539c857fd4230ec9410d76220fe22

GeneMark-ES Suite v4.38 (used with Maker and for training)
http://topaz.gatech.edu/GeneMark/tmp/GMtool_72DIF/gm_et_linux_64.tar.gz

gffcompare v0.10.6
https://github.com/gpertea/gffcompare

HINGE
https://github.com/fxia22/HINGE.git
86db7bc630d2956031c4116739a7998f824738b9

HISAT2 v2.1.0 (for genome evaluations and StringTie assemblies)
https://github.com/infphilo/hisat2.git

HMMer v3.2.1 (for use with BUSCO and Maker)
http://eddylab.org/software/hmmer/hmmer.tar.gz

InterProScan v5.32-71.0

ftp://ftp.ebi.ac.uk/pub/software/unix/iprscan/5/5.32-71.0/interproscan-5.32-71.0-64-bit.tar.gz

KineticsTools v0.6.0
git commit da2286bbd5a6fb2f524c70c6c5616b9715317b83
https://github.com/PacificBiosciences/kineticsTools.git

LAP v1.1
http://www.cbcb.umd.edu/~cmhill/files/lap_release_1.1.zip

Maker v2.31.10
http://yandell.topaz.genetics.utah.edu/maker_downloads/AA48/FC36/CF23/C760F5B508D12D14AFA82FC83AE5/maker-2.31.10.tgz

Maligner
Original: https://github.com/LeeMendelowitz/maligner.git
Fork used: https://github.com/JohnUrban/maligner.git
Commit: 21b70bd7f8615942a65f746dc5934ddd430b32e4
I only changed a hard coded default to allow much longer molecules to align:
static int max_query_frags = 50000 ##Originally 50

MarginAlign
git commit cc28457352d3b9e65ecb46a04a45903ba3f55c61
https://github.com/benedictpaten/marginAlign.git

MaSuRCA v3.1.3
http://www.genome.umd.edu/masurca.html

MEME 5.0.2
http://meme-suite.org/doc/download.html

Megahit v1.0.5
https://github.com/voutcn/megahit.git
a851582f8a6e4ca9cbe41b946dc522336033adeb

Miniasm 0.2-r137-dirty
https://github.com/lh3/miniasm
17d5bd12290e0e8a48a5df5afaeaef4d171aa133

Minimap 0.2-r124-dirty
https://github.com/lh3/minimap
1cd6ae3bc7c7a6f9e7c03c0b7a93a12647bba244

Nanopolish 0.5.0
https://github.com/jts/nanopolish.git
methyltrain version 0.5.0 for R7 data

NSEG (used with RepeatModeler)
ftp://ftp.ncbi.nih.gov/pub/seg/nseg/

PBalign 0.2.0.141024
https://github.com/PacificBiosciences/pbalign

PBDAGCON
Git commit f19aed1668d6ace0ab3ab4eb0e1e7d81139492a8

https://github.com/PacificBiosciences/pbdagcon.git

PBJelly from PBSuite v15.8.24
https://sourceforge.net/p/pb-jelly/wiki/Home/

Platanus v1.2.4
http://platanus.bio.titech.ac.jp

Pbh5tools v 0.8.0
https://github.com/PacificBiosciences/pbh5tools
2679c17687a868690595ae0d9e856c2fead28ff9

Picard Tools 2.1.1
https://github.com/broadinstitute/picard/releases/tag/2.1.1

Pilon v1.18
https://github.com/broadinstitute/pilon/releases/download/v1.18/pilon-1.18.jar

Poreminion v 0.4.4
https://github.com/JohnUrban/poreminion
c6a1bc8d7cfc7da675fdd0979622c4e200a16657

Quiver - GenomicConsensus_v1.1.0 (pbcore-1.2.4, ConcensusCore 1.0.1)
https://github.com/PacificBiosciences/GenomicConsensus

RaCon
https://github.com/isovic/racon
c342fc65f6fd686e7975238c207fb6f3aea671d1

REAPR v1.0.18
ftp://ftp.sanger.ac.uk/pub/resources/software/reapr/Reapr_1.0.18.test_data.tar.gz

RECON v1.08 (used with RepeatModeler)
http://www.repeatmasker.org/RepeatModeler/RECON-1.08.tar.gz

RepBase Repeat Masker Edition 20181026
- used with RepeatMasker/RepeatModeler
https://www.girinst.org/repbase/

RepeatMasker v1.332 (used on its own and within Maker)
- version development-$Id: RepeatMasker,v 1.332 2017/04/17 19:01:11 rhubley Ex

RepeatModeler v1.0.11
http://www.repeatmasker.org/RepeatModeler/RepeatModeler-open-1.0.11.tar.gz

RM BLAST 2.2.28
- used with RepeatMasker/RepeatModeler
ftp://ftp.ncbi.nlm.nih.gov/blast/executables/rmblast/2.2.28/ncbi-rmblastn-2.2.28-x64-linux.tar.gz
ftp://ftp.ncbi.nlm.nih.gov/blast/executables/blast+/2.2.28/ncbi-blast-2.2.28+-x64-linux.tar.gz

Salmon 1.1.0
https://combine-lab.github.io/salmon

SAMtools   v1.3

https://github.com/samtools/samtools/releases/download/1.3/samtools-1.3.tar.bz2

SAMtool (HTSlib) v1.3
https://github.com/samtools/htslib/releases/download/1.3/htslib-1.3.tar.bz2

SciaraTools
https://github.com/JohnUrban/sciara-project-tools

SGA v0.10.14
https://github.com/jts/sga.git
2ab3eae4d3d58b8d0dea3d60f2684c1181049a8f

SMARTdenovo (2016-03-07):
commit 61cf13dcaed6bb561129b60eaa833fa9f976f9b1
https://github.com/ruanjue/smartdenovo

SNAP (Semi-HMM-based Nucleic Acid Parser) gene prediction tool
- used with Maker and training
http://korflab.ucdavis.edu/Software/snap-2013-11-29.tar.gz

Sniffles v1.0.8
commit 51a44a23b01b006cd1241540105376ec934f1512
https://github.com/fritzsedlazeck/Sniffles

Soapdenovo2 v2.04
https://github.com/aquaskyline/SOAPdenovo2.git
dd6a98ba19bb21c3513a46ad5047d08e57583ab0

SPAdes v3.8.0 (and BayesHammer)
http://spades.bioinf.spbau.ru/release3.8.0/SPAdes-3.8.0-Linux.tar.gz

StringTie v1.3.5
https://github.com/gpertea/stringtie

TransRate v1.0.3
https://bintray.com/artifact/download/blahah/generic/transrate-1.0.3-linux-x86_64.tar.gz

Tandem Repeats Finder (TRF) v 4.09
- used with RepeatMasker/RepeatModeler
http://tandem.bu.edu/trf/trf.download.html

Trimmomatic v0.32
http://www.usadellab.org/cms/?page=trimmomatic

Trinity r20140413p1
https://sourceforge.net/projects/trinityrnaseq/files/PREV_CONTENTS/previous_releases/trinityrnaseq_r20140413p1.tar.gz

Velvet 1.2.08
https://www.ebi.ac.uk/~zerbino/velvet/

5.2 Supplemental References:

Altschul SF, Gish W, Miller W, Myers EW, Lipman DJ. 1990. Basic local alignment search tool. *J Mol Biol* **215**: 403–410.

Armstrong MJ, Jin Y, Allen EG, Jin P. 2019. Diverse and dynamic DNA modifications in brain and diseases. *Hum Mol Genet* **28**: R241–R253.

Bailey TL, Elkan C. 1994. Fitting a mixture model by expectation maximization to discover motifs in biopolymers. *Proc Int Conf Intell Syst Mol Biol* **2**: 28–36.

Bankevich A, Nurk S, Antipov D, Gurevich AA, Dvorkin M, Kulikov AS, Lesin VM, Nikolenko SI, Pham S, Prjibelski AD, et al. 2012. SPAdes: A New Genome Assembly Algorithm and Its Applications to Single-Cell Sequencing. *J Comput Biol* **19**: 455–477.

Bao W, Kojima KK, Kohany O. 2015. Repbase Update, a database of repetitive elements in eukaryotic genomes. *Mob DNA* **6**: 11.

Bienz-Tadmor B, Smith HS, Gerbi SA. 1991. The promoter of DNA puff gene II/9-1 of Sciara coprophila is inducible by ecdysone in late prepupal salivary glands of Drosophila melanogaster. *Cell Regul* **2**: 875–88.

Bolger AM, Lohse M, Usadel B. 2014. Trimmomatic: a flexible trimmer for Illumina sequence data. *Bioinformatics* **30**: 2114–20.

Campbell MS, Holt C, Moore B, Yandell M. 2014. Genome Annotation and Curation Using MAKER and MAKER-P. *Curr Protoc Bioinforma* **48**: 4.11.1-39.

Chaisson MJ, Tesler G. 2012. Mapping single molecule sequencing reads using basic local alignment with successive refinement (BLASR): application and theory. *BMC Bioinformatics* **13**: 238.

Chakraborty M, Baldwin-Brown JG, Long AD, Emerson JJ. 2016. Contiguous and accurate de novo assembly of metazoan genomes with modest long read coverage. *Nucleic Acids Res* **44**: e147.

Chin C-S, Alexander DH, Marks P, Klammer AA, Drake J, Heiner C, Clum A, Copeland A, Huddleston J, Eichler EE, et al. 2013. Nonhybrid, finished microbial genome assemblies from long-read SMRT sequencing data. *Nat Methods* **10**: 563–9.

Chin C-S, Peluso P, Sedlazeck FJ, Nattestad M, Concepcion GT, Clum A, Dunn C, O'Malley R, Figueroa-Balderas R, Morales-Cruz A, et al. 2016. Phased diploid genome assembly with single-molecule real-time sequencing. *Nat Methods*.

Clark SC, Egan R, Frazier PI, Wang Z. 2013. ALE: a generic assembly likelihood evaluation framework for assessing the accuracy of genome and metagenome assemblies. *Bioinformatics* **29**: 435–43.

Clark TA, Murray IA, Morgan RD, Kislyuk AO, Spittle KE, Boitano M, Fomenkov A, Roberts RJ, Korlach J. 2012. Characterization of DNA methyltransferase specificities using single-molecule, real-time DNA sequencing. *Nucleic Acids Res* **40**: e29.

Consortium TU. 2019. UniProt: A worldwide hub of protein knowledge. *Nucleic Acids Res* **47**: D506–D515.

Crooks GE, Hon G, Chandonia J-M, Brenner SE. 2004. WebLogo: a sequence logo generator. *Genome Res* **14**: 1188–90.

Crouse H V., Gerbi SA, Liang CM, Magnus L, Mercer IM. 1977. Localization of ribosomal DNA within the proximal X heterochromatin of Sciara coprophila (Diptera, Sciaridae). *Chromosoma* **64**: 305–318.

DiBartolomeis SM, Gerbi SA. 1989. Molecular characterization of DNA puff II/9A genes in Sciara coprophila. *J Mol Biol* **210**: 531–40.

Dolezel J, Bartos J, Voglmayr H, Greilhuber J. 2003. Nuclear DNA content and genome size of trout and human. *Cytometry A* **51**: 127–8; author reply 129.

English AC, Richards S, Han Y, Wang M, Vee V, Qu J, Qin X, Muzny DM, Reid JG, Worley KC, et al. 2012. Mind the gap: upgrading genomes with Pacific Biosciences RS long-read sequencing technology. *PLoS One* **7**: e47768.

Escribá MC, Greciano PG, Méndez-Lago M, De Pablos B, Trifonov VA, Ferguson-Smith MA, Goday C, Villasante A. 2011. Molecular and cytological characterization of repetitive DNA

sequences from the centromeric heterochromatin of Sciara coprophila. *Chromosoma* **120**: 387–397.
Flusberg BA, Webster DR, Lee JH, Travers KJ, Olivares EC, Clark TA, Korlach J, Turner SW. 2010. Direct detection of DNA methylation during single-molecule, real-time sequencing. *Nat Methods* **7**: 461–465.
Foulk MS, Liang C, Wu N, Blitzblau HG, Smith H, Alam D, Batra M, Gerbi SA. 2006. Ecdysone induces transcription and amplification in Sciara coprophila DNA puff II/9A. *Dev Biol* **299**: 151–63.
Foulk MS, Waggener JM, Johnson JM, Yamamoto Y, Liew GM, Urnov FD, Young Y, Lee G, Smith HS, Gerbi SA. 2013. Isolation and characterization of the ecdysone receptor and its heterodimeric partner ultraspiracle through development in Sciara coprophila. *Chromosoma* **122**: 103–19.
Gabrusewycz-Garica N. 1964. Cytological and autoradiographic studies in Sciara coprophila salivary gland chromosomes. *Chromosoma* **15**: 312–44.
Gerbi SA. 1971. Localization and characterization of the ribosomal RNA cistrons in Sciara coprophila. *J Mol Biol* **58**: 499–511.
Ghodsi M, Hill CM, Astrovskaya I, Lin H, Sommer DD, Koren S, Pop M. 2013. De novo likelihood-based measures for comparing genome assemblies. *BMC Res Notes* **6**: 334.
Grabherr MG, Haas BJ, Yassour M, Levin JZ, Thompson DA, Amit I, Adiconis X, Fan L, Raychowdhury R, Zeng Q, et al. 2011. Full-length transcriptome assembly from RNA-Seq data without a reference genome. *Nat Biotechnol* **29**: 644–52.
Greciano PG, Ruiz MF, Kremer L, Goday C. 2009. Two new chromodomain-containing proteins that associate with heterochromatin in Sciara coprophila chromosomes. *Chromosoma* **118**: 361–376.
Hoff KJ, Stanke M. 2019. Predicting Genes in Single Genomes with AUGUSTUS. *Curr Protoc Bioinforma* **65**: e57.
Holt C, Yandell M. 2011. MAKER2: an annotation pipeline and genome-database management tool for second-generation genome projects. *BMC Bioinformatics* **12**: 491.
Hubley R, Finn RD, Clements J, Eddy SR, Jones TA, Bao W, Smit AFA, Wheeler TJ. 2016. The Dfam database of repetitive DNA families. *Nucleic Acids Res* **44**: D81–D89.
Hunt M, Kikuchi T, Sanders M, Newbold C, Berriman M, Otto TD. 2013. REAPR: a universal tool for genome assembly evaluation. *Genome Biol* **14**: R47.
Iyer LM, Zhang D, Aravind L. 2016. Adenine methylation in eukaryotes: Apprehending the complex evolutionary history and functional potential of an epigenetic modification. *BioEssays* **38**: 27–40.
Jain M, Fiddes IT, Miga KH, Olsen HE, Paten B, Akeson M. 2015. Improved data analysis for the MinION nanopore sequencer. *Nat Methods* **12**: 351–356.
Kajitani R, Toshimoto K, Noguchi H, Toyoda A, Ogura Y, Okuno M, Yabana M, Harada M, Nagayasu E, Maruyama H, et al. 2014. Efficient de novo assembly of highly heterozygous genomes from whole-genome shotgun short reads. *Genome Res* **24**: 1384–95.
Kamath GM, Shomorony I, Xia F, Courtade TA, Tse DN. 2016. HINGE: Long-Read Assembly Achieves Optimal Repeat Resolution. *bioRxiv*.
Kerrebrock AW, Srivastava R, Gerbi SA. 1989. Isolation and characterization of ribosomal DNA variants from Sciara coprophila. *J Mol Biol* **210**: 1–13.
Kim D, Paggi JM, Park C, Bennett C, Salzberg SL. 2019. Graph-based genome alignment and genotyping with HISAT2 and HISAT-genotype. *Nat Biotechnol* **37**: 907–915.
Kohli RM, Zhang Y. 2013. TET enzymes, TDG and the dynamics of DNA demethylation. *Nature* **502**: 472–479.
Koren S, Walenz BP, Berlin K, Miller JR, Bergman NH, Phillippy AM. 2017. Canu: Scalable and accurate long-read assembly via adaptive κ-mer weighting and repeat separation. *Genome Res* **27**: 722–736.
Korf I. 2004. Gene finding in novel genomes. *BMC Bioinformatics* **5**: 59.
Koutsovoulos G, Kumar S, Laetsch DR, Stevens L, Daub J, Conlon C, Maroon H, Thomas F, Aboobaker AA, Blaxter M. 2016. No evidence for extensive horizontal gene transfer in the

genome of the tardigrade Hypsibius dujardini. *Proc Natl Acad Sci U S A* **113**: 5053–8.
Kumar S, Jones M, Koutsovoulos G, Clarke M, Blaxter M. 2013. Blobology: exploring raw genome data for contaminants, symbionts and parasites using taxon-annotated GC-coverage plots. *Front Genet* **4**: 237.
Laetsch DR, Blaxter ML. 2017. BlobTools: Interrogation of genome assemblies. *F1000Research* **6**: 1287.
Langmead B, Salzberg SL. 2012. Fast gapped-read alignment with Bowtie 2. *Nat Methods* **9**: 357–9.
Li B, Fillmore N, Bai Y, Collins M, Thomson JA, Stewart R, Dewey CN. 2014. Evaluation of de novo transcriptome assemblies from RNA-Seq data. *Genome Biol* **15**.
Li D, Liu C-M, Luo R, Sadakane K, Lam T-W. 2015. MEGAHIT: an ultra-fast single-node solution for large and complex metagenomics assembly via succinct de Bruijn graph. *Bioinformatics* **31**: 1674–1676.
Li H. 2016. Minimap and miniasm: fast mapping and de novo assembly for noisy long sequences. *Bioinformatics* **32**: 2103–10.
Li H. 2018. Minimap2: Pairwise alignment for nucleotide sequences. *Bioinformatics* **34**: 3094–3100.
Li H, Durbin R. 2009. Fast and accurate short read alignment with Burrows-Wheeler transform. *Bioinformatics* **25**: 1754–60.
Li H, Handsaker B, Wysoker A, Fennell T, Ruan J, Homer N, Marth G, Abecasis G, Durbin R. 2009. The Sequence Alignment/Map format and SAMtools. *Bioinformatics* **25**: 2078–9.
Lin Y, Yuan J, Kolmogorov M, Shen MW, Chaisson M, Pevzner PA. 2016. Assembly of long error-prone reads using de Bruijn graphs. *Proc Natl Acad Sci U S A* **113**: E8396–E8405.
Loman NJ, Quick J, Simpson JT. 2015. A complete bacterial genome assembled de novo using only nanopore sequencing data. *Nat Methods* **advance on**.
Long HK, Blackledge NP, Klose RJ. 2013. ZF-CxxC domain-containing proteins, CpG islands and the chromatin connection. In *Biochemical Society Transactions*, Vol. 41 of, pp. 727–740.
Luo R, Liu B, Xie Y, Li Z, Huang W, Yuan J, He G, Chen Y, Pan Q, Liu Y, et al. 2012. SOAPdenovo2: an empirically improved memory-efficient short-read de novo assembler. *Gigascience* **1**: 18.
Mendelowitz LM, Schwartz DC, Pop M. 2015. Maligner: a fast ordered restriction map aligner. *Bioinformatics* **32**: 1016–1022.
Mok EH, Smith HS, DiBartolomeis SM, Kerrebrock AW, Rothschild LJ, Lange TS, Gerbi SA. 2001. Maintenance of the DNA puff expanded state is independent of active replication and transcription. *Chromosoma* **110**: 186–96.
Nikolenko SI, Korobeynikov AI, Alekseyev MA. 2013. BayesHammer: Bayesian clustering for error correction in single-cell sequencing. *BMC Genomics* **14 Suppl 1**: S7.
Pardue M Lou, Gerbi SA, Eckhardt RA, Gall JG. 1970. Cytological localization of DNA complementary to ribosomal RNA in polytene chromosomes of Diptera. *Chromosoma* **29**: 268–290.
Patro R, Duggal G, Love MI, Irizarry RA, Kingsford C. 2017. Salmon provides accurate, fast, and bias-aware transcript expression. *Nat Methods* **14**: 417–419.
Pertea M, Pertea GM, Antonescu CM, Chang TC, Mendell JT, Salzberg SL. 2015. StringTie enables improved reconstruction of a transcriptome from RNA-seq reads. *Nat Biotechnol* **33**: 290–295.
Quevillon E, Silventoinen V, Pillai S, Harte N, Mulder N, Apweiler R, Lopez R. 2005. InterProScan: protein domains identifier. *Nucleic Acids Res* **33**: W116-20.
Quinlan AR, Hall IM. 2010. BEDTools: a flexible suite of utilities for comparing genomic features. *Bioinformatics* **26**: 841–2.
Rand AC, Jain M, Eizenga J, Musselman-Brown A, Olsen HE, Akeson M, Paten B. 2016. Cytosine Variant Calling with High-throughput Nanopore Sequencing. *bioRxiv*.
Rasch EM. 2006. Genome size and determination of DNA content of the X chromosomes, autosomes, and germ line-limited chromosomes of Sciara coprophila. *J Morphol* **267**:

1316–25.
Rausch C, Hastert FD, Cardoso MC. 2020. DNA Modification Readers and Writers and Their Interplay. *J Mol Biol*.
Rieffel SM, Crouse H V. 1966. The elimination and differentiation of chromosomes in the germ line of sciara. *Chromosoma* **19**: 231–76.
Roach MJ, Schmidt SA, Borneman AR. 2018. Purge Haplotigs: allelic contig reassignment for third-gen diploid genome assemblies. *BMC Bioinformatics* **19**: 460.
Robinson MD, McCarthy DJ, Smyth GK. 2010. edgeR: a Bioconductor package for differential expression analysis of digital gene expression data. *Bioinformatics* **26**: 139–140.
Salzberg SL, Phillippy AM, Zimin A, Puiu D, Magoc T, Koren S, Treangen TJ, Schatz MC, Delcher AL, Roberts M, et al. 2012. GAGE: A critical evaluation of genome assemblies and assembly algorithms. *Genome Res* **22**: 557–67.
Sawaya S, Boocock J, Black MA, Gemmell NJ. 2015. Exploring possible DNA structures in real-time polymerase kinetics using Pacific Biosciences sequencer data. *BMC Bioinformatics* **16**: 21.
Sedlazeck FJ, Rescheneder P, Smolka M, Fang H, Nattestad M, von Haeseler A, Schatz MC. 2018. Accurate detection of complex structural variations using single-molecule sequencing. *Nat Methods* **15**: 461–468.
Simão FA, Waterhouse RM, Ioannidis P, Kriventseva E V, Zdobnov EM. 2015. BUSCO: assessing genome assembly and annotation completeness with single-copy orthologs. *Bioinformatics* **31**.
Simpson JT, Durbin R. 2010. Efficient construction of an assembly string graph using the FM-index. *Bioinformatics* **26**: i367-73.
Simpson JT, Wong K, Jackman SD, Schein JE, Jones SJM, Birol I. 2009. ABySS: a parallel assembler for short read sequence data. *Genome Res* **19**: 1117–23.
Simpson JT, Workman RE, Zuzarte PC, David M, Dursi LJ, Timp W. 2017. Detecting DNA cytosine methylation using nanopore sequencing. *Nat Methods* **14**: 407–410.
Smit A, Hubley R. 2008. RepeatModeler Open-1.0. http://www.repeatmasker.org.
Smit A, Hubley R, Green P. 2013. RepeatMasker Open-4.0. http://www.repeatmasker.org.
Smith-Unna R, Boursnell C, Patro R, Hibberd JM, Kelly S. 2016a. TransRate: reference-free quality assessment of de novo transcriptome assemblies. *Genome Res* **26**: 1134–44.
Smith-Unna R, Boursnell C, Patro R, Hibberd JM, Kelly S. 2016b. TransRate: Reference-free quality assessment of de novo transcriptome assemblies. *Genome Res* **26**: 1134–1144.
Suzuki Y, Korlach J, Turner SW, Tsukahara T, Taniguchi J, Qu W, Ichikawa K, Yoshimura J, Yurino H, Takahashi Y, et al. 2016. AgIn: measuring the landscape of CpG methylation of individual repetitive elements. *Bioinformatics* **32**: 2911–9.
Ter-Hovhannisyan V, Lomsadze A, Chernoff YO, Borodovsky M. 2008. Gene prediction in novel fungal genomes using an ab initio algorithm with unsupervised training. *Genome Res* **18**: 1979–90.
Urban JM, Bliss J, Lawrence CE, Gerbi SA. 2015. Sequencing ultra-long DNA molecules with the Oxford Nanopore MinION. *bioRxiv* doi: 10.1101/019281.
Urban JM, Yamamoto Y, Kadota L, Lee A, Bliss JE, Smith HS, DiBartolomeis SM, Gerbi SA. 2016. The DNA puffs of Sciara coprophila before, during, and after developmentally programmed intrachromosomal DNA amplification. In *The genome and DNA puff sequences of the fungus fly, Sciara coprophila, and genome-wide methods for studying DNA replication.* (eds. J.M. Urban and S.A. Gerbi), Brown University, Providence.
Urnov FD, Liang C, Blitzblau HG, Smith HS, Gerbi SA. 2002. A DNase I hypersensitive site flanks an origin of DNA replication and amplification in Sciara. *Chromosoma* **111**: 291–303.
Vaser R, Sovic I, Nagarajan N, Sikic M. 2016. Fast and accurate de novo genome assembly from long uncorrected reads. *bioRxiv*.
Vezzi F, Narzisi G, Mishra B, Nagarajan N, Pop M, Vezzi F, Narzisi G, Mishra B, Lander E, Linton L, et al. 2012. Reevaluating Assembly Evaluations with Feature Response Curves: GAGE and Assemblathons ed. A. Rzhetsky. *PLoS One* **7**: e52210.
Walker BJ, Abeel T, Shea T, Priest M, Abouelliel A, Sakthikumar S, Cuomo CA, Zeng Q,

Wortman J, Young SK, et al. 2014. Pilon: an integrated tool for comprehensive microbial variant detection and genome assembly improvement. *PLoS One* **9**: e112963.
Wu N, Liang C, DiBartolomeis SM, Smith HS, Gerbi SA. 1993. Developmental progression of DNA puffs in Sciara coprophila: amplification and transcription. *Dev Biol* **160**: 73–84.
Ye C, Hill CM, Wu S, Ruan J, Ma Z (Sam). 2016. DBG2OLC: Efficient Assembly of Large Genomes Using Long Erroneous Reads of the Third Generation Sequencing Technologies. *Sci Rep* **6**: 31900.
Zerbino DR, Birney E. 2008. Velvet: algorithms for de novo short read assembly using de Bruijn graphs. *Genome Res* **18**: 821–9.
Zhu Q, Stöger R, Alberio R. 2018. A lexicon of DNA modifications: their roles in embryo development and the germline. *Front Cell Dev Biol* **6**: 24.
Zimin A V, Marçais G, Puiu D, Roberts M, Salzberg SL, Yorke JA. 2013. The MaSuRCA genome assembler. *Bioinformatics* **29**: 2669–77.
